# Diazomethyl-λ^3^-iodane meets aryne: dipolar cycloaddition and C-to-N iodane shift leading to indazolyl-λ^3^-iodanes†

**DOI:** 10.1039/d5sc00266d

**Published:** 2025-04-02

**Authors:** Shinya Otsuki, Kazuya Kanemoto, Daniel Carter Martos, Eunsang Kwon, Joanna Wencel-Delord, Naohiko Yoshikai

**Affiliations:** a Graduate School of Pharmaceutical Sciences, Tohoku University Sendai 980-8578 Japan kazuya.kanemoto.a1@tohoku.ac.jp naohiko.yoshikai.c5@tohoku.ac.jp; b Laboratoire d'Innovation Moléculaire et Applications (LIMA, UMR CNRS 7042), Université de Strasbourg/Université de Haute Alsace, ECPM 67087 Strasbourg France; c Research and Analytical Center for Giant Molecules, Graduate School of Science, Tohoku University Sendai 980-8578 Japan; d Endowed Research Laboratory of Dimensional Integrated Nanomaterials, Graduate School of Science, Tohoku University Sendai 980-8578 Japan; e Institute of Organic Chemistry, JMU Würzburg Am Hubland Würzburg Germany

## Abstract

Diazomethyl-λ^3^-iodanes have recently emerged as carbyne equivalents in organic synthesis, enabling the construction of multi-substituted carbon centers through strategic sequential activation of the diazo and iodane functional groups. Distinct from such reaction modes, we report here on the reactivity of diazomethyl-λ^3^-iodanes as iodane-bound 1,3-dipoles toward arynes. Equipped with bis(trifluoromethyl)benzyl alcohol-based benziodoxole (BX) moiety, diazomethyl-λ^3^-iodanes undergo annulation with arynes generated from *ortho*-silylaryl triflates and cyclic diarylhalonium salts, resulting in indazolyl-λ^3^-iodanes through [3 + 2] cycloaddition and carbon-to-nitrogen iodane migration. DFT calculations reveal that diazomethyl-BX prefers [3 + 2] cycloaddition with aryne over aryne insertion into the carbon–iodine(iii) bond (carboiodanation) and that the subsequent iodane migration proceeds through two consecutive 1,5-iodane shifts. The utility of these indazolyl-BXs as indazole-transfer agents has been demonstrated by α-functionalization of *N*,*N*-dimethylaniline derivatives.

## Abstract 2

Diazomethyl-λ^3^-iodanes act as iodane-bound 1,3-dipoles in reactions with arynes, forming indazolyl-λ^3^-iodanes *via* [3 + 2] cycloaddition and a 1,3-iodane shift. The resulting indazolyl-λ^3^-iodanes serve as novel oxidative indazole-transfer reagents.

## Graphical abstract

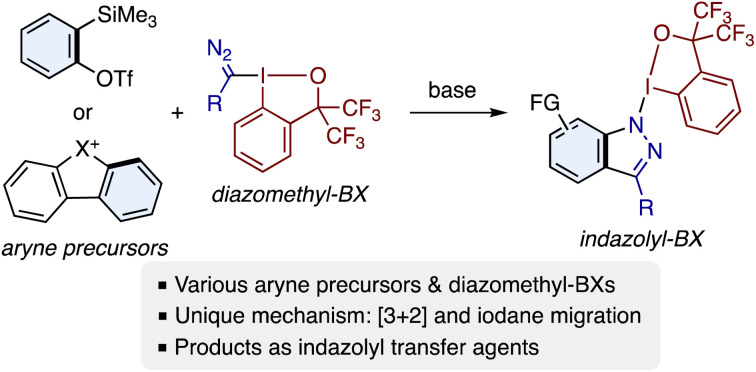

## Introduction

**Scheme 1:**
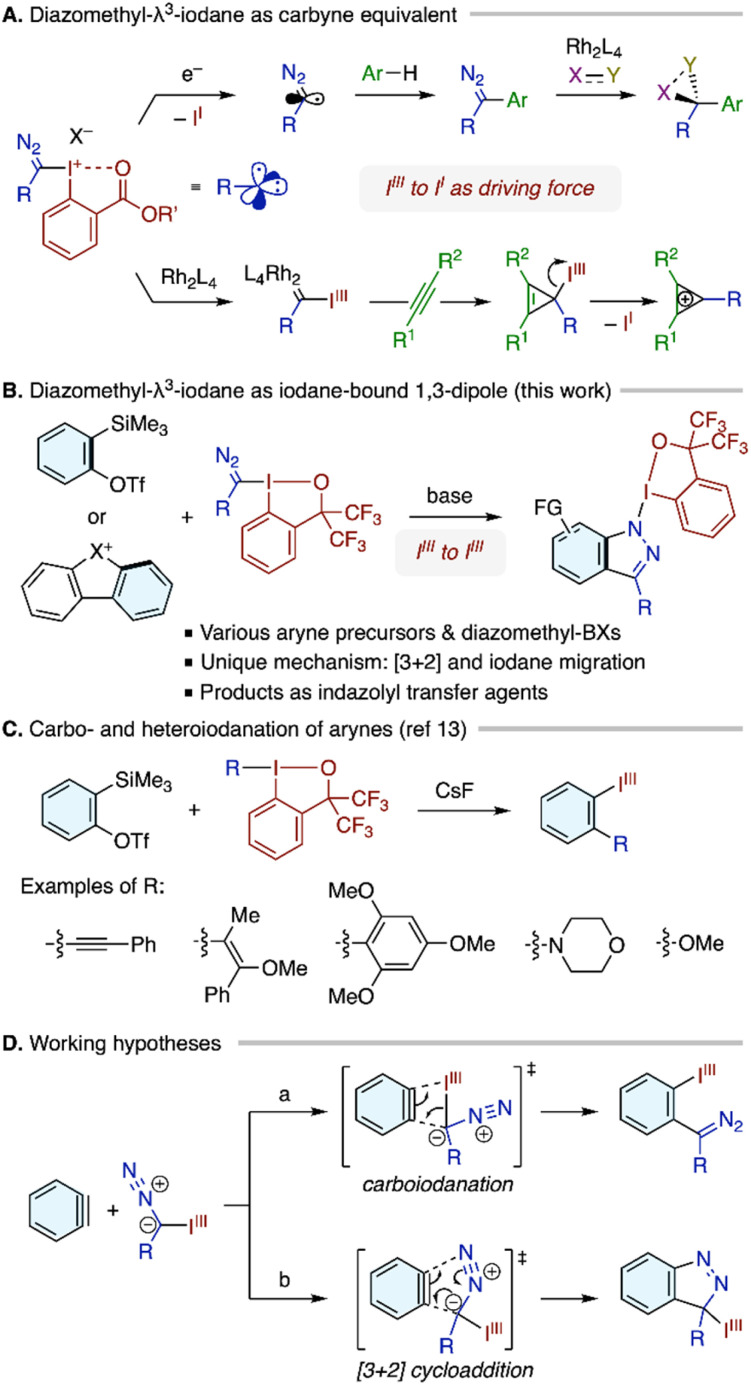
Background and summary of the present study.

Organo-λ^3^-iodanes, especially those equipped with cyclic and pseudocyclic frameworks, have attracted significant attention as valuable reagents for functional group transfer in organic synthesis.^1^ Within this category, diazomethyl-λ^3^-iodanes have emerged as notable carbyne surrogates, following the pioneering work of Suero and coworkers in 2018 (Scheme 1A).^2–4^ The diazo and iodane groups in these reagents can be strategically activated through two major, distinct mechanisms. In one approach, single-electron reduction of the C–I(iii) bond generates a diazomethyl radical, which can then undergo alkylation of (hetero)aromatic compounds^2^ and other radical transformations.^5^ The remaining diazo group in the product can be harnessed *via* rhodium-catalyzed carbene transfer reactions, such as X–H insertion and cyclopropanation, allowing for the divergent construction of multisubstituted tetrahedral carbons. Alternatively, initial activation of the diazo group with rhodium leads to the formation of an α-iodanyl Rh-carbene.^6^ The excellent leaving group ability of the iodanyl moiety allows this intermediate to act as an effective Rh-carbynoid, which exhibits dual carbene-carbocation reactivity toward alkenes and alkynes.

Drawing inspirations from these literature precedents and other reported reaction modes, we demonstrate here that diazomethyl-λ^3^-iodanes can act as iodane-bound 1,3-dipoles in reactions with arynes (Scheme 1B). Supported by a benziodoxole (BX) moiety derived from bis(trifluoromethyl)benzyl alcohol, these compounds participate in coupling with arynes, generated from appropriate precursors such as *ortho*-silylaryl triflate^7^ and cyclic diaryl λ^3^-chloranes/bromanes,^8,9^ resulting in the formation of indazolyl-λ^3^-iodanes as novel *N*-based λ^3^-iodane derivatives.^10^ The reaction is proposed to proceed through a concerted [3 + 2] cycloaddition between the diazomethyl group and the aryne,^11^ followed by a carbon-to-nitrogen shift of the iodane moiety. This proposal is substantiated by density functional theory (DFT) calculations, which suggest that the migration of the iodane moiety occurs through two consecutive 1,5-iodane shifts. The overall process represents a unique preparative approach to organo-λ^3^-iodanes that is distinct from simple ligand substitution on an iodine(iii) electrophile with a nucleophile.^12^ The indazolyl-λ^3^-iodanes proved to serve as reagents for the α-functionalization of *N*,*N*-dimethylaniline derivatives.

The discovery of the present reaction was guided by our recent study on the carbo- and heteroiodanation reactions of arynes, where organo-λ^3^-iodanes behave as organometallic-like nucleophiles toward the highly electrophilic C–C triple bond of aryne to afford *ortho*-functionalized aryl-λ^3^-iodanes (Scheme 1C).^13^ These reactions proved feasible with organobenziodoxoles having innately nucleophilic ligands such as alkynyl, electron-rich vinyl/aryl, amino, and alkoxy groups. Given this background and the intrinsic nucleophilic character of the diazomethyl carbon, we hypothesized that diazomethyl-BX may also undergo the addition across aryne to afford *ortho*-(diazomethyl)aryl-BX (Scheme 1D, path a). At the same time, an alternative hypothesis was drawn from the known reactivity of diazo compounds toward [3 + 2] cycloaddition with arynes (Scheme 1D, path b).^11^

## Results and discussion

The diazomethyl-λ^3^-iodanes requisite for the present study could be synthesized from acetoxybenziodoxole and the corresponding diazo compounds (see the ESI† for details). Subsequent exploration of their reactivity toward arynes revealed the relevance of our second hypothesis (Scheme 1D, path b). Thus, the reaction between 3-methoxybenzyne, generated by activation of *ortho*-trimethylsilylaryl triflate 1a with CsF, with diazoester derivative 2a proceeded smoothly in acetonitrile at room temperature, affording indazolyl-BX 3aa as a single regioisomer in 85% isolated yield (Table 1, entry 1). X-ray crystallographic analysis unambiguously established the molecular structure of 3aa, the indazole ring of which was likely constructed through regioselective [3 + 2] cycloaddition (*vide infra*).^14^ The reaction became somewhat sluggish in ethereal solvents such as DME and THF (entries 2 and 3) and did not take place at all in toluene (entry 4). Among other common fluoride activators, TBAF caused the formation of a complex mixture (entry 5), whereas KF/18-crown-6 proved as effective as CsF (entry 6). A high yield of 3aa could also be achieved by using 1a as the limiting reagent in combination with an excess amount (3 equiv.) of 2a (entry 7). In later studies, these “iodane-excess” conditions proved to work better for the majority of Kobayashi-type aryne precursors (*vide infra*). Notably, the diazomethylbenziodoxolone 2a′, derived from 2-iodobenzoic acid, failed to undergo an annulation reaction with 3-methoxybenzyne but produced a complex mixture, demonstrating the importance of the cyclic skeleton of the reagent (entry 8).

**Table 1:** Annulation of 3-methoxybenzyne and diazomethyl-BX 2a into iodazolyl-BX 3aa^a^

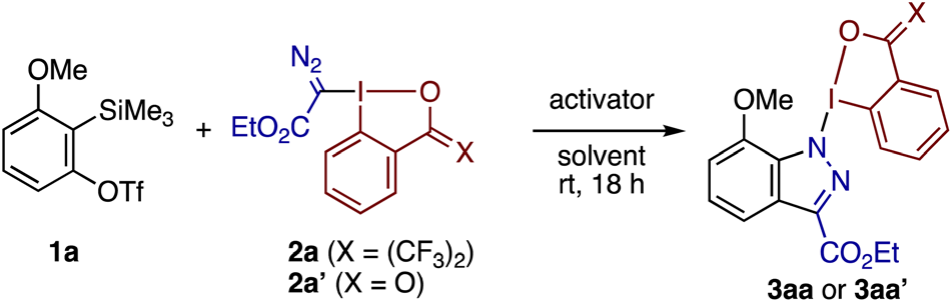			
Entry	Activator	Solvent	Yield^b^ [%]
1	CsF	MeCN	85^c^
2	CsF	DME	70
3	CsF	THF	65
4	CsF	Toluene	0
5	TBAF	THF	0
6	KF/18-crown-6	THF	80
7^d^	CsF	MeCN	91
8^e^	CsF	MeCN	0

a Unless otherwise noted, the reaction was performed using 0.2 mmol of 1a, 0.1 mmol of 2a, and 0.4 mmol of activator in a solvent at a concentration of 0.2 M.

b Determined by ^1^H NMR using 1,1,2,2-tetrachloroethane as an internal standard.

c Isolated yield.

d The reaction was performed using 0.1 mmol of 1a, 0.3 mmol of 2a, and 0.2 mmol of CsF.

e Reagent 2a′ was used instead of 2a.

Subsequently, we explored the scope of the indazolyl-BX synthesis from *ortho*-silylaryl triflates and diazomethyl-BXs (Table 2). Diazomethyl-BXs bearing benzyl and aryl ester moieties underwent the annulation with 3-methoxybenzyne to afford the corresponding products 3ab and 3ac in moderate to good yields. Likewise, diazomethyl-BXs bearing a variety of aryl ketone moieties participated in the reaction with 3-methoxybenzyne, furnishing the desired products 3ad–3ak in moderate yields. A series of unsubstituted and substituted arynes generated from the respective silylaryl triflate were capable of undergoing annulation with 2a, affording the corresponding indazolyl-BXs 3ba–3ha in moderate yields. The reaction between parent benzyne and 2a could be performed on a 1 mmol scale, albeit in somewhat diminished yield (see 3ba; 52% (1 mmol) *vs.* 62% (0.1 mmol)). Similar to 3-methoxybenzyne, 3-methoxy-5-halobenzynes reacted with exclusive regioselectivity, affording the corresponding regioisomers 3ca and 3da. The reaction of 1-naphthalyne displayed high regioselectivity (11 : 1), where the diazomethyl carbon was preferentially introduced to the 2-position of the naphthalene ring (see 3ga). Not unexpectedly, 4-methylbenzyne exhibited no regiochemical preference, producing an equimolar mixture of 3ha and its regioisomer. Unlike these cases, the reaction of 4,5-dimethoxybenzyne and 2a resulted in the free indazole derivative 3ia-H, presumably through the *in situ* loss of the iodanyl group upon [3 + 2] cycloaddition. Like this case, the modest yield and failure with Kobayashi aryne precursors are partly or fully ascribed to the decomposition of the desired indazolyl-BX under the reaction conditions (Fig. S2†). Given the generally higher yields obtained with cyclic diarylhalonium precursors (*vide infra*), we suspect that fluoride ion is responsible for this undesirable decomposition, while the exact mechanism of decomposition remains unclear. Additionally, some of the products also proved to undergo partial or full decomposition during silica gel chromatography purification.

**Table 2:** Scope of indazolyl-BX synthesis *via* annulation of arynes with diazomethyl-BXs^a^

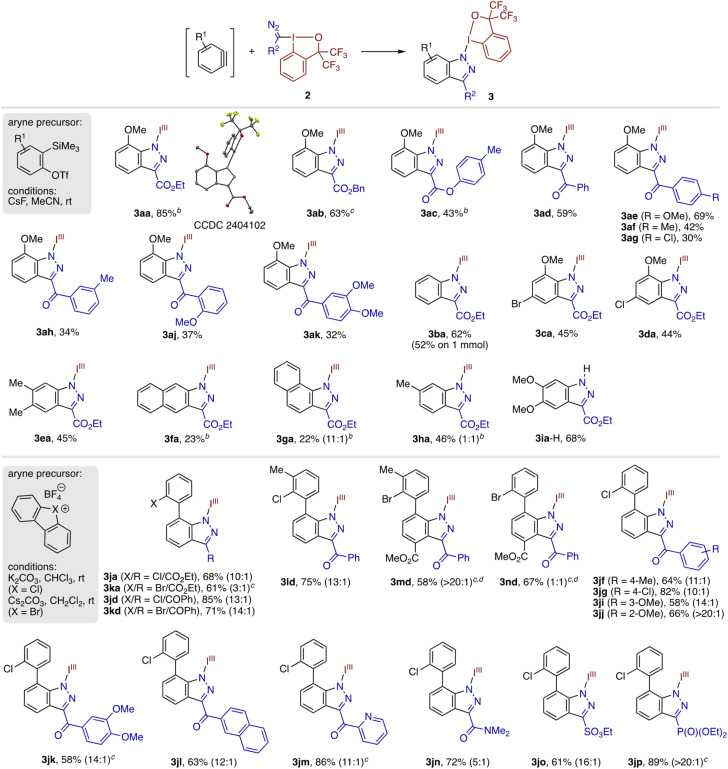

a Conditions for the reaction using *ortho*-trimethylsilylaryl triflate: aryne precursor (0.1 mmol), diazomethyl-BX (0.3 mmol), CsF (0.2 mmol), MeCN (0.1 or 0.2 M), rt, 18 h. Conditions for the reaction using cyclic diarylchloronium/bromonium salt: aryne precursor (0.05 mmol), diazomethyl-BX (0.1 or 0.15 mmol), K_2_CO_3_ (for X = Cl, 0.15 mmol) or Cs_2_CO_3_ (for X = Br, 0.15 mmol), CHCl_3_ (for X = Cl, 0.1 M) or CH_2_Cl_2_ (for X = Br, 0.1 M), rt, 18 h. Unless otherwise noted, isolated yields are shown. The ratio in the parentheses refers to the regioisomer ratio determined by ^1^H NMR analysis of the crude product.

b Aryne precursor (0.2 mmol), diazomethyl-BX (0.1 mmol), and CsF (0.4 mmol) were used.

c Yield determined by ^1^H NMR using 1,1,2,2-tetrachloroethane as an internal standard.

d Aryne precursor (0.05 mmol), diazomethyl-BX (0.1 mmol), Cs_2_CO_3_ (0.1 mmol), and THF (0.05 M) were used.

In light of the innately sensitive carbonyl groups in the diazomethyl-BX reagents, the present reaction appeared incompatible with many common methods of aryne generation that employ either a strong base or a nucleophilic organometallic reagent as an activator.^7,15^ In this respect, cyclic diaryl-λ^3^-chloranes and bromanes, capable of generating *ortho*-haloaryl-substituted arynes with the aid of mild base,^8,9^ appeared attractive as alternatives to *ortho*-trimethylsilylaryl triflates. To our delight, the present diazomethyl-BXs were found to engage successfully with these aryne precursors (Table 2). Thus, the reaction between cyclic diarylchloronium tetrafluoroborate and the diazoester reagent 2a took place smoothly in the presence of K_2_CO_3_ at room temperature, affording the hetero-biaryl-type indazolyl-BX 3ja in 68% yield and high regioselectivity (10 : 1), with C–C bond formation preferentially taking place at the distal position. The analogous diarylbromonium salt also took part in the reaction with 2a to give 3ka, albeit with diminished regioselectivity (3 : 1). Meanwhile, both the halonium salts reacted very smoothly with the diazoketone reagent 2d, furnishing the desired products 3jd and 3kd in good yields with high regioselectivity. An additional methyl substituent on the chloronium salt was well tolerated, affording the product 3ld with equally good yield and regioselectivity. More structurally complex aryne containing 2-bromo-3-methylphenyl and methoxycarbonyl groups at the 3- and 6-positions exhibited excellent regioselectivity (see 3md). In contrast, analogous aryne with 2-bromophenyl group afforded an equimolar mixture of 3nd and its regioisomer, highlighting significant influence of subtle structural change on the regioselectivity. The chloronium reagent displayed tolerance to a wide variety of diazomethyl-BXs, including those containing not only various ketones (3jf–3jm) but also amide (3jn), sulfonate ester (3jo), and phosphate ester (3jp), to furnish the desired biaryl-type products in good to high yields while displaying regioselectivity higher than 10 : 1 in most cases. In none of these reactions we observed oxidative decomposition of the product that could potentially be induced by the Cl(iii)/Br(iii) salt. Notably, the 2-pyridyl ketone, amide, sulfonate, and phosphate-containing reagents failed to produce annulation products with the Kobayashi-type aryne precursor. We also note in passing that the reaction between chloronium salt 1j and 2a could even be performed under mechanochemical conditions (30 Hz) to give the product 3ja in a moderate yield (60% by ^1^H NMR), whereas a mechanochemical reaction between Kobayashi precursor 1a and 2a was unproductive. These observations underscore the outstanding performance of the chloronium salt in the present reaction.

Upon establishing the scope of the cycloaddition, we briefly evaluated the arynophilicity of the present diazomethyliodane.^16^ Thus, competitive reactions of 2a using furan or benzyl azide as counterparts toward parent benzyne resulted in notable preference for 3aa over the cycloadduct of the counterpart (see the ESI†). This demonstrates that 2a is significantly more reactive than our previously reported organoiodane arynophiles (Scheme 1C), all of which were less arynophilic than furan.^13^

**Fig. 1:**
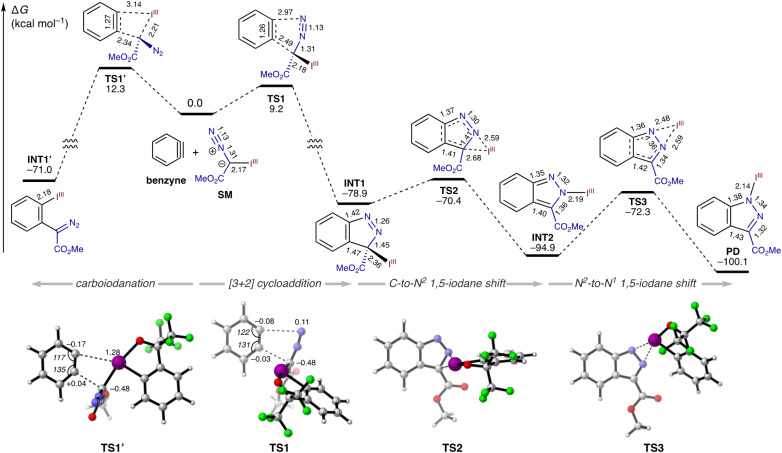
Computed energy profile for the reaction between diazomethyl-BX and benzyne (level: M06-2X/6-311++G(2df,2p)-SDD(for I)/SMD(THF)//M06-2X/6-31G(d)-SDD(for I)). Bond lengths are indicated in Å.

To gain insight into the mechanism of the present reaction, we explored our initial hypotheses (Scheme 1D) using DFT calculations (Fig. 1). The [3 + 2] cycloaddition between parent benzyne and model diazomethyl-BX (SM) proceeds *via* a concerted transition state TS1 with an activation energy of 9.2 kcal mol^−1^. This process is highly exergonic, leading to 3*H*-indazol-3-yl-BX (INT1) with a free energy change of −78.9 kcal mol^−1^. Notably, the alternative pathway, namely, the insertion of benzyne into the carbon–iodine(iii) bond, is also feasible through a four-centered transition state TS1′, which directly leads to *ortho*-diazomethylphenyl-BX (INT1′) with significant exergonicity. However, its activation energy (12.3 kcal mol^−1^) is distinctly higher than that of the [3 + 2] cycloaddition, consistent with the lack of observation of the expected carboiodanation product. Featuring a typically unstable C(sp^3^)–iodine(iii) bond, INT1 undergoes a facile 1,5-iodane shift (TS2, Δ*G*^‡^ = 8.5 kcal mol^−1^) from the C^3^-position to the N^2^-position of the indazole ring to afford 2*H*-indazol-2-yl-BX (INT2), which is more stable than INT1 by over 15 kcal mol^−1^. Finally, INT2 undergoes a second 1,5-iodane shift (TS3, Δ*G*^‡^ = 22.6 kcal mol^−1^) from the N^2^ position to the N^1^ position, leading to the experimentally relevant 1*H*-indazol-1-yl-BX (PD), with a finite but significant additional stabilization (*ca.* 5 kcal mol^−1^) from INT2.

The competitive transition states of [3 + 2] cycloaddition (TS1) and carboiodanation (TS1′) merit further discussion. Reflecting the nucleophilic nature of the diazomethyl carbon, both TS1 and TS1′ exhibit similar distortions in the benzyne segment, with a wider bond angle (131–135°) at the carbon accepting the nucleophilic diazomethyl carbon and a narrower bond angle (117–122°) at the other carbon. Notably, the degree of aryne distortion is slightly more pronounced in TS1′ than in TS1, which is also reflected in a more polarized C–C triple bond in TS1′, as indicated by the natural population analysis (NPA) charges (C1, +0.04; C2, –0.17 in TS1′ and C1, –0.03; C2, –0.08 in TS1). This pronounced aryne distortion and polarization in TS1′ may be attributed to the more electrostatic nature of the aryne–arynophile interaction in TS1′, due to the highly positive iodine(iii) center. The mode of aryne distortion in TS1 aligns with the perfect regioselectivity observed with 3-methoxybenzyne (see 3aa), whose distal carbon is distorted toward linearity to serve as a more electrophilic site.^17^ Our DFT calculations support this conjecture (Fig. S4†). The [3 + 2] cycloaddition between 3-methoxybenzyne and SM, following the experimentally observed regioselectivity, involves monotonously downhill potential energy surface without a discernible transition state. In contrast, a transition state leading to the alternative regioisomer is located approximately 3 kcal mol^−1^ higher in energy. Meanwhile, 3-(2-halophenyl)benzynes, generated from cyclic diarylchloronium and bromonium salts, have been shown to display slightly higher linearly at the proximal carbon, indicating its intrinsic inclination toward accepting nucleophilic attack.^8*c*^ Nevertheless, the observed regioselectivity for these arynes can be rationalized by steric factors, favoring the attack of the bulky α-iodanyl diazomethyl carbon to the distal position of 3-arylaryne. The comparison of two possible transition states for the [3 + 2] cycloaddition between 3-(2-chlorophenyl)benzyne and SM was in accordance with this regioselectivity (Fig. S5†).

**Scheme 2:**
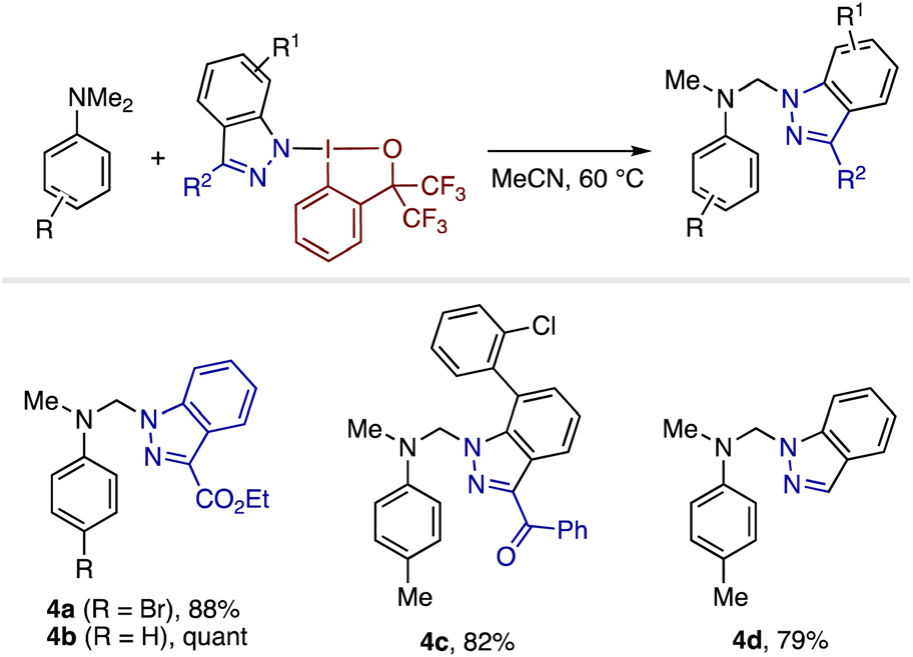
Use of indazolyl-BXs as group transfer agents for the functionalization of *N*,*N*-dimethylaniline derivatives.

Inspired by the seminal studies of Zhdankin and Kiyokawa/Minakata on the use of nitrogen-bound benziodoxolones as reagents for radical C(sp^3^)–H functionalization,^18^ we identified the ability of the present indazolyl-BXs as indazole-transfer agents (Scheme 2). Thus, the reaction between 4-bromo-*N*,*N*-dimethylaniline and 3ba in MeCN at 60 °C resulted in the installation of the indazolyl group at the *α*-position, affording the product 4a in 88% yield.^18^ Analogous reactions took place smoothly using different dimethylanilines and indazolyl-BXs, furnishing the corresponding products 4b and 4c in high yields. Besides the indazolyl-BXs accessible by the present [3 + 2] reaction, parent indazolyl-BX (3x), which can be synthesized independently from indazole and benziodoxole chloride under basic conditions (see the ESI†), also proved to participate in the same reaction, furnishing the product 4d in good yield. As this reaction was significantly shut down by the addition of TEMPO, we presume that the reaction proceeds through a mechanism involving radical processes initiated by single-electron transfer from *N*,*N*-dimethylaniline to indazolyl-BX (see Scheme S1† for a proposed reaction pathway).

## Conclusions

In summary, we have demonstrated the reactivity of diazomethyl-λ^3^-iodanes supported by benziodoxole moiety (diazomethyl-BXs) as iodane-bound 1,3-dipole toward arynes, leading to the formation of indazolyl-λ^3^-iodanes through [3 + 2] cycloaddition and carbon-to-nitrogen iodane migration. The present reaction proved compatible with Kobayashi-type *ortho*-trimethylsilylaryl triflate and cyclic diaryl-λ^3^-chloranes/bromanes as aryne precursors. The latter displayed particularly excellent tolerance to a broad range of diazomethyl-BXs, which represents a unique merger of two distinct and complementary hypervalent halogen reagents in a single transformation. The DFT calculations demonstrated the preference for the [3 + 2] cycloaddition over the alternative carboiodanation pathway as well as the stepwise nature of the subsequent iodane shift event. The resulting indazolyl-BXs proved to serve as a new class of N-based iodane group transfer agents,^10,19^ as demonstrated by the C(sp^3^)–H functionalization of *N*,*N*-dimethylanilines. The present findings are also notable considering that, despite the extensive use of azole heterocycles as stabilizing elements in cyclic and pseudocyclic iodane compounds,^20^ λ^3^-iodanes with unsupported azolyl-iodine(iii) bonds remain relatively scarce and their reactivity less explored.^21^ Further investigations into the merger of aryne chemistry and nucleophilic hypervalent iodine chemistry are ongoing in our laboratories.

## Data availability

The data supporting this article have been included as part of the ESI.† Crystallographic data for 3aa has been deposited at the CCDC under 2404102 and can be obtained from the joint Cambridge Crystallographic Data Centre and Fachinformationszentrum Karlsruhe Access Structures service.

## Author contributions

K. K. and N. Y. conceived the project. S. O. performed experiments with the assistance of D. C. M. E. K. performed X-ray crystallographic analysis. N. Y. performed DFT calculations. N. Y. wrote the original draft. K. K., J. W. -D., and N. Y. reviewed and edited the manuscript. N. Y. administered the project.

## Conflicts of interest

There are no conflicts to declare.

## Supplementary Material

SC-OLF-D5SC00266D-s001

SC-OLF-D5SC00266D-s002
