# Supplementary material for: Diazomethyl-λ3-iodane meets aryne: dipolar cycloaddition and C-to-N iodane shift leading to indazolyl-λ3-iodanes
Source: Chem Sci. 2025 Apr 2;16(18):8053–9. doi: 10.1039/d5sc00266d (PMC11976445; doi:10.1039/d5sc00266d)

Supplementary Information (SI) for Chemical Science.
This journal is © The Royal Society of Chemistry 2025

Supporting Information

Diazomethyl-λ^3-iodane Meets Aryne: Dipolar Cycloadditon and C-to-N Iodane Shift Leading to Indazolyl-λ^3-iodanes

Shinya Otsuki,[a] Kazuya Kanemoto,[a]* Daniel Carter Martos,[b] Eunsang Kwon,[c,d] Joanna Wencel-Delord,[b,e] and Naohiko Yoshikai[a]*

[a] Graduate School of Pharmaceutical Sciences
Tohoku University
Sendai 980-8578, Japan

[b] Laboratoire d'Innovation Moléculaire et Applications (LIMA, UMR CNRS 7042)
Université de Strasbourg/Université de Haute Alsace
ECPM, 67087 Strasbourg, France

[c] Research and Analytical Center for Giant Molecules, Graduate School of Science
Tohoku University
Sendai 980-8578, Japan

[d] Endowed Research Laboratory of Dimensional Integrated Nanomaterials, Graduate School of Science
Tohoku University
Sendai 980-8578, Japan

[e] Institute of Organic Chemistry
JMU Würzburg
Am Hubland, Würzburg, Germany

Table of Contents

1. Material and Methods S3

2. Preparation of Starting Materials S4

3. Reaction of Diazomethyl-λ^3-iodanes with Arynes S13

4. C(sp^3)–H Indazolylation of *N*,*N*-Dimethylanilines with Indazolyl-λ^3-iodanes S35

5. Competition Experiments to Evaluate Arynophilicity of Diazomethyl-λ^3-iodane S38

6. X-Ray Crystallographic Analysis S40

7. DFT Calculations S42

8. References S58

9. ^{1}H and ^{13}C NMR Spectra S60

1. Material and Methods

General. All reactions dealing with air- or moisture-sensitive compounds were performed by standard Schlenk techniques in oven-dried reaction vessels under argon. Analytical thin-layer chromatography (TLC) was performed on Merck 60 F254 silica gel plates. Column chromatography was performed using flash chromatography with 40–63 μm silica gel (Silica Gel 60N, Kanto Chemical Co., Inc.). Preparative thin layer chromatography was conducted using a 20 ×20 cm glass sheet coated with a 1mm thick layer of silica gel (FUJIFILM Wako Pure Chemical Corporation, Wakogel® B-5F, Cat. No. 230-00043). ^{1}H, ^{13}C, ^{19}F, and ^{31}P nuclear magnetic resonance (NMR) spectra were recorded on a Varian Mercury (400 MHz) or a JEOL-ECA600 (600 MHz) spectrometer. ^{1}H and ^{13}C NMR spectra were reported in parts per million (ppm) downfield from an internal standard, tetramethylsilane (0.00 ppm for ^{1}H NMR in $CDCl_3$) and $CHCl_3$ (77.16 ppm for ^{13}C NMR in $CDCl_3$), respectively. ^{19}F NMR spectra are referenced to external standard (CF_3CO_2H, –76.6 ppm in $CDCl_3$). ^{31}P NMR spectra are referenced to external standard (PPh_3, –6.0 ppm in $CDCl_3$). The following abbreviations (or combinations thereof) indicate multiplicities: s = singlet, d = doublet, t = triplet, q = quartet, m = multiplet. Melting points were determined with an MPA100 OptiMelt apparatus. High-resolution mass spectra (HRMS) were recorded on a JEOL JMS-DX-303, a JEOL JMS-700, or a JEOL JMS-T100GC spectrometer with a magnetic sector time-of-flight mass analyzer.

Materials. Unless otherwise noted, commercial reagents were purchased from Tokyo Chemical Industry Co., Ltd., Kanto Chemical Co., Inc., Signa-Aldrich Japan, FUJIFILM Wako Pure Chemical Corporation, and other commercial suppliers and were used as received. Anhydrous DMSO, DMF, and THF were purchased from FUJIFILM Wako Pure Chemical Corporation and were used as received.

2. Preparation of Starting Materials

Figure S1 shows the aryne- and iodane-related starting materials used in this study. 3,3-Bis(trifluoromethyl)-1λ^3-benzo[*d*][1,2]iodaoxol-1(3*H*)-yl acetate (acetoxy benziodoxole, **AcOBX**) was prepared according to the literature procedure.[1] Aryne precursors **1a**, **1b**, **1e, 1f**, **1h**, and **1i** were commercially purchased and used without further purification. Substrates **1c**,[2] **1d**,[2] **1g**,[3] **1j**,[4] **1k**,[5] **1l**,[4] **1m**,[6] and **1n**[6] were synthesized according to the reported procedures. Preparation of substrates **2a–2n** is described below.

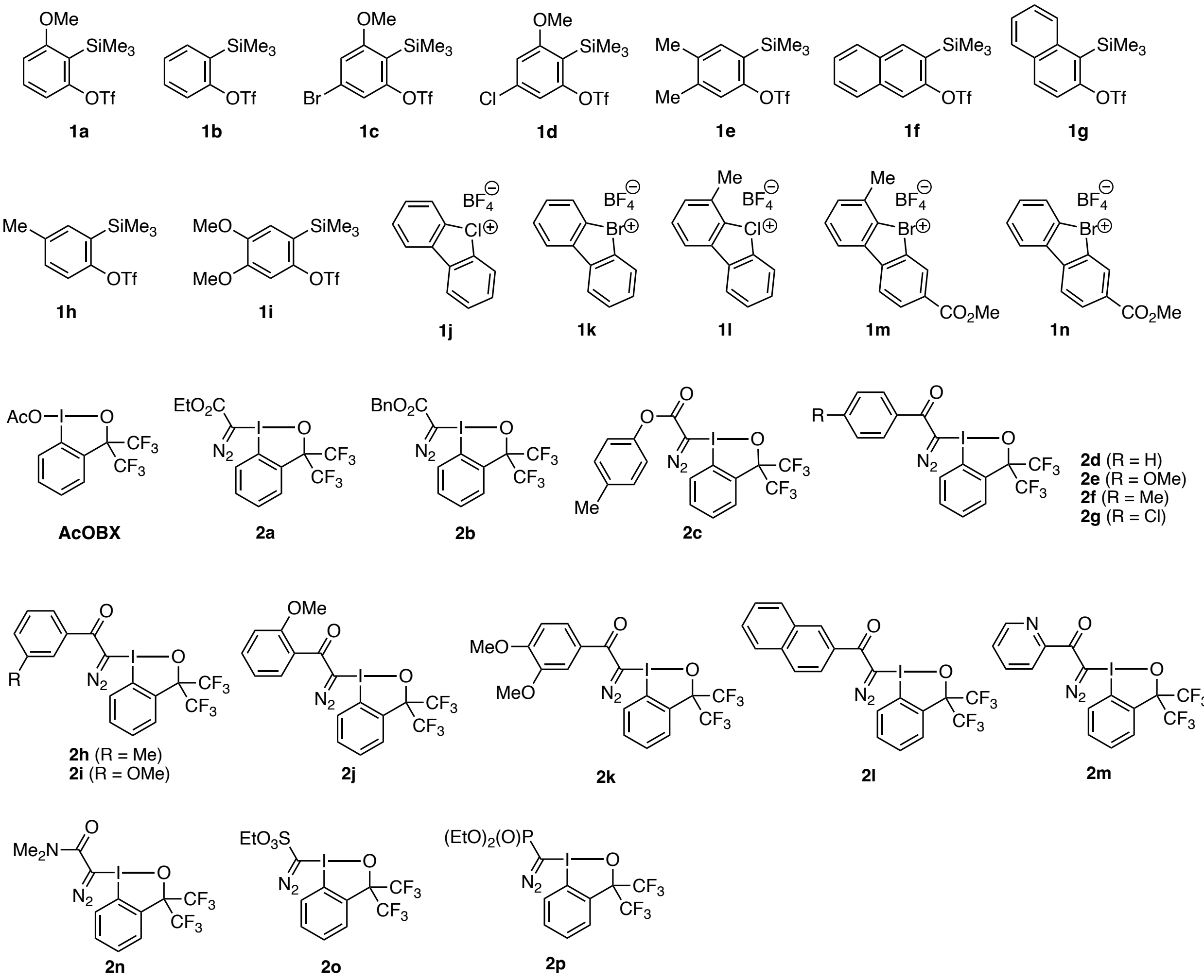

Figure S1. Substrates used in this study.

Synthesis of diazomethyl-λ^3-iodanes (2)

Table S1. Optimization of diazomethylbenziodoxol (**2a**) synthesis

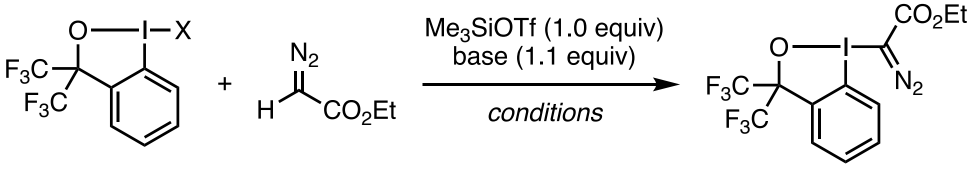

2a

entry	X	Me_3SiOTf	base	conditions	yield
1	OAc	1.0 equiv	pyridine (1.1 equiv)	CH_2Cl_2, rt, 4 h	82%
2	OTf	-	pyridine (1.1 equiv)	CH_2Cl_2, rt, 4 h	trace
3	OTf	1.0 equiv	pyridine (1.1 equiv)	CH_2Cl_2, rt, 4 h	70%
4[a]	OTf	-	pyridine (2.0 equiv)	CH_2Cl_2, 0 °C, 21 h	13%
5[a]	OTf	-	DIPEA (2.0 equiv)	CH_2Cl_2, 0 °C, 21 h	complex
6	Cl	-	pyridine (1.1 equiv)	CH_2Cl_2, rt, 24 h	N.R.
7	OAc	1.0 equiv	pyridine (1.1 equiv)	DCE, 50 °C, 4 h	80%

[a]BXT (1.2 equiv) and diazo compound (1.0 equiv) were used.

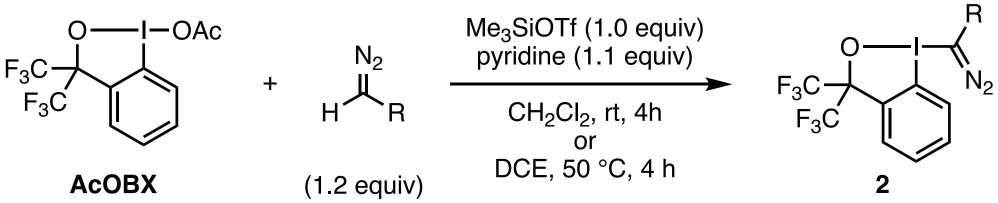

2

General Procedure A: Under an argon atmosphere, to a mixture of AcOBX (0.21 g, 0.49 mmol, 1.0 equiv) suspended in dichloromethane (1.0 mL, 0.5 M) was added trimethylsilyl trifluoromethanesulfonate (90 µL, 0.50 mmol, 1.0 equiv) at room temperature. The resulting mixture was stirred for 10 minutes at this temperature, followed by slow addition of pyridine (45 µL, 0.58 mmol, 1.1 equiv). The resulting mixture was stirred for 1 h, followed by slow addition of a solution of 2-diazo compounds (0.60 mmol, 1.2 equiv) in dichloromethane (0.20 mL). The reaction mixture was stirred for 4 h at room temperature and then was washed with water (10 mL x 2), dried over $MgSO_4$, and evaporated under reduced pressure. The residue was purified by column chromatography (silica gel, 27 g, hexane/ethyl acetate = 10/1 to 3/1) to afford the desired compound **2**.

General Procedure B: Under an argon atmosphere, to a mixture of AcOBX (0.21 g, 0.49 mmol, 1.0 equiv) suspended in 1,2-dichloroethane (1.0 mL, 0.5 M) was added trimethylsilyl trifluoromethanesulfonate (90 µL, 0.50 mmol, 1.0 equiv) at room temperature. The resulting mixture was stirred for 10 minutes at this temperature, followed by slow addition of pyridine (45 µL, 0.58 mmol, 1.1 equiv). The resulting mixture was stirred for 1 h, followed by slow addition of

a solution of 2-diazo compounds (0.60 mmol, 1.2 equiv) in 1,2-dichloroethane (0.20 mL). The reaction mixture was stirred for 4 h at 50 °C and then was washed with water (10 mL x 2), dried over $MgSO_4$, and evaporated under reduced pressure. The residue was purified by column chromatography (silica gel, 27 g, hexane/ethyl acetate = 10/1 to 3/1) to afford the desired compound **2**.

Ethyl 2-(3,3-bis(trifluoromethyl)-1λ³-benzo[*d*][1,2]iodaoxol-1(3*H*)-yl)-2-diazoacetate (2a)

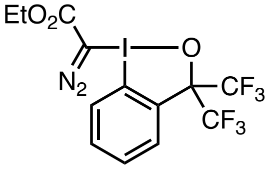

General Procedure A: Yellow solid (200 mg, 85%); m.p. 120-123 °C; R_f 0.26 (hexane/EtOAc = 3/1); **^{1}H NMR** (400 MHz, $CDCl_3$) δ 7.87-7.83 (m, 1H), 7.72-7.68 (m, 2H), 7.57-7.53 (m, 1H), 4.28 (q, J=7.2 Hz, 2H), 1.28 (t, J = 7.2 Hz, 3H); **^{13}C{^{1}H} NMR** (101 MHz, $CDCl_3$) δ 164.5, 132.9, 131.13, 131.08, 130.6, 126.4, 123.5 (q, $J_{C\text{-}F}$ = 289.5 Hz), 111.8, 82.1-81.5 (m), 62.8, 36.9, 14.3; **^{19}F NMR** (376 MHz, $CDCl_3$) δ –76.0; **HRMS** (FAB^+) Calcd for $C_{13}H_{10}F_6IN_2O_3^+$ $[M+H]^+$ 482.9635, found 482.9638.

Benzyl 2-(3,3-bis(trifluoromethyl)-1λ³-benzo[*d*][1,2]iodaoxol-1(3*H*)-yl)-2-diazoacetate (2b)

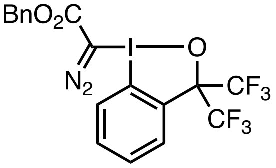

General Procedure A: Yellow solid (342 mg, 63%); m.p. 125-127 °C; R_f 0.20 (hexane/EtOAc = 3/1); **^{1}H NMR** (400 MHz, $CDCl_3$) δ 7.85 (d, J = 7.4 Hz, 1H), 7.69 (app. t, J = 7.3 Hz, 1H), 7.62 (app. t, J = 7.7 Hz, 1H), 7.49 (d, J = 8.2 Hz, 1H), 7.37-7.26 (m, 5H), 5.25 (s, 2H); **^{13}C{^{1}H} NMR** (150 MHz, $CDCl_3$) δ 164.4, 135.1, 133.0, 131.1, 131.0, 130.6, 128.64, 128.60, 128.2, 126.4, 123.5 (q, $J_{C\text{-}F}$ = 289.4 Hz), 111.8, 82.0-81.7 (m), 68.1, 36.8; **^{19}FNMR** (376 MHz, $CDCl_3$) δ –76.1; HRMS (FAB^+) Calcd for $C_{18}H_{12}F_6IN_2O_3^+$ $[M+H]^+$ 544.9791, found 544.9790.

4-Methylphenyl 2-(3,3-bis(trifluoromethyl)-1λ³-benzo[*d*][1,2]iodaoxol-1(3*H*)-yl)-2-diazoacetate (2c)

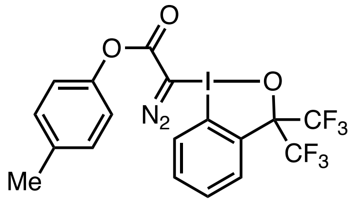

General Procedure B: Yellow solid (441 mg, 54%); m.p. 140-143 °C; R_f 0.46 (hexane/EtOAc = 3/1); **^{1}H NMR** (400 MHz, $CDCl_3$) δ 7.88 (d, J = 6.8 Hz, 1H), 7.79-7.66 (m, 3H), 7.17 (d, J = 8.2

Hz, 2H), 6.99 (d, $J = 8.6$ Hz, 2H), 2.34 (s, 3H); **$^{13}C\{^1H\}$ NMR** (150 MHz, $CDCl_3$) δ 163.3, 148.3, 136.0, 133.1, 131.3, 131.1, 130.8, 130.0, 126.4, 123.5 (q, $J_{C\text{-}F} = 288.7$ Hz), 120.9, 111.9, 36.9, 20.8 (the signal for the carbon bonded to the CF_3 groups, which typically appear as a weak multiplet around 82-81 ppm, was not identified due to the low S/N ratio); **^{19}FNMR** (376 MHz, $CDCl_3$) δ – 76.0; HRMS (FAB^+) Calcd for $C_{18}H_{12}F_6IN_2O_3^+$ $[M+H]^+$ 544.9791, found 544.9806.

2-(3,3-Bis(trifluoromethyl)-1λ^3-benzo[*d*][1,2]iodaoxol-1(3*H*)-yl)-2-diazo-1-phenylethan-1-one (2d)

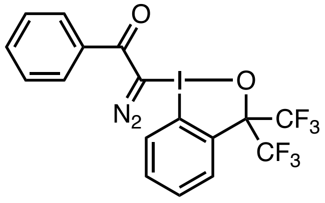

General Procedure A: Yellow solid (112 mg, 43%); m.p. 94-97 °C; R_f 0.41 (hexane/EtOAc = 3/1); **^{1}H NMR** (400 MHz, $CDCl_3$) δ 7.87 (d, $J = 6.0$ Hz, 1H), 7.73-7.68 (m, 4H), 7.61-7.43 (m, 4H); **$^{13}C\{^1H\}$ NMR** (150MHz, $CDCl_3$) δ 186.8, 135.2, 133.1, 132.8, 131.3, 131.2, 130.7, 129.0, 127.5, 126.6, 123.5 (q, $J_{C\text{-}F} = 288.8$ Hz), 111.4, 82.0-81.6 (m), 49.4; **^{19}F NMR** (376 MHz, $CDCl_3$) δ –76.0; **HRMS** (FAB^+) Calcd for $C_{17}H_{10}F_6IN_2O_2^+$ $[M+H]^+$ 514.9686, found 514.9686.

2-(3,3-Bis(trifluoromethyl)-1λ^3-benzo[*d*][1,2]iodaoxol-1(3*H*)-yl)-2-diazo-1-(4-methoxyphenyl)ethan-1-one (2e)

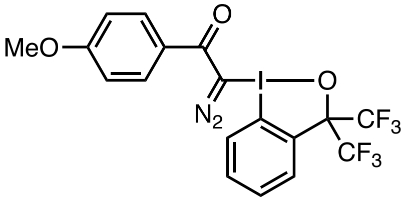

General Procedure A: Yellow solid (247 mg, 46%); m.p. 97-100 °C; R_f 0.48 (hexane/EtOAc = 3/1); ^{1}H NMR (400 MHz, $CDCl_3$) δ 7.87 (d, $J = 6.0$ Hz, 1H), 7.71-7.66 (m, 4H), 7.55 (d, $J = 7.6$ Hz, 1H), 6.97 (d, $J = 8.8$ Hz, 2H), 3.88 (s, 3H); $^{13}C\{^1H\}$ NMR (150 MHz, $CDCl_3$) δ 185.2, 163.3, 133.1, 131.3, 131.2, 130.7, 130.0, 127.7, 126.6, 123.5 (q, $J_{C\text{-}F} = 289.1$ Hz), 114.2, 111.5, 55.6, 48.9 (the signal for the carbon bonded to the CF_3 groups, which typically appear as a weak multiplet around 82-81 ppm, was not identified due to the low S/N ratio); ^{19}F NMR (376 MHz, $CDCl_3$) δ – 75.9; HRMS (FAB^+) Calcd for $C_{18}H_{12}F_6IN_2O_3^+$ $[M+H]^+$ 544.9791, found 544.9810.

2-(3,3-Bis(trifluoromethyl)-1λ^3-benzo[*d*][1,2]iodaoxol-1(3*H*)-yl)-2-diazo-1-(4-methylphenyl)ethan-1-one (2f)

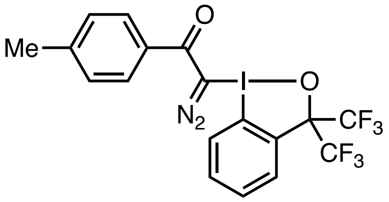

General Procedure B: Yellow solid (184 mg, 35%); m.p. 110-113 °C; R_f 0.44 (hexane/EtOAc = 3/1); **^{1}H NMR** (400 MHz, $CDCl_3$) δ 7.87 (d, J = 7.3 Hz, 1H), 7.73-7.66 (m, 2H), 7.60 (d, J = 8.2 Hz, 2H), 7.57-7.53 (m, 1H), 7.30 (d, J = 8.2 Hz, 2H), 2.44 (s, 3H); **^{13}C{^{1}H} NMR** (150 MHz, $CDCl_3$) δ 186.4, 143.8, 133.1, 132.5, 131.3, 131.2, 130.7, 129.6, 127.7, 126.6, 123.5 (q, $J_{C\text{-}F}$ = 289.4 Hz), 111.5, 49.3, 21.6 (the signal for the carbon bonded to the CF_3 groups, which typically appear as a weak multiplet around 82-81 ppm, was not identified due to the low S/N ratio); **^{19}F NMR** (376 MHz, $CDCl_3$) δ –75.9; **HRMS** (FAB$^+$) Calcd for $C_{18}H_{12}F_6IN_2O_2^+$ $[M+H]^+$ 528.9842, found 528.9835.

2-(3,3-Bis(trifluoromethyl)-1λ^3-benzo[*d*][1,2]iodaoxol-1(3*H*)-yl)-2-diazo-1-(4-chlorophenyl)ethan-1-one (2g)

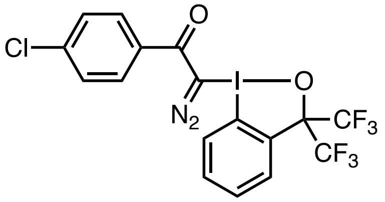

General Procedure B: Yellow solid (202 mg, 37%); m.p. 107-110 °C; R_f 0.44 (hexane/EtOAc = 3/1); **^{1}H NMR** (400 MHz, $CDCl_3$) δ 7.88 (d, J = 6.7 Hz, 1H), 7.75-7.69 (m, 2H), 7.64 (d, J = 8.5 Hz, 2H), 7.55-7.51 (m, 1H), 7.48 (d, J = 8.4 Hz, 2H); **^{13}C{^{1}H} NMR** (150 MHz, $CDCl_3$) δ 185.6, 139.2, 133.5, 133.2, 131.32, 131.30, 130.8, 129.3, 129.0, 126.6, 123.5 (q, $J_{C\text{-}F}$ = 289.4 Hz), 111.5, 82.1-81.5 (m), 49.1; **^{19}F NMR** (376 MHz, $CDCl_3$) δ –76.0; **HRMS** (FAB$^+$) Calcd for $C_{17}H_9{}^{35}ClF_6IN_2O_2^+$ $[M+H]^+$ 548.9296, found 548.9310.

2-(3,3-Bis(trifluoromethyl)-1λ^3-benzo[*d*][1,2]iodaoxol-1(3*H*)-yl)-2-diazo-1-(*m*-tolyl)ethan-1-one (2h)

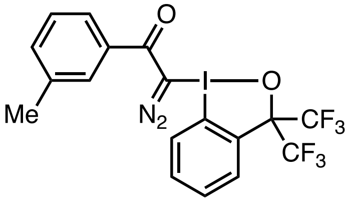

General Procedure B: Yellow solid (145 mg, 27%); m.p. 101-104 °C; R_f 0.49 (hexane/EtOAc = 3/1); **^{1}H NMR** (400 MHz, $CDCl_3$) δ 7.89-7.85 (m, 1H), 7.73-7.67 (m, 2H), 7.58-7.55 (m, 1H), 7.48-7.46 (m, 2H), 7.42-7.35 (m, 2H), 2.39 (s, 3H); **^{13}C{^{1}H} NMR** (150 MHz, $CDCl_3$) δ 187.0, 139.1, 135.2, 133.6, 133.1, 131.3, 131.2, 130.7, 128.8, 128.1, 126.6, 124.6, 123.5 (q, $J_{C\text{-}F}$ = 287.6 Hz), 111.5, 82.0-81.6 (m), 49.4, 21.3; **^{19}F NMR** (376 MHz, $CDCl_3$) δ –76.0; **HRMS** (FAB$^+$) Calcd

for $C_{18}H_{12}F_6IN_2O_2^+$ $[M+H]^+$ 528.9842, found 528.9859.

2-(3,3-Bis(trifluoromethyl)-1λ^3-benzo[*d*][1,2]iodaoxol-1(3*H*)-yl)-2-diazo-1-(3-methoxyphenyl)ethan-1-one (2i)

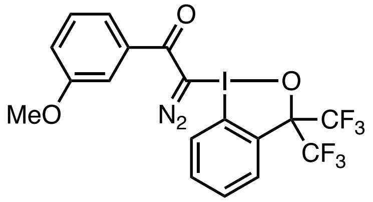

General Procedure B: Yellow solid (248 mg, 46%); m.p. 90-93 °C; R_f 0.29 (hexane/EtOAc = 3/1); **^{1}H NMR** (400 MHz, $CDCl_3$) δ 7.90-7.84 (m, 1H), 7.73-7.67 (m, 2H), 7.58-7.55 (m, 1H), 7.39 (app. t, J = 8.0 Hz, 1H), 7.26-7.23 (m, 1H), 7.19-7.18 (m, 1H), 7.11 (dd, J = 8.2, 2.6 Hz, 1H), 3.81 (s, 3H); **$^{13}C\{^1H\}$ NMR** (150 MHz, $CDCl_3$) δ 186.6, 160.0, 136.4, 133.1, 131.29, 131.25, 130.8, 130.0, 126.5, 123.5 (q, $J_{C\text{-}F}$ = 288.3 Hz), 119.6, 119.0, 112.5, 111.5, 55.4, 49.5 (signal for the carbon bonded to the CF_3 groups, which typically appear as a weak multiplet around 82-81 ppm, was not identified due to the low S/N ratio); **^{19}F NMR** (376 MHz, $CDCl_3$) δ –76.0; **HRMS** (FAB^+) Calcd for $C_{18}H_{12}F_6IN_2O_3^+$ $[M+H]^+$ 544.9791, found 544.9889.

2-(3,3-Bis(trifluoromethyl)-1λ^3-benzo[*d*][1,2]iodaoxol-1(3*H*)-yl)-2-diazo-1-(2-methoxyphenyl)ethan-1-one (2j)

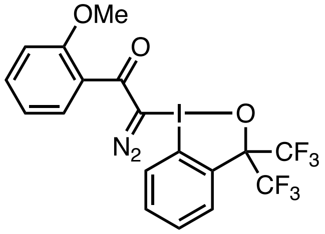

General Procedure A: Yellow solid (223 mg, 51%); m.p. 93-96 °C; R_f 0.24 (hexane/EtOAc = 3/1); **^{1}H NMR** (400 MHz, $CDCl_3$) δ 7.89-7.85 (m, 1H), 7.80-7.76 (m, 1H), 7.72-7.65 (m, 2H), 7.49 (app. dt, J = 7.7, 1.6 Hz, 1H), 7.44 (dd, J = 7.6, 1.6 Hz, 1H), 7.08 (app. t, J = 7.6 Hz, 1H), 7.00 (d, J = 8.4 Hz, 1H), 3.90 (s, 3H); **$^{13}C\{^1H\}$ NMR** (150 MHz, $CDCl_3$) δ 186.6, 155.9, 133.0, 132.7, 131.0, 130.8, 130.4, 129.5, 126.8, 125.4, 123.3 (q, $J_{C\text{-}F}$ = 289.4 Hz), 121.4, 111.5, 111.0, 81.9-81.4 (m), 55.9 (signal for the carbon bonded to the CF_3 groups, which typically appear as a weak multiplet around 82-81 ppm, was not identified due to the low S/N ratio); **^{19}F NMR** (376 MHz, $CDCl_3$) δ –76.0; **HRMS** (FAB^+) Calcd for $C_{18}H_{12}F_6IN_2O_3^+$ $[M+H]^+$ 544.9791, found 544.9788.

2-(3,3-Bis(trifluoromethyl)-1λ^3-benzo[*d*][1,2]iodaoxol-1(3*H*)-yl)-2-diazo-1-(3,4-methoxyphenyl)ethan-1-one (2k)

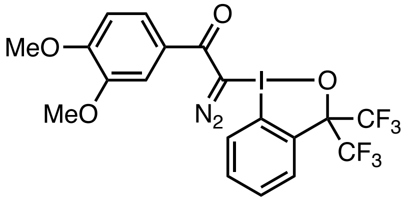

General Procedure A: Yellow solid (186 mg, 32%); m.p. 109-112 °C; R_f 0.12 (hexane/EtOAc = 3/1); **^{1}H NMR** (400 MHz, $CDCl_3$) δ 7.90-7.85 (m, 1H), 7.73-7.68 (m, 2H), 7.61-7.58 (m, 1H), 7.36 (dd, J = 8.4, 2.1 Hz, 1H), 7.24 (d, J = 2.0 Hz, 1H), 6.90 (d, J = 8.4 Hz, 1H), 3.95 (s, 3H), 3.85 (s, 3H); **^{13}C{^{1}H} NMR** (150 MHz, $CDCl_3$) δ 185.0, 152.9, 149.2, 132.9, 131.1, 131.0, 130.5, 127.6, 126.4, 123.3 (q, $J_{C\text{-}F}$ = 289.4 Hz), 121.4, 111.4, 110.6, 110.1, 55.9, 55.7, 48.6 (signal for the carbon bonded to the CF_3 groups, which typically appear as a weak multiplet around 82-81 ppm, was not identified due to the low S/N ratio); **^{19}F NMR** (376 MHz, $CDCl_3$) δ –76.0; **HRMS** (FAB^+) Calcd for $C_{19}H_{14}F_6IN_2O_4^+$ $[M+H]^+$ 574.9897, found 574.9890.

2-(3,3-Bis(trifluoromethyl)-1λ^3-benzo[*d*][1,2]iodaoxol-1(3*H*)-yl)-2-diazo-1-(naphthalen-2-yl)ethan-1-one (2l)

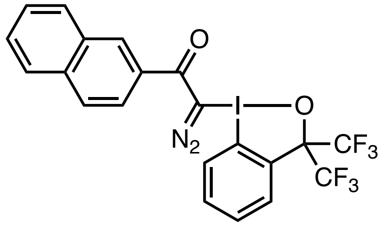

General Procedure A: Yellow solid (223 mg, 40%); m.p. 73-76 °C; R_f 0.20 (*n*-hexane/EtOAc = 3/1); **^{1}H NMR** (400 MHz, $CDCl_3$) δ 8.21 (s, 1H), 7.97-7.87 (m, 4H), 7.76-7.71 (m, 3H), 7.66-7.57 (m, 3H); **^{13}C{^{1}H} NMR** (150 MHz, $CDCl_3$) δ 186.6, 135.2, 133.2, 132.4, 132.3, 131.31, 131.27, 130.8, 129.12, 129.11, 128.64, 128.57, 127.9, 127.3, 126.6, 123.7, 123.6 (q, $J_{C\text{-}F}$ = 290.5 Hz), 111.6, 81.9-81.6 (m), 49.5; **^{19}F NMR** (376 MHz, $CDCl_3$) δ –76.0; **HRMS** (FAB^+) Calcd for $C_{21}H_{12}F_6IN_2O_2^+$ $[M+H]^+$ 564.9842, found 564.9845.

2-(3,3-Bis(trifluoromethyl)-1λ^3-benzo[*d*][1,2]iodaoxol-1(3*H*)-yl)-2-diazo-1-(pyridin-2-yl)ethan-1-one (2m)

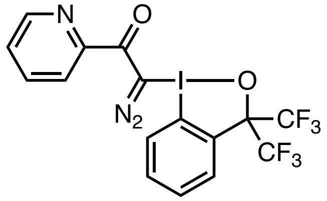

General Procedure A: Yellow solid (340 mg, 75%); m.p. 107.1-116.2 °C; R_f 0.37 (hexane/EtOAc = 3/1); **^{1}H NMR** (400 MHz, $CDCl_3$) δ 8.63-8.56 (m, 1H), 8.04 (d, J = 7.9, 1H), 7.91 (app. dt, J = 8.6, 1.6 Hz, 1H), 7.86 (d, J = 7.8 Hz, 1H), 7.69-7.59 (m, 3H), 7.54-7.51 (m, 1H); **^{13}C{^{1}H} NMR** (150 MHz, $CDCl_3$) δ 182.9, 151.9, 148.0, 137.5, 132.9, 131.2, 130.9, 130.4, 127.5, 126.8, 123.6

(q, $J_{C\text{-}F}$ = 289.1 Hz), 122.8, 111.5, 82.1-81.7 (m) (the signal for the carbon which is attached the diazo nitrogen was not observed); **^{19}F NMR** (376 MHz, $CDCl_3$) δ – 76.0; **HRMS** (FAB^+) Calcd for $C_{16}H_9F_6IN_3O_2^+$ $[M+H]^+$ 515.9638, found 515.9646.

2-(3,3-Bis(trifluoromethyl)-1λ^3-benzo[*d*][1,2]iodaoxol-1(3*H*)-yl)-2-diazo-*N*,*N*-dimethylacetamide (2n)

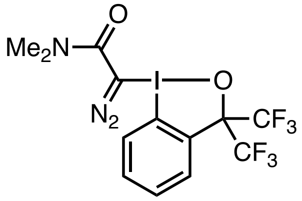

General Procedure B: Yellow solid (45.7 mg, 19%); m.p. 108-111 °C; R_f 0.13 (hexane/EtOAc = 1/1); **^{1}H NMR** (400 MHz, $CDCl_3$) δ 7.87-7.83 (m, 1H), 7.72-7.62 (m, 3H), 3.08 (s, 6H); **^{13}C{^{1}H} NMR** (150 MHz, $CDCl_3$) δ 163.5, 133.0, 131.3, 131.0, 130.6, 127.0, 123.5 (q, $J_{C\text{-}F}$ = 289.4 Hz), 111.9, 38.5, 38.2 (the signals for the carbons attached to the diazo nitrogen and CF_3 groups were not observed); **^{19}F NMR** (376 MHz, $CDCl_3$) δ –76.1; **HRMS** (FAB^+) Calcd for $C_{13}H_{11}F_6IN_3O_2^+$ $[M+H]^+$ 481.9795, found 481.9784.

Ethyl (3,3-bis(trifluoromethyl)-1λ^3-benzo[*d*][1,2]iodaoxol-1(3*H*)-yl)(diazo)methanesulfonate (2o)

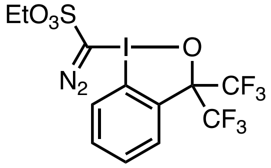

General Procedure B: Yellow solid (205 mg, 42%); m.p. 105-108 °C; R_f 0.39 (hexane/EtOAc = 3/1); **^{1}H NMR** (400 MHz, $CDCl_3$) δ 7.86 (d, *J* =7.5 Hz, 1H), 7.84-7.73 (m, 3H), 4.36 (q, *J* =7.2 Hz, 2H), 1.43 (t, *J* = 7.2 Hz, 3H); **^{13}C{^{1}H} NMR** (150 MHz, $CDCl_3$) δ 133.5, 131.6, 131.1, 130.6, 127.4, 123.3 (q, $J_{C\text{-}F}$ = 288.7 Hz), 112.2, 82.4-82.0 (m), 68.3, 40.4, 14.8; **^{19}F NMR** (376 MHz, $CDCl_3$) δ –76.0; **HRMS** (FAB^+) Calcd for $C_{12}H_{10}F_6IN_2O_4S^+$ $[M+H]^+$ 518.9305, found 518.9307.

Diethyl ((3,3-bis(trifluoromethyl)-1λ^3-benzo[*d*][1,2]iodaoxol-1(3*H*)-yl)(diazo)methyl)phosphonate (2p)

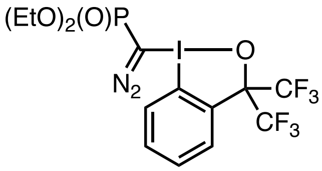

General Procedure B: Yellow solid (116.1 mg, 40%); m.p. 89-92 °C; R_f 0.19 (hexane/EtOAc = 1/1); **^{1}H NMR** (400 MHz, $CDCl_3$) δ 7.88-7.81 (m, 2H), 7.78-7.68 (m, 2H), 4.23-4.13 (m, 4H), 1.31 (t, *J* =7.2 Hz, 6H); **^{13}C{^{1}H} NMR** (150 MHz, $CDCl_3$) δ 132.7, 131.3, 131.1, 130.4, 127.2, 123.4 (q, $J_{C\text{-}F}$ = 289.0 Hz), 111.4, 82.0-81.6 (m), 63.69, 63.65, 22.3 (d, $J_{C\text{-}P}$ = 207.9 Hz), 16.1, 16.0;

^{19}F NMR (376 MHz, $CDCl_3$) δ –76.1; **^{31}P NMR** (243 MHz, $CDCl_3$) δ 15.0; **HRMS** (FAB^+) Calcd for $C_{14}H_{15}F_6IN_2O_4P^+$ $[M+H]^+$ 546.9713, found 546.9735.

3. Reaction of Diazomethyl-λ^3-iodanes with Arynes

Table S2. Optimization of indazolyl-λ^3-iodane synthesis from **1a** and **2a**

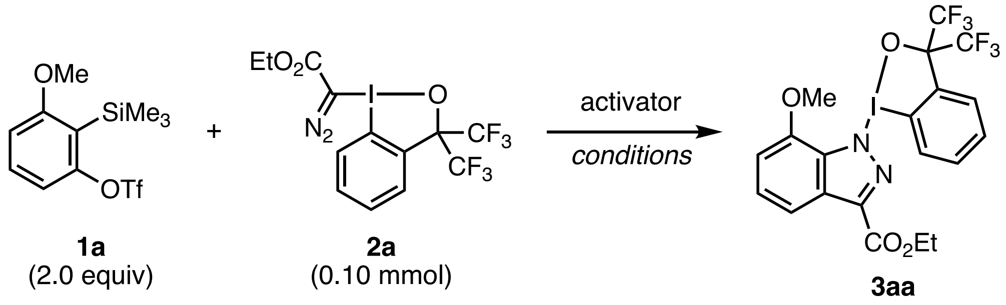

entry	activator	solvent	conditions	yield
1	CsF (4.0 equiv)	MeCN	rt, 18 h	85%
2	CsF (4.0 equiv)	DME	rt, 18 h	70%[a]
3	CsF (4.0 equiv)	THF	rt, 18 h	65%[a]
4	CsF (4.0 equiv)	Toluene	rt, 18 h	N.R.
5	TBAF (4.0 equiv)	THF	rt, 18 h	complex
6	TBAT (4.0 equiv)	THF	rt, 18 h	complex
7	KF/18C6 (4.0 equiv)	THF	rt, 18 h	80%[a]
8	CsF (4.0 equiv)	MeCN	10 °C, 18 h	75%[a]
9	CsF (4.0 equiv)	MeCN	rt, 6 h	61%[a]
10[b]	CsF (2.0 equiv)	MeCN	rt, 18 h	83%[a]
11[c]	CsF (2.0 equiv)	MeCN	rt, 18 h	91%[a]

[a]Yields were determined by ^{1}NMR analysis using 1,1,2,2-tetrachloroethane as an internal standard. [b]**1a** (1.0 equiv) and **2a** (2.0 equiv) were used. [c]**1a** (1.0 equiv) and **2a** (3.0 equiv) were used.

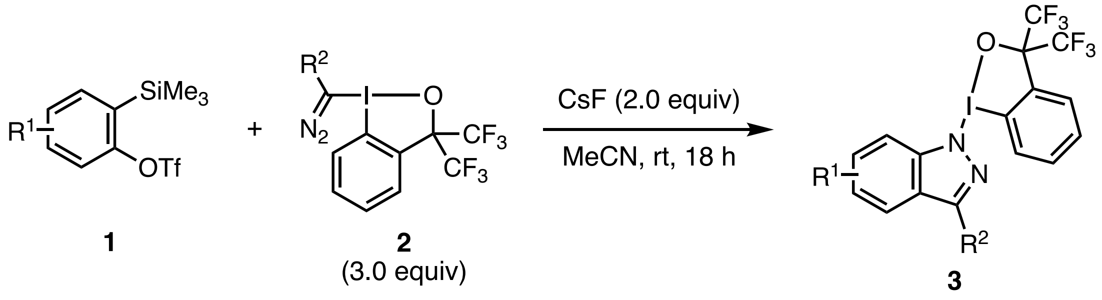

General Procedure C: In a 4 mL vial equipped with a magnetic stir bar, diazomethylbenziodoxole **2** (0.30 mmol, 3.0 equiv) was dissolved in acetonitrile (1.0 mL). To this solution were added *o*-silylaryl triflate **1** (0.10 mmol, 1.0 equiv) and cesium fluoride (30.4 mg, 0.20 mmol, 2.0 equiv) at room temperature. The resulting mixture was stirred at the same temperature for 18 h under an argon atmosphere and then added ethyl acetate and filtered. The filtrate was concentrated under

reduced pressure, and the residue was purified by column chromatography (silica gel, 18 g, hexane/ethyl acetate = 10/1 to 5/1) to afford the desired product **3**.

Ethyl 1-(3,3-bis(trifluoromethyl)-1λ^3-benzo[*d*][1,2]iodaoxol-1(3*H*)-yl)-7-methoxy-1*H*-indazole-3-carboxylate (3aa)

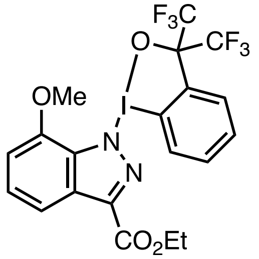

Colorless solid (50.0 mg, 85%); m.p. 188-191 °C; R_f 0.51 (hexane/EtOAc = 3/1); **^{1}H NMR** (400 MHz, $CDCl_3$) δ 7.86 (d, *J* = 8.2 Hz, 1H), 7.77 (d, *J* = 7.8 Hz, 1H), 7.60 (app. dt, *J* = 7.7, 0.9 Hz, 1H), 7.44 (ddd, *J* = 8.5, 7.2, 1.3 Hz, 1H), 7.30-7.24 (m, 1H), 6.77 (d, *J* = 7.6 Hz, 1H), 6.36 (d, *J* = 8.4 Hz, 1H), 4.54 (q, *J* = 7.2 Hz, 2H), 3.79 (s, 3H), 1.49 (t, *J* = 7.1 Hz, 3H); **^{13}C{^{1}H} NMR** (150 MHz, $CDCl_3$) δ 162.4, 146.3, 141.1, 137.8, 133.4, 131.0, 130.7, 129.8, 127.5, 125.3, 124.6, 123.2 (q, $J_{C\text{-}F}$ = 286.2 Hz), 117.7, 113.9, 106.1, 61.3, 55.3, 14.5 (the signal for the carbon bonded to the CF_3 groups, which typically appear as a weak multiplet around 82-81 ppm, was not identified due to the low S/N ratio); **^{19}F NMR** (376 MHz, $CDCl_3$) δ –75.9; **HRMS** (FAB^+) Calcd for $C_{20}H_{15}F_6IN_2O_4^+$ $[M]^+$ 587.9975, found 587.9981.

Benzyl 1-(3,3-bis(trifluoromethyl)-1λ^3-benzo[*d*][1,2]iodaoxol-1(3*H*)-yl)-7-methoxy-1*H*-indazole-3-carboxylate (3ab)

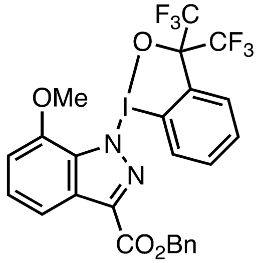

Colorless solid (63%; The yield was determined by ^{1}H NMR analysis using 1,1,2,2-tetrachloroethane as an internal standard because separation from the starting material was difficult; A small amount of analytically pure sample was obtained by purification on PTLC); m.p. 162-165 °C; R_f 0.41 (hexane/EtOAc = 3/1); **^{1}H NMR** (400 MHz, $CDCl_3$) δ 7.82-7.76 (m, 2H), 7.60 (app. t, *J* = 7.6 Hz, 1H), 7.54-7.52 (m, 2H), 7.45-7.34 (m, 4H), 7.24 (m, 1H), 6.75 (d, *J* = 7.7 Hz, 1H), 6.37 (d, *J* = 8.4 Hz, 1H), 5.52 (s, 2H), 3.78 (s, 3H); **^{13}C{^{1}H} NMR** (150 MHz, $CDCl_3$) δ 162.2, 146.3, 140.8, 137.8, 135.9, 133.4, 131.1, 130.7, 129.8, 128.62, 128.60, 128.4, 127.5, 125.3, 124.7, 123.1 (q, $J_{C\text{-}F}$ = 286.6 Hz), 117.6, 113.8, 106.1, 66.9, 55.3 (the signal for the carbon bonded to the CF_3 groups, which typically appear as a weak multiplet around 82-81 ppm, was not identified due to the low S/N ratio); **^{19}FNMR** (376 MHz, $CDCl_3$) δ –76.0 ; **HRMS** (FAB^+) Calcd for

$C_{25}H_{17}F_6IN_2O_4^+$ [M]$^+$ 650.0132, found 650.0143.

***p*-Tolyl 1-(3,3-bis(trifluoromethyl)-1λ^3-benzo[*d*][1,2]iodaoxol-1(3*H*)-yl)-7-methoxy-1*H*-indazole-3-carboxylate (3ac)**

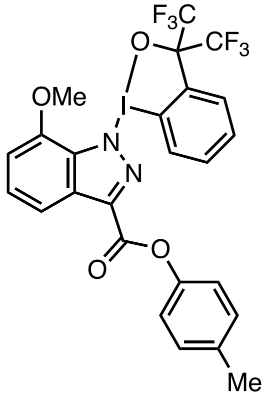

Colorless solid (28.5 mg, 43%); m.p. 193-196 °C; R_f 0.54 (hexane/EtOAc = 3/1); **^{1}H NMR** (400 MHz, $CDCl_3$) δ 7.90 (d, J = 8.2 Hz, 1H), 7.79 (d, J = 7.8 Hz, 1H), 7.62 (app. t, J = 7.8 Hz, 1H), 7.48 (app. t, J = 7.9 Hz, 1H), 7.29 (app. t, J = 7.9 Hz, 1H), 7.24 (d, J = 8.4 Hz, 2H), 7.18 (d, J = 8.6 Hz, 2H), 6.80 (d, J = 7.6 Hz, 1H), 6.44 (d, J = 8.4 Hz, 1H), 3.81 (s, 3H), 2.38 (s, 3H); **^{13}C{^{1}H} NMR** (150 MHz, $CDCl_3$) δ 161.0, 148.3, 146.4, 140.2, 137.9, 135.7, 133.5, 131.1, 130.8, 130.0, 129.8, 127.5, 125.7, 125.0, 123.1 (q, $J_{C\text{-}F}$ = 288.3 Hz), 121.6, 117.6, 113.8, 106.3, 55.3, 20.9 (the signal for the carbon bonded to the CF_3 groups, which typically appear as a weak multiplet around 82-81 ppm, was not identified due to the low S/N ratio); **^{19}F NMR** (376 MHz, $CDCl_3$) δ –75.9; **HRMS** (FAB$^+$) Calcd for $C_{25}H_{17}F_6IN_2O_4^+$ [M]$^+$ 650.0132, found 650.0139.

(1-(3,3-Bis(trifluoromethyl)-1λ^3-benzo[*d*][1,2]iodaoxol-1(3*H*)-yl)-7-methoxy-1*H*-indazol-3-yl)(phenyl)methanone (3ad)

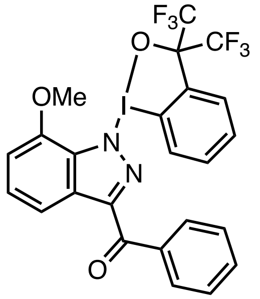

Yellow solid (36.5 mg, 59%); m.p. 170-173 °C; R_f 0.70 (hexane/EtOAc = 2/1); **^{1}H NMR** (400 MHz, $CDCl_3$) δ 8.35 (d, J = 7.4 Hz, 2H), 8.07 (d, J = 8.2 Hz, 1H), 7.78 (d, J = 7.7 Hz, 1H), 7.62-7.58 (m, 2H), 7.51 (app. t, J = 8.0 Hz, 2H), 7.45 (app. t, J = 8.4 Hz, 1H), 7.31 (app. t, J = 8.2 Hz, 1H), 6.79 (d, J = 7.7 Hz, 1H), 6.40 (d, J = 8.4 Hz, 1H), 3.80 (s, 3H); **^{13}C{^{1}H} NMR** (150 MHz, $CDCl_3$) δ 188.3, 147.6, 146.2, 137.6, 137.4, 133.5, 132.7, 131.1, 130.8, 130.7, 129.8, 128.2, 127.4, 126.1, 125.1, 123.2 (q, $J_{C\text{-}F}$ = 288.0 Hz), 117.7, 114.7, 106.5, 84.6-84.2 (m), 55.3; **^{19}FNMR** (376 MHz, $CDCl_3$) δ –75.9; **HRMS** (FAB$^+$) Calcd for $C_{24}H_{16}F_6IN_2O_3^+$ [M+H]$^+$ 621.0104, found

621.0110.

(1-(3,3-Bis(trifluoromethyl)-1λ^3-benzo[*d*][1,2]iodaoxol-1(3*H*)-yl)-7-methoxy-1*H*-indazol-3-yl)(4-methoxyphenyl)methanone (3ae)

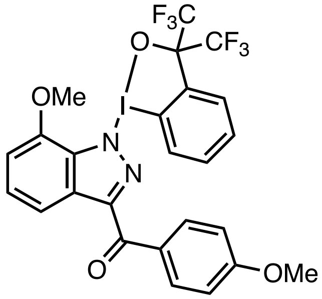

Colorless solid (44.7 mg, 69%); m.p. 164-167 °C; R_f 0.51 (hexane/EtOAc = 3/1); **^{1}H NMR** (400 MHz, $CDCl_3$) δ 8.42 (d, *J* = 9.0 Hz, 2H), 8.05 (d, *J* = 8.2 Hz, 1H), 7.78 (d, *J* = 7.6 Hz, 1H), 7.59 (app. t, *J* = 7.7 Hz, 1H), 7.44 (app. t, *J* = 7.8 Hz, 1H), 7.28 (app. t, *J* = 8.0 Hz, 1H), 6.99 (d, *J* = 8.9 Hz, 2H), 6.78 (d, *J* = 7.6 Hz, 1H), 6.38 (d, *J* = 8.4 Hz, 1H), 3.88 (s, 3H), 3.80 (s, 3H); **^{13}C{^{1}H} NMR** (150 MHz, $CDCl_3$) δ 186.7, 163.5, 147.9, 146.2, 137.3, 133.4, 133.0, 131.0, 130.8, 130.4, 129.8, 127.4, 126.2, 124.2, 123.2 (q, $J_{C\text{-}F}$ = 289.8 Hz), 117.7, 114.8, 113.6, 106.4, 84.6-84.2 (m), 55.4, 55.2; **^{19}FNMR** (376 MHz, $CDCl_3$) δ –75.9; **HRMS** (FAB$^+$) Calcd for $C_{25}H_{17}F_6IN_2O_4^+$ $[M]^+$ 650.0132, found 650.0132.

(1-(3,3-Bis(trifluoromethyl)-1λ^3-benzo[*d*][1,2]iodaoxol-1(3*H*)-yl)-7-methoxy-1*H*-indazol-3-yl)(*p*-tolyl)methanone (3af)

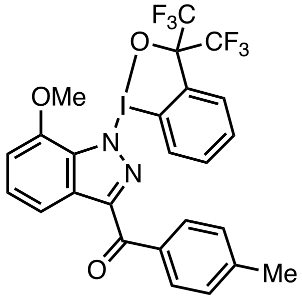

Colorless solid (26.3 mg, 42%); m.p. 150-153 °C; R_f 0.43 (hexane/EtOAc = 3/1); **^{1}H NMR** (400 MHz, $CDCl_3$) δ 8.26 (d, *J* = 8.2 Hz, 2H), 8.05 (d, *J* = 8.2 Hz, 1H), 7.78 (d, *J* = 7.7 Hz, 1H), 7.60 (app. t, *J* = 7.6 Hz, 1H), 7.44 (app. dt, *J* = 7.8, 1.3 Hz, 1H), 7.32-7.28 (m, 3H), 6.79 (d, *J* = 7.7 Hz, 1H), 6.38 (d, *J* = 8.4 Hz, 1H), 3.80 (s, 3H), 2.43 (s, 3H); **^{13}C{^{1}H} NMR** (150 MHz, $CDCl_3$) δ 188.0, 147.8, 146.2, 143.6, 137.3, 135.0, 133.4, 131.0, 130.8, 129.8, 129.0, 127.4, 126.1, 125.0, 123.2 (q, $J_{C\text{-}F}$ = 287.6 Hz), 117.7, 114.8, 106.4, 55.3, 21.7 (the signal for the carbon bonded to the CF_3 groups, which typically appear as a weak multiplet around 82-81 ppm, was not identified due to the low S/N ratio); **^{19}FNMR** (376 MHz, $CDCl_3$) δ –75.9; **HRMS** (FAB$^+$) Calcd for $C_{25}H_{17}F_6IN_2O_3^+$ $[M]^+$ 634.0183, found 634.0180.

(1-(3,3-Bis(trifluoromethyl)-1λ^3-benzo[*d*][1,2]iodaoxol-1(3*H*)-yl)-7-methoxy-1*H*-indazol-3-yl)(4-chlorophenyl)methanone (3ag)

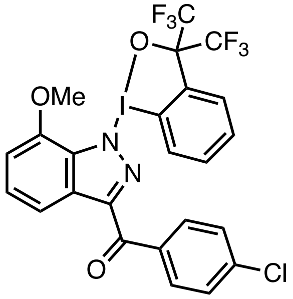

Colorless solid (19.0 mg, 30%); m.p. 154-157 °C; R_f 0.54 (hexane/EtOAc = 3/1); **^{1}H NMR** (400 MHz, $CDCl_3$) δ 8.34 (d, *J* = 8.6 Hz, 2H), 8.06 (d, *J* = 8.2 Hz, 1H), 7.79 (d, *J* = 7.2 Hz, 1H), 7.61 (app. t, *J* = 7.7 Hz, 1H), 7.49-7.30 (m, 3H), 7.32 (app. t, *J* = 8.0 Hz, 1H), 6.80 (d, *J* = 7.6 Hz, 1H), 6.38 (d, *J* = 8.4 Hz, 1H), 3.81 (s, 3H); **^{13}C{^{1}H} NMR** (150 MHz, $CDCl_3$) δ 186.9, 147.4, 146.2, 139.2, 137.4, 135.9, 133.5, 132.1, 131.1, 130.8, 129.9, 128.6, 127.4, 126.1, 125.3, 123.2 (q, $J_{C\text{-}F}$ = 288.3 Hz), 117.6, 114.7, 106.6, 55.3 (the signal for the carbon bonded to the CF_3 groups, which typically appear as a weak multiplet around 82-81 ppm, was not identified due to the low S/N ratio); **^{19}FNMR** (376 MHz, $CDCl_3$) δ –75.9; **HRMS** (FAB^+) Calcd for $C_{24}H_{14}{}^{35}ClF_6IN_2O_3{}^+$ $[M]^+$ 653.9636, found 653.9638.

(1-(3,3-Bis(trifluoromethyl)-1λ^3-benzo[*d*][1,2]iodaoxol-1(3*H*)-yl)-7-methoxy-1*H*-indazol-3-yl)(*m*-tolyl)methanone (3ah)

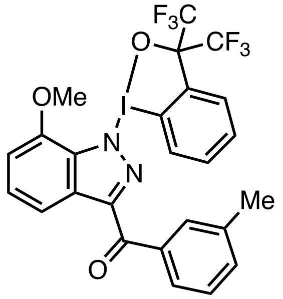

Yellow solid (21.3 mg, 34%); m.p. 125-128 °C; R_f 0.45 (hexane/EtOAc = 3/1); **^{1}H NMR** (400 MHz, $CDCl_3$) δ 8.15 (d, *J* = 6.1 Hz, 1H), 8.08 (s, 1H), 8.05 (d, *J* = 8.2 Hz, 1H), 7.78 (d, *J* = 7.5 Hz, 1H), 7.60 (app. t, *J* = 7.6 Hz, 1H), 7.46-7.37 (m, 3H), 7.31 (app. t, *J* = 8.0 Hz, 1H), 6.80 (d, *J* = 7.6 Hz, 1H), 6.38 (d, *J* = 8.4 Hz, 1H), 3.81 (s, 3H), 2.43 (s, 3H); **^{13}C{^{1}H} NMR** (150 MHz, $CDCl_3$) δ 188.7, 147.8, 146.2, 138.0, 137.7, 137.4, 133.6, 133.4, 131.1, 130.9, 130.8, 129.8, 128.11, 128.08, 127.5, 126.1, 125.1, 123.2 (q, $J_{C\text{-}F}$ = 287.6 Hz), 117.7, 114.7, 106.5, 55.3, 21.4 (the signal for the carbon bonded to the CF_3 groups, which typically appear as a weak multiplet around 82-81 ppm, was not identified due to the low S/N ratio); **^{19}FNMR** (376 MHz, $CDCl_3$) δ –75.9; **HRMS** (FAB^+) Calcd for $C_{25}H_{17}F_6IN_2O_3{}^+$ $[M]^+$ 634.0183, found 634.0187.

(1-(3,3-Bis(trifluoromethyl)-1λ^3-benzo[*d*][1,2]iodaoxol-1(3*H*)-yl)-7-methoxy-1*H*-indazol-3-

yl)(2-methoxyphenyl)methanone (3aj)

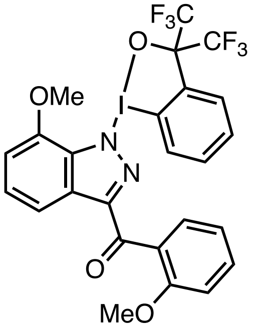

Yellow solid (24.2 mg, 37%); m.p. 117-120 °C; R_f 0.54 (hexane/EtOAc = 3/1); **^{1}H NMR** (600 MHz, $CDCl_3$) δ 7.98 (dd, J = 8.1, 2.5 Hz, 1H), 7.76 (dd, J = 7.3, 0.3 Hz, 1H), 7.61-7.58 (m, 2H), 7.49-7.43 (m, 2H), 7.30-7.25 (m, 1H), 7.05-7.00 (m, 2H), 6.77 (d, J = 7.6 Hz, 1H), 6.38 (d, J = 8.4 Hz, 1H), 3.78 (s, 3H), 3.75 (s, 3H); **^{13}C{^{1}H} NMR** (150 MHz, $CDCl_3$) δ 190.3, 158.0, 148.3, 146.1, 137.6, 133.3, 132.3, 131.0, 130.8, 130.5, 129.7, 129.0, 127.4, 125.3, 125.0, 123.2 (q, $J_{C\text{-}F}$ = 287.3 Hz), 120.2, 117.7, 114.6, 111.8, 106.3, 55.7, 55.2 (the signal for the carbon bonded to the CF_3 groups, which typically appear as a weak multiplet around 82-81 ppm, was not identified due to the low S/N ratio); **^{19}FNMR** (376 MHz, $CDCl_3$) δ –75.9; **HRMS** (FAB$^+$) Calcd for $C_{25}H_{17}F_6IN_2O_4^+$ [M]$^+$ 650.0132, found 650.0158.

(1-(3,3-Bis(trifluoromethyl)-1λ^3-benzo[*d*][1,2]iodaoxol-1(3*H*)-yl)-7-methoxy-1*H*-indazol-3-yl)(3,4-dimethoxyphenyl)methanone (3ak)

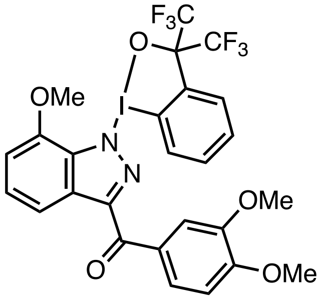

Pale yellow solid (21.5 mg, 32%); m.p. 137-140 °C; R_f 0.34 (hexane/EtOAc = 3/1); **^{1}H NMR** (400 MHz, $CDCl_3$) δ 8.22 (dd, J = 8.5, 2.0 Hz, 1H), 8.05 (d, J = 8.2 Hz, 1H), 7.93 (d, J = 2.0 Hz, 1H), 7.79 (d, J = 7.6 Hz, 1H), 7.60 (app. t, J = 7.2 Hz, 1H), 7.45 (app. dt, J = 7.7, 1.4 Hz, 1H), 7.31 (app. t, J = 8.1 Hz, 1H), 6.97 (d, J = 8.6 Hz, 1H), 6.80 (d, J = 7.6 Hz, 1H), 6.37 (d, J = 8.4 Hz, 1H), 3.96 (s, 3H), 3.92 (s, 3H), 3.83 (s, 3H); **^{13}C{^{1}H} NMR** (150 MHz, $CDCl_3$) δ 186.5, 153.3, 148.8, 148.0, 146.2, 137.3, 133.5, 131.1, 130.9, 130.3, 129.9, 127.4, 126.2, 126.1, 125.0, 123.2 (q, $J_{C\text{-}F}$ = 289.4 Hz), 117.7, 114.8, 112.6, 110.1, 106.4, 56.1, 55.9, 55.3 (the signal for the carbon bonded to the CF_3 groups, which typically appear as a weak multiplet around 82-81 ppm, was not identified due to the low S/N ratio); **^{19}FNMR** (376 MHz, $CDCl_3$) δ –75.9; **HRMS** (FAB$^+$) Calcd for $C_{26}H_{19}F_6IN_2O_5^+$ [M]$^+$ 680.0237, found 680.0233.

Ethyl 1-(3,3-bis(trifluoromethyl)-1λ^3-benzo[*d*][1,2]iodaoxol-1(3*H*)-yl)-1*H*-indazole-3-carboxylate (3ba)

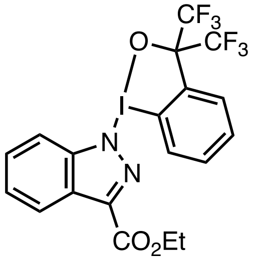

Colorless solid (35.0 mg, 62%); m.p. 169-172 °C; R_f 0.71 (hexane/EtOAc = 2/1); **^{1}H NMR** (400 MHz, $CDCl_3$) δ 8.29 (d, J = 7.8 Hz, 1H), 7.81 (d, J = 7.5 Hz, 1H), 7.63 (app. t, J = 7.3 Hz, 1H), 7.50-7.36 (m, 4H), 6.27 (d, J = 8.5 Hz, 1H), 4.56 (q, J = 7.1 Hz, 2H), 1.50 (t, J = 7.1 Hz, 3H); **^{13}C{^{1}H} NMR** (101 MHz, $CDCl_3$) δ 162.3, 147.6, 140.8, 133.8, 131.5, 131.1, 130.3, 127.9, 127.3, 123.9, 123.4, 123.1(q, $J_{C\text{-}F}$ = 288.5 Hz), 122.4, 115.8, 111.6, 61.4, 14.5 (the signal for the carbon bonded to the CF_3 groups, which typically appear as a weak multiplet around 82-81 ppm, was not identified due to the low S/N ratio); **^{19}F NMR** (376 MHz, $CDCl_3$) δ –75.8; **HRMS** (FAB$^+$) Calcd for $C_{19}H_{13}F_6IN_2O_3^+$ [M]$^+$ 557.9870, found 557.9865.

Ethyl 1-(3,3-bis(trifluoromethyl)-1λ^3-benzo[*d*][1,2]iodaoxol-1(3*H*)-yl)-5-bromo-7-methoxy-1*H*-indazole-3-carboxylate (3ca)

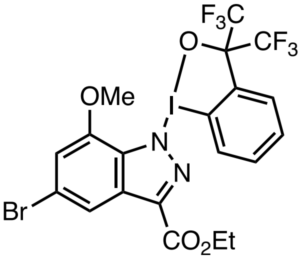

Colorless solid (29.6 mg, 45%); m.p. 152-155 °C; R_f 0.55 (hexane/EtOAc = 3/1); **^{1}H NMR** (400 MHz, $CDCl_3$) δ 8.03 (d, J = 1.4 Hz, 1H), 7.78 (d, J = 7.4 Hz, 1H), 7.62 (app. t, J = 7.5 Hz, 1H), 7.46 (app. dt, J = 7.8, 1.4 Hz, 1H), 6.85 (d, J = 1.4 Hz, 1H), 6.30 (d, J = 8.4 Hz, 1H), 4.54 (q, J = 7.2 Hz, 2H), 3.79 (s, 3H), 1.49 (t, J = 7.2 Hz, 3H); **^{13}C{^{1}H} NMR** (150 MHz, $CDCl_3$) δ 161.9, 146.4, 140.3, 136.7, 133.5, 131.2, 131.0, 130.8, 129.9, 127.4, 126.0, 123.1 (q, $J_{C\text{-}F}$ = 289.1 Hz), 117.3, 116.5, 110.1, 61.5, 55.7, 14.5 (two signals of CF_3 groups, which typically appear as a quartet around 123 ppm was not identified due to the low S/N ratio and the signal for the carbon bonded to the CF_3 groups, which typically appear as a weak multiplet around 82-81 ppm, was not identified due to the low S/N ratio); **^{19}F NMR** (376 MHz, $CDCl_3$) δ –75.9; **HRMS** (FAB$^+$) Calcd for $C_{20}H_{14}{}^{79}BrF_6IN_2O_4^+$ [M]$^+$ 665.9080, found 665.9084.

Ethyl 1-(3,3-bis(trifluoromethyl)-1λ^3-benzo[*d*][1,2]iodaoxol-1(3*H*)-yl)-5-chloro-7-methoxy-1*H*-indazole-3-carboxylate (3da)

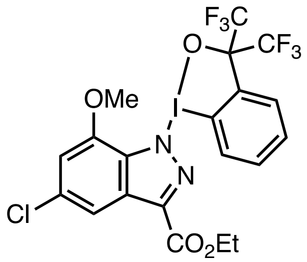

Colorless solid (28.0 mg, 44%); m.p. 172-175 °C; R_f 0.55 (hexane/EtOAc = 3/1); **^{1}H NMR** (600 MHz, $CDCl_3$) δ 7.84 (d, J = 1.6 Hz, 1H), 7.78 (d, J = 7.3 Hz, 1H), 7.61 (app. t, J = 7.5 Hz, 1H), 7.46 (app. dt, J = 7.9, 1.3 Hz, 1H), 6.73 (d, J = 1.4 Hz, 1H), 6.31 (d, J = 8.5 Hz, 1H), 4.53 (q, J = 7.2 Hz, 2H), 3.79 (s, 3H), 1.49 (t, J = 7.2 Hz, 3H); **^{13}C{^{1}H} NMR** (150 MHz, $CDCl_3$) δ 162.0, 146.3, 140.5, 136.5, 133.5, 131.2, 131.0, 130.8, 129.9 127.4, 125.4, 123.1 (q, $J_{C\text{-}F}$ = 289.1 Hz), 117.5, 113.3, 107.7, 61.5, 55.7, 14.4 (two signals of CF_3 groups, which typically appear as a quartet around 123 ppm was not identified due to the low S/N ratio and the signal for the carbon bonded to the CF_3 groups, which typically appear as a weak multiplet around 82-81 ppm, was not identified due to the low S/N ratio); **^{19}F NMR** (376 MHz, $CDCl_3$) δ –75.9; **HRMS** (FAB$^+$) Calcd for $C_{20}H_{14}{}^{35}ClF_6IN_2O_4^+$ $[M]^+$ 621.9586, found 621.9593.

Ethyl 1-(3,3-bis(trifluoromethyl)-1λ^3-benzo[*d*][1,2]iodaoxol-1(3*H*)-yl)-5,6-dimethyl-1*H*-indazole-3-carboxylate (3ea)

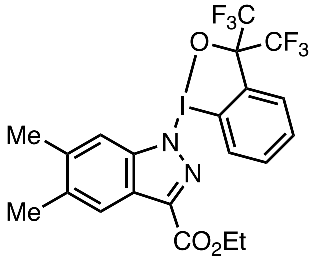

Colorless solid (25.1 mg, 45%); m.p. 175-178 °C; R_f 0.24 (hexane/EtOAc = 3/1); **^{1}H NMR** (400 MHz, $CDCl_3$) δ 8.01 (s, 1H), 7.80 (d, J = 7.7 Hz, 1H), 7.61 (app. t, J = 7.8 Hz, 1H), 7.43 (app. dt, J = 7.9, 1.3 Hz, 1H), 7.14 (s, 1H), 6.24 (d, J = 8.0 Hz, 1H), 4.54 (q, J = 7.2 Hz, 2H), 2.42 (s, 3H), 2.39 (s, 3H), 1.49 (t, J = 7.2 Hz, 3H); **^{13}C{^{1}H} NMR** (150 MHz, $CDCl_3$) δ 162.5, 146.9, 140.2, 138.3, 133.7, 131.3, 131.0, 130.2, 127.3, 123.1 (q, $J_{C\text{-}F}$ = 288.0 Hz), 122.1, 121.5, 116.0, 111.3, 84.4-84.0 (m), 61.3, 20.9, 20.2, 14.5; **^{19}F NMR** (376 MHz, $CDCl_3$) δ –75.9; **HRMS** (FAB$^+$) Calcd for $C_{21}H_{17}F_6IN_2O_3^+$ $[M]^+$ 586.0183, found 586.0195.

Ethyl 1-(3,3-bis(trifluoromethyl)-1λ^3-benzo[*d*][1,2]iodaoxol-1(3*H*)-yl)-1*H*-benzo[*f*]indazole-3-carboxylate (3fa)

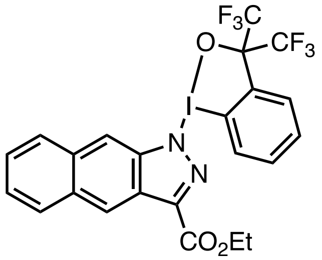

Yellow solid (14.0 mg, 23%); m.p. 131-134 °C; R_f 0.25 (hexane/EtOAc = 3/1); **^{1}H NMR** (400 MHz, $CDCl_3$) δ 8.86 (s, 1H), 8.08 (d, J = 8.3 Hz, 1H), 7.92 (d, J = 8.3 Hz, 1H), 7.85-7.80 (m, 2H), 7.61 (app. t, J = 7.7 Hz, 1H), 7.53-7.48 (m, 2H), 7.39 (app. dt, J = 7.9, 1.3 Hz, 1H), 6.36 (d, J = 8.4 Hz, 1H), 4.61 (q, J = 7.2 Hz, 2H), 1.54 (t, J = 7.2 Hz, 3H); **^{13}C{^{1}H} NMR** (150 MHz, $CDCl_3$) δ 162.2, 145.8, 141.0, 133.7, 133.0, 131.5, 131.2, 130.6, 130.4, 129.4, 127.8, 127.3, 126.9, 124.9, 123.3, 123.1 (q, J_{C-F} = 288.3 Hz), 121.6, 115.8, 107.1, 61.6, 14.5 (the signal for the carbon bonded to the CF_3 groups, which typically appear as a weak multiplet around 82-81 ppm, was not identified due to the low S/N ratio); **^{19}F NMR** (376 MHz, $CDCl_3$) δ –75.8; **HRMS** (FAB^+) Calcd for $C_{23}H_{15}F_6IN_2O_3^+$ $[M]^+$ 608.0026, found 608.0017.

Ethyl 1-(3,3-bis(trifluoromethyl)-1λ^3-benzo[*d*][1,2]iodaoxol-1(3*H*)-yl)-1*H*-benzo[*g*]indazole-3-carboxylate (3ga)

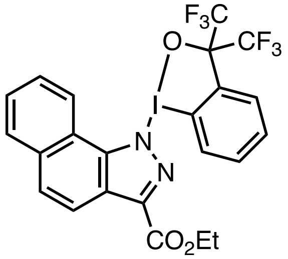

Colorless solid (27.0 mg, 22%; obtained as a 11:1 mixture of regioisomers; contains trace amounts of deiodinated byproducts (ethyl 1*H*-benzo[*g*]indazole-3-carboxylate and regioisomer), which were difficult to separate); m.p. 138-141 °C; R_f 0.61 (hexane/EtOAc = 3/1); **^{1}H NMR** (400 MHz, $CDCl_3$) δ 9.49 (d, J = 8.4 Hz, 1H), 7.90 (d, J = 7.8 Hz, 1H), 7.80 (d, J = 7.4 Hz, 1H), 7.75-7.70 (m, 2H), 7.62-7.55 (m, 2H), 7.39 (app. dt, J = 7.9 Hz, 1.3 Hz, 1H), 7.33 (d, J = 9.0 Hz, 1H), 6.15(d, J = 8.5 Hz, 1H), 4.62 (q, J = 7.1 Hz, 2H), 1.54 (t, J = 7.1 Hz, 3H); **^{13}C{^{1}H} NMR** (150 MHz, $CDCl_3$) δ 163.4, 146.4, 142.1, 133.9, 131.5, 131.1, 130.9, 130.7, 130.3, 128.8, 127.9, 127.3, 127.0, 126.5, 125.9, 123.1 (q, J_{C-F} = 287.3 Hz), 118.7, 116.1, 84.6-84.2 (m), 61.9, 14.5; **^{19}F NMR** (376 MHz, $CDCl_3$) δ –75.8; **HRMS** (FAB^+) Calcd for $C_{23}H_{15}F_6IN_2O_3^+$ $[M]^+$ 608.0026, found 608.0039.

Ethyl 1-(3,3-bis(trifluoromethyl)-1λ³-benzo[*d*][1,2]iodaoxol-1(3*H*)-yl)-6-methyl-1*H*-indazole-3-carboxylate (3ha) and ethyl 1-(3,3-bis(trifluoromethyl)-1λ³-benzo[*d*][1,2]iodaoxol-1(3*H*)-yl)-5-methyl-1*H*-indazole-3-carboxylate (3ha')

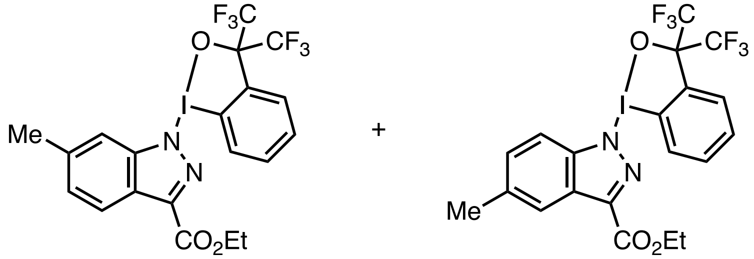

Colorless solid (39.1 mg, 46%, obtained as a 1:1 mixture of regioisomers); m.p. 144-147 °C; R_f 0.55 (hexane/EtOAc = 3/1); **^{1}H NMR** (400 MHz, $CDCl_3$) δ 8.12 (d, J = 8.3 Hz, 1H), 8.05 (s, 1H), 7.71-7.79 (m, 1H+1H), 7.64-7.59 (m, 1H+1H), 7.46-7.40 (m, 1H+1H), 7.29-7.28 (m, 1H+1H), 7.19 (d, J = 8.4 Hz, 1H), 7.15 (s, 1H), 6.28 (d, J = 8.5 Hz, 1H), 6.24 (d, J = 8.4 Hz, 1H), 4.59-4.50 (m, 2H+2H), 2.53 (s, 3H), 2.50 (s, 3H), 1.51-1.47 (m, 3H+3H); **^{13}C{^{1}H} NMR** (150 MHz, $CDCl_3$) δ 162.4, 162.3, 148.2, 146.3, 140.7, 140.2, 138.7, 133.8, 133.74, 133.71, 131.4, 131.0, 130.2, 129.9, 127.32, 127.30, 126.2, 123.8, 123.1 (q, $J_{C\text{-}F}$ = 287.3 Hz), 121.9, 121.6, 121.4, 115.92, 115.89, 111.2, 111.0, 84.4-84.0 (m), 61.3, 21.9, 21.4, 14.47, 14.45; **^{19}F NMR** (376 MHz, $CDCl_3$) δ –75.85, –75.86; **HRMS** (FAB$^+$) Calcd for $C_{20}H_{15}F_6IN_2O_3^+$ [M]$^+$ 572.0026, found 572.0023.

As discussed in the main text, the reaction employing Kobayashi aryne precursors often resulted in modest yields, and in some cases, failed to produce the desired indazolyl-BXs in synthetically meaningful yields (>5%) for unambiguous characterization. The problematic substrates and the corresponding challenges are summarized in Figure S2.

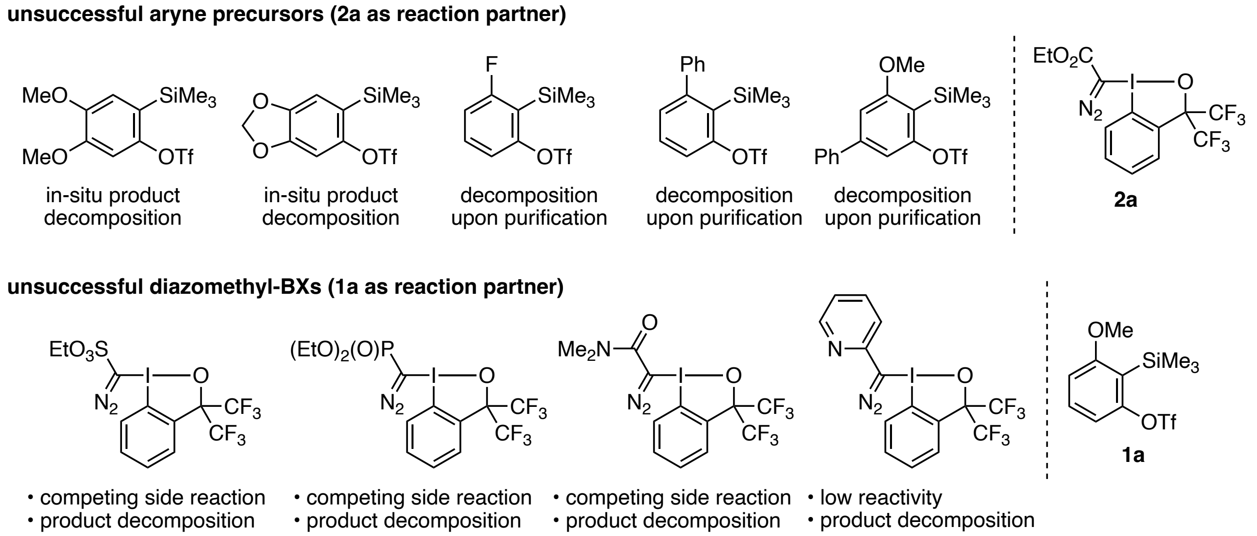

Figure S2. Summary of unsuccessful reactions using Kobayashi-type aryne precursors.

Table S3. Optimization of indazolyl-λ[3]-iodane synthesis from cyclic diarylhalonium salts

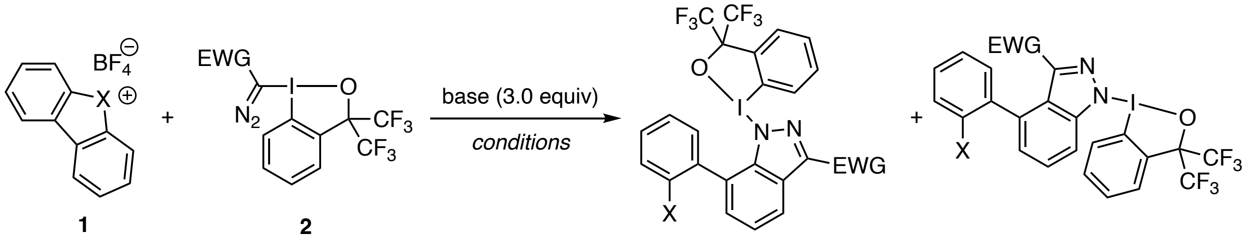

1 (0.05 mmol) **2** (2.0 equiv) **3** **3'**

entry	EWG	**2**	X	base	conditions	yield (**3**+**3'**)
1	CO_2Et	3.0 equiv	Cl	K_2CO_3	$CHCl_3$, rt, 18 h	68%
2	COPh	3.0 equiv	Cl	K_2CO_3	$CHCl_3$, rt, 18 h	85%
3	COPh	2.0 equiv	Cl	K_2CO_3	$CHCl_3$, rt, 18 h	88%[a]
4	COPh	1.5 equiv	Cl	K_2CO_3	$CHCl_3$, rt, 18 h	53%[a]
5	CO_2Et	3.0 equiv	Br	Cs_2CO_3	CH_2Cl_2, rt, 18 h	31%
6	CO_2Et	3.0 equiv	Br	K_2CO_3	$CHCl_3$, rt, 18 h	30%[a]
7	COPh	3.0 equiv	Br	Cs_2CO_3	CH_2Cl_2, rt, 18 h	71%

[a]Yields were determined by [1]NMR analysis using 1,1,2,2-tetrachloroethane as an internal standard.

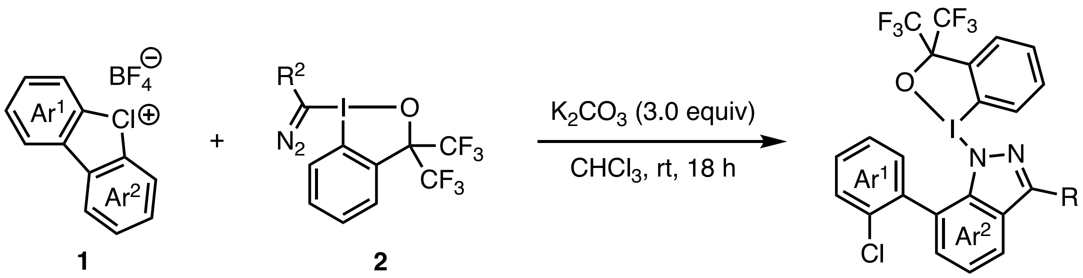

1 **2** (2.0 equiv) **3**

General Procedure D: In a 4 mL vial equipped with a magnetic stir bar, diazomethylbenziodoxole **2** (0.10 mmol, 2.0 equiv) was dissolved in chloroform (0.50 mL). To this solution were added cyclic diaryl λ[3]-chlorane (0.050 mmol, 1.0 equiv) and potassium carbonate (20.7 mg, 0.15 mmol, 3.0 equiv) at room temperature. The resulting mixture was stirred at the same temperature for 18 h under an argon atmosphere and then added chloroform and filtered. The filtrate was concentrated under reduced pressure, and the residue was purified by column chromatography (silica gel, 15 g, hexane/ethyl acetate = 10/1 to 5/1) to afford the desired product **3**.

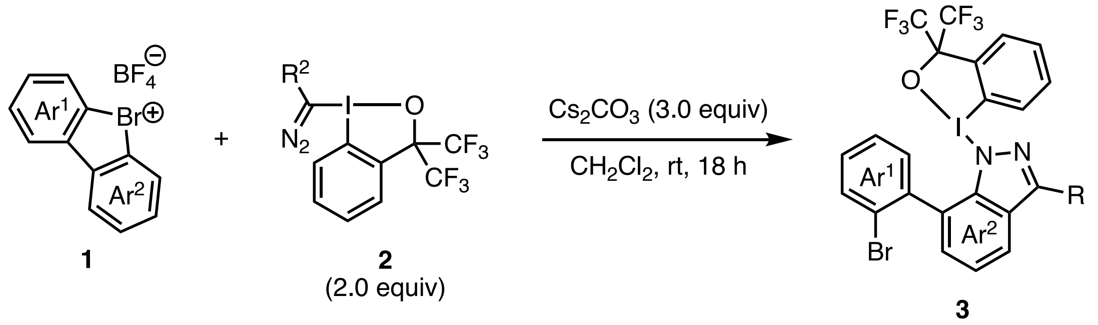

General Procedure E: In a 4 mL vial equipped with a magnetic stir bar, diazomethylbenziodoxole **2** (0.15 mmol, 3.0 equiv) was dissolved in dichloromethane (0.50 mL). To this solution were added cyclic diaryl λ^3-bromane (0.050 mmol, 1.0 equiv) and cesium carbonate (48.9 mg, 0.15 mmol, 3.0 equiv) at room temperature. The resulting mixture was stirred at the same temperature for 18 h under an argon atmosphere and added dichloromethane and filtered. The filtrate was concentrated under reduced pressure, and the residue was purified by column chromatography (silica gel, 15 g, hexane/ethyl acetate = 10/1 to 5/1) to afford the desired product **3**.

Ethyl 1-(3,3-bis(trifluoromethyl)-1λ^3-benzo[*d*][1,2]iodaoxol-1(3*H*)-yl)-7-(2-chlorophenyl)-1*H*-indazole-3-carboxylate (3ja)

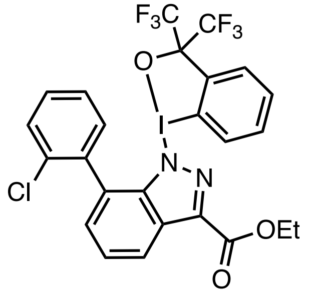

The reaction was performed using 3 equiv of diazomethyliodane **2a**. Colorless solid (22.8 mg, 68%; 77% yield according to ^{1}H NMR analysis of the crude product using 1,1,2,2-tetrachloroethane as an internal standard; regioisomer ratio = 10:1); m.p. 164-166 °C; *R*f 0.26 (hexane/EtOAc = 3/1); **^{1}H NMR** (400 MHz, $CDCl_3$) δ 8.41 (dd, *J* = 8.4, 1.2 Hz, 1H), 7.64 (d, *J* = 7.6 Hz, 1H), 7.55 (app. t, *J* = 7.5 Hz, 1H), 7.47-7.39 (m, 3H), 7.35-7.28 (m, 2H), 7.12-7.07 (m, 2H), 6.03 (d, *J* = 8.0 Hz, 1H), 4.61-4.52 (m, 2H), 1.50 (t, *J* = 7.1 Hz, 3H); **^{13}C{^{1}H} NMR** (150 MHz, $CDCl_3$) δ 162.3, 144.3, 141.5, 135.0, 134.7, 133.3, 131.7, 131.1, 130.7, 130.6, 129.8, 129.7, 128.9, 127.5, 126.9, 124.6, 123.6, 123.3, 122.8 (q, $J_{C\text{-}F}$ = 286.6 Hz), 122.2, 116.9, 61.4, 14.5 (the signal for the carbon bonded to the CF_3 groups, which typically appear as a weak multiplet around 82-81 ppm, was not identified due to the low S/N ratio); **^{19}F NMR** (376 MHz, $CDCl_3$) δ –75.8 (q, $J_{F\text{-}F}$ = 8.8 Hz), –76.0 (q, $J_{F\text{-}F}$ = 8.8 Hz); **HRMS** (FAB$^+$) Calcd for $C_{25}H_{17}{}^{35}ClF_6IN_2O_3{}^+$ [M+H]$^+$ 668.9871, found 668.9866. Note that the regiochemistry of the major products in this and the following examples, featuring sterically more congested iodane moiety, could be readily assigned by the diagnostic quartet pair in ^{19}F NMR, indicating the restricted rotation around the C–I bond (by contrast, all the products obtained from Kobayashi aryne precursors gave singlet signals in ^{19}F

NMR).[7,8] The regiochemistry of **3ha** was also confirmed by HMQC and HMBC spectra.

Ethyl 1-(3,3-bis(trifluoromethyl)-1λ³-benzo[*d*][1,2]iodaoxol-1(3*H*)-yl)-7-(2-bromophenyl)-1*H*-indazole-3-carboxylate (3ka)

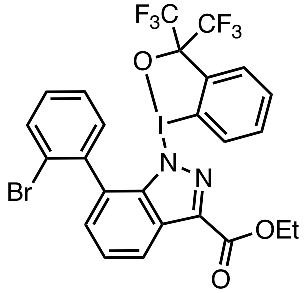

With 3.0 equiv of 2a: Colorless solid (12.7 mg, 36%; 61% yield according to ¹H NMR analysis of the crude product using 1,1,2,2-tetrachloroethane as an internal standard; regioisomer ratio = 3:1); m.p. 90-92 °C; R_f 0.34 (hexane/EtOAc = 4/1); **¹H NMR** (600 MHz, $CDCl_3$) δ 8.41 (d, J = 8.3 Hz, 1H), 7.67-7.61 (m, 2H), 7.55 (app. t, J = 7.5 Hz, 1H), 7.47-7.39 (m, 2H), 7.30-7.23 (m, 2H), 7.15-7.08 (m, 2H), 6.02 (d, J = 8.5 Hz, 1H), 4.62-4.52 (m, 2H), 1.50 (t, J = 7.1 Hz, 3H); **¹³C{¹H} NMR** (150 MHz, $CDCl_3$) δ 162.3, 144.1, 141.5, 137.0, 133.3, 132.9, 131.6, 131.1, 130.8, 130.6, 129.8, 128.8, 127.54, 127.51, 126.4, 125.2, 123.6, 123.3, 122.9 (q, $J_{C\text{-}F}$ = 286.9 Hz), 122.2, 117.0, 61.4, 14.5 (the signal for the carbon bonded to the CF_3 groups, which typically appear as a weak multiplet around 82-81 ppm, was not identified due to the low S/N ratio); **¹⁹F NMR** (376 MHz, $CDCl_3$) δ –75.8 (q, $J_{F\text{-}F}$ = 8.3 Hz), –76.1 (q, $J_{F\text{-}F}$ = 8.3 Hz); **HRMS** (FAB⁺) Calcd for $C_{25}H_{17}{}^{79}BrF_6IN_2O_3^+$ $[M+H]^+$ 712.9366, found 712.9366.

(1-(3,3-Bis(trifluoromethyl)-1λ³-benzo[*d*][1,2]iodaoxol-1(3*H*)-yl)-7-(2-chlorophenyl)-1*H*-indazol-3-yl)(phenyl)methanone (3jd)

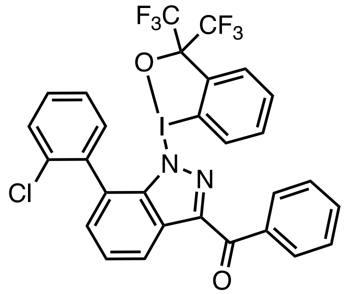

Colorless solid (29.7 mg, 85%, regioisomer ratio = 13:1); m.p. 69-72 °C; R_f 0.31 (hexane/EtOAc = 3/1); **¹H NMR** (400 MHz, $CDCl_3$) δ 8.61 (dd, J = 8.2, 1.1 Hz, 1H), 8.35 (dd, J = 8.2, 1.0 Hz, 2H), 7.66 (d, J = 7.7 Hz, 1H), 7.60-7.30 (m, 9H), 7.15-7.09 (m, 2H), 6.03 (d, J = 8.4 Hz, 1H); **¹³C{¹H} NMR** (101 MHz, $CDCl_3$) δ 188.4, 147.8, 144.0, 137.4, 135.2, 134.7, 133.4, 132.8, 131.7, 131.1, 130.7, 130.6, 129.9, 129.7, 129.1, 128.2, 127.4, 127.0, 124.3, 124.10, 124.07, 123.0, 122.9 (q, $J_{C\text{-}F}$ = 286.9 Hz), 116.7, 84.2-83.8 (m); **¹⁹FNMR** (376 MHz, $CDCl_3$) δ –75.8 (q, $J_{F\text{-}F}$ = 9.2 Hz), –76.0 (q, $J_{F\text{-}F}$ = 9.6 Hz); **HRMS** (FAB⁺) Calcd for $C_{29}H_{16}{}^{35}ClF_6IN_2O_2^+$ $[M]^+$ 699.9844, found

699.9854.

(1-(3,3-Bis(trifluoromethyl)-1λ^3-benzo[*d*][1,2]iodaoxol-1(3*H*)-yl)-7-(2-bromophenyl)-1*H*-indazol-3-yl)(phenyl)methanone (3kd)

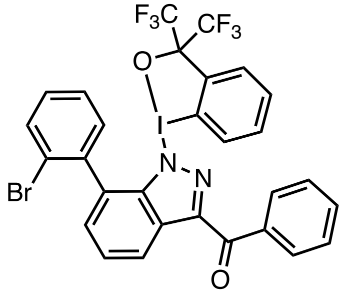

Colorless solid (27.6 mg, 71%, regioisomer ratio = 14:1); m.p. 154-157 °C; R_f 0.31 (hexane/EtOAc = 3/1); **^{1}H NMR** (400 MHz, $CDCl_3$) δ 8.61 (dd, *J* = 8.2, 1.0 Hz, 1H), 8.35 (d, *J* = 7.2 Hz, 2H), 7.65 (d, *J* = 8.1 Hz, 2H), 7.61-7.47 (m, 5H), 7.41 (app. dt, *J* = 7.8, 1.4 Hz, 1H), 7.31-7.24 (m, 2H), 7.17-7.11 (m, 2H), 6.01 (d, *J* = 8.5 Hz, 1H); **^{13}C{^{1}H} NMR** (101 MHz, $CDCl_3$) δ 188.4, 147.9, 143.8, 137.5, 137.2, 133.4, 132.93, 132.86, 131.7, 131.1, 130.8, 130.74, 130.69, 129.9, 128.9, 128.3, 127.6, 127.5, 126.1, 125.1, 124.11, 124.07, 123.0, 122.9 (q, $J_{C\text{-}F}$ = 288.3 Hz), 116.8, 84.2-83.8 (m); **^{19}FNMR** (376 MHz, $CDCl_3$) δ –75.8 (q, $J_{F\text{-}F}$ = 8.3 Hz), –76.1 (q, $J_{F\text{-}F}$ = 8.3 Hz); **HRMS** (FAB$^+$) Calcd for $C_{29}H_{16}{}^{79}BrF_6IN_2O_2^+$ $[M]^+$ 743.9339, found 743.9349.

(1-(3,3-Bis(trifluoromethyl)-1λ^3-benzo[*d*][1,2]iodaoxol-1(3*H*)-yl)-7-(2-chloro-3-methylphenyl)-1*H*-indazol-3-yl)(phenyl)methanone (3ld)

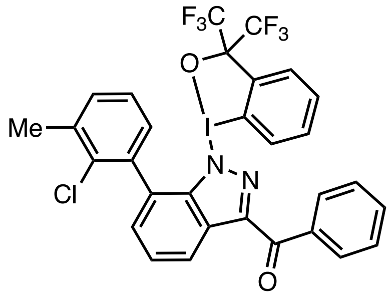

Colorless solid (54.8 mg, 75% (major regioisomer); regioisomer ratio = 13:1); m.p. 190-193 °C; R_f 0.62 (hexane/EtOAc = 3/1); **^{1}H NMR** (400 MHz, $CDCl_3$) δ 8.59 (dd, *J* = 8.2, 1.1 Hz, 1H), 8.35-8.33 (m, 2H), 7.66 (d, *J* = 7.7 Hz, 1H), 7.60-7.40 (m, 6H), 7.32-7.7.30 (m, 1H), 7.27-7.25 (m, 1H), 7.03-6.98 (m, 2H), 6.15 (dd, *J* = 8.4, 0.8 Hz, 1H), 2.32 (s, 3H); **^{13}C{^{1}H} NMR** (150 MHz, $CDCl_3$) δ 188.4, 147.9, 144.1, 137.5, 137.4, 135.3, 134.7, 133.3, 132.8, 131.8, 131.1, 130.7, 130.6, 129.8, 129.3, 128.9, 128.3, 127.6, 126.4, 125.0, 124.12, 124.09, 122.9 (q, $J_{C\text{-}F}$ = 290.1 Hz), 122.8, 116.5, 84.3-83.9 (m), 20.5; **^{19}FNMR** (376 MHz, $CDCl_3$) δ –75.9 (q, $J_{F\text{-}F}$ = 7.8 Hz), –76.1 (q, $J_{F\text{-}F}$ = 7.9 Hz); **HRMS** (FAB$^+$) Calcd for $C_{30}H_{18}{}^{35}ClF_6IN_2O_2^+$ $[M]^+$ 714.0000, found 714.0010.

Methyl 3-benzoyl-1-(3,3-bis(trifluoromethyl)-1λ³-benzo[*d*][1,2]iodaoxol-1(3*H*)-yl)-7-(2-bromo-3-methylphenyl)-1*H*-indazole-4-carboxylate (3md)

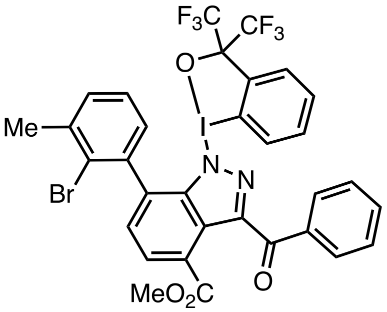

The reaction was performed using 2 equiv of diazomethyl-BX and 2 equiv of Cs_2CO_3 in THF (0.05 M). Colorless solid (58%; Yield was determined by ^{1}H NMR analysis using 1,1,2,2-tetrachloroethane as an internal standard; regioisomer ratio > 20:1); m.p. 101-104 °C; R_f 0.34 (hexane/EtOAc = 3/1); **^{1}H NMR** (400 MHz, $CDCl_3$) δ 8.09 (d, *J* = 8.1, 2H), 7.92 (d, *J* = 7.4 Hz, 1H), 7.66-7.55 (m, 3H), 7.51-7.49 (m, 3H), 7.35 (d, *J* = 7.4 Hz, 1H), 7.29 (d, *J* = 7.5 Hz, 1H), 7.07 (app. t, *J* = 7.6 Hz, 1H), 6.96 (d, *J* = 7.4 Hz, 1H), 6.20 (d, *J* = 8.3 Hz, 1H), 3.70 (s, 3H), 2.39 (s, 3H); **^{13}C{^{1}H} NMR** (150 MHz, $CDCl_3$) δ 189.7, 166.7, 149.2, 144.3, 139.7, 137.0, 133.45, 133.37, 131.9, 131.2, 131.0, 130.6, 130.3, 129.8, 128.8, 128.5, 128.3, 127.7, 127.2, 126.9, 125.3, 124.8, 122.9 (q, $J_{C\text{-}F}$ = 288.0 Hz), 120.0, 116.7, 84.3-83.9 (m), 51.8, 23.6; **^{19}FNMR** (376 MHz, $CDCl_3$) δ –75.9 (q, $J_{F\text{-}F}$ = 8.9 Hz), –76.2 (q, $J_{F\text{-}F}$ = 8.8 Hz); **HRMS** (FAB$^+$) Calcd for $C_{32}H_{21}{}^{79}BrF_6IN_2O_4{}^+$ $[M+H]^+$ 816.9628, found 816.9658.

Methyl 3-benzoyl-1-(3,3-bis(trifluoromethyl)-1λ³-benzo[*d*][1,2]iodaoxol-1(3*H*)-yl)-7-(2-bromo-phenyl)-1*H*-indazole-4-carboxylate (3nd) and Methyl 3-benzoyl-1-(3,3-bis(trifluoromethyl)-1λ³-benzo[*d*][1,2]iodaoxol-1(3*H*)-yl)-4-(2-bromophenyl)-1*H*-indazole-7-carboxylate (3nd')

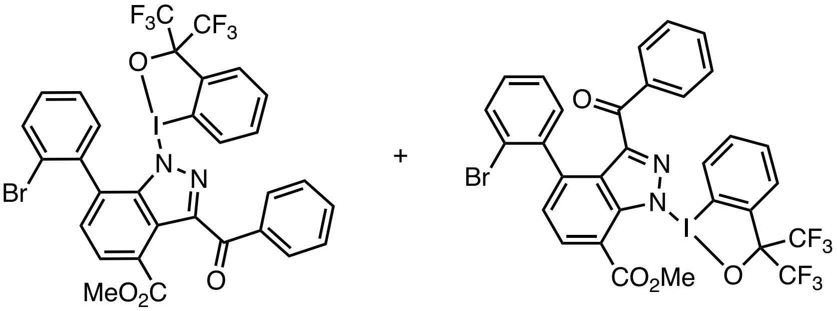

The reaction was performed using 2 equiv of diazomethyl-BX and 2 equiv of Cs_2CO_3 in THF (0.05 M). Colorless solid (67%; The yield was determined by ^{1}H NMR analysis using 1,1,2,2-tetrachloroethane as an internal standard because separation from the starting material was difficult by silica gel chromatography; regioisomer ratio = 1:1. A small amount of analytically pure sample was obtained by further purification on PTLC); m.p. 119-122 °C; R_f 0.50 (hexane/EtOAc = 3/1); **^{1}H NMR** (400 MHz, $CDCl_3$) δ 9.32 (d, *J* = 1.6 Hz, 1H), 8.64 (d, *J* = 8.6, 1.0 Hz, 1H), 8.39-8.35 (m, 4H), 7.99 (d, *J* = 1.6 Hz, 1H), 7.88 (d, *J* = 7.9, 2.1 Hz, 1H), 7.76-7.73 (m, 2H), 7.68-7.41 (m,

14H), 7.30 (d, J = 7.1 Hz, 2H), 7.19-7.11 (m, 2H), 6.12 (d, J = 8.4 Hz, 1H), 6.01 (d, J = 8.5 Hz, 1H), 3.99 (s, 3H), 3.74 (s, 3H); **^{13}C{^{1}H} NMR** (150 MHz, $CDCl_3$) δ188.3, 187.9, 166.8, 165.3, 148.8, 147.9, 145.6, 143.7, 137.6, 137.4, 137.1, 136.5, 133.7, 133.5, 133.1, 133.0, 132.6, 131.7, 131.4, 131.3, 131.1, 130.8, 130.6, 130.4, 130.0, 129.9, 129.6, 129.4, 129.1, 128.4, 128.3, 127.7, 127.4, 126.3, 126.0, 125.9, 125.0, 124.3, 124.1, 123.9, 123.8, 123.5, 122.8 (q, $J_{C\text{-}F}$ = 289.4 Hz), 116.6, 52.4, 52.2 (the signal for the carbon bonded to the CF_3 groups, which typically appear as a weak multiplet around 82-81 ppm, was not identified due to the low S/N ratio); **^{19}F NMR** (376 MHz, $CDCl_3$) δ –75.8 (q, $J_{F\text{-}F}$ = 8.6 Hz), –75.9 (q, $J_{F\text{-}F}$ = 8.8 Hz), –76.1 (q, $J_{F\text{-}F}$ = 8.1 Hz), –76.2 (q, $J_{F\text{-}F}$ = 8.8 Hz); **HRMS** (FAB^+) Calcd for $C_{31}H_{18}{}^{79}BrF_6IN_2O_4$ [M] 801.9399, found 801.9409.

(1-(3,3-Bis(trifluoromethyl)-1λ^3-benzo[*d*][1,2]iodaoxol-1(3*H*)-yl)-7-(2-chlorophenyl)-1*H*-indazol-3-yl)(*p*-tolyl)methanone (3jf)

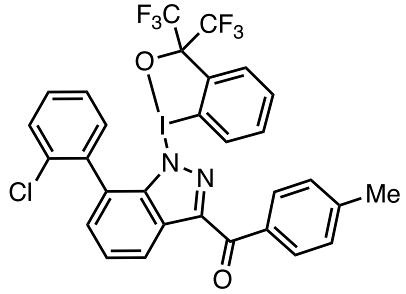

Colorless solid (23.9 mg, 64%; regioisomer ratio = 11:1); m.p. 148-151 °C; R_f 0.68 (hexane/EtOAc = 3/1); **^{1}H NMR** (400 MHz, $CDCl_3$) δ 8.60 (dd, J = 8.2, 1.1 Hz, 1H), 8.26 (d, J = 8.2 Hz, 2H), 7.65 (d, J = 7.5 Hz, 1H), 7.55 (app. dt, J = 7.5, 0.9 Hz, 1H), 7.50-7.29 (m, 7H), 7.14-7.12 (m, 2H), 6.01 (dd, J = 8.4, 0.7 Hz, 1H), 2.42 (s, 3H); **^{13}C{^{1}H} NMR** (150 MHz, $CDCl_3$) δ 188.1, 148.0, 144.7, 144.0, 143.8, 135.3, 134.9, 134.7, 133.4, 131.7, 131.4, 131.1, 130.9, 130.6, 129.9, 129.8, 129.0, 127.4, 127.0, 124.3, 124.1, 124.0, 123.1, 122.9 (q, $J_{C\text{-}F}$ = 286.9 Hz), 116.8, 21.7 (the signal for the carbon bonded to the CF_3 groups, which typically appear as a weak multiplet around 82-81 ppm, was not identified due to the low S/N ratio); **^{19}FNMR** (376 MHz, $CDCl_3$) δ –75.8 (q, $J_{F\text{-}F}$ = 8.3 Hz), –76.1 (q, $J_{F\text{-}F}$ = 9.4 Hz); **HRMS** (FAB^+) Calcd for $C_{30}H_{18}{}^{35}ClF_6IN_2O_2^+$ $[M]^+$ 714.0000, found 714.0023.

(1-(3,3-Bis(trifluoromethyl)-1λ^3-benzo[*d*][1,2]iodaoxol-1(3*H*)-yl)-7-(2-chlorophenyl)-1*H*-indazol-3-yl)(4-chlorophenyl)methanone (3jg)

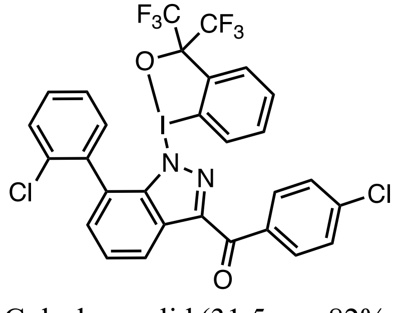

Colorless solid (31.5 mg, 82%, regioisomer ratio = 10:1); m.p. 142-145 °C; R_f 0.68 (hexane/EtOAc

= 3/1); **^{1}HNMR** (400 MHz, $CDCl_3$) δ 8.61 (dd, *J* = 8.2, 1.0 Hz, 1H), 8.34 (d, *J* = 8.6 Hz, 2H), 7.67 (d, *J* = 7.6 Hz, 1H), 7.58-7.32 (m, 8H), 7.15-7.12 (m, 2H), 6.00 (d, *J* = 8.4 Hz, 1H); **^{13}C{^{1}H} NMR** (150 MHz, $CDCl_3$) δ 187.0, 147.6, 144.0, 139.4, 135.7, 135.1, 134.7, 133.4, 132.2, 131.7, 131.2, 130.7, 130.0, 129.8, 129.2, 128.6, 127.3, 127.0, 124.4, 124.2, 124.1, 123.0, 122.9 (q, $J_{C\text{-}F}$ = 286.9 Hz), 116.6 (the signal for the carbon bonded to the CF_3 groups, which typically appear as a weak multiplet around 82-81 ppm, was not identified due to the low S/N ratio); **^{19}FNMR** (376 MHz, $CDCl_3$) δ –75.8 (q, $J_{F\text{-}F}$ = 8.9 Hz), –76.1 (q, $J_{F\text{-}F}$ = 8.8 Hz); **HRMS** (FAB^+) Calcd for $C_{29}H_{15}{}^{35}Cl_2F_6IN_2O_2{}^+$ $[M]^+$ 733.9454, found 733.9463.

(1-(3,3-Bis(trifluoromethyl)-1λ^3-benzo[*d*][1,2]iodaoxol-1(3*H*)-yl)-7-(2-chlorophenyl)-1*H*-indazol-3-yl)(2-methoxyphenyl)methanone (3ji)

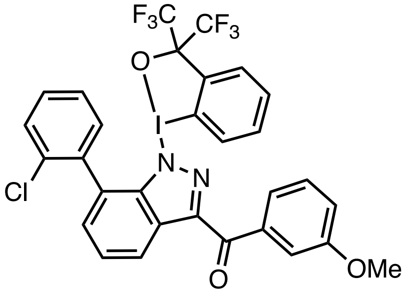

The reaction was performed using 2.6 equiv of diazomethyliodane **2h**. Colorless solid (21.0 mg, 58% (major regioisomer); 89% yield according to ^{1}H NMR analysis of the crude product using 1,1,2,2-tetrachloroethane as an internal standard; regioisomer ratio = 14:1); m.p. 180-183 °C; R_f 0.55 (hexane/EtOAc = 3/1); **^{1}H NMR** (400 MHz, $CDCl_3$) δ 8.60 (dd, *J* = 8.2, 1.0 Hz, 1H), 7.98 (d, *J* = 7.7 Hz, 1H), 7.88 (app. t, *J* = 1.5 Hz, 1H), 7.66 (d, *J* = 7.6 Hz, 1H), 7.56 (app. t, *J* = 7.0 Hz, 1H), 7.52-7.32 (m, 6H), 7.15-7.13 (m, 3H), 6.00 (d, *J* = 7.6 Hz, 1H), 3.80 (s, 3H); **^{13}C{^{1}H} NMR** (150 MHz, $CDCl_3$) δ 188.0, 159.4, 147.9, 144.0, 138.7, 135.2, 134.7, 133.4, 131.7, 131.1, 130.70, 130.68, 129.9, 129.8, 129.3, 129.1, 127.4, 127.1, 124.3, 124.1, 123.6, 123.0, 122.9 (q, $J_{C\text{-}F}$ = 287.7 Hz), 119.7, 116.8, 114.8, 55.3 (the signal for the carbon bonded to the CF_3 groups, which typically appear as a weak multiplet around 82-81 ppm, was not identified due to the low S/N ratio); **^{19}FNMR** (376 MHz, $CDCl_3$) δ –75.9 (q, $J_{F\text{-}F}$ = 8.8 Hz), –76.1 (q, $J_{F\text{-}F}$ = 8.9 Hz); **HRMS** (FAB^+) Calcd for $C_{30}H_{19}{}^{35}ClF_6IN_2O_3{}^+$ $[M+H]^+$ 731.0028, found 731.0043.

(1-(3,3-Bis(trifluoromethyl)-1λ^3-benzo[*d*][1,2]iodaoxol-1(3*H*)-yl)-7-(2-chlorophenyl)-1*H*-indazol-3-yl)(2-methoxyphenyl)methanone (3jj)

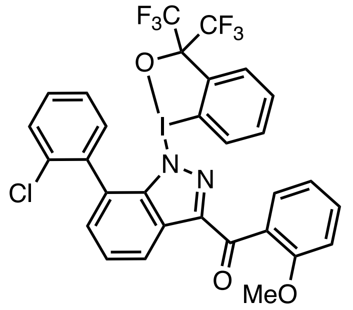

Colorless solid (25.6 mg, 66%, regioisomer ratio = >20:1); m.p. 145-148 °C; R_f 0.46 (hexane/EtOAc = 3/1); **^{1}H NMR** (400 MHz, $CDCl_3$) δ 8.53 (d, J = 8.1 Hz, 1H), 7.65-7.60 (m, 2H), 7.55 (app. t, J = 7.2 Hz, 1H), 7.49-7.40 (m, 4H), 7.35-7.28 (m, 2H), 7.13-6.98 (m, 4H), 6.00 (d, J = 8.4 Hz, 1H), 3.70 (s, 3H); **^{13}C{^{1}H} NMR** (150 MHz, $CDCl_3$) δ 190.3, 158.1, 148.5, 144.3, 135.2, 134.7, 133.2, 132.4, 131.7, 131.0, 130.7, 130.63, 130.59, 129.8, 129.7, 128.9, 127.4, 127.0, 124.3, 124.0, 123.3, 122.86, 122.85 (q, $J_{C\text{-}F}$ = 286.9 Hz), 120.2, 116.9, 111.8, 55.7 (the signal for the carbon bonded to the CF_3 groups, which typically appear as a weak multiplet around 82-81 ppm, was not identified due to the low S/N ratio); **^{19}FNMR** (376 MHz, $CDCl_3$) δ –75.9 (q, $J_{F\text{-}F}$ = 8.4 Hz), –76.1 (q, $J_{F\text{-}F}$ = 8.9 Hz); **HRMS** (FAB$^+$) Calcd for $C_{30}H_{18}{}^{35}ClF_6IN_2O_3^+$ [M]$^+$ 729.9949, found 729.9967.

(1-(3,3-Bis(trifluoromethyl)-1λ^3-benzo[*d*][1,2]iodaoxol-1(3*H*)-yl)-7-(2-chlorophenyl)-1*H*-indazol-3-yl)(3,4-dimethoxyphenyl)methanone (3jk)

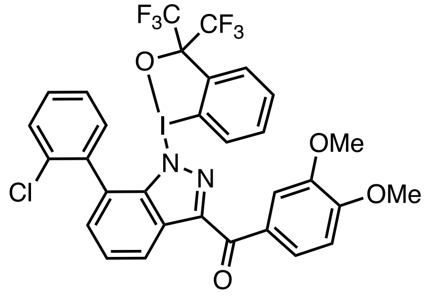

Colorless solid (21.0 mg, 58% (major regioisomer); regioisomer ratio = 14:1); m.p. 154-157 °C; R_f 0.55 (hexane/EtOAc = 3/1); **^{1}H NMR** (400 MHz, $CDCl_3$) δ 8.59 (d, J = 8.2, 1H), 8.20 (dd, J = 8.5, 1.9 Hz, 1H), 7.97 (d, J = 1.9 Hz, 1H), 7.67 (d, J = 7.4 Hz, 1H), 7.56 (app. t, J = 7.5 Hz, 1H), 7.50-7.31 (m, 5H), 7.17-7.14 (m, 2H), 6.95 (d, J = 8.6 Hz, 1H), 6.03 (d, J = 8.4 Hz, 1H), 3.95 (s, 3H), 3.86 (s, 3H); **^{13}C{^{1}H} NMR** (150 MHz, $CDCl_3$) δ 186.4, 153.3, 148.7, 148.2, 143.9, 135.2, 134.7, 133.3, 131.7, 131.1, 130.7, 130.6, 130.2, 129.9, 129.8, 129.0, 127.4, 127.0, 126.0, 124.24, 124.17, 123.9, 123.1, 122.9 (q, $J_{C\text{-}F}$ = 285.8 Hz), 120.0, 116.8, 112.7, 110.1, 84.4-83.8 (m), 56.0, 55.7; **^{19}FNMR** (376 MHz, $CDCl_3$) δ –75.9 (q, $J_{F\text{-}F}$ = 8.4 Hz), –76.2 (q, $J_{F\text{-}F}$ = 8.4 Hz); **HRMS** (FAB$^+$) Calcd for $C_{31}H_{21}{}^{35}ClF_6IN_2O_4^+$ [M+H]$^+$ 761.0133, found 761.0163.

(1-(3,3-Bis(trifluoromethyl)-1λ^3-benzo[*d*][1,2]iodaoxol-1(3*H*)-yl)-7-(2-chlorophenyl)-1*H*-indazol-3-yl)(naphthalen-2-yl)methanone (3jl)

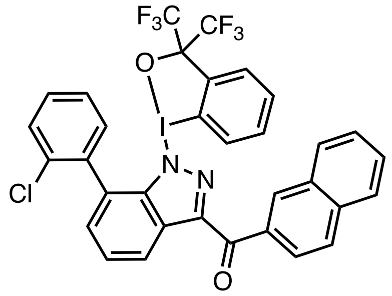

Colorless solid (25.4 mg, 63%, regioisomer ratio = 12:1); m.p. 148-151 °C; R_f 0.67 (hexane/EtOAc = 3/1); **^{1}H NMR** (400 MHz, $CDCl_3$) δ 8.98 (s, 1H), 8.64 (dd, J = 8.2, 1.0 Hz, 1H), 8.35 (dd, J = 8.7, 1.7 Hz, 1H), 7.95-7.92 (m, 2H), 7.88 (d, J = 8.1 Hz, 1H), 7.66 (d, J = 7.5 Hz, 1H), 7.60-7.33 (m, 8H), 7.18-7.12 (m, 2H), 6.05 (d, J = 8.5 Hz, 1H); **^{13}C{^{1}H} NMR** (150 MHz, $CDCl_3$) δ 188.3, 148.1, 144.1, 135.5, 135.3, 134.7, 133.4, 133.1, 132.5, 131.7, 131.2, 130.69, 130.68, 129.90, 129.87, 129.8, 129.1, 128.4, 128.1, 127.7, 127.4, 127.1, 126.5, 126.0, 124.4, 124.2, 124.1, 123.1, 122.9 (q, $J_{C\text{-}F}$ = 286.9 Hz), 116.8 (the signal for the carbon bonded to the CF_3 groups, which typically appear as a weak multiplet around 82-81 ppm, was not identified due to the low S/N ratio); **^{19}FNMR** (376 MHz, $CDCl_3$) δ –75.8 (q, $J_{F\text{-}F}$ = 8.3 Hz), –76.1 (q, $J_{F\text{-}F}$ = 8.9 Hz); **HRMS** (FAB^+) Calcd for $C_{33}H_{19}{}^{35}ClF_6IN_2O_2^+$ $[M+H]^+$ 751.0078, found 751.0072.

(1-(3,3-Bis(trifluoromethyl)-1λ^3-benzo[*d*][1,2]iodaoxol-1(3*H*)-yl)-7-(2-chlorophenyl)-1*H*-indazol-3-yl)(pyridin-2-yl)methanone (3jm)

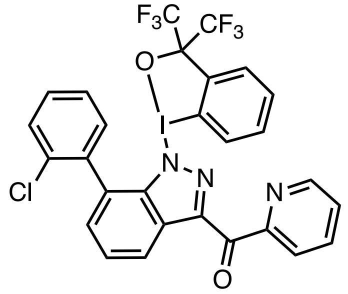

Yellow solid (86%; The yield was determined by ^{1}H NMR analysis using 1,1,2,2-tetrachloroethane as an internal standard because separation from the starting material was difficult by silica gel chromatography; regioisomer ratio = 11:1. A small amount of analytically pure sample was obtained by further purification on PTLC); R_f 0.16 (hexane/EtOAc = 3/1); **^{1}H NMR** (400 MHz, $CDCl_3$) δ 8.81 (d, J = 4.7 Hz, 1H), 8.54 (d, J = 8.2 Hz, 1H), 8.26 (d, J = 7.9 Hz, 1H), 7.89 (app. dt, J = 7.8, 1.7 Hz, 1H), 7.64 (d, J = 7.6 Hz, 1H), 7.56-7.30 (m, 7H), 7.11-7.10 (m, 2H), 6.03 (d, J = 7.7 Hz, 1H); **^{13}C{^{1}H} NMR** (150 MHz, $CDCl_3$) δ 187.8, 154.8, 149.6, 149.5, 147.4, 144.1, 136.7, 135.2, 134.7, 133.4, 131.7, 131.1, 130.6, 129.8, 129.1, 127.6, 126.9, 126.4, 125.4, 124.43, 124.41, 124.2, 124.1, 122.84 (q, $J_{C\text{-}F}$ = 286.2 Hz), 122.80, 116.9 (the signal for the carbon bonded to the CF_3 groups, which typically appear as a weak multiplet around 82-81 ppm, was not identified due to the low S/N ratio); **^{19}FNMR** (376 MHz, $CDCl_3$) δ –75.8 (q, $J_{F\text{-}F}$ = 8.8 Hz), –76.1 (q, $J_{F\text{-}F}$ = 8.8 Hz); **HRMS** (FAB^+) Calcd for $C_{28}H_{15}{}^{35}ClF_6IN_3O_2^+$ $[M+H]^+$ 701.9874, found 701.9882.

1-(3,3-Bis(trifluoromethyl)-1λ³-benzo[*d*][1,2]iodaoxol-1(3*H*)-yl)-7-(2-chlorophenyl)-*N*,*N*-dimethyl-1*H*-indazole-3-carboxamideethyl (3jn)

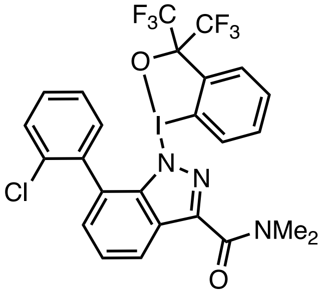

Colorless solid (22.0 mg, 72% (major regioisomer); regioisomer ratio = 4.5:1); m.p. 180-183 °C; R_f 0.45 (hexane/EtOAc = 1/1); **^{1}HNMR** (400 MHz, $CDCl_3$) δ 8.28 (dd, J = 8.2, 1.1 Hz, 1H), 7.66 (d, J = 7.7 Hz, 1H), 7.55 (app. t, J = 8.0 Hz, 1H), 7.44-7.26 (m, 5H), 7.12-7.11 (m, 2H), 6.01 (d, J = 8.4 Hz, 1H), 3.40 (s, 3H), 3.21 (s, 3H); **^{13}C{^{1}H} NMR** (150 MHz, $CDCl_3$) δ 163.6, 145.2, 143.7, 135.3, 134.7, 133.3, 131.7, 131.0, 130.6, 130.5, 129.82, 129.75, 128.9, 127.4, 127.0, 124.0, 123.9, 122.94 (q, $J_{C\text{-}F}$ = 300.2 Hz), 122.89, 122.5, 116.9, 39.1, 36.0 (the signal for the carbon bonded to the CF_3 groups, which typically appear as a weak multiplet around 82-81 ppm, was not identified due to the low S/N ratio); **^{19}FNMR** (376 MHz, $CDCl_3$) δ –75.9 (q, $J_{F\text{-}F}$ = 8.3 Hz), –76.2 (q, $J_{F\text{-}F}$ = 8.3 Hz); **HRMS** (FAB$^+$) Calcd for $C_{25}H_{17}{}^{35}ClF_6IN_3O_2{}^+$ $[M+H]^+$ 668.0031, found 668.0042.

Ethyl 1-(3,3-bis(trifluoromethyl)-1λ³-benzo[*d*][1,2]iodaoxol-1(3*H*)-yl)-7-(2-chlorophenyl)-1*H*-indazole-3-sulfonate (3jo)

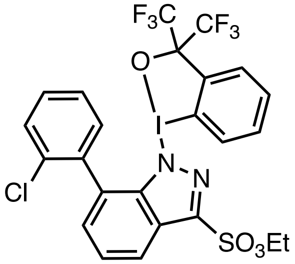

Colorless solid (21.5 mg, 61% (major regioisomer); regioisomer ratio = 16:1); m.p. 161-164 °C; R_f 0.53 (hexane/EtOAc = 3/1); **^{1}H NMR** (400 MHz, $CDCl_3$) δ 8.23 (d, J = 8.3 Hz, 1H), 7.67 (d, J = 7.9 Hz, 1H), 7.59 (app. t, J = 7.5 Hz, 1H), 7.51-7.43 (m, 3H), 7.37-7.33 (m, 2H), 7.14-7.12 (m, 2H), 6.05 (d, J = 8.4 Hz, 1H), 4.39 (q, J = 7.2 Hz, 2H), 1.38 (t, J = 7.0 Hz, 3H); **^{13}C{^{1}H} NMR** (150 MHz, $CDCl_3$) δ 144.9, 144.3, 134.61, 134.56, 133.5, 131.7, 131.4, 130.9, 130.7, 130.0, 129.8, 129.6, 127.4, 127.1, 124.9, 124.2, 122.7 (q, $J_{C\text{-}F}$ = 286.2 Hz), 121.1, 120.7, 116.5, 68.4, 14.9 (the signal for the carbon bonded to the CF_3 groups, which typically appear as a weak multiplet around 82-81 ppm, was not identified due to the low S/N ratio); **^{19}FNMR** (376 MHz, $CDCl_3$) δ –75.8 (q, $J_{F\text{-}F}$ = 8.3 Hz), –76.1 (q, $J_{F\text{-}F}$ = 8.8 Hz); **HRMS** (FAB$^+$) Calcd for $C_{24}H_{16}{}^{35}ClF_6IN_2O_4S^+$ $[M+H]^+$ 704.9541, found 704.9520.

Diethyl (1-(3,3-bis(trifluoromethyl)-1λ^3-benzo[*d*][1,2]iodaoxol-1(3*H*)-yl)-7-(2-chlorophenyl)-1*H*-indazol-3-yl)phosphonate (3jp)

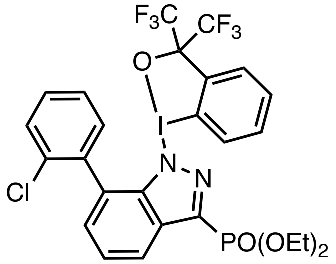

The reaction was performed using 3 equiv of diazomethyl-BX **2p**. Colorless solid (89%; The yield was determined by ^{1}H NMR analysis using 1,1,2,2-tetrachloroethane as an internal standard because separation from the starting material was difficult by silica gel chromatography; regioisomer ratio > 20:1. A small amount of analytically pure sample was obtained by purification on PTLC); m.p. 221-224 °C; R_f 0.53 (hexane/EtOAc = 1/2); **^{1}HNMR** (400 MHz, $CDCl_3$) δ 8.24 (d, J = 7.2 Hz, 1H), 7.66 (d, J = 7.6 Hz, 1H), 7.56 (dd, J = 7.8 Hz, J = 7.2 Hz, 1H), 7.43-7.26 (m, 5H), 7.11-7.10 (m, 2H), 5.97 (d, J = 8.4 Hz, 1H), 4.35-4.26 (m, 4H), 1.41-1.36 (m, 6H); **^{13}C{^{1}H} NMR** (150 MHz, $CDCl_3$) δ 143.6 (d, $J_{C\text{-}P}$ = 9.0 Hz), 135.2, 134.6, 133.1, 131.6, 131.1, 130.7, 130.6, 129.8, 129.7, 128.9, 127.4, 127.0, 126.0, 125.8, 124.4, 123.1, 122.9 (q, $J_{C\text{-}F}$ = 287.7 Hz), 121.6, 116.9, 84.2-83.8 (m), 62.9 (t, $J_{C\text{-}P}$ = 5.7 Hz), 16.4, (d, $J_{C\text{-}P}$ = 6.1 Hz); **^{19}FNMR** (376 MHz, $CDCl_3$) δ –75.9 (q, $J_{F\text{-}F}$ = 8.3 Hz), –76.2 (q, $J_{F\text{-}F}$ = 8.4 Hz); **^{31}P NMR** (243 MHz, $CDCl_3$) δ 9.3; **HRMS** (FAB$^+$) Calcd for $C_{26}H_{22}{}^{35}ClF_6IN_2O_4P^+$ [M+H]$^+$ 732.9949, found 732.9978.

Procedure for the 1 mmol-scale synthesis of 3ba

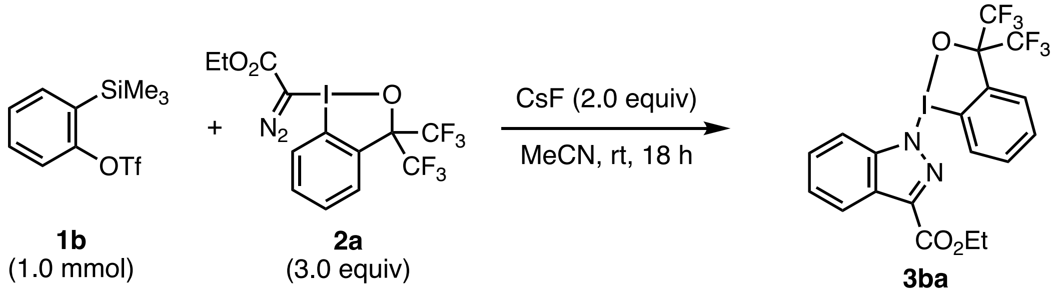

In a 50 mL two-necked flask equipped with a magnetic stir bar, ethyl 2-(3,3-bis(trifluoromethyl)-1λ^3-benzo[*d*][1,2]iodaoxol-1(3*H*)-yl)-2-diazoacetate (**2a**; 1.44 g, 3.0 mmol, 3.0 equiv) was dissolved in acetonitrile (10 mL). To this solution were added *o*-silylaryl triflate (**1b**; 0.30 g, 1.0 mmol, 1.0 equiv) and cesium fluoride (0.30 g, 2.0 mmol, 2.0 equiv) at room temperature. The resulting mixture was stirred at the same temperature for 18 h under an argon atmosphere, added ethyl acetate, and filtered. The filtrate was concentrated under reduced pressure, and the residue was purified by column chromatography (silica gel, 25 g, hexane/ethyl acetate = 10/1 to 5/1) to afford the desired product (**3ba**; 288 mg, 0.52 mmol, 51%) as a colorless solid.

Ball mill reaction for the synthesis of 3ha

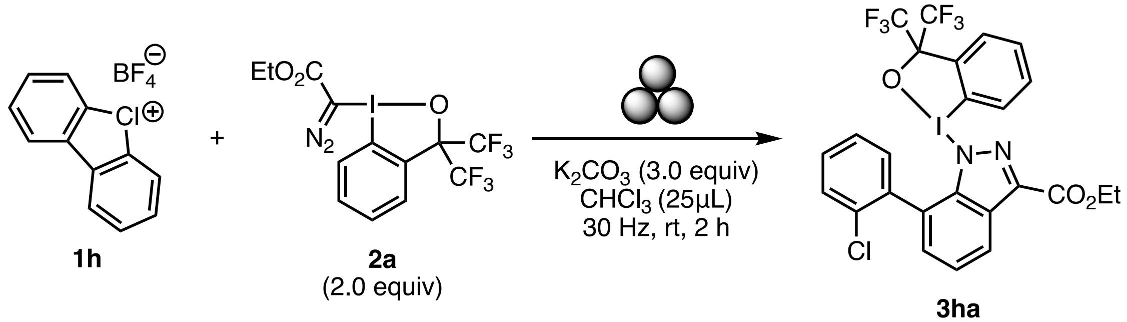

A 1.5 mL stainless miller jar equipped with a 4 mm stainless ball was charged sequentially with cyclic diaryl λ^3-chlorane (**1h**; 7.4 mg, 0.027 mmol, 1.0 equiv), ethyl 2-(3,3-bis(trifluoromethyl)-1λ^3-benzo[*d*][1,2]iodaoxol-1(3*H*)-yl)-2-diazoacetate (**2a**; 25.6 mg, 0.053 mmol, 2.0 equiv), K_2CO_3 (12.9 mg, 0.093 mmol, 3.5 equiv), and $CHCl_3$ (25 μL). This jar was closed, and the mixture was subjected to 30 Hz milling for 2 h. The mixture was then dissolved in $CHCl_3$ (4 mL), and the solution was concentrated under reduced pressure. To the residue was added 1,1,2,2-tetrachloroethane (15.6 mg, 92.9 μmol) as an internal standard, and the mixture was dissolved in $CDCl_3$. The solution was analyzed by ^{1}H NMR to determine the yields of ethyl 1-(3,3-bis(trifluoromethyl)-1λ^3-benzo[*d*][1,2]iodaoxol-1(3*H*)-yl)-7-(2-chlorophenyl)-1*H*-indazole-3-carboxylate (**3ha**; 60%).

4. C(sp³)–H Indazolylation of *N*,*N*-Dimethylanilines with Indazolyl-λ³-iodanes

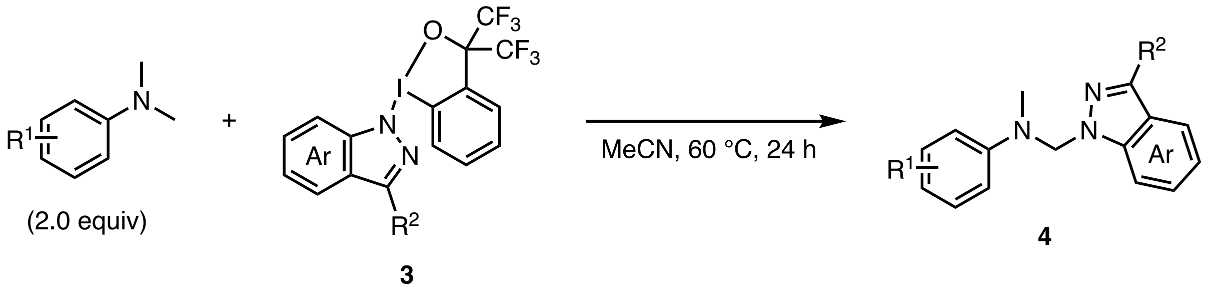

General procedure F: In a 4 mL vial equipped with a magnetic stir bar, indazolyl-λ³-iodine **3** (0.10 mmol, 1.0 equiv) and dimethylaniline (0.20 mmol, 2.0 equiv) were dissolved in acetonitrile (1.0 mL). The reaction mixture was stirred at 60 °C for 24 h under an argon atmosphere, added ethyl acetate, and filtered. The filtrate was concentrated under reduced pressure, and the residue was purified by column chromatography (NH silica gel, 16 g, hexane/ethyl acetate = 10/1 to 5/1) to afford the desired product **4**.

Ethyl 1-(((4-bromophenyl)(methyl)amino)methyl)-1*H*-indazole-3-carboxylate (4a)

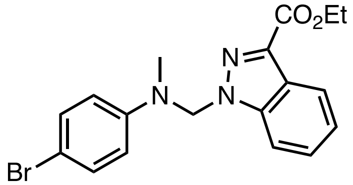

Colorless solid (34.6 mg, 88%); m.p. 129-132 °C; R_f 0.22 (hexane/EtOAc = 3/1); **¹H NMR** (400 MHz, $CDCl_3$) δ 8.20 (d, *J* = 8.5 Hz, 1H), 7.40-7.21 (m, 5H), 6.87 (d, *J* = 8.8 Hz, 2H), 5.96 (s, 2H), 4.53 (q, *J* = 7.2 Hz, 2H), 3.01 (s, 3H), 1.49 (t, *J* = 7.2 Hz, 3H); **¹³C{¹H} NMR** (150 MHz, $CDCl_3$) δ 162.6, 147.1, 140.3, 135.5, 132.1, 127.2, 124.0, 123.2, 122.3, 116.3, 111.7, 110.2, 68.3, 61.1, 37.6, 14.4; **HRMS** (FAB^+) Calcd for $C_{18}H_{18}{}^{79}BrN_3O_2^+$ $[M]^+$ 387.0577, found 387.0582.

Ethyl 1-((methyl(phenyl)amino)methyl)-1*H*-indazole-3-carboxylate (4b)

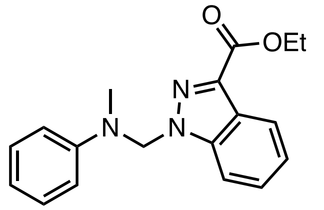

Colorless solid (34.2 mg, quant); m.p. 91-94 °C; R_f 0.44 (hexane/EtOAc = 3/1); **¹H NMR** (400 MHz, $CDCl_3$) δ 8.19 (d, *J* = 7.0 Hz, 1H), 7.35-7.18 (m, 5H), 7.00 (d, *J* = 8.1 Hz, 2H), 6.89 (app. t, *J* = 7.3 Hz, 1H), 6.00 (s, 2H), 4.54 (q, *J* = 7.2 Hz, 2H), 3.00 (s, 3H), 1.49 (t, *J* = 7.2 Hz, 3H); **¹³C{¹H} NMR** (150 MHz, $CDCl_3$) δ 162.7, 148.2, 140.4, 135.3, 129.4, 126.9, 124.1, 123.1, 122.1, 119.6, 115.0, 110.5, 68.8, 61.0, 37.2, 14.4; **HRMS** (FAB^+) Calcd for $C_{18}H_{19}N_3O_2^+$ $[M]^+$ 309.1472, found 309.1468.

(7-(2-Chlorophenyl)-1-((methyl(*p*-tolyl)amino)methyl)-1*H*-indazol-3-yl)(phenyl)methanone (4c)

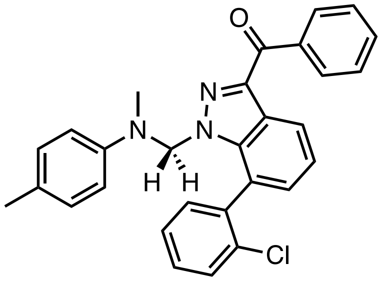

Yellow oil (32.0 mg, 82%); R_f 0.71 (hexane/EtOAc = 3/1); **^{1}H NMR** (400 MHz, $CDCl_3$) δ 8.58 (dd, *J* = 8.2, 1.0 Hz, 1H), 8.11 (d, *J* = 7.2 Hz, 2H), 7.55-7.50 (m, 3H), 7.44-7.40 (m, 3H), 7.36-7.28 (m, 3H), 7.00 (d, *J* = 8.2 Hz, 2H), 6.65 (d, *J* = 8.6 Hz, 2H), 5.60 (d, *J* = 13.9 Hz, 1H), 5.35 (d, *J* = 13.9 Hz, 1H), 2.77 (s, 3H), 2.26 (s, 3H); **^{13}C{^{1}H} NMR** (150 MHz, $CDCl_3$) δ 188.2, 145.7, 141.9, 138.6, 137.6, 137.5, 134.3, 132.2, 132.0, 130.8, 130.0, 129.8, 129.5, 129.4, 128.2, 127.8, 127.2, 125.3, 123.4, 123.3, 122.7, 114.8, 67.5, 38.5, 20.3; **HRMS** (FAB$^+$) Calcd for $C_{29}H_{24}{}^{35}ClN_3O^+$ [M]$^+$ 465.1602, found 465.1592.

***N*-((1*H*-Indazol-1-yl)methyl)-*N*,4-dimethylaniline (4d)**

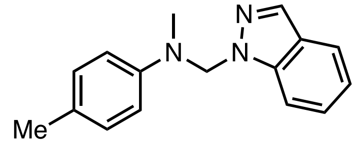

Brown oil (79%, Yield was determined by ^{1}H NMR analysis based on 1,1,2,2-tetrachloroethane); R_f 0.79 (hexane/EtOAc = 3/1); **^{1}H NMR** (400 MHz, $CDCl_3$) δ 7.99 (s, 1H), 7.70 (d, *J* = 8.1 Hz, 1H), 7.28-7.26 (m, 2H), 7.13-7.07 (m, 3H), 6.92 (d, *J* = 8.7 Hz, 2H), 5.84 (s, 2H), 2.97 (s, 3H), 2.27 (s, 3H); **^{13}C{^{1}H} NMR** (150 MHz, $CDCl_3$) δ 146.4, 139.5, 133.4, 129.8, 128.6, 126.4, 124.4, 121.0, 120.7, 115.3, 109.7, 67.7, 37.6, 20.4; **HRMS** (FAB$^+$) Calcd for $C_{16}H_{17}N_3^+$ [M]$^+$ 251.1417, found 251.1416.

Independent synthesis of indazolyl-BX from parent indazole:
1-(3,3-Bis(trifluoromethyl)-1λ^3-benzo[*d*][1,2]iodaoxol-1(3*H*)-yl)-1*H*-indazole (3x)

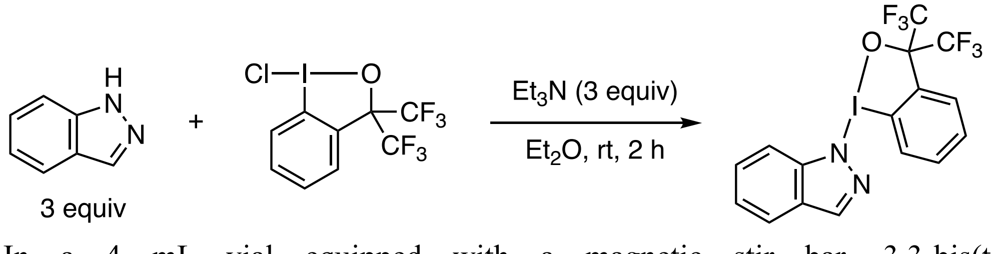

In a 4 mL vial equipped with a magnetic stir bar, 3,3-bis(trifluoromethyl)-1λ^3-benzo[*d*][1,2]iodaoxol-1(3*H*)-yl chloride (80.8 mg, 0.20 mmol, 1.0 equiv) was dissolved in Et_2O (1.0 mL). To this solution were added indazole (70.8 mg, 0.60 mmol, 3.0 equiv) and Et_3N (60.7 mg, 0.60 mmol, 3.0 equiv) at room temperature. The resulting mixture was stirred at the same

temperature for 2 h under an argon atmosphere. The mixture was concentrated under reduced pressure, and the residue was purified by column chromatography (silica gel, 18 g, hexane/ethyl acetate = 9/1 to 5/1) to afford the desired product **3x**.

Colorless solid (40.0 mg, 41%); m.p. 138-141 °C; R_f 0.71 (hexane/EtOAc = 3/1); **^{1}H NMR** (400 MHz, $CDCl_3$) δ 8.20 (d, J = 1.0 Hz, 1H), 7.82-7.78 (m, 2H), 7.58 (app. dt, J = 7.6, 0.9 Hz, 1H), 7.43-7.37 (m, 3H), 7.23 (app. dt, J = 7.2, 1.2 Hz, 1H), 6.21 (dd, J = 8.4, 0.7 Hz, 1H); **^{13}C{^{1}H} NMR** (150 MHz, $CDCl_3$) δ 146.4, 139.5, 133.4, 131.21, 131.15, 130.1, 127.6, 127.1, 124.1, 123.2 (q, J_{C-F} = 289.8 Hz), 122.0, 121.2, 116.3, 111.2, 84.3-83.5 (m); **^{19}F NMR** (376 MHz, $CDCl_3$) δ –75.9; **HRMS** (FAB$^+$) Calcd for $C_{16}H_{10}F_6IN_2O^+$ [M+H]$^+$ 486.9737, found 486.9744.

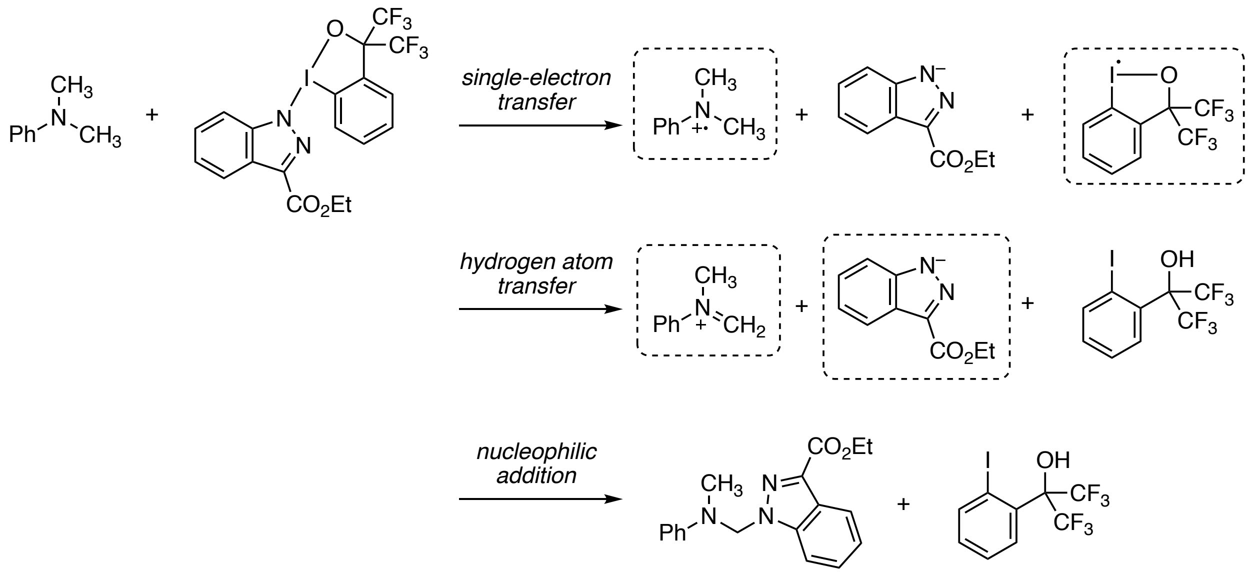

Scheme S1. Putative mechanism for the C(sp^3)–H indazolylation reaction

5. Competition Experiments to Evaluate Arynophilicity of Diazomethyl-λ^3-iodane

Reaction of *o*-silylaryl triflate 1b with a mixture of diazomethyl-λ^3-iodane 2a and furan

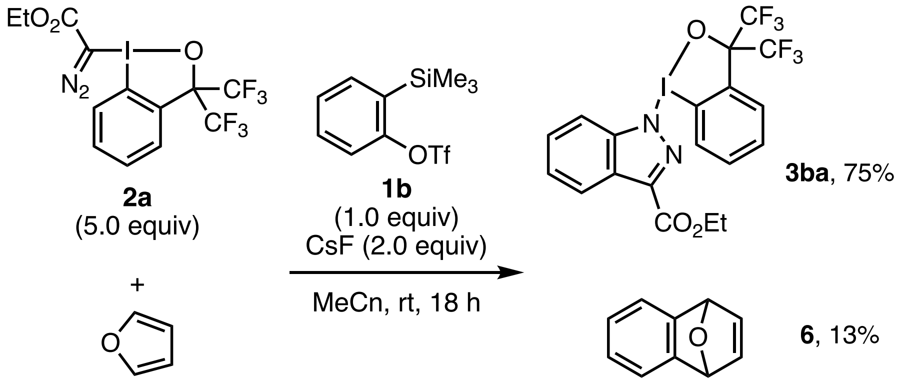

In a 4 mL vial equipped with a magnetic stir bar, ethyl 2-(3,3-bis(trifluoromethyl)-1λ^3-benzo[*d*][1,2]iodaoxol-1(3*H*)-yl)-2-diazoacetate (**2a**; 48.1 mg, 0.10 mmol, 5.1 equiv) and furan (6.8 mg, 5.0 mmol, 5.0 equiv) were dissolved in MeCN (1.0 mL). To this solution were added 2-(trimethylsilyl)phenyl trifluoromethanesulfonate (**1b**; 6.0 mg, 20.1 μmol, 1.0 equiv) and cesium fluoride (6.1 mg, 40 μmol, 2.0 equiv). The resulting mixture was stirred at room temperature for 18 h and then added ethyl acetate and filtered. The filtrate was concentrated under reduced pressure. To the residue was added 1,1,2,2-tetrachloroethane (15.6 mg, 92.9 μmol) as an internal standard, and the mixture was dissolved in $CDCl_3$. The solution was analyzed by ^1H NMR to determine the yields of ethyl 1-(3,3-bis(trifluoromethyl)-1λ^3-benzo[*d*][1,2]iodaoxol-1(3*H*)-yl)-1*H*-indazole-3-carboxylate (**3ba**; 75.0%) and 1,4-dihydro-1,4-epoxynaphthalene (**6**; 12.5%).

$$\log (k_{\mathbf{2a}}/k_{\text{furan}}) = \log (75.0/12.5) = 0.778$$

Reaction of *o*-silylaryl triflate 1b with a mixture of diazomethyl-λ^3-iodane 2a and benzyl azide

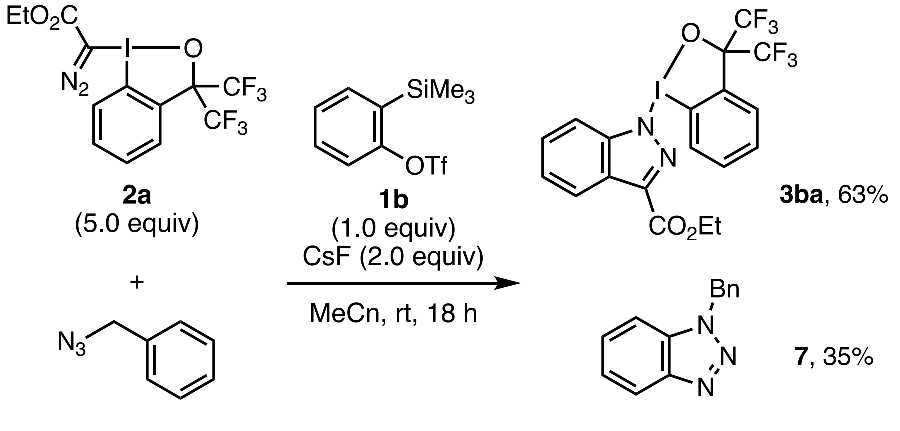

In a 4 mL vial equipped with a magnetic stir bar, ethyl 2-(3,3-bis(trifluoromethyl)-1λ^3-benzo[*d*][1,2]iodaoxol-1(3*H*)-yl)-2-diazoacetate (**2a**; 48.1 mg, 0.10 mmol, 5.1 equiv) and benzyl azide (13.3 mg, 0.10 mmol, 5.0 equiv) were dissolved in MeCN (0.80 mL). To this solution were added 2-(trimethylsilyl)phenyl trifluoromethanesulfonate (**1b**; 6.0 mg, 20.1 μmol, 1.0 equiv) and cesium fluoride (6.1 mg, 40 μmol, 2.0 equiv). The resulting mixture was stirred at room temperature for 18 h and then added ethyl acetate and filtered. The filtrate was concentrated under reduced pressure. To the residue was added 1,1,2,2-tetrachloroethane (16.5 mg, 98.3 μmol) as an internal standard, and the mixture was dissolved in $CDCl_3$. The solution was analyzed by ^{1}H NMR to determine the yields of ethyl 1-(3,3-bis(trifluoromethyl)-1λ^3-benzo[*d*][1,2]iodaoxol-1(3*H*)-yl)-1*H*-indazole-3-carboxylate (**3ba**; 62.5%) and 1-benzyl-1*H*-benzo[*d*][1,2,3]triazole (**7**; 35.0%).

$$\log (k_{\mathbf{2a}}/k_{\text{azide}}) = \log (62.5/35.0) = 0.252$$

6. X-Ray Crystallographic Analysis

X-ray crystal structure analysis of 3aa

Single crystals suitable for X-ray crystallography were obtained by recrystallization from CH_2Cl_2/hexane. A suitable crystal was selected and mounted on a Bruker D8 goniometer diffractometer. The crystal was kept at 99.8(3) K during data collection. Using Olex2,[9] the structure was solved with the olex2.solve[10] structure solution program using Charge Flipping and refined with the SHELXL[11] refinement package using Least Squares minimization.

Crystallographic data of **3aa** has been deposited on Cambridge Crystallographic Data Center, deposition no. CCDC 2404102.

Table S4. Crystal data and structure refinements for ethyl 1-(3,3-bis(trifluoromethyl)-1λ^3-benzo[*d*][1,2]iodaoxol-1(3*H*)-yl)-7-methoxy-1*H*-indazole-3-carboxylate (**3aa**):

CCDC number	2404102
Empirical formula	$C_{20}H_{15}F_6IN_2O_4$
Formula weight	588.24
Space system	triclinic
Space group	P-1
a/Å	8.32510(10)
b/Å	8.88170(10)
c/Å	14.9449(2)
α/°	73.4200(10)
β/°	86.6070(10)
γ/°	82.9770(10)
Volume/Å^3	1050.81(2)
Z	2
Temperature/K	99.8(3)
2Θ range for data collection/°	2.407 to 31.405
ρ_{calcd} g/cm^3	1.859
μ/mm^{-1}	1.608
F_000	576
Crystal_size/mm^3	0.2 x 0.2 x 0.05
Radiation	Mo K\a
Reflections collected	61227
Independent	6176
Index ranges	$-12 \leqq h \leqq 11$, $-12 \leqq k \leqq 12$, $-19 \leqq l \leqq 21$
Data/restraints/parameters	6176/0/300
Final R indexes R [I>2σ(I)]gt	R_1 = 0.0183, wR_2 = 0.0410
Final R indexes R [all data]	R_1 = 0.0204, wR_2 = 0.0417

Goodness-of-fit on F^2	1.050
Largest peak/deepest hole eÅ^{-3}	0.569/-0.351

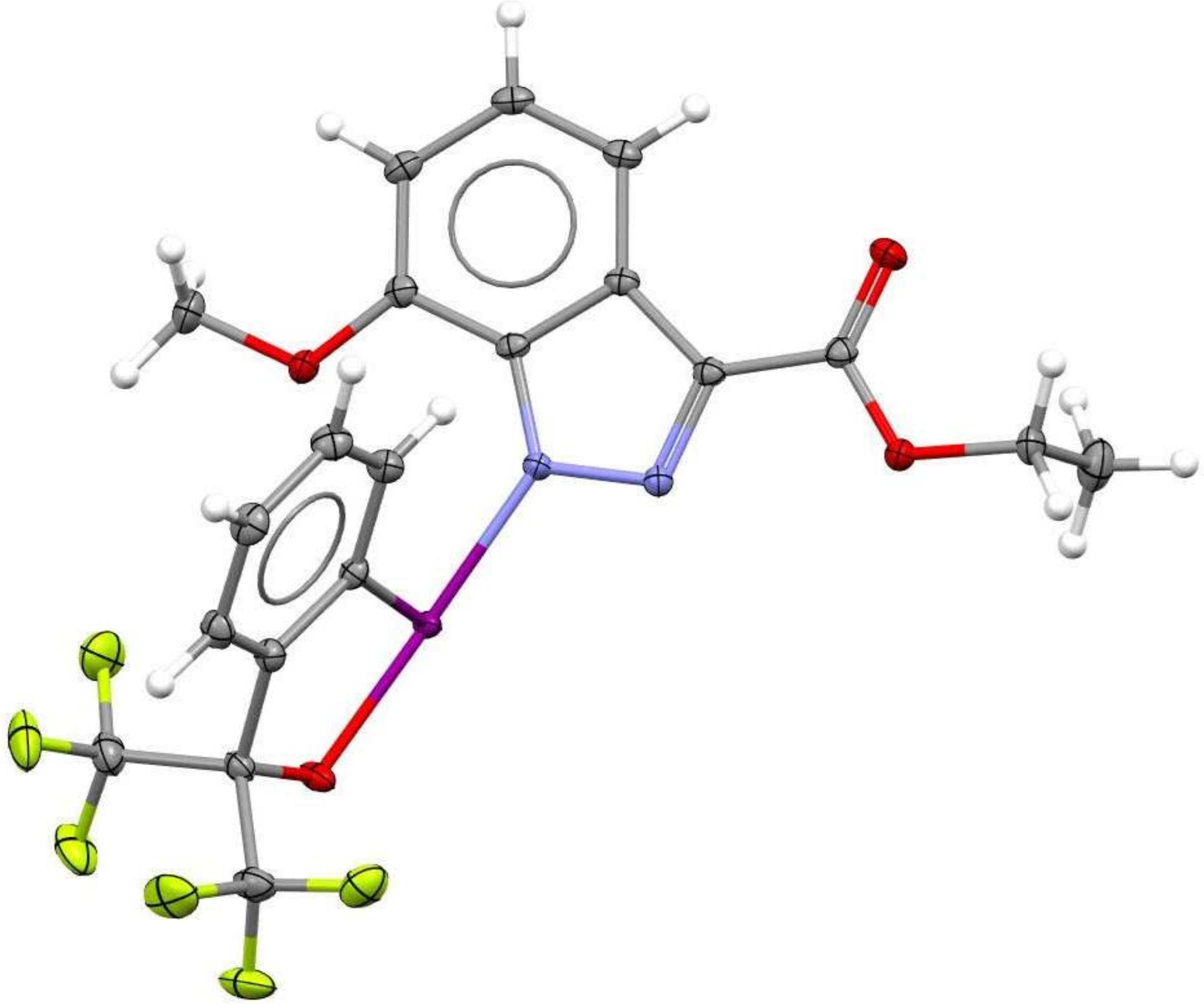

Figure S3. Thermal ellipsoid (50 % probability) plot of **3aa**, CCDC No. 2404102. Color code of atoms: hydrogen, white; carbon, gray; fluorine, yellow; iodine, purple; oxygen, red; nitrogen, blue.

7. DFT Calculations

Computational Method

All the density functional theory (DFT) calculations were carried out using the Gaussian 16 program.[12] Geometry optimizations were performed with the M06-2X functional[13,14,15] and a combined basis set B1 (i.e., the SDD effective core potential[16] for iodine and the 6-31G(d) basis set for all other atoms). Harmonic frequency calculations were performed for each stationary point to ensure that it is either an energy minimum (no imaginary frequency) or a transition state (only one imaginary frequency). For each transition state, intrinsic reaction coordinate (IRC)[17] analysis was performed to ensure that it connects the correct reactant and product. For the stationary points on the reaction pathways, single-point energy calculations were further performed with the M06-2X functional and a combined basis set B2 (i.e., the SDD effective core potential for iodine, the 6-311++G(2df,2p) basis set for all other atoms). The SMD model[18] with acetonitrile as the solvent was used for all the calculations. The single-point energies corrected by the thermal correction to Gibbs free energies (TCG, obtained from frequency calculations) were used as the Gibbs free energies reported in this work, corresponding to the reference state of 1 mol/L, 298.15 K. The natural population analysis (NPA) charges are calculated at the M06-2X/6-31G(d)-SDD(for I)/SMD(MeCN) level. The 3-D structures were drawn using CYLView software.[19]

Regioselectivity of [3+2] Cycloaddition with Unsymmetrical Arynes

3-Methoxybenzyne. No transition state could be located for the [3+2] cycloaddition between 3-methoxybenzyne and diazomethyl-BX (**SM**) leading to the experimentally observed regioselectivity. On the other hand, a transition state (**TS1-OMe-minor**) is located for [3+2] cycloaddition with the opposite regioselectivity (Figure S4a). Figure S4b illustrates the calculated energy profile (M06-2X/B1) for the approach of **SM** toward 3-methoxybenzyne, obtained by a series of structural optimizations at fixed distances (*d*) between the distal aryne carbon and the diazomethyl carbon, as referenced to the energy of **TS1-OMe-minor**. This profile demonstrates the monotonously downhill potential energy surface, which is ca. 3 kcal mol^{-1} below **TS-OMe1-minor**.

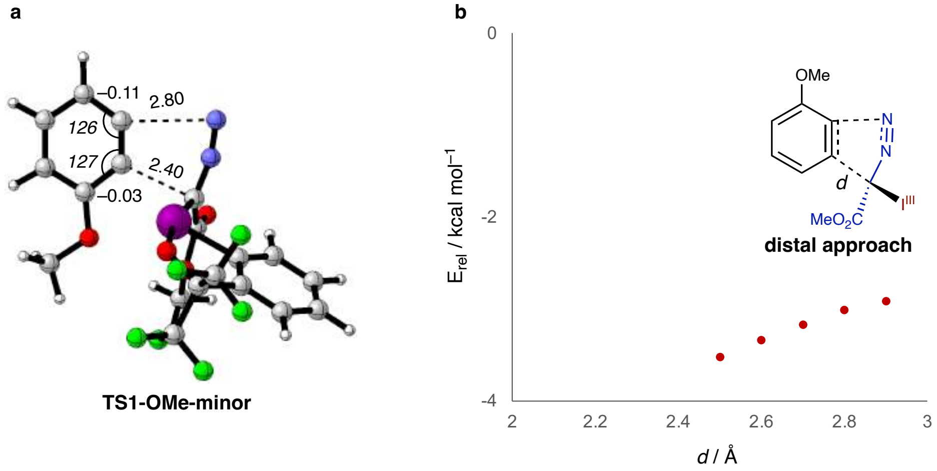

Figure S4. [3+2] Cycloaddition between 3-methoxybenzyne and diazomethyl-BX (**SM**). (a) Optimized structure of the disfavored transition state (**TS1-OMe-minor**). (b) Calculated energy profile for the distal approach of **SM** to 3-methoxybenzyne, where *d* represents the distance between the distal aryne carbon and the diazomethyl carbon, and E_{rel} corresponds to relative energy of the optimized structure at a fixed distance, referenced against the energy of **TS1-OMe-minor**.

3-(2-Chlorophenyl)benzyne. Two regioisomeric transition states were located for the [3+2] cycloaddition between 3-(2-chlorophenyl)benzyne and SM (Figure S5). In accordance with the experimental observations, the transition state (**TS1-ClPh-major**) leading to the observed regioselectivity (i.e., addition of the diazomethyl carbon to the distal aryne carbon) was energetically favored over the other (**TS1-ClPh-minor**).

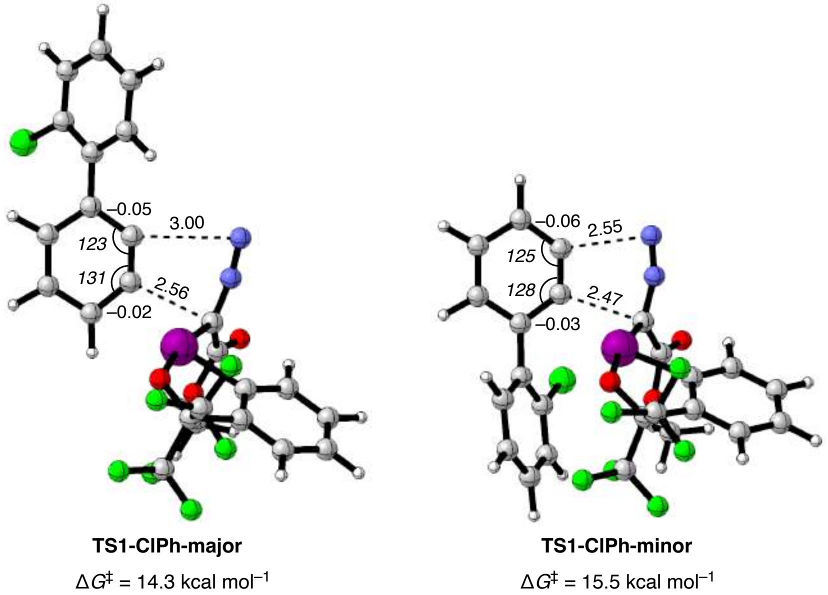

Figure S5. Transition states for the [3+2] cycloaddition between 3-(2-chlorophenyl)benzyne and diazomethyl-BX (**SM**).

Table S5. Energy data for the optimized structures (Figures 1, S4, and S5; hartrees).

Structure	E(M06-2X/ B1)	TCG	E(M06-2X/ B2)	TCG+ E(M06-2X/B2)	Imaginary Frequency
**SM**	-1406.44390819	0.135966	-1407.00819623	-1406.87223	
**benzyne**	-230.810363	0.048845	-230.8919608	-230.8431158	
**TS1**	-1637.26058723	0.205328	-1637.90963989	-1637.704312	63.89i
**INT1**	-1637.42177752	0.216189	-1638.06100877	-1637.84482	
**TS1’**	-1637.25956604	0.205911	-1637.90530196	-1637.699391	105.67i
**INT1’**	-1637.40082721	0.214291	-1638.04655508	-1637.832264	
**TS2**	-1637.41446713	0.216024	-1638.05033227	-1637.834308	134.23i
**INT2**	-1637.44654344	0.216854	-1638.08713060	-1637.870277	
**TS3**	-1637.40567704	0.215837	-1638.04706302	-1637.831226	177.19i
**PD**	-1637.44938898	0.216078	-1638.09471074	-1637.878633	
**TS1-OMe-minor**	-1751.74097728	0.236015			123.84i
**3-(2-chlorophenyl) benzyne**	-921.335989726	0.112095	-921.544540359	-921.4324454	
**TS1-ClPh-major**	-2327.79067265	0.268870	-2328.55074974	-2328.28187974	20.42i
**TS1-ClPh-minor**	-2327.79607120	0.275274	-2328.55518939	-2328.27991539	199.56i

Cartesian Coordinates

SM

C	2.941121	-0.662568	-0.219923
C	3.766935	0.349211	0.438619
O	4.920734	0.589413	0.164566
O	3.059679	0.996710	1.378427
C	3.784459	2.013818	2.071432
H	4.126929	2.778543	1.371290
H	3.083779	2.434421	2.790185
H	4.649639	1.583258	2.579371
N	3.525072	-1.307397	-1.195650
N	4.003149	-1.885353	-2.035942
I	0.881767	-1.147521	0.267128
C	0.163576	0.588195	-0.797416
C	1.015586	1.417533	-1.499283
C	-1.195460	0.793755	-0.679364
C	0.459828	2.527218	-2.132670
H	2.078533	1.212874	-1.563663
C	-1.730310	1.921173	-1.310192
C	-0.906200	2.775578	-2.034440
H	1.101263	3.194077	-2.699342
H	-2.791378	2.123477	-1.231878
H	-1.335676	3.643384	-2.523797
C	-2.012240	-0.207325	0.153322
C	-3.218498	-0.709556	-0.665601
C	-2.483932	0.499023	1.441144
F	-2.799799	-1.178037	-1.844968
F	-3.857486	-1.687171	-0.032956
F	-4.112963	0.266349	-0.909378
F	-3.181162	1.619264	1.195428
F	-3.246140	-0.298995	2.184692
F	-1.409790	0.843537	2.163807
O	-1.278840	-1.301502	0.499229

Benzyne

C	-0.702881	1.052759	-0.000025
C	-1.460779	-0.132405	0.000110
C	-0.622474	-1.232516	-0.000070
C	0.622482	-1.232517	-0.000070
C	1.460779	-0.132398	0.000112
C	0.702876	1.052762	-0.000024
H	-1.224697	2.005983	-0.000132
H	-2.544747	-0.133049	0.000040
H	2.544747	-0.133035	0.000034
H	1.224687	2.005989	-0.000137

TS1

C	4.351859	-1.138625	-1.109381
C	3.678658	-0.793978	-0.102682
C	5.254884	-2.197254	-1.058919
C	3.650675	-1.295887	1.182937
C	5.325441	-2.804800	0.204483
H	5.862106	-2.544983	-1.888764
C	4.549465	-2.369736	1.292595
H	3.041910	-0.939725	2.007820
H	6.005213	-3.639925	0.350860
H	4.646885	-2.876787	2.248436
C	2.083963	1.067736	-0.535008
C	2.531692	1.994808	0.509326
O	3.309143	2.904892	0.345600
O	1.952914	1.688606	1.681666
C	2.341319	2.529185	2.770726
H	2.093575	3.569628	2.551926
H	1.779713	2.171179	3.631462
H	3.415907	2.446619	2.945331
N	2.661973	1.204342	-1.701100
N	3.228202	1.130555	-2.670842
I	0.450009	-0.371260	-0.371189
C	-1.010168	1.210215	-0.478040
C	-0.659978	2.525648	-0.716845
C	-2.305926	0.789000	-0.254091
C	-1.679100	3.475704	-0.734975
H	0.369498	2.817278	-0.893187
C	-3.311548	1.759394	-0.259546
C	-2.996674	3.091612	-0.504065
H	-1.434710	4.514803	-0.929357
H	-4.337764	1.466242	-0.074501
H	-3.787337	3.834401	-0.512775
C	-2.539444	-0.705109	0.014051
C	-3.675064	-1.235018	-0.884350
C	-2.898880	-0.882422	1.503837
F	-3.434669	-0.912647	-2.158101
F	-3.778542	-2.556185	-0.801032
F	-4.871085	-0.714145	-0.551124
F	-3.946510	-0.133259	1.878806
F	-3.179543	-2.150465	1.793213
F	-1.848891	-0.506982	2.248213
O	-1.430879	-1.449236	-0.263607

INT1

C	3.941560	-0.693045	-1.239692
C	3.437746	-0.381875	0.027249

C	4.866443	-1.709900	-1.442202
C	3.838840	-1.107297	1.146620
C	5.274523	-2.426424	-0.321557
H	5.243809	-1.925975	-2.435836
C	4.767819	-2.127861	0.951242
H	3.455821	-0.873425	2.134276
H	5.995217	-3.230217	-0.430497
H	5.109604	-2.704612	1.804856
C	2.454928	0.678414	-0.243518
C	2.323434	1.874877	0.645066
O	2.070113	2.995114	0.282571
O	2.491987	1.522109	1.929971
C	2.343840	2.592434	2.867522
H	1.339345	3.015041	2.800246
H	2.511281	2.149718	3.847476
H	3.076830	3.375342	2.664970
N	2.573471	0.965488	-1.656569
N	3.377830	0.168959	-2.211517
I	0.466387	-0.564951	-0.021843
C	-0.829137	0.989897	-0.777279
C	-0.353620	2.129691	-1.397097
C	-2.167571	0.712857	-0.566216
C	-1.302314	3.067433	-1.806053
H	0.702875	2.293922	-1.572544
C	-3.093462	1.675566	-0.972929
C	-2.657818	2.844439	-1.588637
H	-0.965723	3.973407	-2.298745
H	-4.150881	1.501597	-0.813974
H	-3.386894	3.582488	-1.906240
C	-2.534383	-0.616777	0.099148
C	-3.647057	-1.328530	-0.697675
C	-2.996715	-0.341320	1.544371
F	-3.315884	-1.391272	-1.988315
F	-3.838310	-2.566011	-0.254851
F	-4.822421	-0.680073	-0.605859
F	-3.993756	0.551131	1.604855
F	-3.403791	-1.453736	2.150957
F	-1.964535	0.158058	2.240552
O	-1.469967	-1.481520	0.130465

TS1’

I	-0.413858	-0.473770	-0.320355
C	0.940651	1.195190	-0.420473
C	0.531118	2.500352	-0.638955
C	2.263483	0.849201	-0.210457
C	1.498703	3.502110	-0.655290

H	-0.510344	2.752169	-0.799058
C	3.219134	1.868612	-0.215889
C	2.836497	3.185551	-0.442691
H	1.197537	4.529315	-0.832695
H	4.260256	1.626351	-0.042382
H	3.588187	3.967759	-0.450366
C	2.587699	-0.628406	0.045516
C	3.704208	-1.099818	-0.907928
C	3.020648	-0.791633	1.516683
F	3.371224	-0.811614	-2.169269
F	3.895520	-2.410876	-0.817048
F	4.881357	-0.500831	-0.649474
F	3.364868	-2.047507	1.788981
F	4.056123	-0.002900	1.843022
F	1.993488	-0.457794	2.308809
O	1.509184	-1.435758	-0.180902
C	-3.114236	-2.070136	-0.099689
C	-4.005756	-3.133349	0.101126
C	-3.605870	-0.902614	-0.163081
C	-5.356417	-2.776293	0.200400
H	-3.699014	-4.172808	0.174790
C	-4.878648	-0.387349	-0.088149
C	-5.784442	-1.441317	0.109245
H	-6.101065	-3.553134	0.351557
H	-5.168779	0.655886	-0.156047
H	-6.842578	-1.211323	0.194639
C	-2.140323	0.883673	-0.525888
N	-2.545027	1.195095	-1.737608
N	-2.888601	1.404852	-2.787436
C	-2.641640	1.783988	0.519651
O	-3.484981	2.636889	0.344643
O	-2.052322	1.524629	1.689958
C	-2.524043	2.312132	2.785969
H	-3.592348	2.144227	2.935556
H	-1.954896	1.977832	3.650875
H	-2.349948	3.372194	2.591894

INT1'

I	-0.459192	-0.192350	1.401093
C	0.242254	-1.168436	-0.393789
C	-0.600398	-1.931766	-1.182034
C	1.568759	-0.932393	-0.687879
C	-0.079864	-2.468623	-2.357550
H	-1.631456	-2.113947	-0.897019
C	2.065606	-1.465602	-1.881347
C	1.246074	-2.227352	-2.706516

H	-0.715764	-3.074227	-2.995256
H	3.096911	-1.282820	-2.157877
H	1.647356	-2.637315	-3.627341
C	2.393023	-0.087035	0.306124
C	3.754654	-0.764436	0.567419
C	2.603378	1.309459	-0.318871
F	3.572530	-2.043780	0.907695
F	4.408463	-0.161183	1.554300
F	4.559394	-0.745350	-0.514571
F	3.376530	2.079421	0.445003
F	3.152315	1.260459	-1.541266
F	1.409267	1.915764	-0.441934
O	1.765639	0.037436	1.499295
C	-2.534849	-0.648323	0.922421
C	-3.218779	-1.500128	1.783295
C	-3.193896	-0.076420	-0.176931
C	-4.560647	-1.806837	1.557487
H	-2.701547	-1.945679	2.629166
C	-4.536712	-0.399109	-0.396343
C	-5.215850	-1.261291	0.459803
H	-5.083608	-2.477566	2.231680
H	-5.052076	0.043682	-1.244131
H	-6.258211	-1.497250	0.271377
C	-2.487894	0.805926	-1.138142
N	-2.415445	0.410614	-2.390092
N	-2.342893	0.064488	-3.459145
C	-1.850436	2.104262	-0.894292
O	-1.240349	2.732382	-1.727407
O	-2.042772	2.502298	0.373452
C	-1.322778	3.686524	0.729141
H	-1.631009	4.521766	0.097996
H	-1.571448	3.877911	1.771721
H	-0.250142	3.518843	0.608686

TS2

C	3.377368	-1.639039	-0.459627
C	3.998013	-0.503721	0.117895
C	3.913786	-2.936786	-0.302229
C	5.200772	-0.650367	0.842221
C	5.068876	-3.056951	0.430063
H	3.426567	-3.794028	-0.755491
C	5.709069	-1.919614	0.994872
H	5.694903	0.213690	1.270393
H	5.513430	-4.036123	0.578160
H	6.624822	-2.063854	1.559789
C	3.154081	0.568179	-0.278521

C	3.314321	2.009992	-0.038453
O	2.655999	2.897829	-0.535375
O	4.322693	2.246693	0.819241
C	4.568341	3.625479	1.088652
H	3.686758	4.088147	1.537431
H	5.407495	3.645922	1.781985
H	4.816385	4.155350	0.166500
N	2.156761	0.093849	-1.034766
N	2.284005	-1.253777	-1.164624
I	-0.136880	-1.044510	-0.673245
C	-0.899338	0.975016	-0.595633
C	-0.166214	2.077814	-0.960634
C	-2.209676	1.009625	-0.143006
C	-0.788655	3.320725	-0.829657
H	0.854986	1.996036	-1.311276
C	-2.805755	2.264329	-0.013964
C	-2.093781	3.410785	-0.357806
H	-0.230239	4.211423	-1.097606
H	-3.823335	2.337550	0.351451
H	-2.568918	4.380620	-0.254739
C	-2.873698	-0.313369	0.197795
C	-4.266974	-0.420907	-0.454338
C	-2.970359	-0.465097	1.728462
F	-4.183530	-0.131728	-1.752208
F	-4.764436	-1.645200	-0.328177
F	-5.135146	0.434851	0.108418
F	-3.614129	0.561959	2.292808
F	-3.595693	-1.587642	2.071656
F	-1.731981	-0.504309	2.236656
O	-2.152490	-1.387539	-0.309839

INT2

C	3.962861	-1.486350	-0.030700
C	4.139054	-0.074413	0.023748
C	5.055855	-2.350544	-0.291394
C	5.419727	0.495021	-0.187085
C	6.282030	-1.775214	-0.490257
H	4.905087	-3.424069	-0.327948
C	6.461706	-0.359407	-0.438104
H	5.557919	1.569296	-0.145030
H	7.144676	-2.402388	-0.692210
H	7.456291	0.043904	-0.601707
C	2.848941	0.408622	0.293906
C	2.310149	1.760427	0.430167
O	1.132840	2.026078	0.551300
O	3.281155	2.685207	0.392638

C	2.823466	4.033257	0.506917
H	2.306848	4.178909	1.457680
H	3.717117	4.652541	0.455289
H	2.138700	4.272388	-0.309308
N	2.037797	-0.681118	0.375888
N	2.673338	-1.827106	0.188497
I	-0.070232	-0.866736	0.945778
C	-0.624487	-0.119230	-0.975447
C	0.284016	0.116170	-1.988216
C	-1.983490	0.100534	-1.081112
C	-0.217340	0.617893	-3.187531
H	1.341244	-0.087523	-1.862817
C	-2.461086	0.623095	-2.285100
C	-1.578565	0.874164	-3.330010
H	0.464847	0.807212	-4.009648
H	-3.518864	0.826746	-2.400002
H	-1.959127	1.271992	-4.264745
C	-2.848431	-0.216563	0.140864
C	-4.079662	-1.049651	-0.264734
C	-3.275452	1.107380	0.806249
F	-3.697565	-2.102705	-0.988974
F	-4.734787	-1.491508	0.802078
F	-4.942799	-0.331830	-1.004515
F	-3.898873	1.926087	-0.051480
F	-4.089439	0.894956	1.838073
F	-2.184132	1.736269	1.254662
O	-2.163719	-0.977159	1.063807

TS3

C	3.801087	-1.225417	-0.994369
C	3.811657	-0.286894	0.069428
C	4.730199	-2.282341	-1.042531
C	4.757395	-0.396187	1.113003
C	5.639565	-2.375435	-0.013901
H	4.711729	-2.989043	-1.865166
C	5.653432	-1.438444	1.054696
H	4.774064	0.326298	1.921217
H	6.367078	-3.181182	-0.010891
H	6.395208	-1.551771	1.839104
C	2.745598	0.577184	-0.268456
C	2.414797	1.926586	0.221230
O	1.560531	2.647259	-0.243368
O	3.166319	2.254373	1.284552
C	2.891522	3.544398	1.833636
H	1.851251	3.603939	2.160474
H	3.568831	3.655109	2.678457

H	3.074749	4.319759	1.087285
N	2.207200	0.145899	-1.495007
N	2.813582	-0.930637	-1.902949
I	0.338449	-0.508321	0.171511
C	-0.953978	0.592786	-1.149317
C	-0.495392	1.389130	-2.176564
C	-2.285751	0.414337	-0.818449
C	-1.462336	2.052647	-2.931506
H	0.565841	1.483248	-2.370371
C	-3.230423	1.102058	-1.582751
C	-2.814592	1.910928	-2.635170
H	-1.144831	2.686114	-3.752907
H	-4.283878	0.998072	-1.352652
H	-3.557013	2.436388	-3.226394
C	-2.622202	-0.484836	0.365316
C	-3.691032	-1.526756	-0.023521
C	-3.100125	0.382756	1.546511
F	-3.322883	-2.156935	-1.138394
F	-3.853684	-2.430685	0.935411
F	-4.881750	-0.947280	-0.246054
F	-4.132256	1.165150	1.212492
F	-3.466341	-0.364752	2.584234
F	-2.092495	1.173557	1.935055
O	-1.526617	-1.222041	0.788692

PD

C	2.404463	-1.207581	-0.327837
C	3.728451	-0.842826	-0.015118
C	1.922787	-2.515851	-0.166675
C	4.623637	-1.819232	0.460047
C	2.819273	-3.452007	0.304970
H	0.893388	-2.775548	-0.396218
C	4.157147	-3.108506	0.611891
H	5.646580	-1.552641	0.698238
H	2.491670	-4.477030	0.446374
H	4.825766	-3.880908	0.977711
C	3.764368	0.563221	-0.294385
C	4.883694	1.521989	-0.160802
O	4.839842	2.698648	-0.409624
O	5.988859	0.894708	0.291523
C	7.124706	1.743082	0.450697
H	6.908084	2.537721	1.167857
H	7.925423	1.102153	0.816210
H	7.397827	2.195359	-0.505017
N	2.591674	0.995527	-0.725607
N	1.774233	-0.065940	-0.763867

I	-0.283548	0.180269	-1.284942
C	-0.726288	0.336212	0.807343
C	0.256283	0.443663	1.772134
C	-2.081313	0.310545	1.069642
C	-0.165639	0.518001	3.098861
H	1.308473	0.483886	1.515150
C	-2.479140	0.366475	2.407077
C	-1.521697	0.473809	3.410753
H	0.575288	0.612466	3.885587
H	-3.532846	0.329976	2.656162
H	-1.839868	0.525782	4.446553
C	-3.030123	0.186470	-0.127098
C	-4.169444	1.219130	-0.027086
C	-3.594298	-1.248408	-0.160048
F	-3.663459	2.435646	0.177979
F	-4.890908	1.246690	-1.141140
F	-5.003261	0.942093	0.991346
F	-4.185429	-1.592790	0.990666
F	-4.475717	-1.408840	-1.142475
F	-2.579895	-2.103372	-0.366598
O	-2.384486	0.435825	-1.313721

TS1-OMe-minor

C	4.106564	-0.616988	-1.712562
C	3.482310	-0.489352	-0.625524
C	5.124156	-1.532303	-1.956720
C	3.703529	-1.198646	0.543339
C	5.409849	-2.304890	-0.824560
H	5.663319	-1.654286	-2.888996
C	4.727147	-2.151499	0.396364
H	6.198041	-3.051926	-0.878810
H	5.007849	-2.782590	1.232893
C	1.840787	1.257222	-0.578959
C	2.304325	2.045627	0.575815
O	3.190727	2.864829	0.540914
O	1.600582	1.722424	1.666740
C	2.082884	2.301725	2.877962
H	2.081975	3.391108	2.807649
H	1.401578	1.963418	3.656567
H	3.099557	1.953946	3.075114
N	2.319481	1.608501	-1.745666
N	2.843304	1.658760	-2.742929
I	0.245034	-0.236537	-0.529862
C	-1.265395	1.287933	-0.354664
C	-0.965581	2.635538	-0.418066
C	-2.538046	0.795627	-0.142059

C	-2.010433	3.543478	-0.261492
H	0.046140	2.983828	-0.592795
C	-3.570361	1.722116	0.029849
C	-3.304964	3.085529	-0.034902
H	-1.804511	4.607390	-0.315844
H	-4.578598	1.371021	0.211874
H	-4.116171	3.794360	0.093648
C	-2.719067	-0.728542	-0.083427
C	-3.853951	-1.162851	-1.032606
C	-3.043639	-1.127371	1.370735
F	-3.627964	-0.680715	-2.257740
F	-3.934323	-2.486304	-1.114277
F	-5.056850	-0.709856	-0.631999
F	-4.119559	-0.489404	1.856898
F	-3.261194	-2.435439	1.483925
F	-2.000849	-0.809488	2.150188
O	-1.594427	-1.390227	-0.478565
O	2.980420	-0.956165	1.661113
C	3.248394	-1.767865	2.787956
H	2.558031	-1.442369	3.565565
H	3.072736	-2.825631	2.561189
H	4.280957	-1.635611	3.131364

3-(2-chlorophenyl)benzyne

C	-1.812267	-1.279028	0.421027
C	-0.919119	-0.285481	-0.051242
C	-1.663524	0.792557	-0.499240
C	-2.898148	0.940825	-0.533997
C	-3.840041	0.018880	-0.110138
C	-3.206941	-1.134415	0.389825
H	-1.397753	-2.186635	0.851756
H	-4.918450	0.127695	-0.132592
H	-3.819821	-1.943695	0.777594
C	0.546594	-0.451438	-0.055485
C	1.439962	0.624106	0.052883
C	1.102155	-1.733048	-0.180859
C	2.817795	0.429698	0.054370
C	2.474287	-1.939802	-0.179324
H	0.433047	-2.577718	-0.309978
C	3.337121	-0.853956	-0.058536
H	3.470648	1.290570	0.146466
H	2.868579	-2.945134	-0.283602
H	4.412225	-1.000448	-0.059092
Cl	0.863782	2.262469	0.206691

TS1-ClPh-major

C	3.222898	0.391828	-0.025481
C	2.301824	0.871093	0.679405
C	4.314068	-0.298422	0.508845
C	2.096074	0.830631	2.043586
C	4.210531	-0.424527	1.911247
C	3.148812	0.122118	2.648750
H	1.272458	1.269151	2.596853
H	4.973906	-0.978993	2.446754
H	3.140907	-0.008722	3.727259
C	0.324188	1.723562	-0.712358
C	0.277892	3.091555	-0.187578
O	0.802107	4.051309	-0.700944
O	-0.434061	3.129629	0.950360
C	-0.528834	4.426039	1.545255
H	-0.997984	5.126684	0.851809
H	-1.141083	4.295535	2.435515
H	0.465131	4.794046	1.807000
N	1.093796	1.537843	-1.752051
N	1.846754	1.263634	-2.542747
I	-0.815375	0.011651	0.016163
C	-2.600643	0.730857	-0.953187
C	-2.601355	1.853784	-1.758732
C	-3.729698	-0.010222	-0.666455
C	-3.815014	2.253123	-2.314538
H	-1.691336	2.407424	-1.961981
C	-4.939843	0.415389	-1.221564
C	-4.976859	1.536723	-2.043160
H	-3.844279	3.126503	-2.957655
H	-5.848088	-0.135027	-1.008686
H	-5.921548	1.853129	-2.472591
C	-3.578558	-1.225647	0.260675
C	-4.219736	-2.467996	-0.389548
C	-4.260939	-0.902006	1.605760
F	-3.732787	-2.635411	-1.622461
F	-3.963457	-3.566010	0.311980
F	-5.557321	-2.356261	-0.491546
F	-5.546356	-0.544570	1.463657
F	-4.215905	-1.937771	2.439787
F	-3.618556	0.124748	2.179923
O	-2.269803	-1.528031	0.494888
C	5.380972	-0.901288	-0.325529
C	6.738095	-0.974849	0.014185
C	5.006447	-1.429269	-1.569985
C	7.668502	-1.574543	-0.831058
C	5.921895	-2.030581	-2.420353

H	3.960515	-1.354883	-1.853675
C	7.260618	-2.111053	-2.045267
H	8.709919	-1.603209	-0.530253
H	5.592431	-2.434620	-3.371827
H	7.990409	-2.578920	-2.697948
Cl	7.362653	-0.257031	1.481904

TS1-ClPh-minor

C	3.760581	0.409161	-2.240551
C	3.222465	-0.022295	-1.180495
C	4.481631	-0.374748	-3.130615
C	3.217744	-1.328051	-0.702058
C	4.529872	-1.723121	-2.746628
H	4.961743	-0.022642	-4.037737
C	3.915644	-2.185856	-1.572576
H	5.059180	-2.435094	-3.374021
H	3.990928	-3.238206	-1.309708
C	1.576012	1.658666	-0.443266
C	1.823909	2.197330	0.909239
O	2.501931	3.165214	1.154992
O	1.145259	1.485372	1.817388
C	1.298884	1.927896	3.165556
H	0.948703	2.957909	3.264262
H	0.687484	1.253597	3.763174
H	2.348361	1.870788	3.461634
N	2.043644	2.377650	-1.435238
N	2.608434	2.681374	-2.366860
I	0.000018	0.199533	-0.875355
C	-1.534149	1.537316	-0.179764
C	-1.286587	2.864647	0.117548
C	-2.766250	0.933198	-0.018051
C	-2.341991	3.628545	0.611500
H	-0.310121	3.311679	-0.035229
C	-3.806166	1.709752	0.499342
C	-3.592446	3.049218	0.806462
H	-2.178592	4.676071	0.842270
H	-4.780854	1.263316	0.655266
H	-4.410147	3.644408	1.199109
C	-2.886897	-0.551523	-0.394824
C	-4.140463	-0.784377	-1.261939
C	-2.968447	-1.379626	0.904935
F	-4.148221	0.066607	-2.290619
F	-4.167298	-2.022158	-1.743639
F	-5.280552	-0.597409	-0.570004
F	-3.973703	-0.993273	1.704262
F	-3.123261	-2.678554	0.652022

F	-1.826903	-1.226762	1.592295
O	-1.815933	-0.972190	-1.127034
C	2.515501	-1.803608	0.512385
C	2.717229	-1.229441	1.770973
C	1.544570	-2.807765	0.401712
C	1.957947	-1.611150	2.872629
C	0.771605	-3.190739	1.490189
H	1.372308	-3.253170	-0.574827
C	0.974080	-2.582687	2.726967
H	2.147950	-1.146787	3.834795
H	-0.001887	-3.941572	1.368433
H	0.368210	-2.864958	3.581762
Cl	3.962057	-0.027732	1.999745

8. References

1. P. Caramenti, R. K. Nandi, J. Waser, Metal-Free Oxidative Cross Coupling of Indoles with Electron-Rich (Hetero)arenes. *Chem. Eur. J.* **2018**, *24*, 10049-10053.
2. S. Yoshida, K. Shinmori, T. Nonaka, T. Hosoya, Facile Synthesis of Diverse Multisubstituted *ortho*-Silylaryl Triflates via C–H Borylation. *Chem. Lett.* **2015**, *44*, 1324-1326.
3. B. Michel, M. F. Greaney, Continuous-Flow Synthesis of Trimethylsilylphenyl Perfluorosulfonate Benzyne Precursors. *Org. Lett.* **2014**, *16*, 2684-2687.
4. M. Lanzi, T. Rogge, T. S. Truong, K. N. Houk, J. Wencel-Delord, Cyclic Diaryl λ^3-Chloranes: Reagents and Their C–C and C–O Couplings with Phenols via Aryne Intermediates. *J. Am. Chem. Soc.* **2023**, *145*, 345-358.
5. M. Lanzi, Q. Dherbassy, J. Wencel-Delord, Cyclic Diaryl λ^3-Bromanes as Original Aryne Precursors. *Angew. Chem. Int. Ed.* **2021**, *60*, 14852-14857.
6. D. C. Martos, M. De Abreu, P. Hauk, P. Fackler and J. Wencel-Delord, Easy Access to Polyhalogenated Biaryls: Regioselective (Di)halogenation of Hypervalent Bromines and Chlorines. *Chem. Sci.* **2024**, *15*, 6770–6776
7. W. Ding, C. Wang, J. R. Tan, C. C. Ho, F. León, F. García, N. Yoshikai, Site-Selective Aromatic C–H λ^3-Iodanation with a Cyclic Iodine(III) Electrophile in Solution and Solid Phases. *Chem. Sci.* **2020**, *11*, 7356–7361.
8. S. Abe, J. Kikuchi, A. Matsumoto, N. Yoshikai, Stable and Responsive Atropisomerism around a Carbon–Iodine Bond. *ChemRxiv* **2024**, DOI: 10.26434/chemrxiv-2024-t16qt.
9. O. V. Dolomanov, L. J. Bourhis, R. J. Gildea, J. A. K. Howard, H. Puschmann, OLEX2: A Complete Structure Solution, Refinement and Analysis Program. *J. Appl. Cryst.* **2009**, *42*, 339-341.
10. L. J. Bourhis, O. V. Dolomanov, R. J. Gildea, J. A. K. Howard, H. Puschmann, The Anatomy of a Comprehensive Constrained, Restrained Refinement. *Acta Cryst.* **2015**, *A71*, 59-75.
11. G. M. Sheldrick, Crystal Structure Refinement with SHELXL. *Acta Cryst.* **2015**, *C71*, 3-8.
12. M. J. Frisch, G. W. Trucks, H. B. Schlegel, G. E. Scuseria, M. A. Robb, J. R. Cheeseman, G. Scalmani, V. Barone, G. A. Petersson, H. Nakatsuji, X. Li, M. Caricato, A. V. Marenich, J. Bloino, B. G. Janesko, R. Gomperts, B. Mennucci, H. P. Hratchian, J. V. Ortiz, A. F. Izmaylov, J. L. Sonnenberg, D. Williams-Young, F. Ding, F. Lipparini, F. Egidi, J. Goings, B. Peng, A. Petrone, T. Henderson, D. Ranasinghe, V. G. Zakrzewski, J. Gao, N. Rega, G. Zheng, W. Liang, M. Hada, M. Ehara, K. Toyota, R. Fukuda, J. Hasegawa, M. Ishida, T. Nakajima, Y. Honda, O. Kitao, H. Nakai, T. Vreven, K. Throssell, J. A. Montgomery, Jr., J. E. Peralta, F. Ogliaro, M. J. Bearpark, J. J. Heyd, E. N. Brothers, K. N. Kudin, V. N. Staroverov, T. A. Keith, R. Kobayashi, J. Normand, K. Raghavachari, A. P. Rendell, J. C. Burant, S. S. Iyengar, J. Tomasi, M. Cossi, J. M. Millam, M. Klene, C. Adamo, R. Cammi, J. W. Ochterski, R. L. Martin, K. Morokuma, O. Farkas, J. B. Foresman, Gaussian 16, Revision A.03; Gaussian, Inc.: Wallingford, CT (2016).

13. Y. Zhao, D. G. Truhlar, Density Functional for Spectroscopy: No Long-Range Self-Interaction Error, Good Performance for Rydberg and Charge-Transfer States, and Better Performance on Average than B3LYP for Ground States. *J. Phys. Chem. A* **2006**, *110*, 13126–13130.
14. Y. Zhao, D. G. Truhlar, Density Functionals with Broad Applicability in Chemistry. *Acc. Chem. Res.* **2008**, *41*, 157–167.
15. Y. Zhao, D. G. Truhlar, Applications and Validations of the Minnesota Density Functionals. *Chem. Phys. Lett*. **2011**, *502*, 1–13.
16. M. Dolg, U. Wedig, H. Stoll, H. Preuss, Energy-Adjusted ab Initio Pseudopotentials for the First Row Transition Elements. *J. Chem. Phys.* **1987**, *86*, 866–872.
17. K. Fukui, The Path of Chemical Reactions – The IRC Approach. *Acc. Chem. Res*. **1981**, *14*, 363–368.
18. A. V. Marenich, C. J. Cramer, D. G. Truhlar, Universal Solvation Model Based on Solute Electron Density and on a Continuum Model of the Solvent Defined by the Bulk Dielectric Constant and Atomic Surface Tensions. *J. Phys. Chem. B* **2009**, *113*, 6378–6396.
19. C. Y. Legault, *CYLview20*; Université de Sherbrooke, 2020 (http://www.cylview.org).

9. ^{1}H and ^{13}C NMR Spectra

^{1}H NMR spectrum of **2a** (400 MHz, $CDCl_3$)

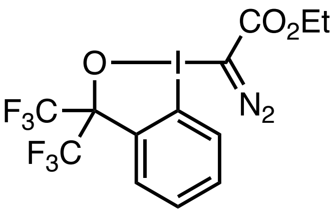

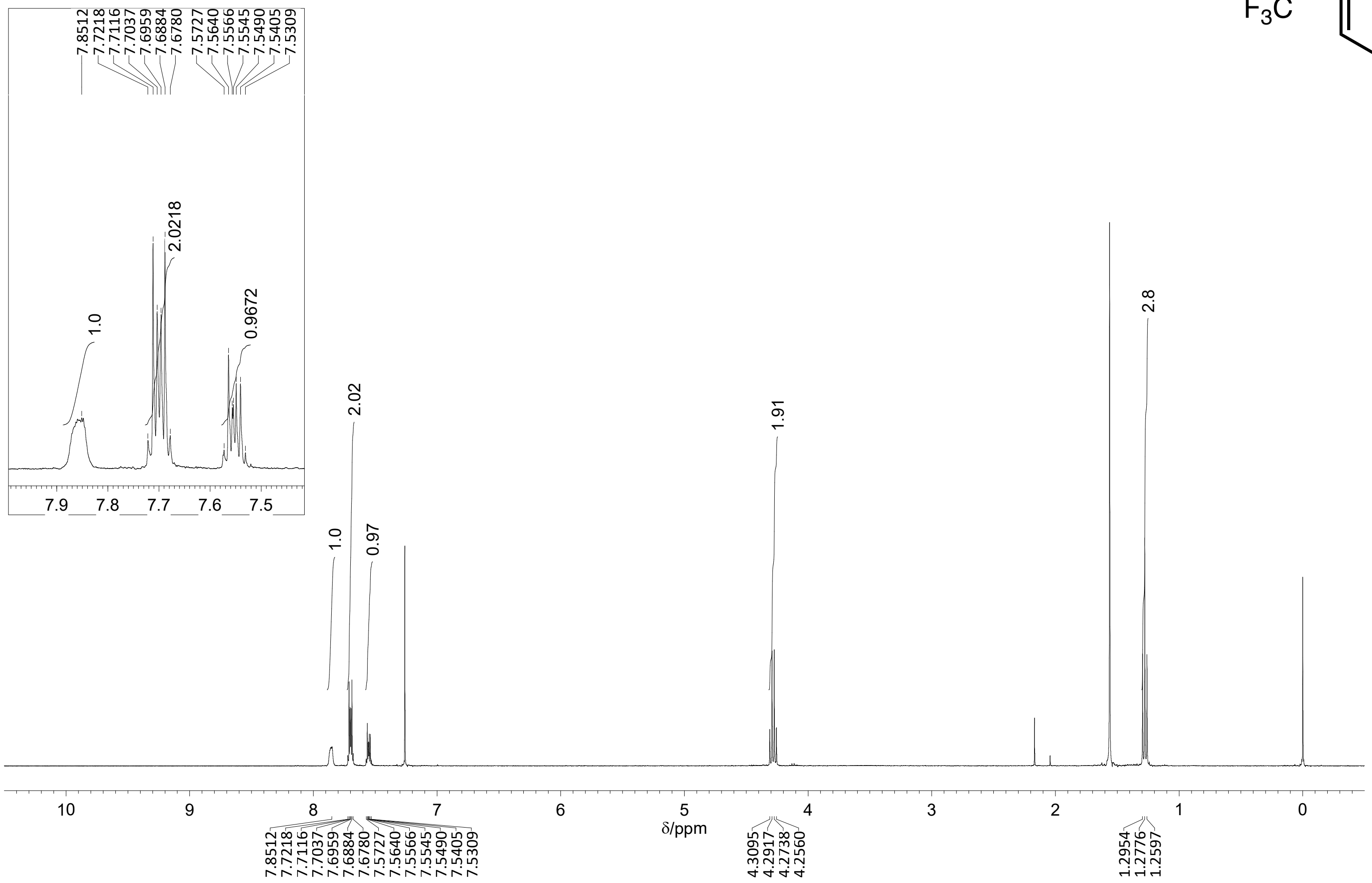

$^{13}C\{^{1}H\}$ NMR spectrum of **2a** (101 MHz, $CDCl_3$)

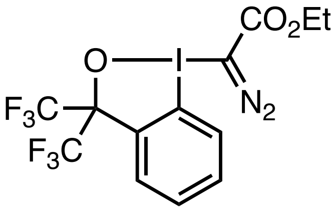

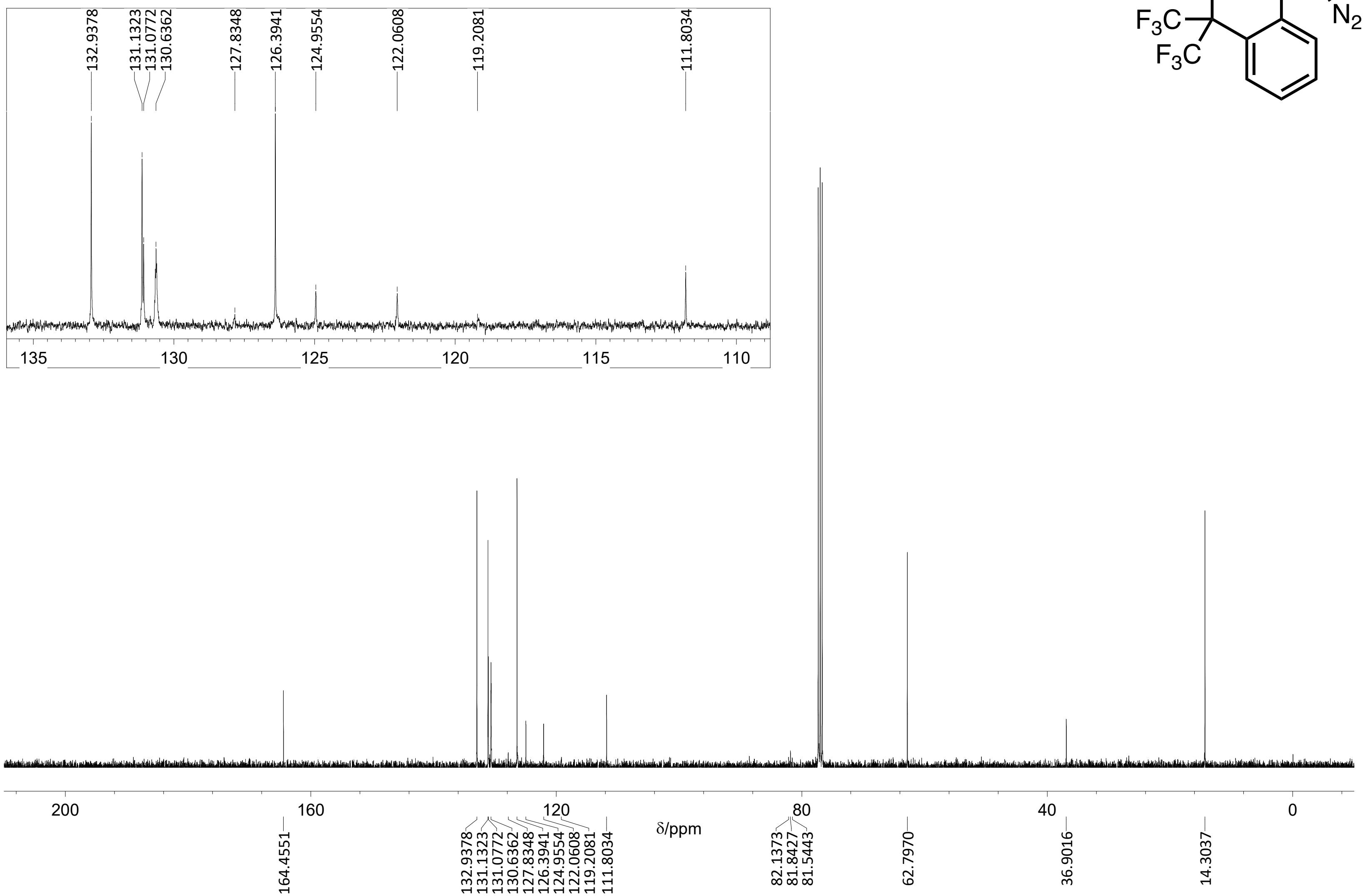

^{1}H NMR spectrum of **2b** (400 MHz, $CDCl_3$)

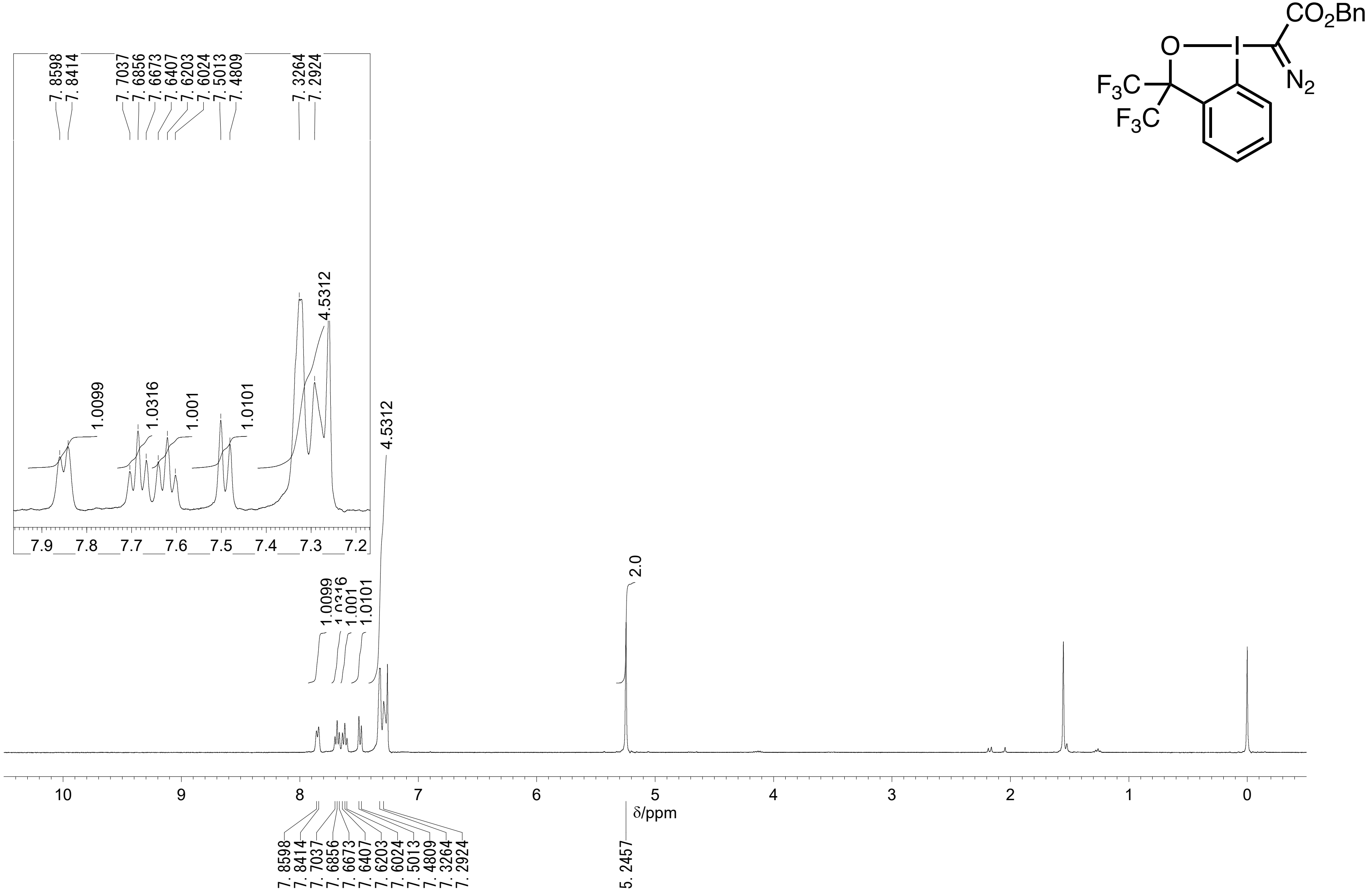

$^{13}C\{^{1}H\}$ NMR spectrum of **2b** (150 MHz, $CDCl_3$)

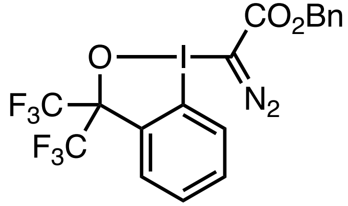

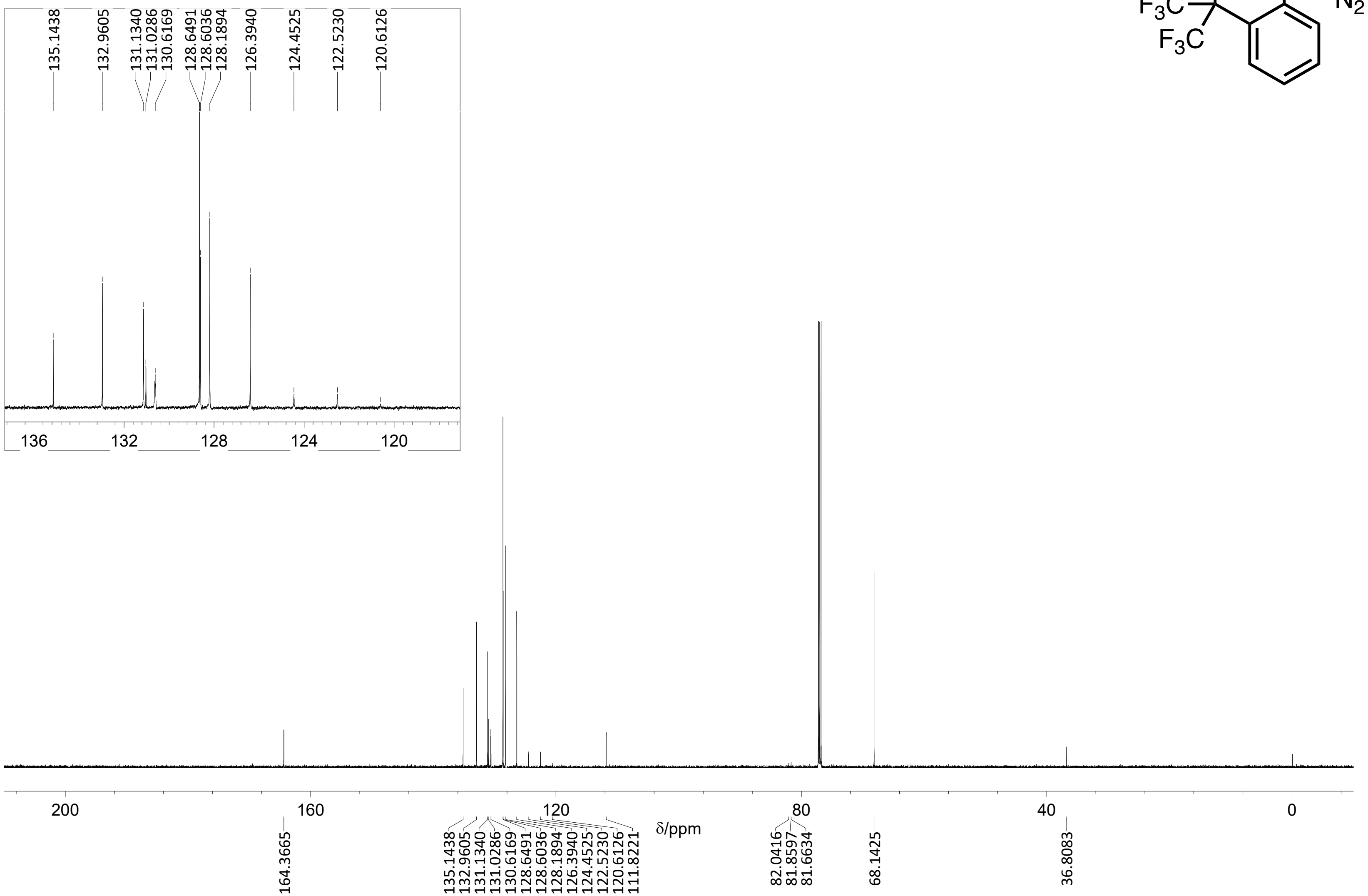

^{1}H NMR spectrum of **2c** (400 MHz, $CDCl_3$)

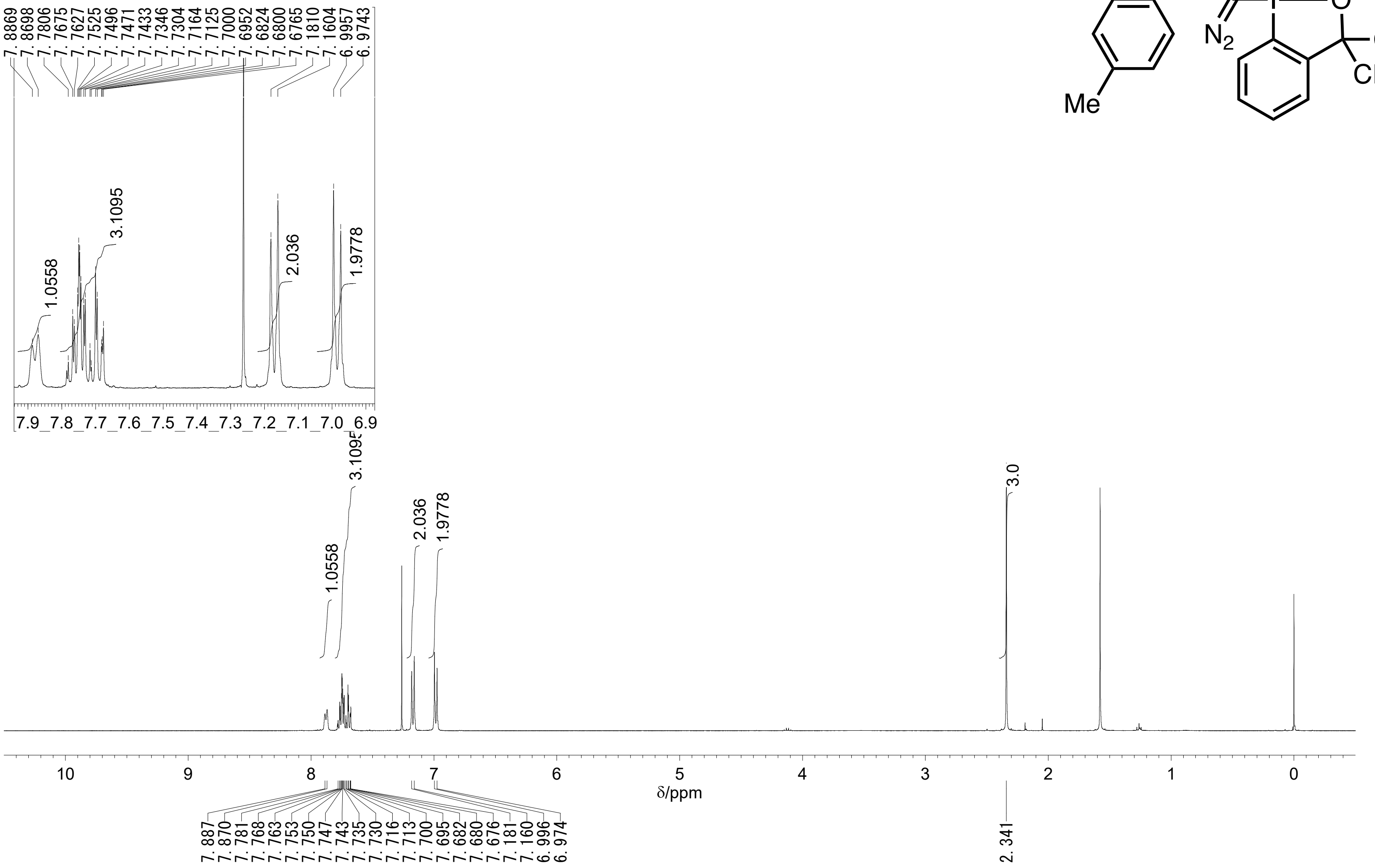

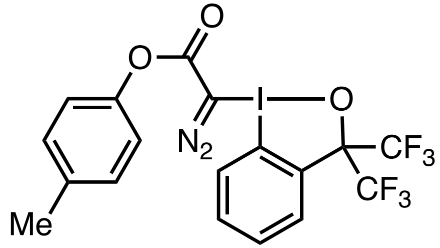

$^{13}C\{^1H\}$ NMR spectrum of **2c** (150 MHz, $CDCl_3$)

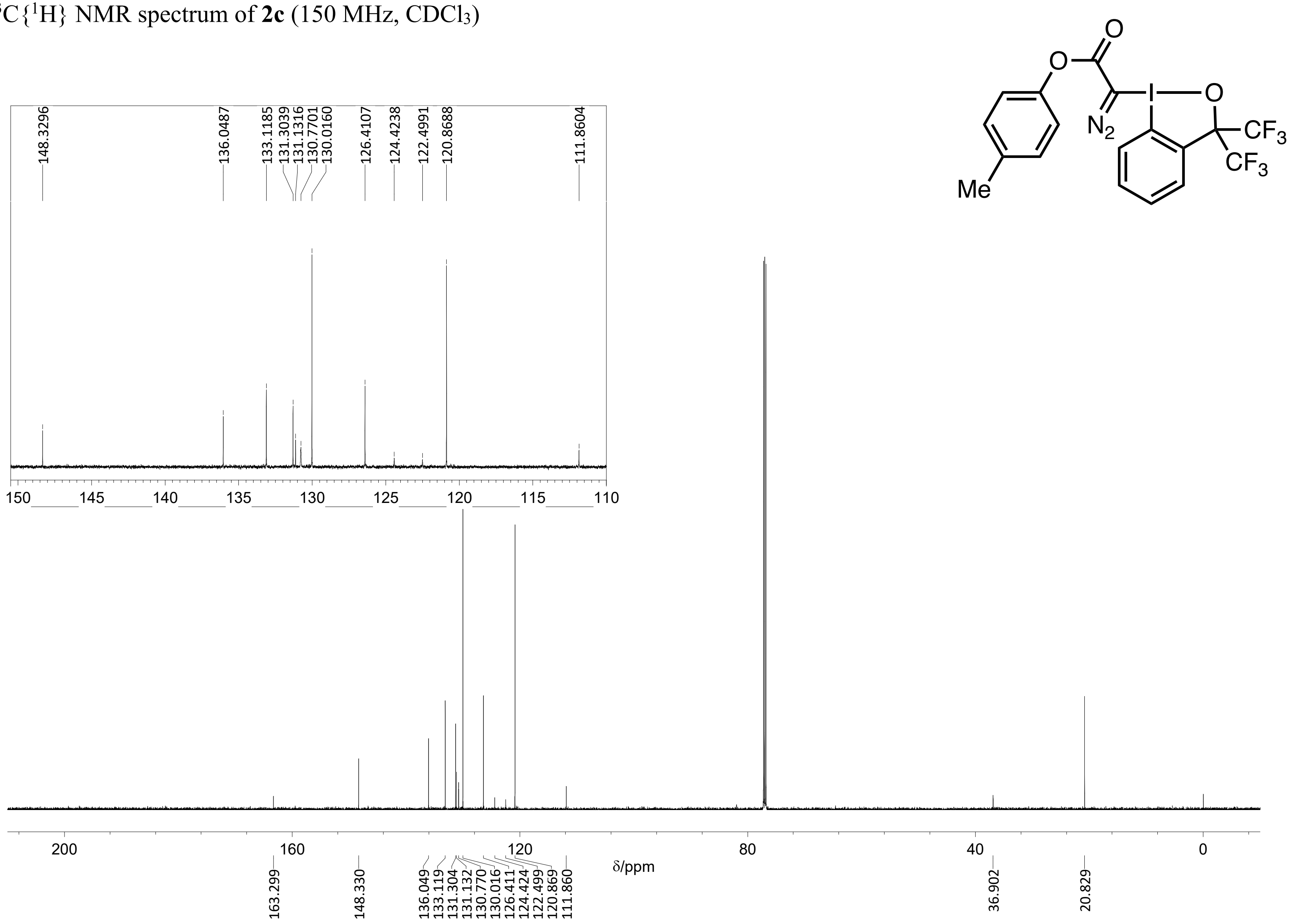

^{1}H NMR spectrum of **2d** (400 MHz, $CDCl_3$)

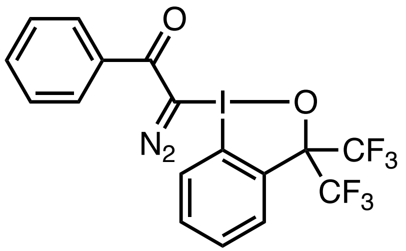

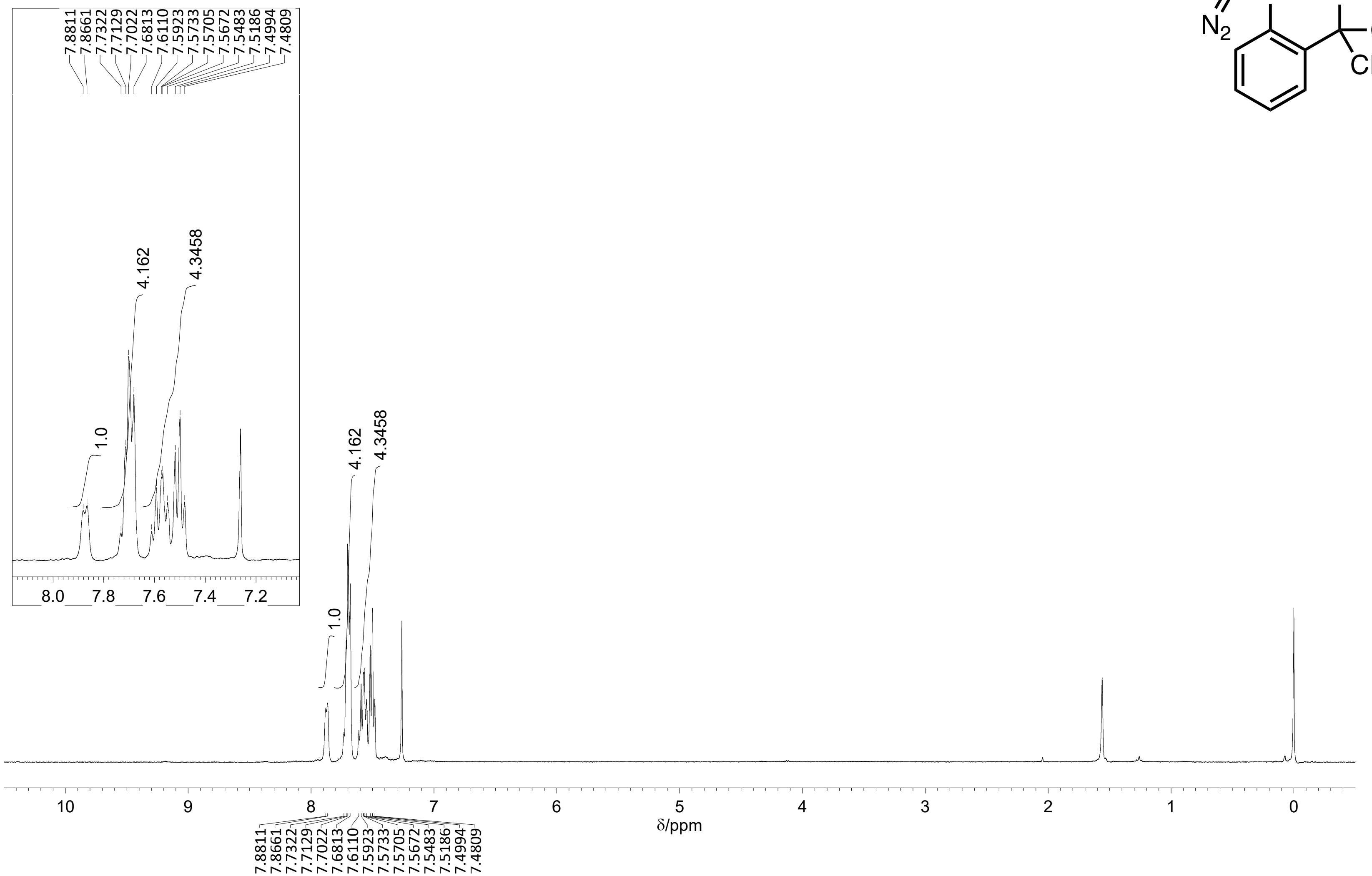

$^{13}C\{^{1}H\}$ NMR spectrum of **2d** (150 MHz, CDCl3)

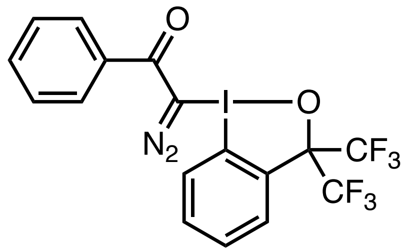

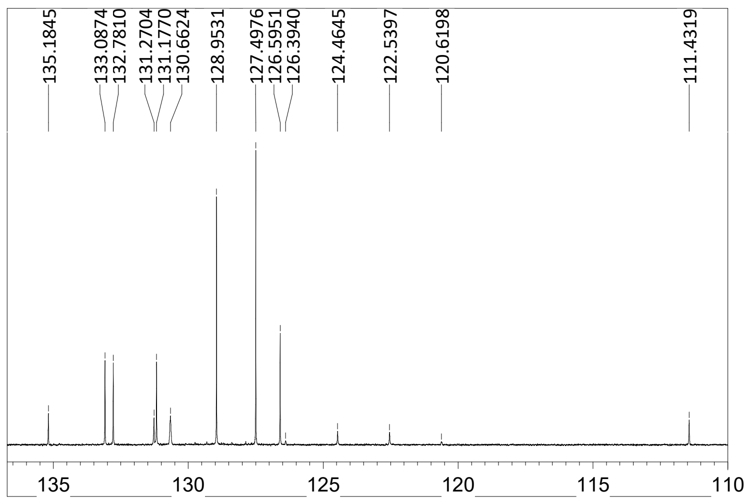

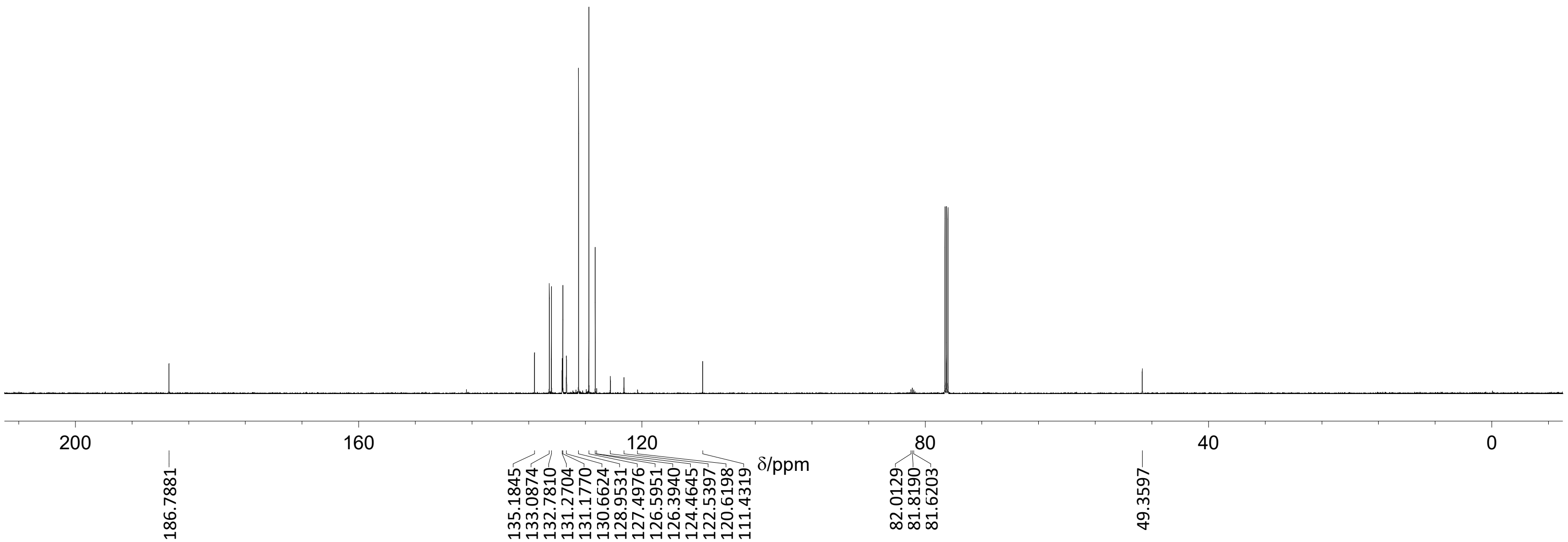

^1H NMR spectrum of **2e** (400 MHz, $CDCl_3$)

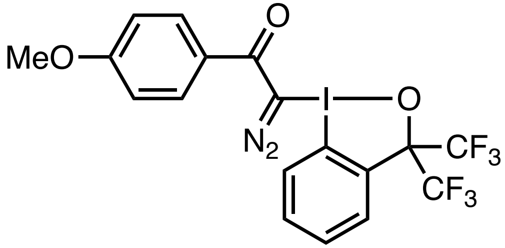

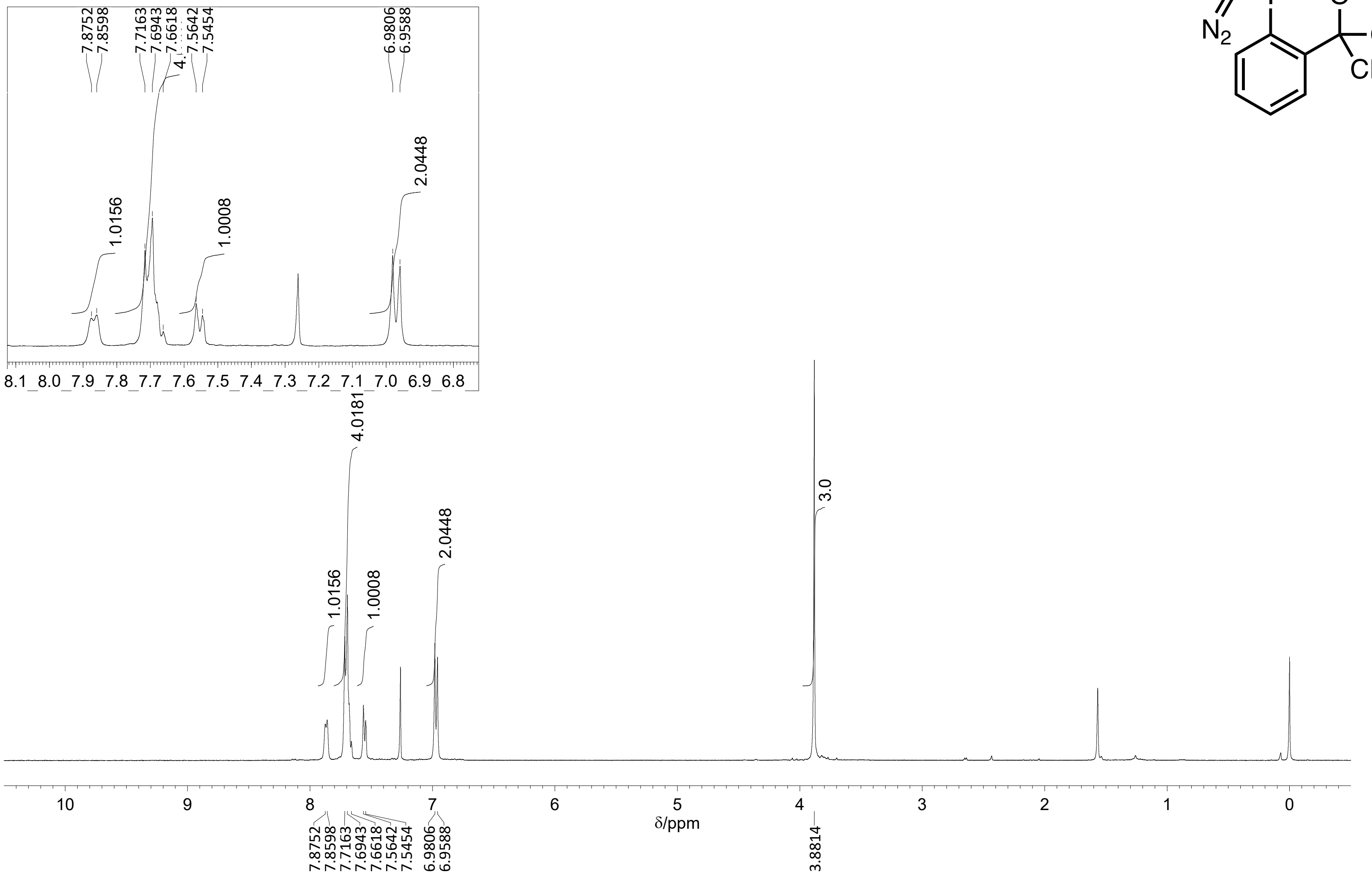

$^{13}C\{^1H\}$ NMR spectrum of **2e** (150 MHz, $CDCl_3$)

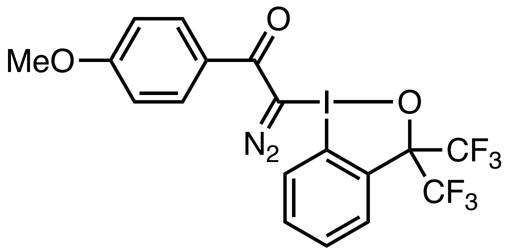

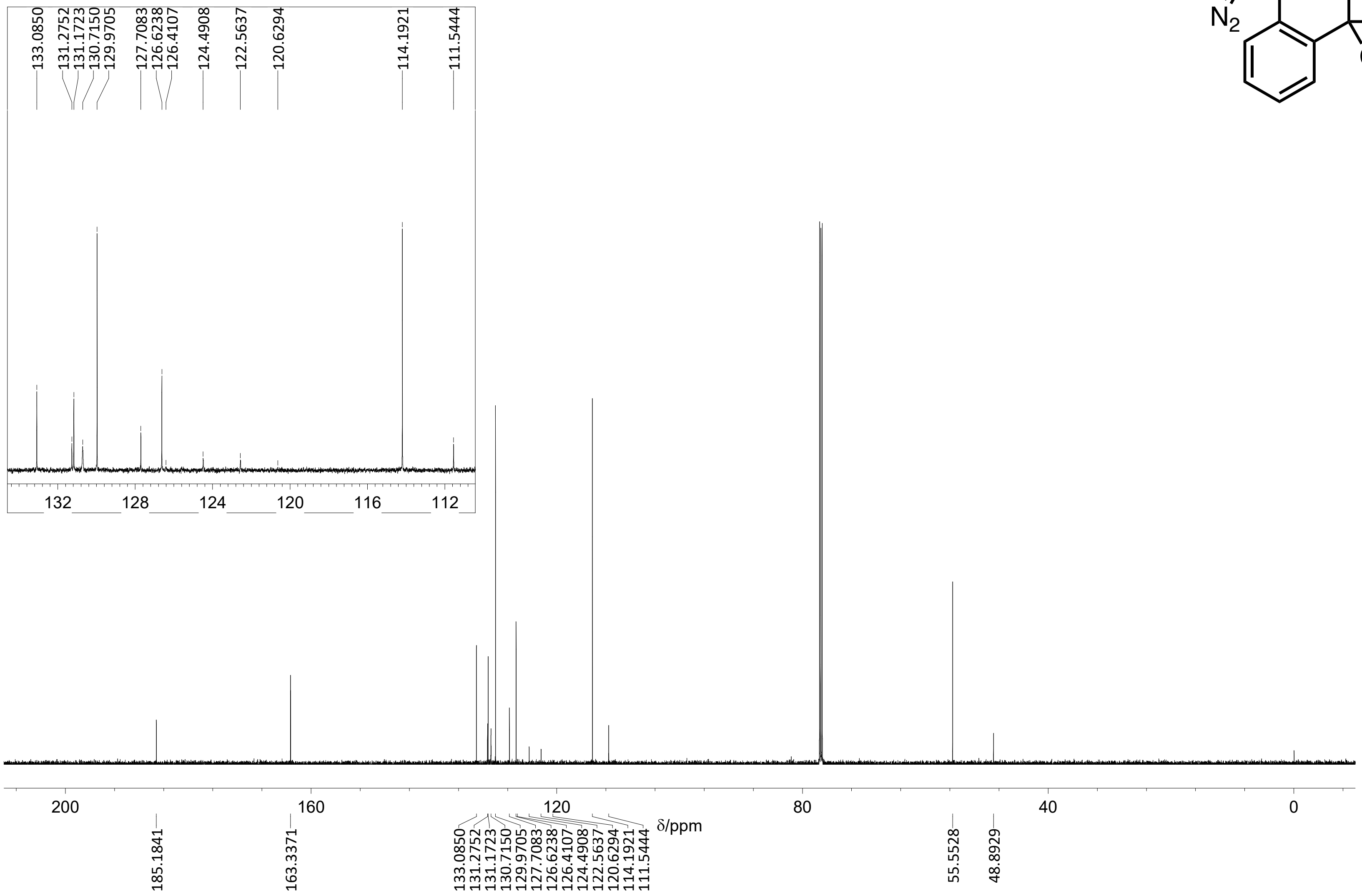

^{1}H NMR spectrum of **2f** (400 MHz, $CDCl_3$)

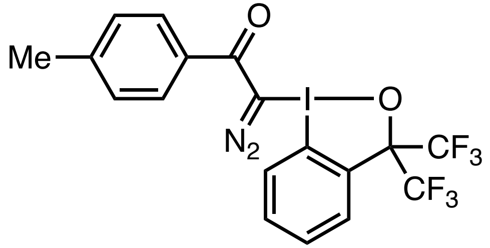

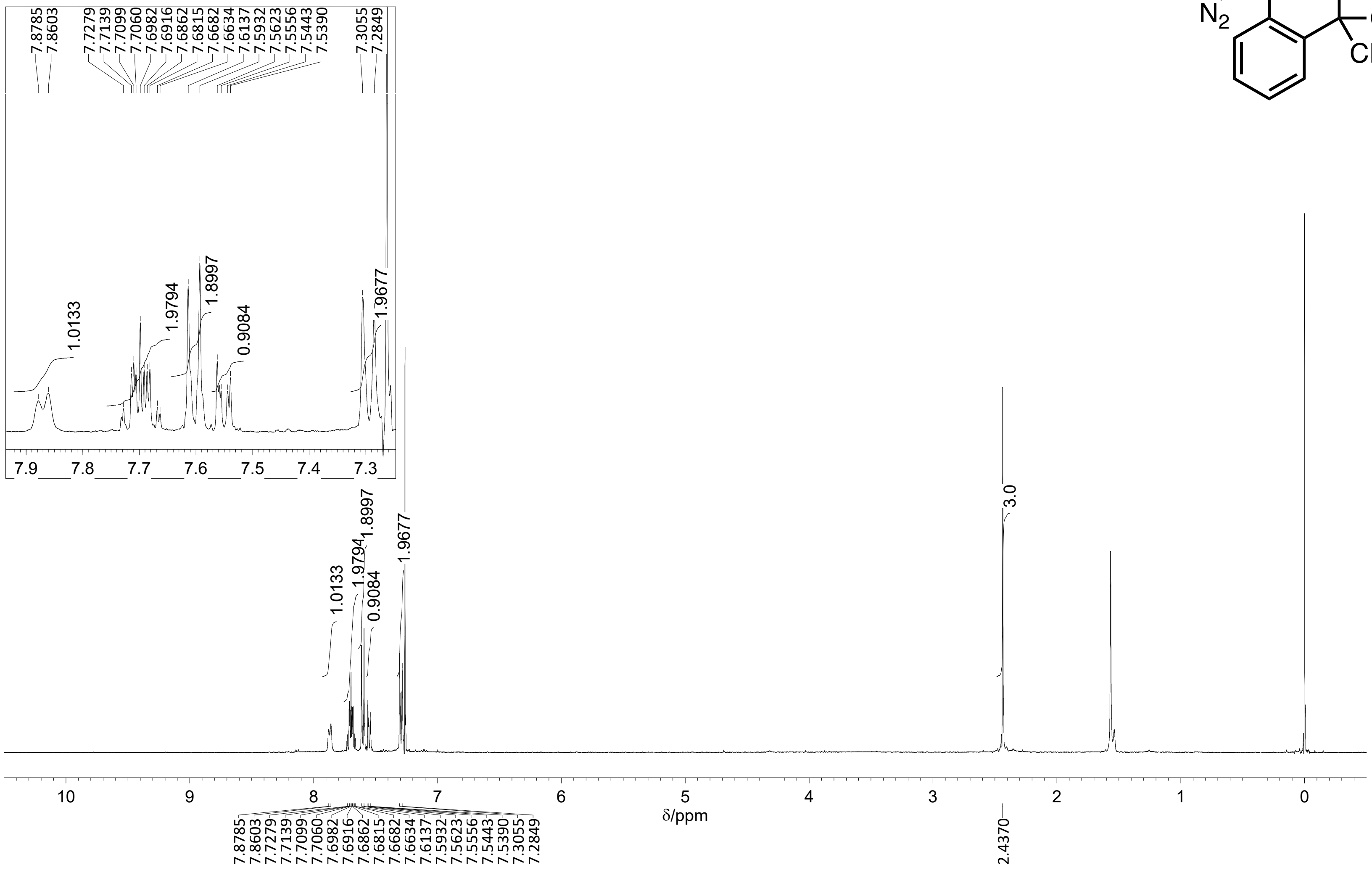

$^{13}C\{^{1}H\}$ NMR spectrum of **2f** (150 MHz, $CDCl_3$)

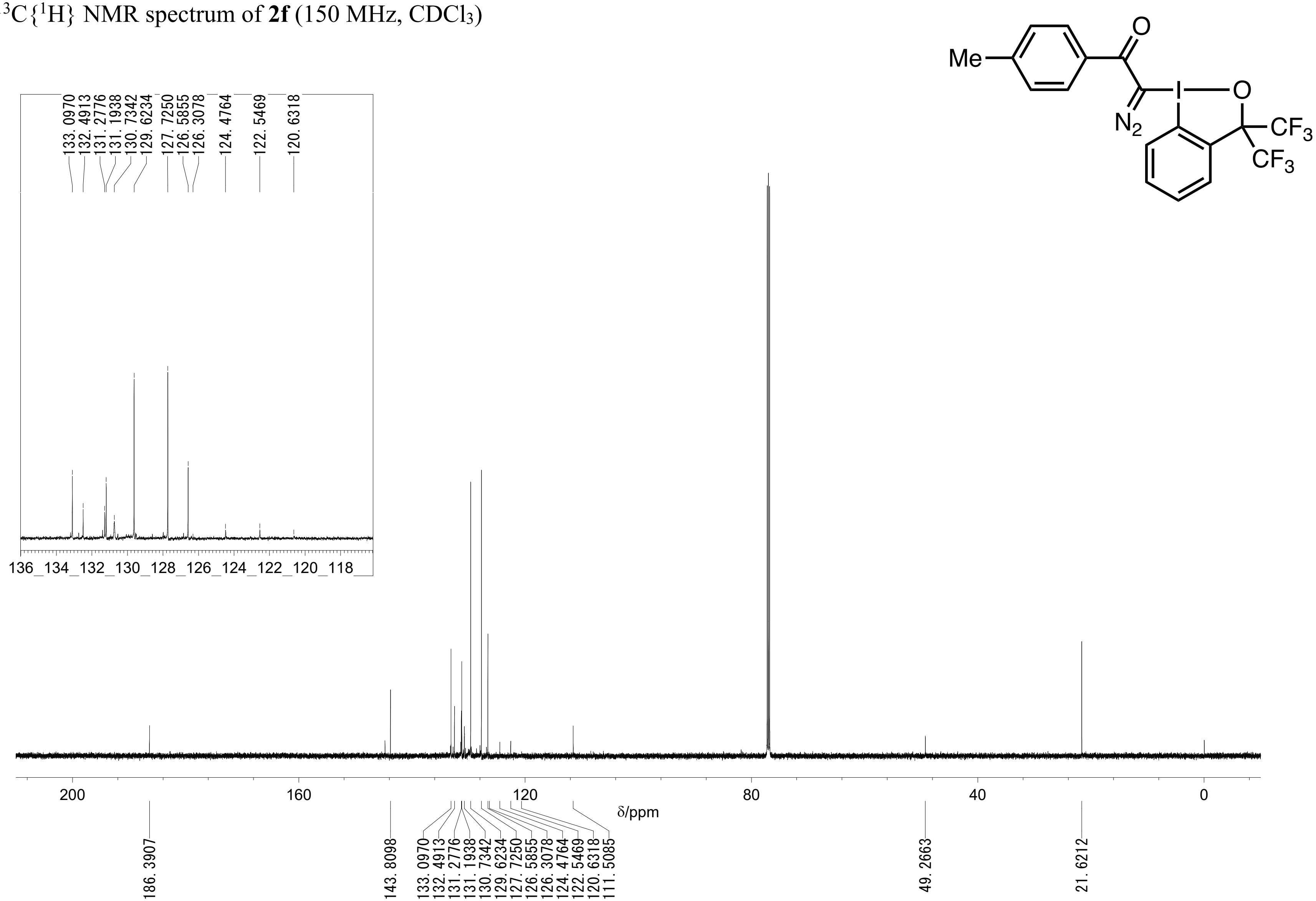

^{1}H NMR spectrum of **2g** (400 MHz, $CDCl_3$)

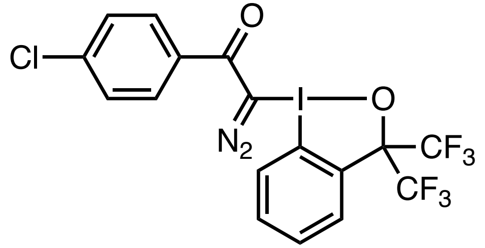

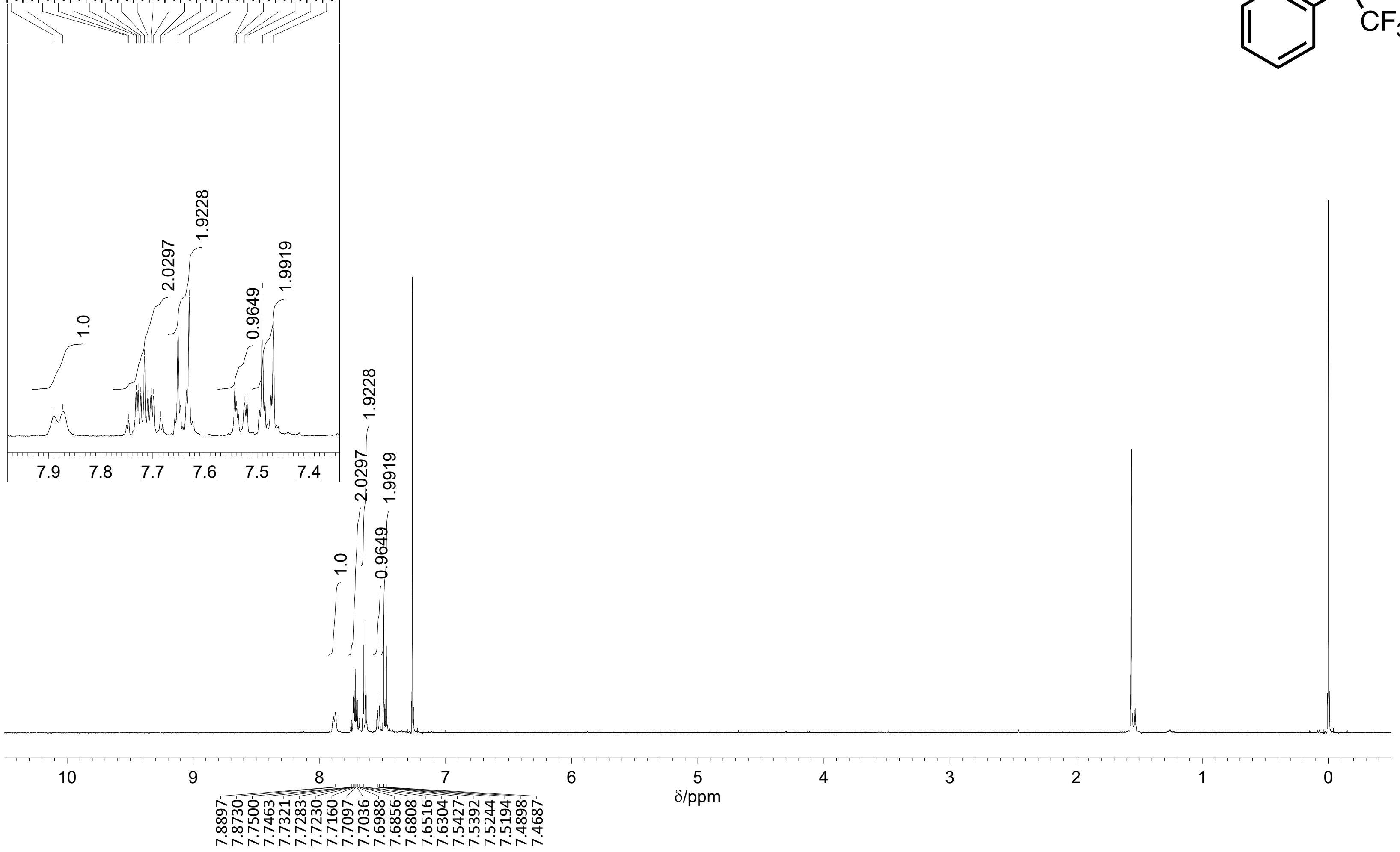

$^{13}C\{^{1}H\}$ NMR spectrum of **2g** (150 MHz, $CDCl_3$)

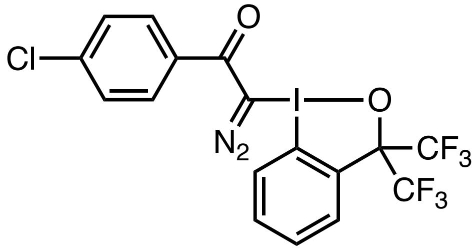

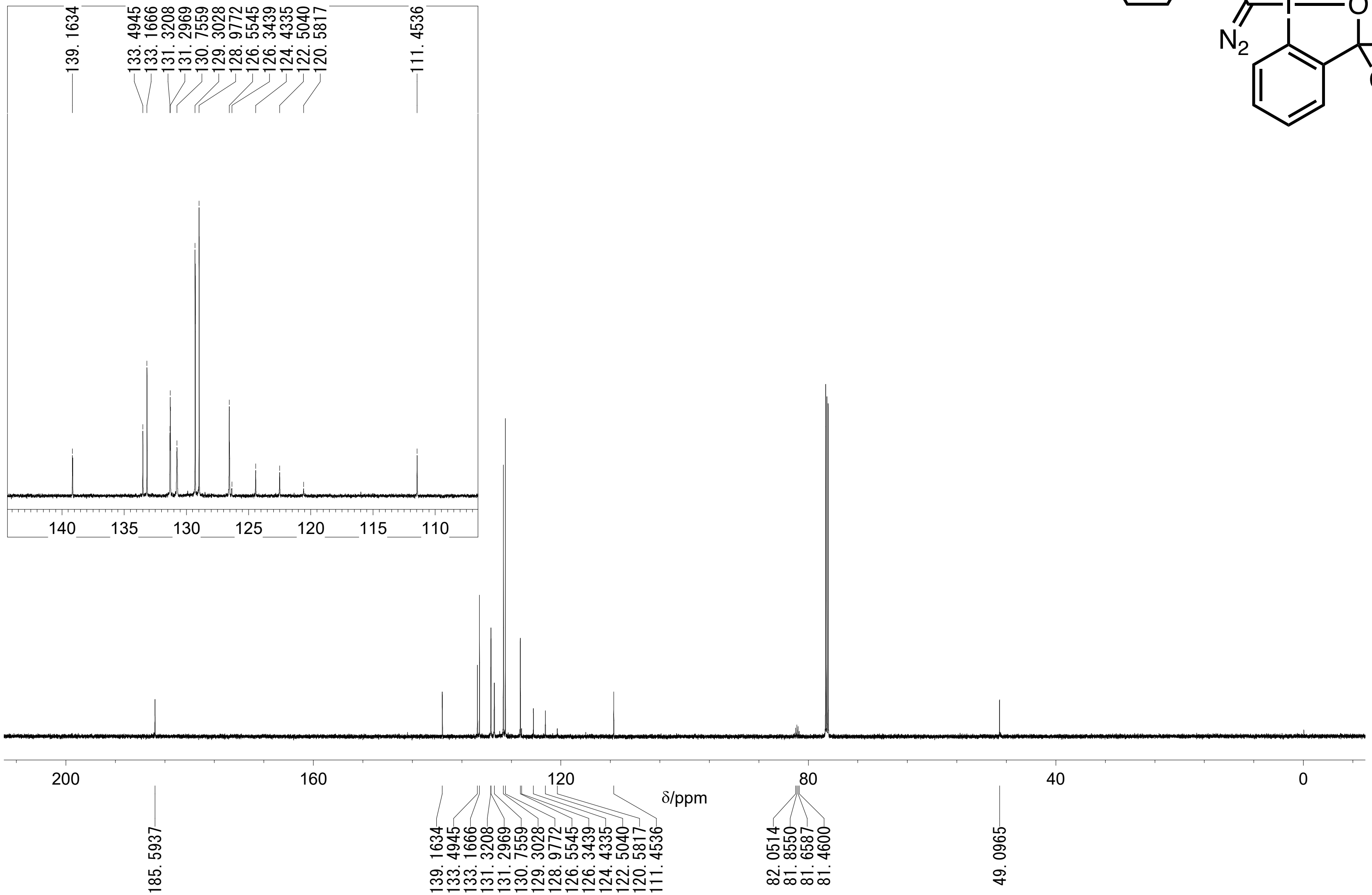

^{1}H NMR spectrum of **2h** (400 MHz, $CDCl_3$)

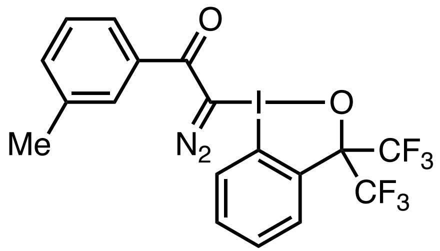

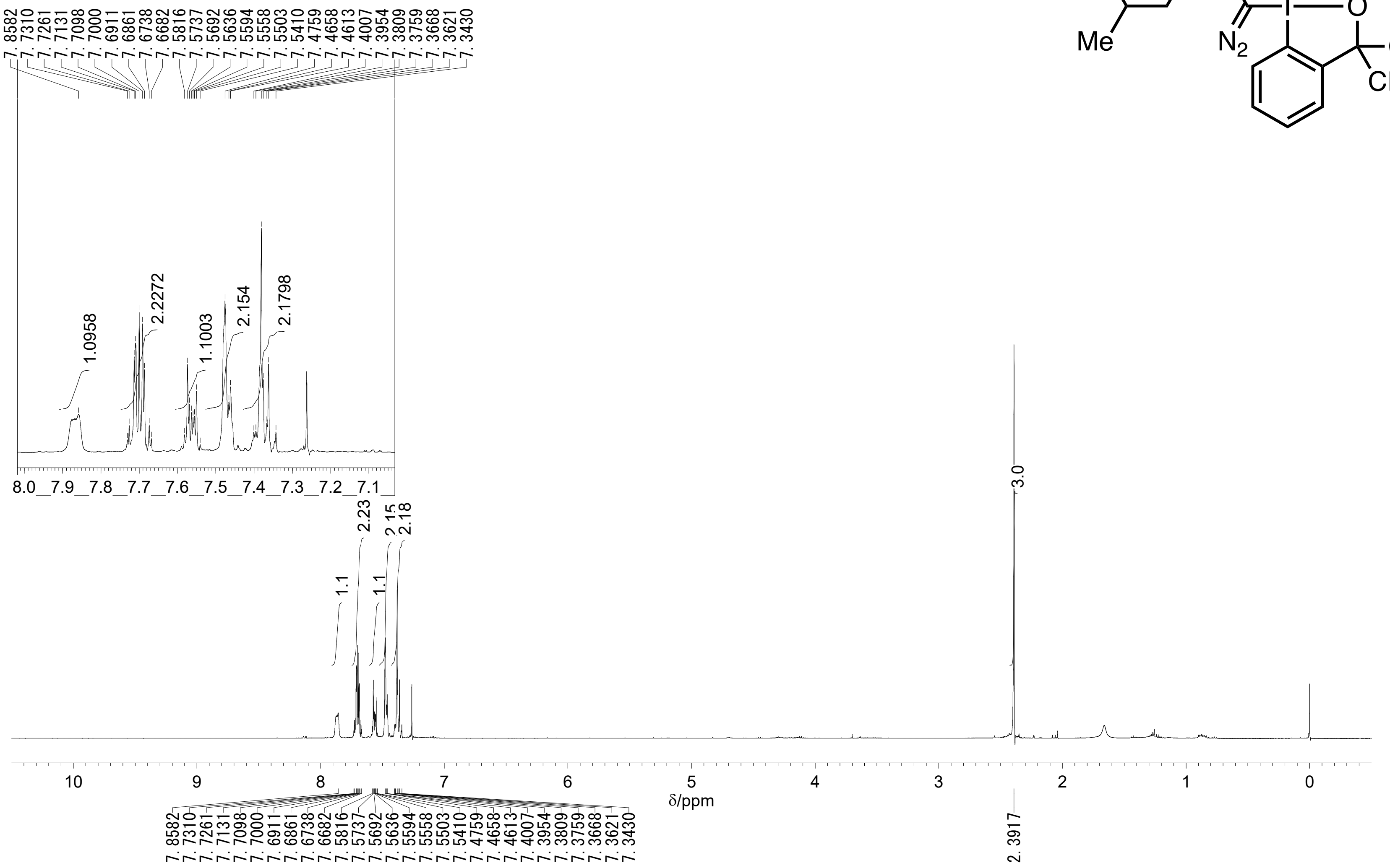

$^{13}C\{^{1}H\}$ NMR spectrum of **2h** (150 MHz, $CDCl_3$)

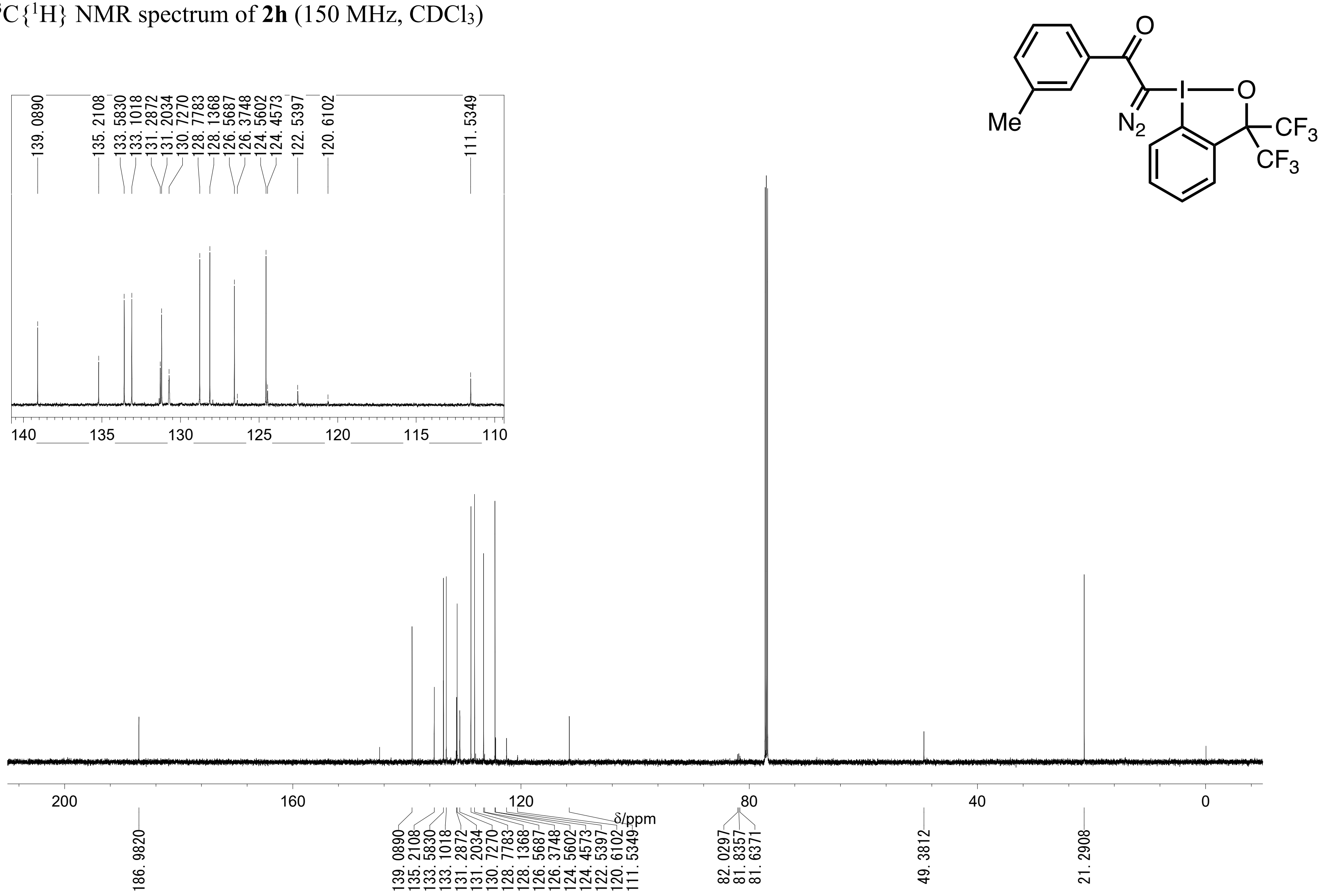

^{1}H NMR spectrum of **2i** (400 MHz, $CDCl_3$)

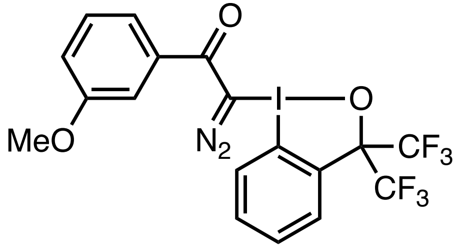

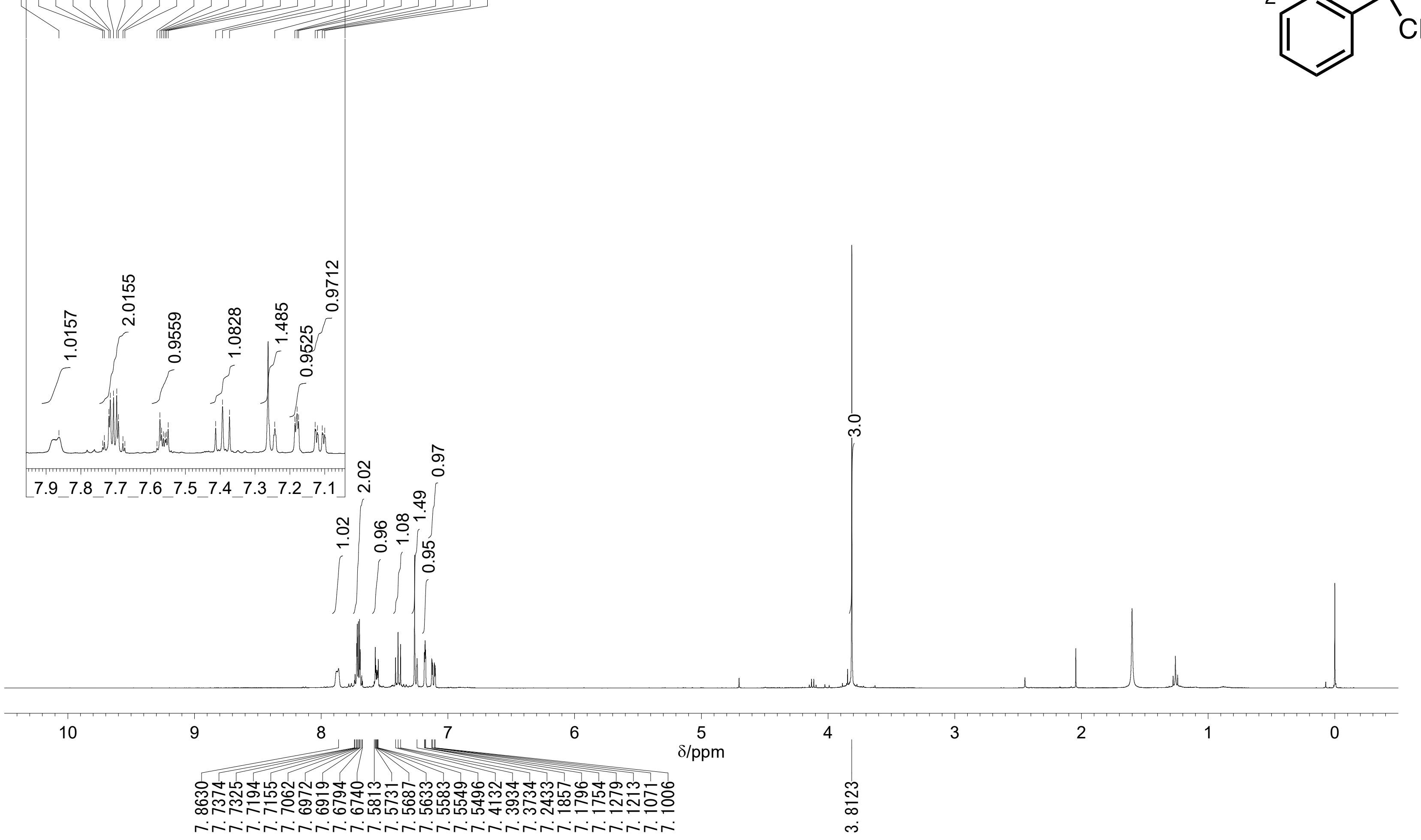

$^{13}C\{^1H\}$ NMR spectrum of **2i** (150 MHz, $CDCl_3$)

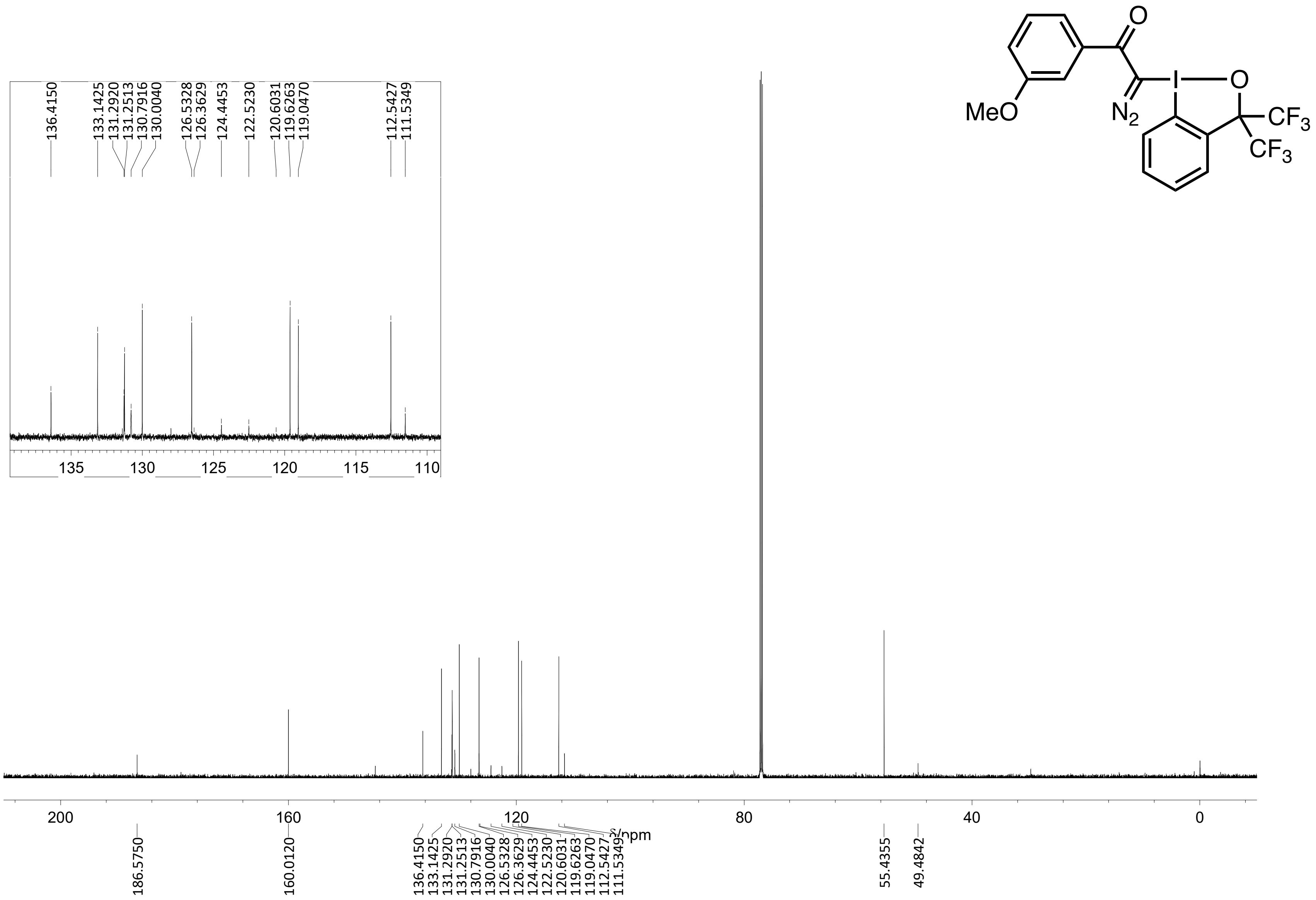

^{1}H NMR spectrum of **2j** (400 MHz, $CDCl_3$)

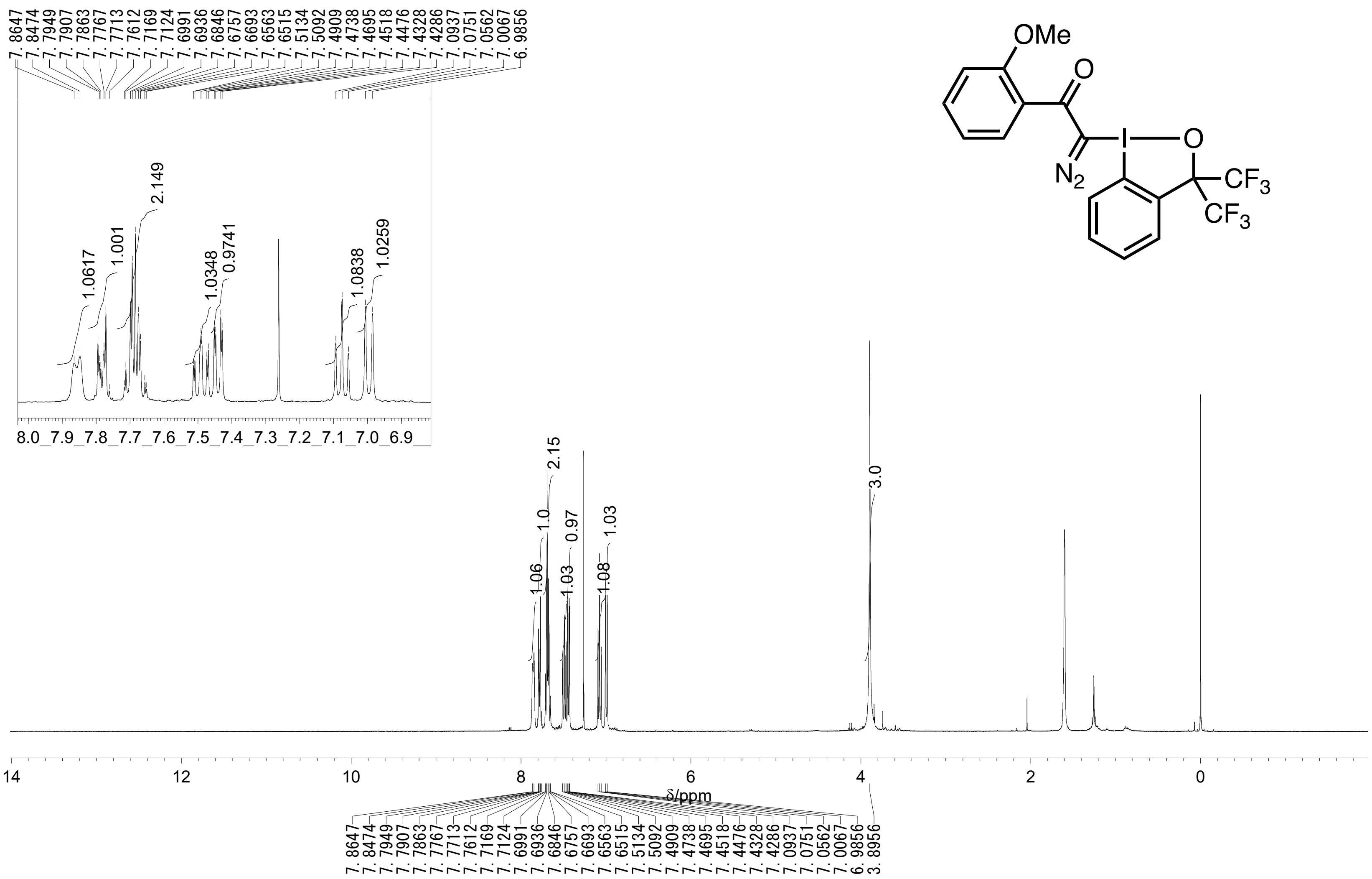

$^{13}C\{^1H\}$ NMR spectrum of **2j** (150 MHz, $CDCl_3$)

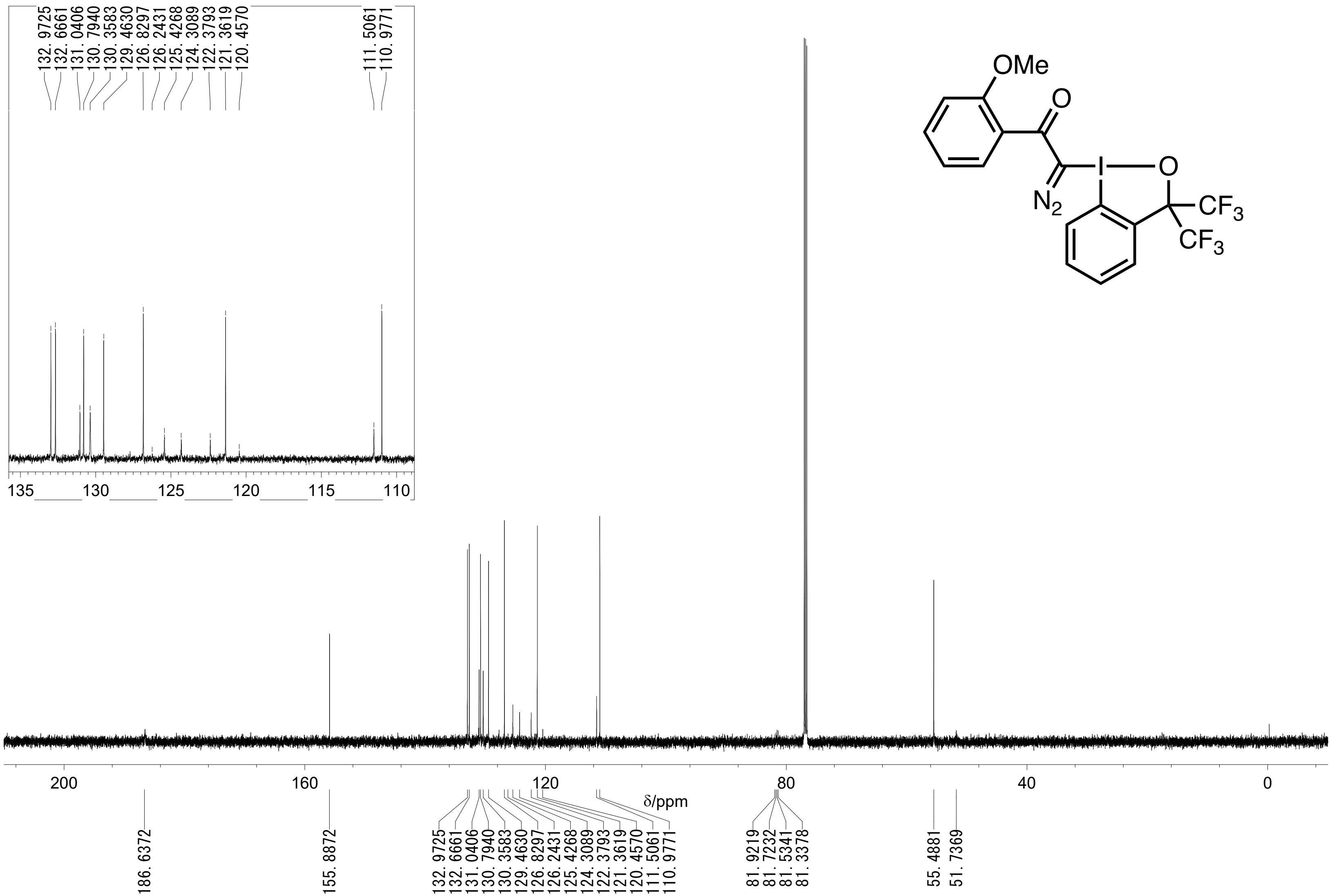

^{1}H NMR spectrum of **2k** (400 MHz, $CDCl_3$)

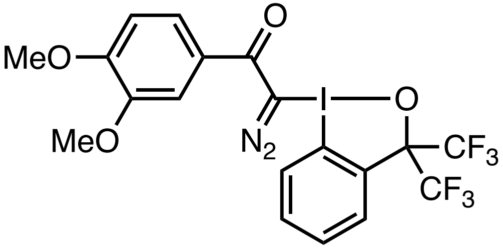

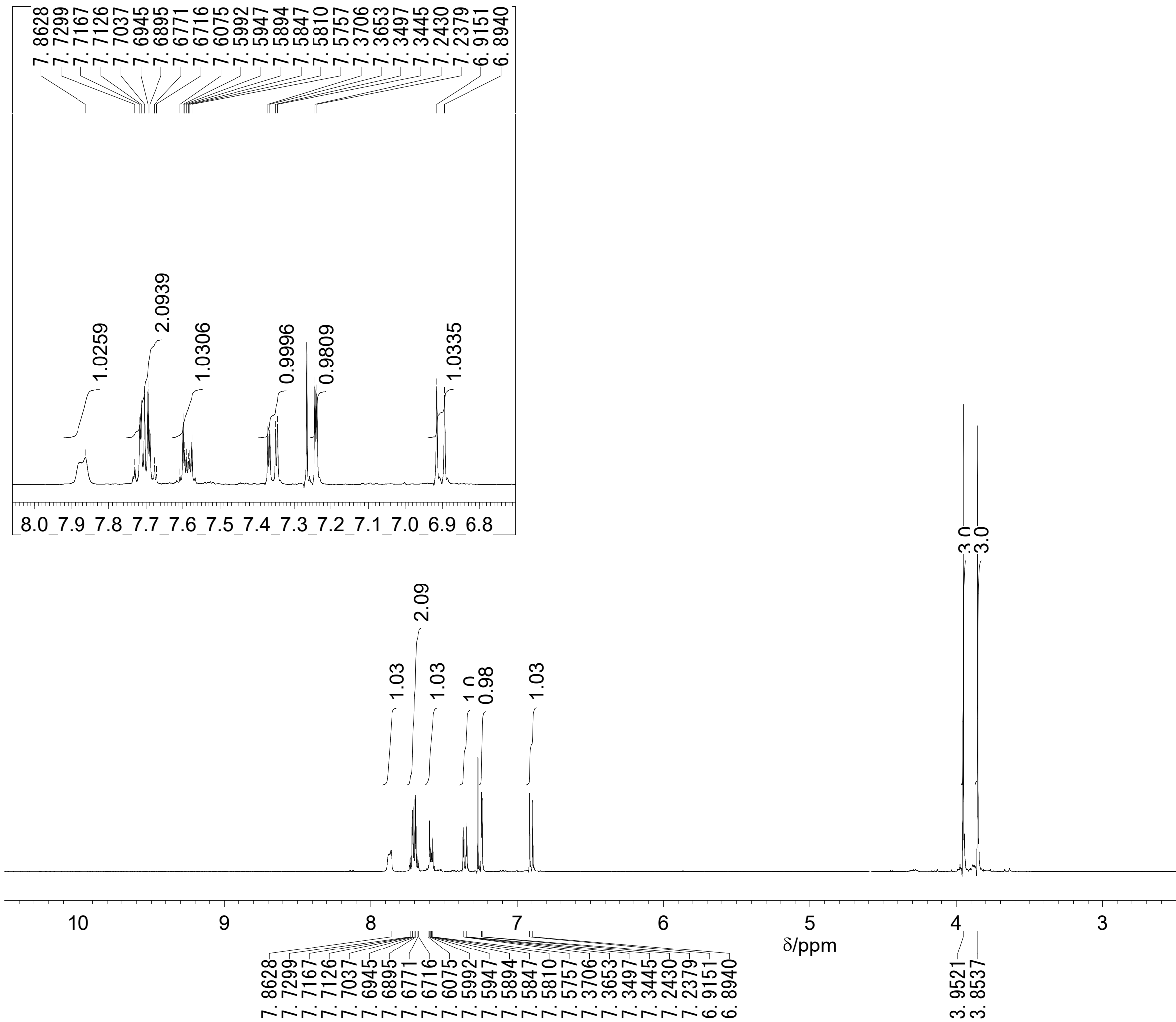

$^{13}C\{^1H\}$ NMR spectrum of **2k** (150 MHz, $CDCl_3$)

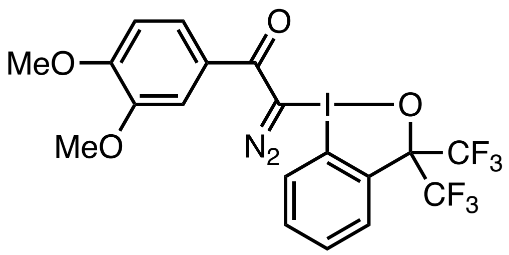

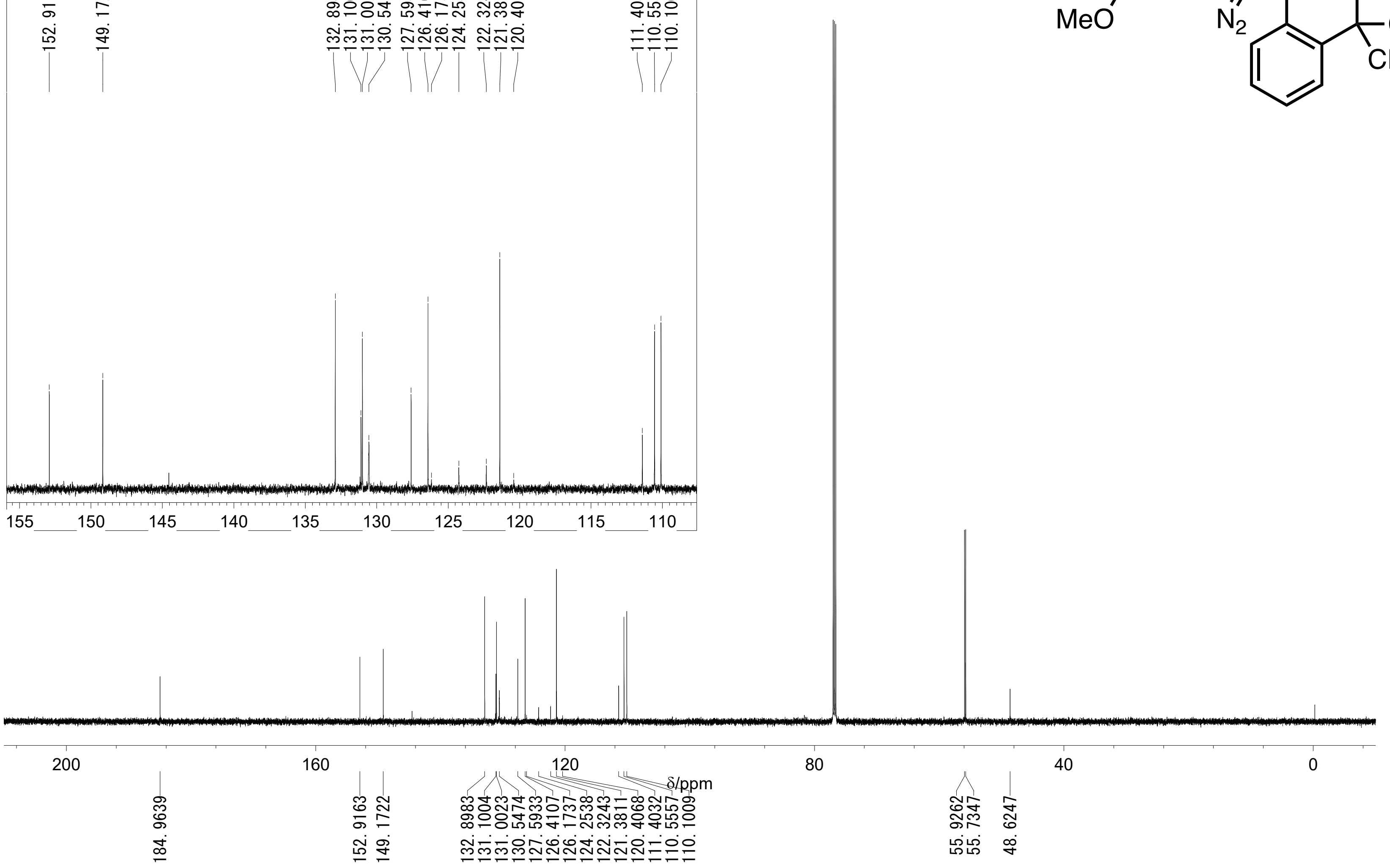

^{1}H NMR spectrum of **2l** (400 MHz, $CDCl_3$)

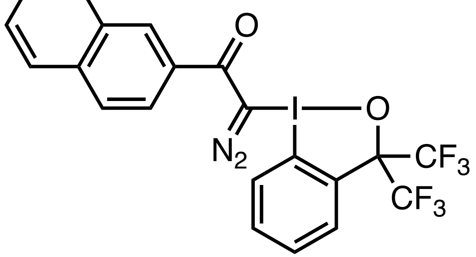

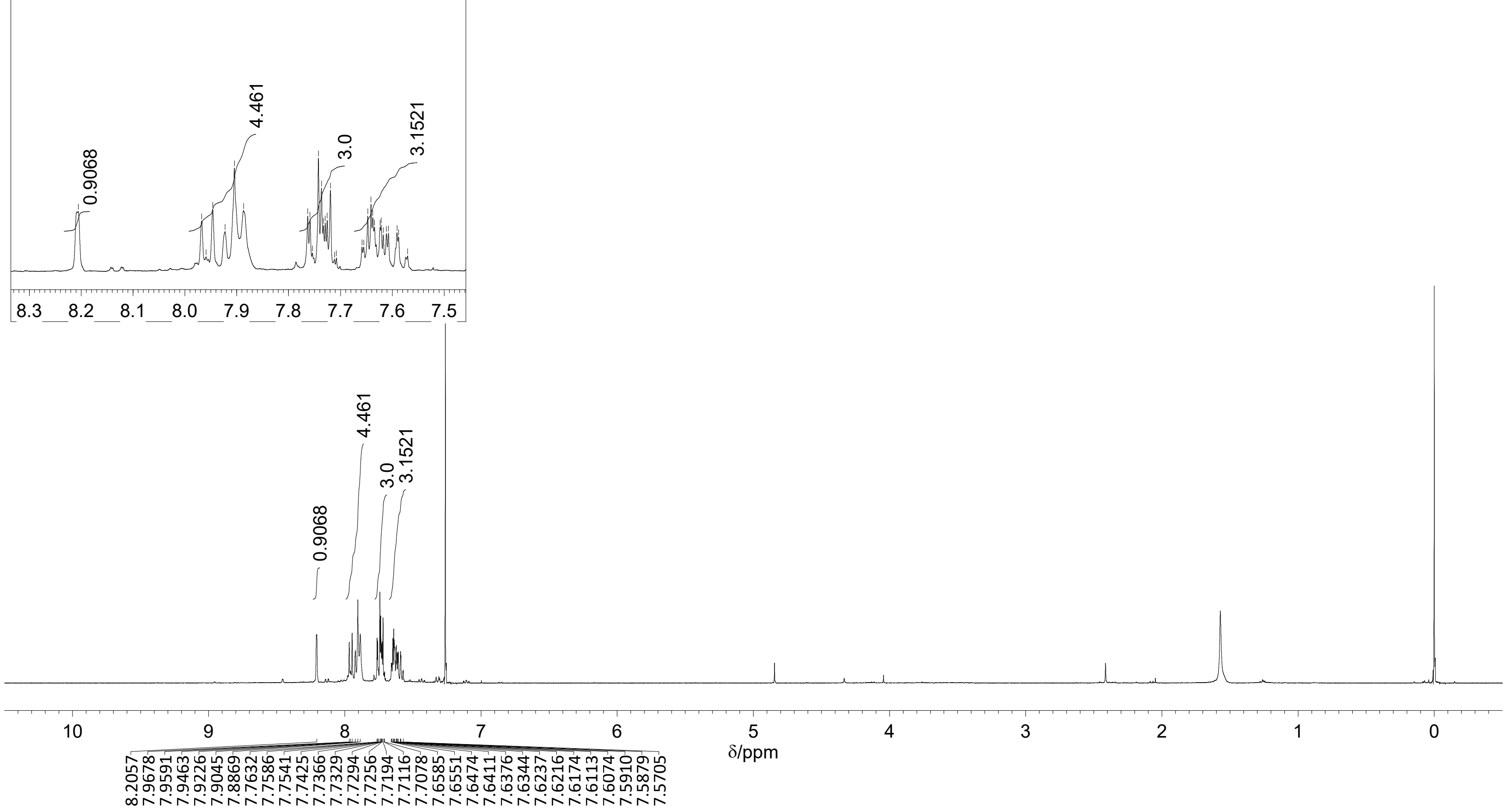

$^{13}C\{^1H\}$ NMR spectrum of **2l** (150 MHz, $CDCl_3$)

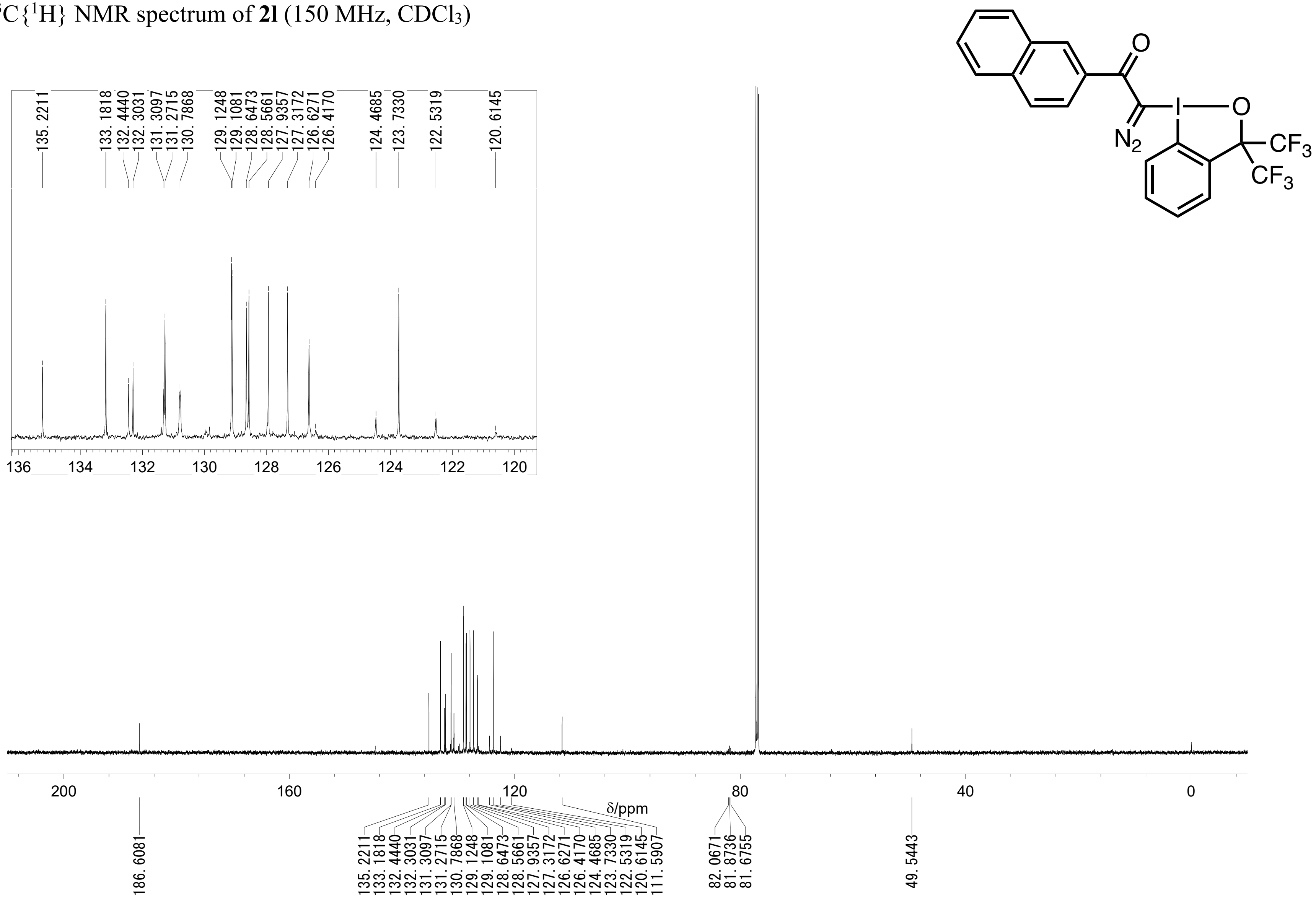

^{1}H NMR spectrum of **2m** (400 MHz, $CDCl_3$)

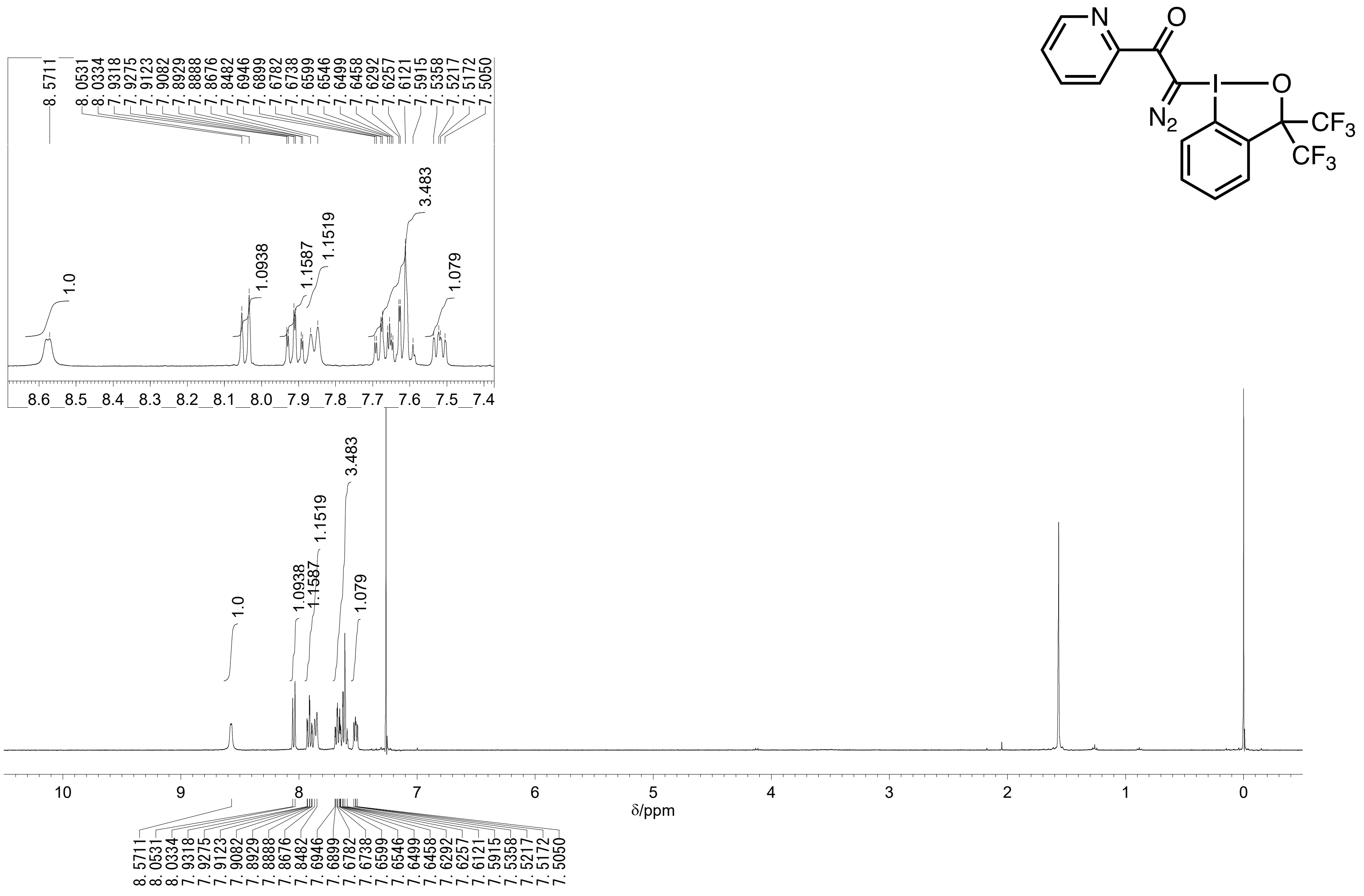

$^{13}C\{^{1}H\}$ NMR spectrum of **2m** (150 MHz, $CDCl_3$)

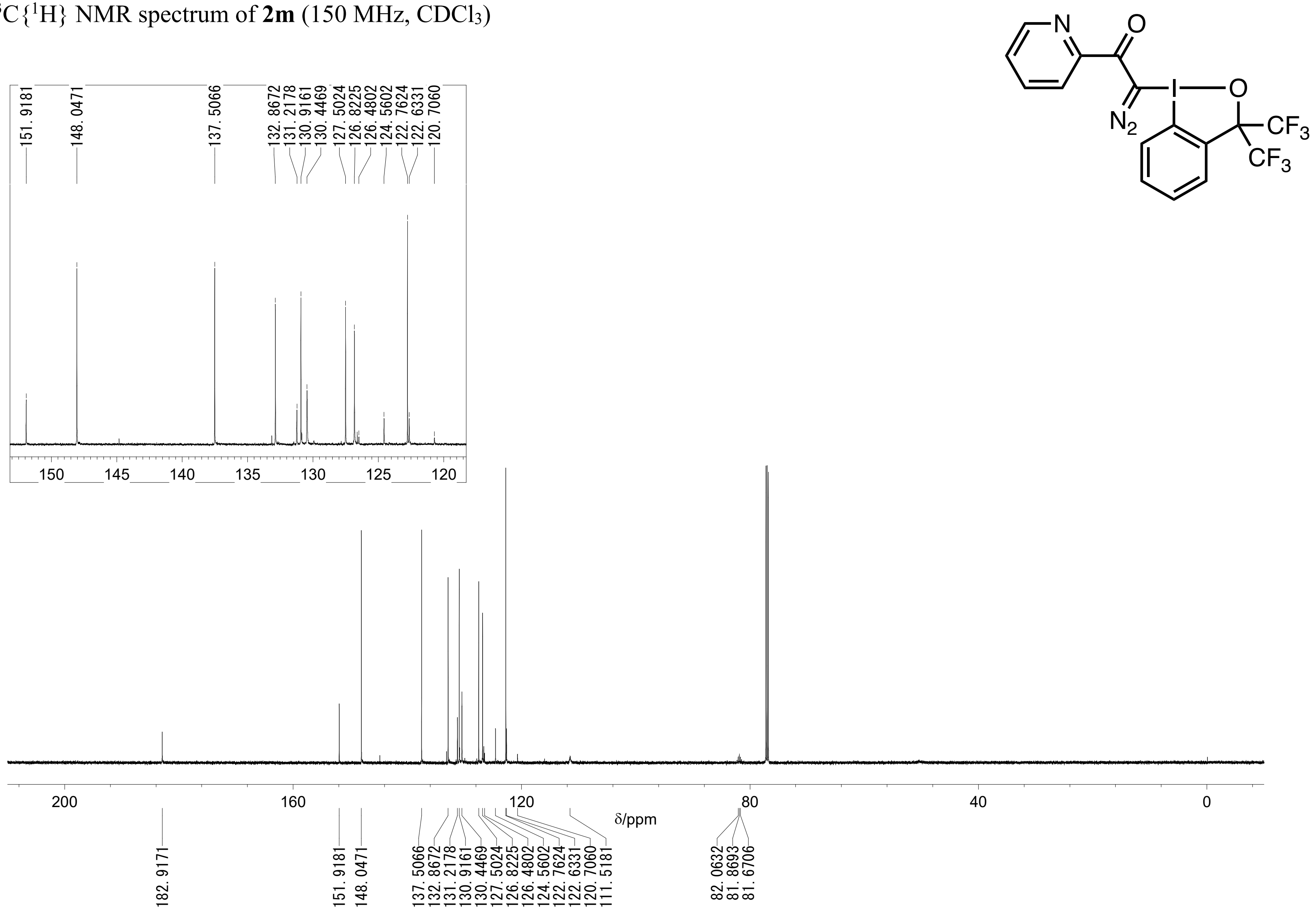

^{1}H NMR spectrum of **2n** (400 MHz, $CDCl_3$)

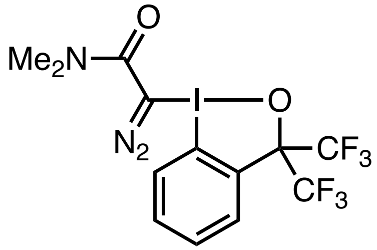

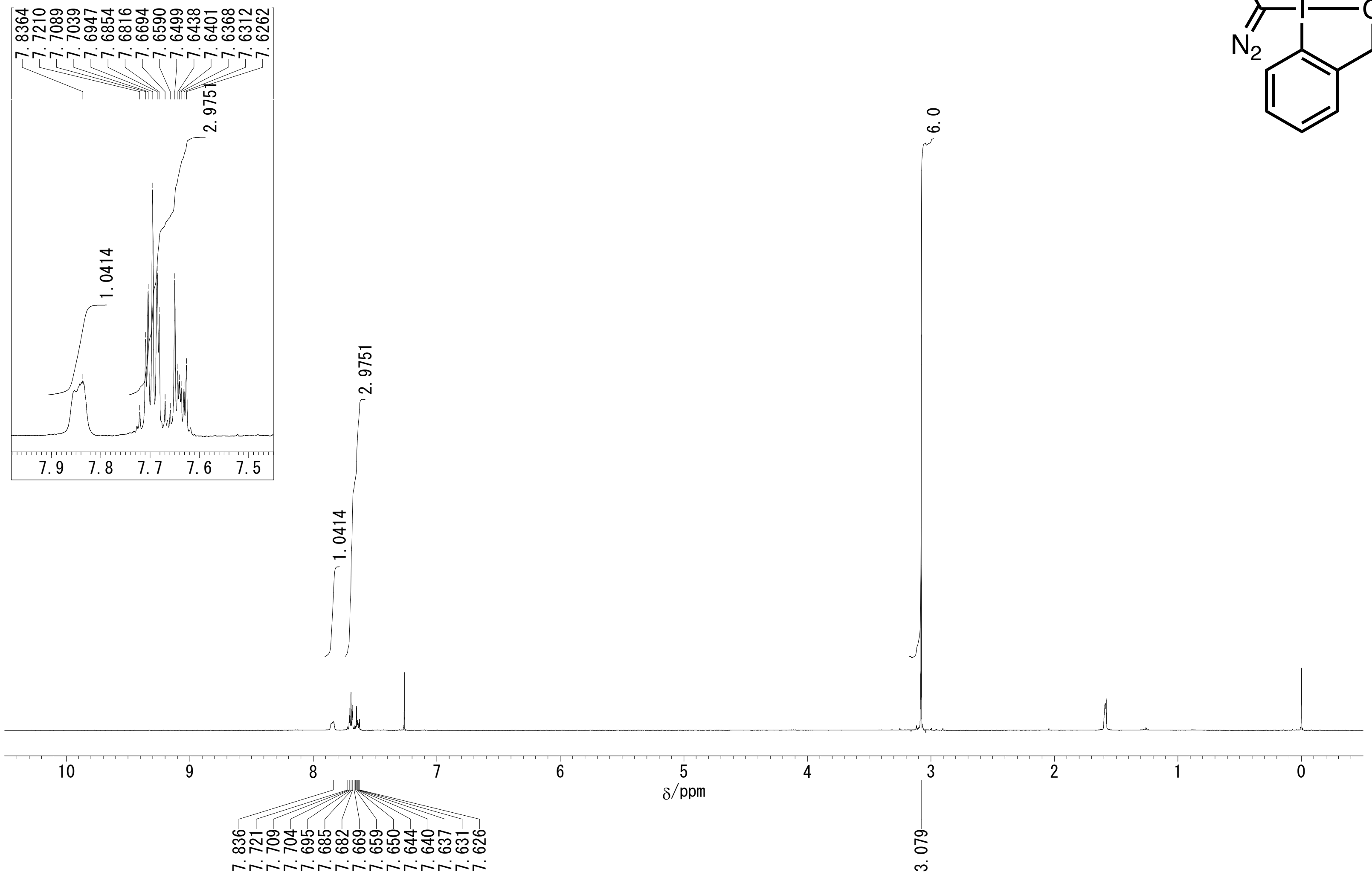

$^{13}C\{^{1}H\}$ NMR spectrum of **2n** (150 MHz, $CDCl_3$)

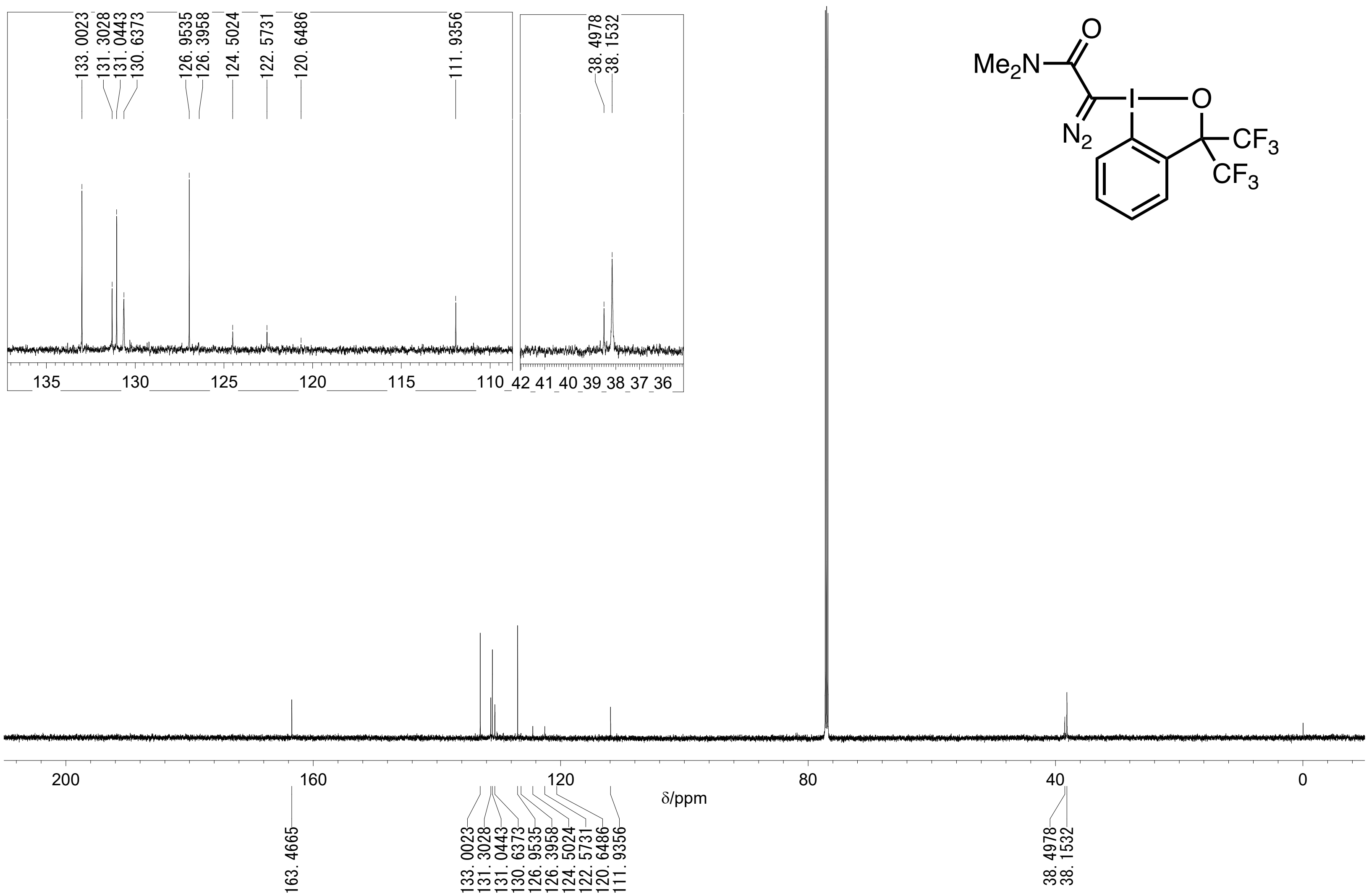

^{1}H NMR spectrum of **2o** (400 MHz, $CDCl_3$)

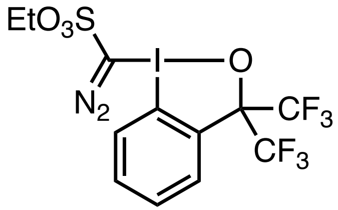

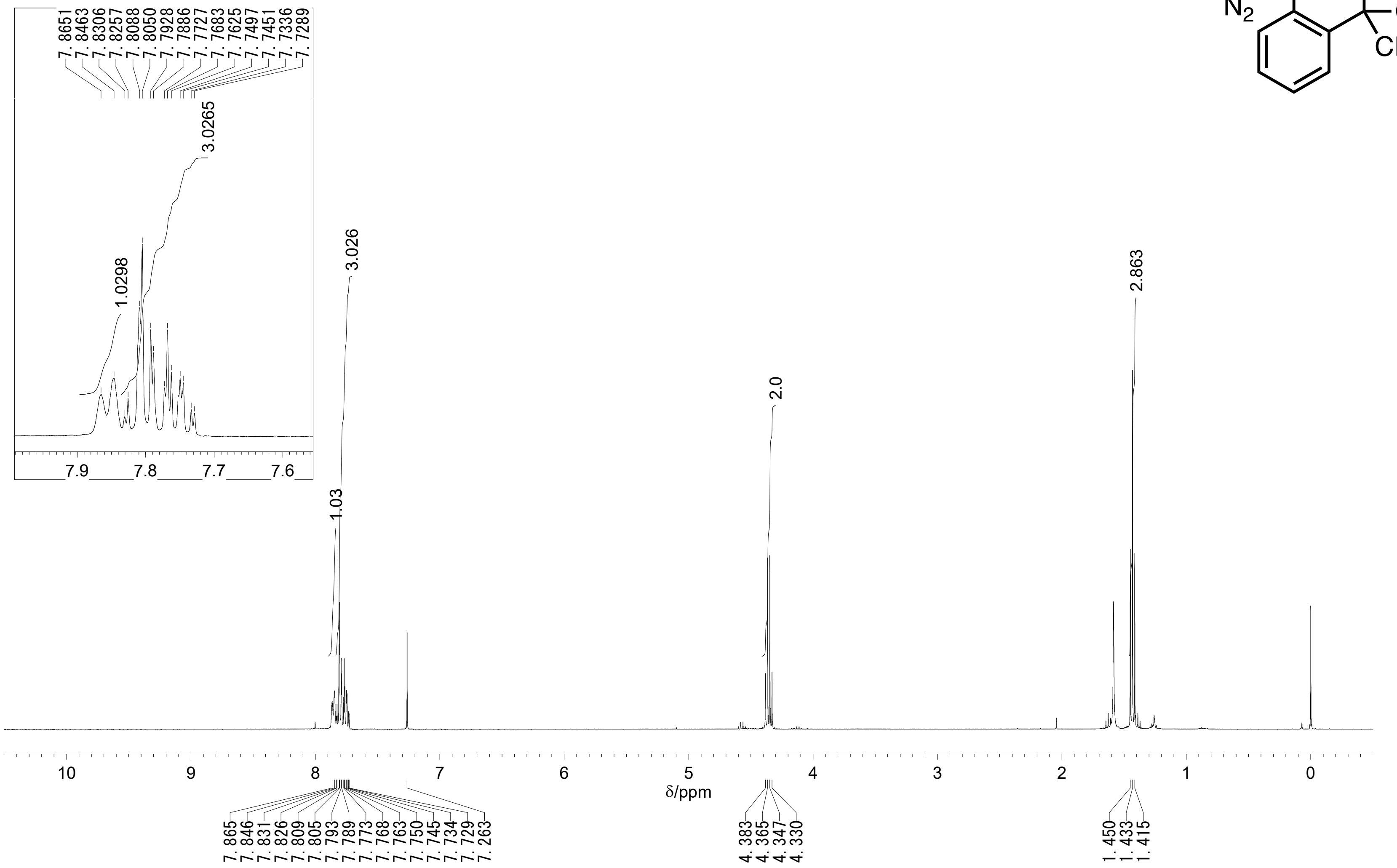

$^{13}C\{^{1}H\}$ NMR spectrum of **2o** (150 MHz, $CDCl_3$)

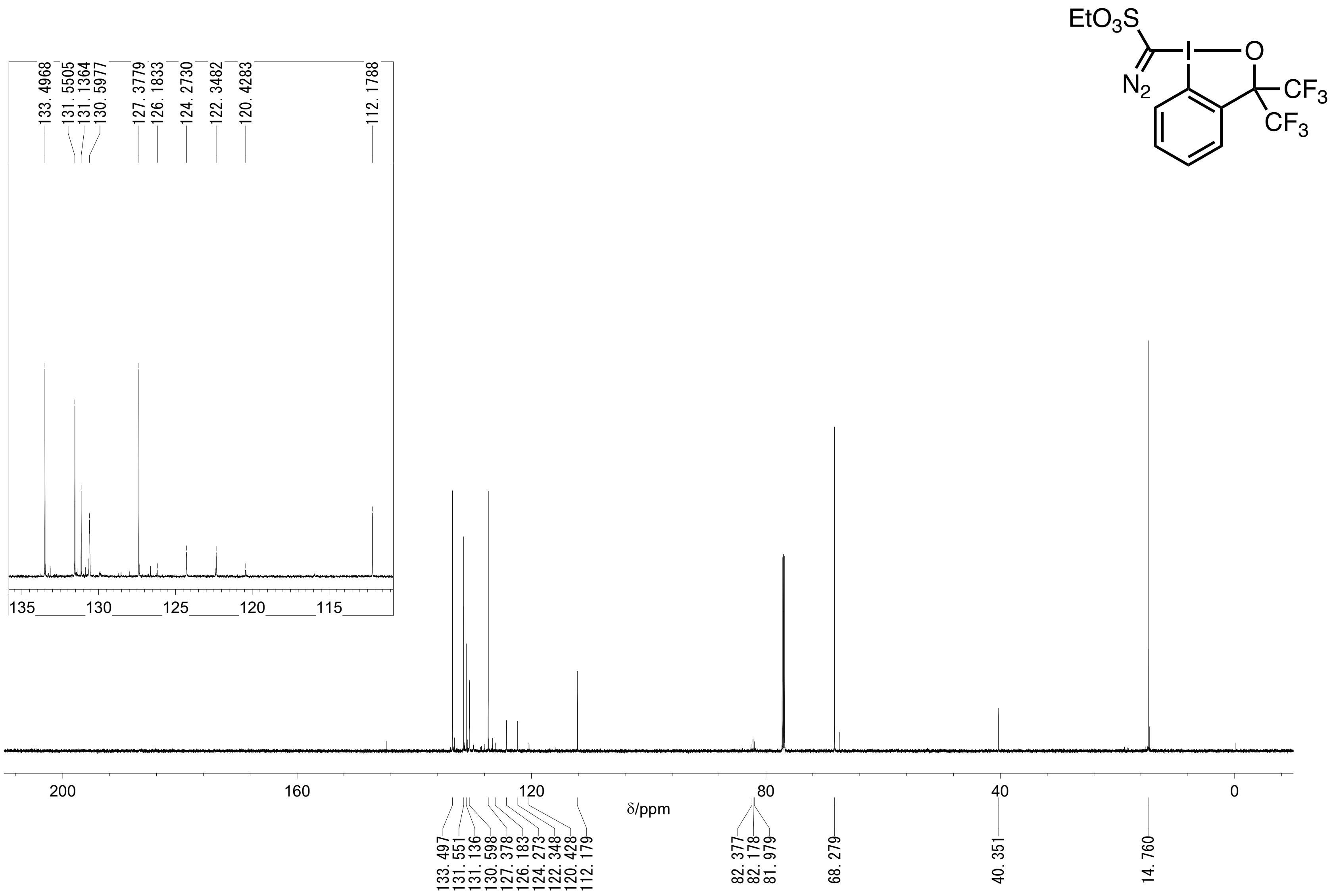

^{1}H NMR spectrum of **2p** (400 MHz, $CDCl_3$)

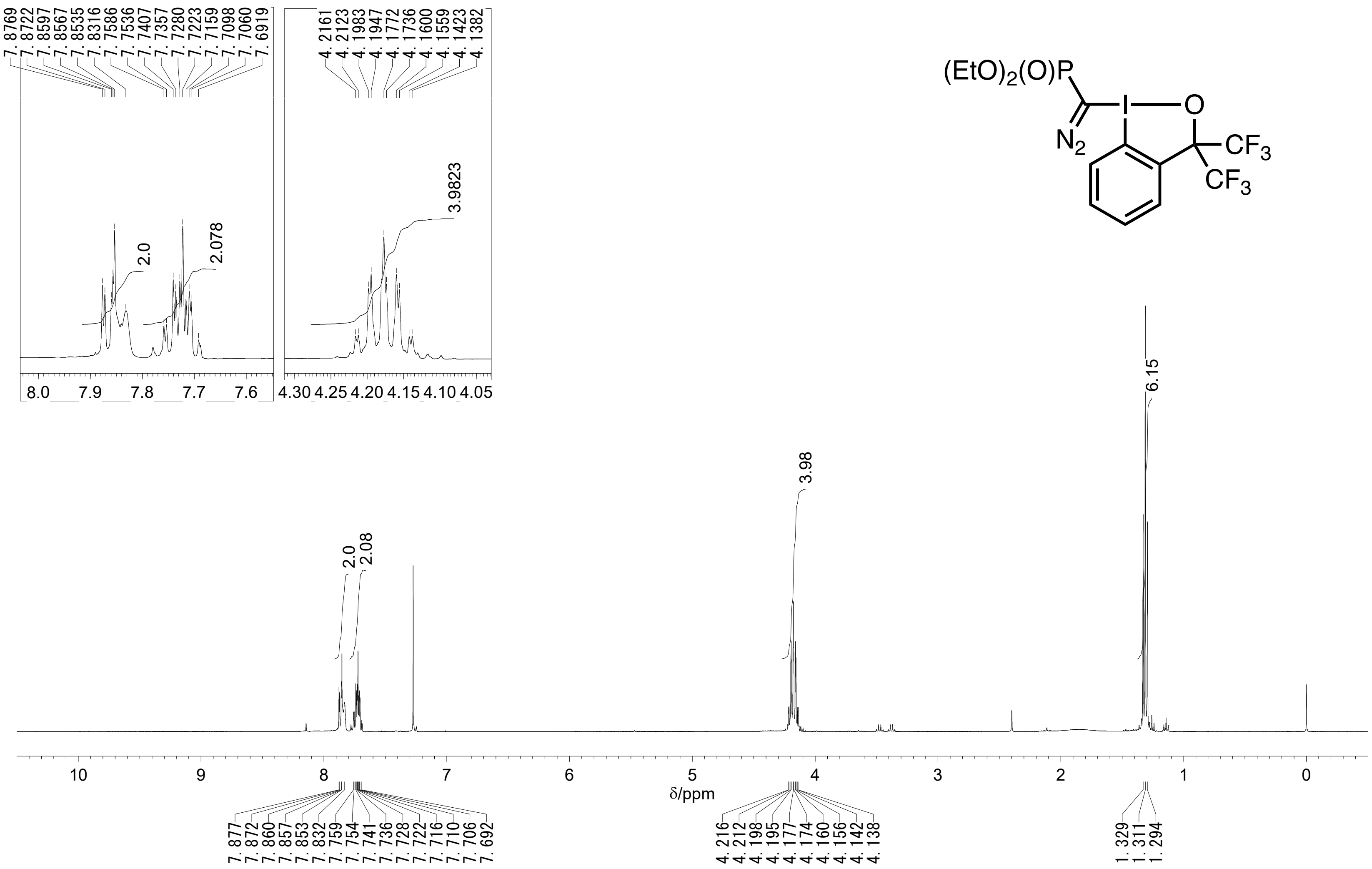

$^{13}C\{^1H\}$ NMR spectrum of **2p** (150 MHz, $CDCl_3$)

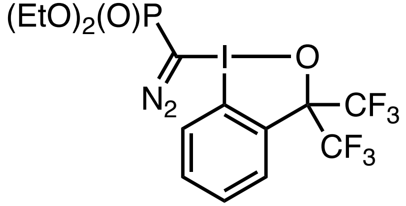

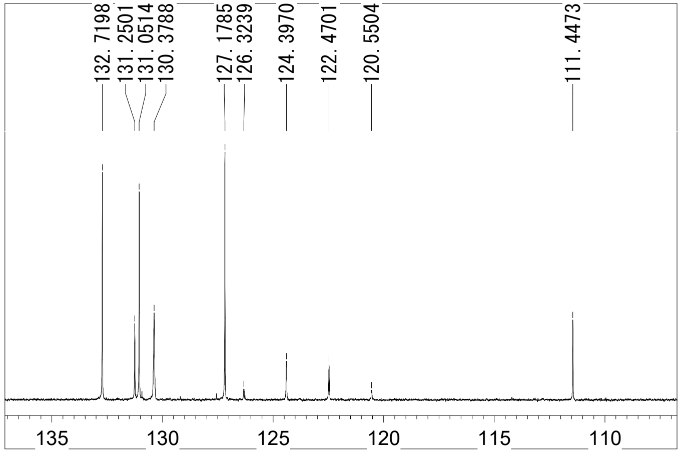

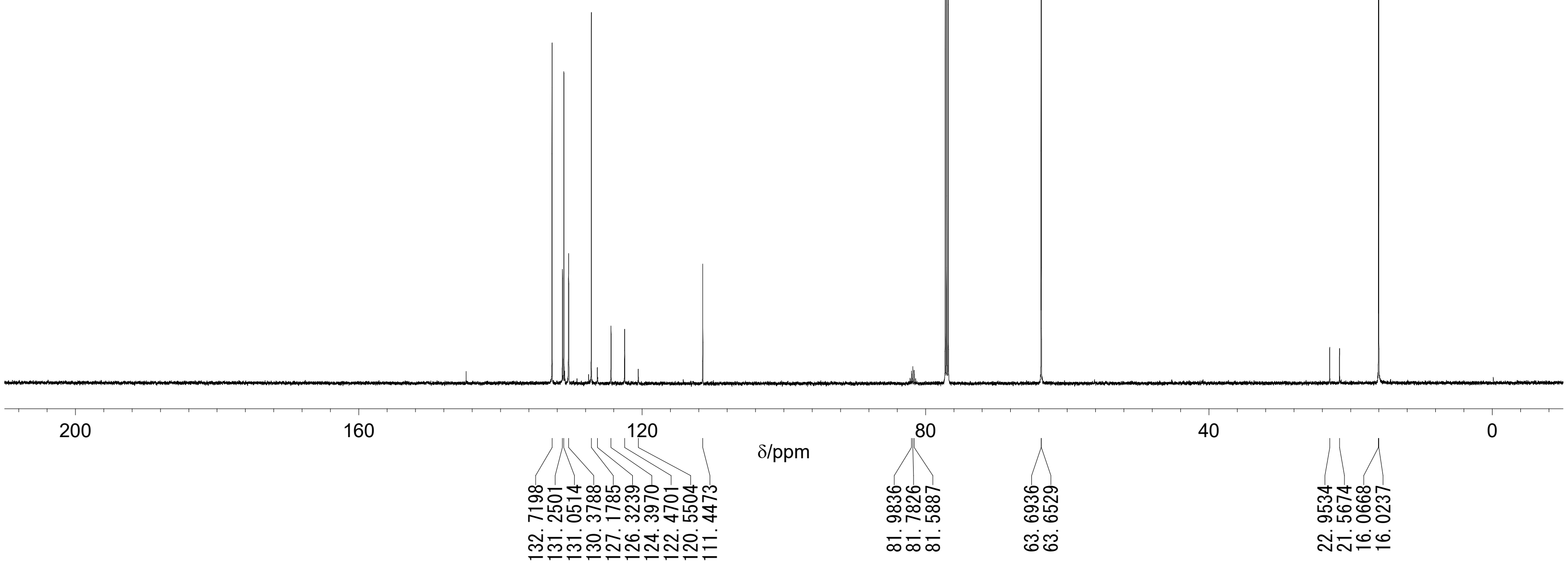

^{1}H NMR spectrum of **3aa** (400 MHz, $CDCl_3$)

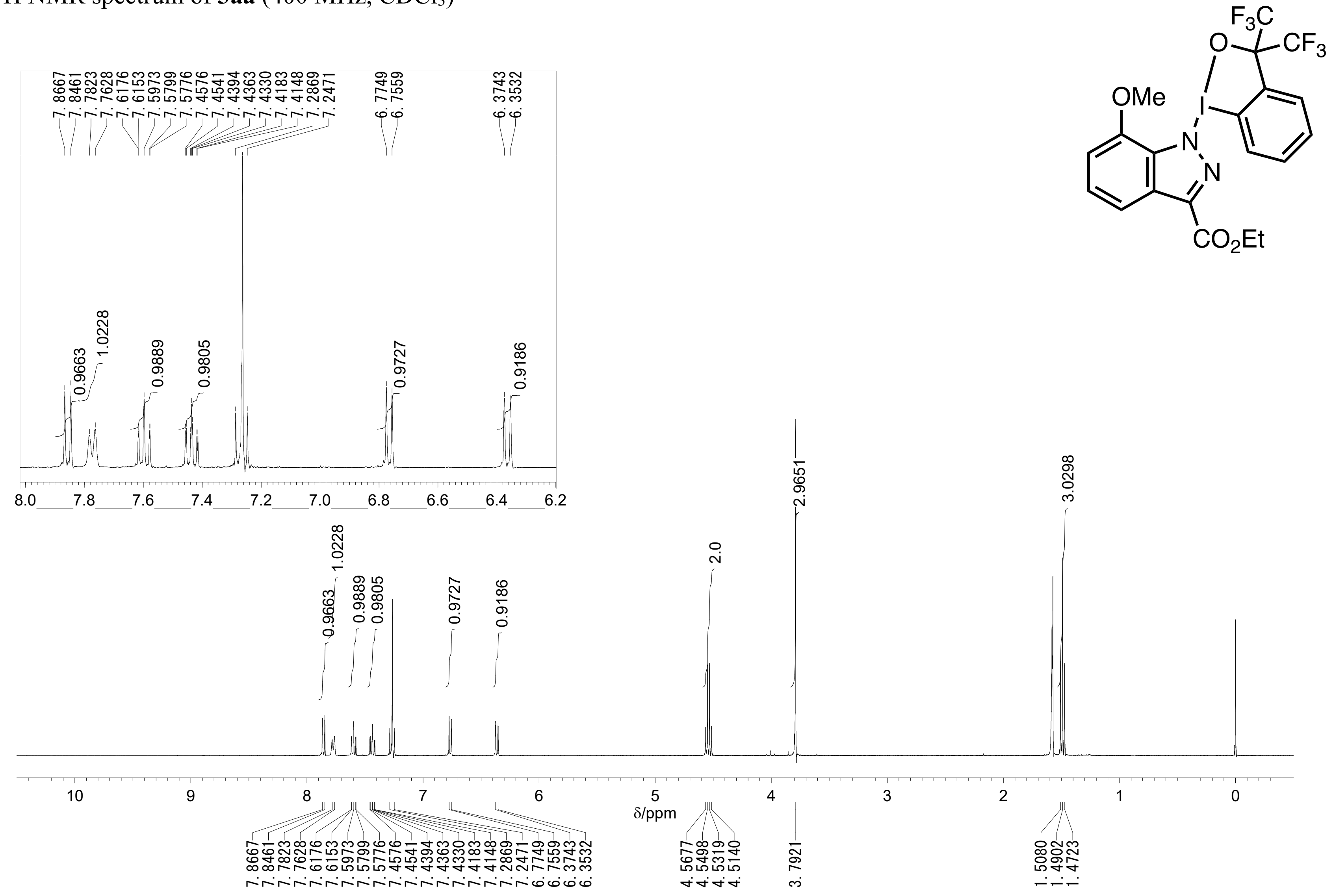

$^{13}C\{^{1}H\}$ NMR spectrum of **3aa** (150 MHz, $CDCl_3$)

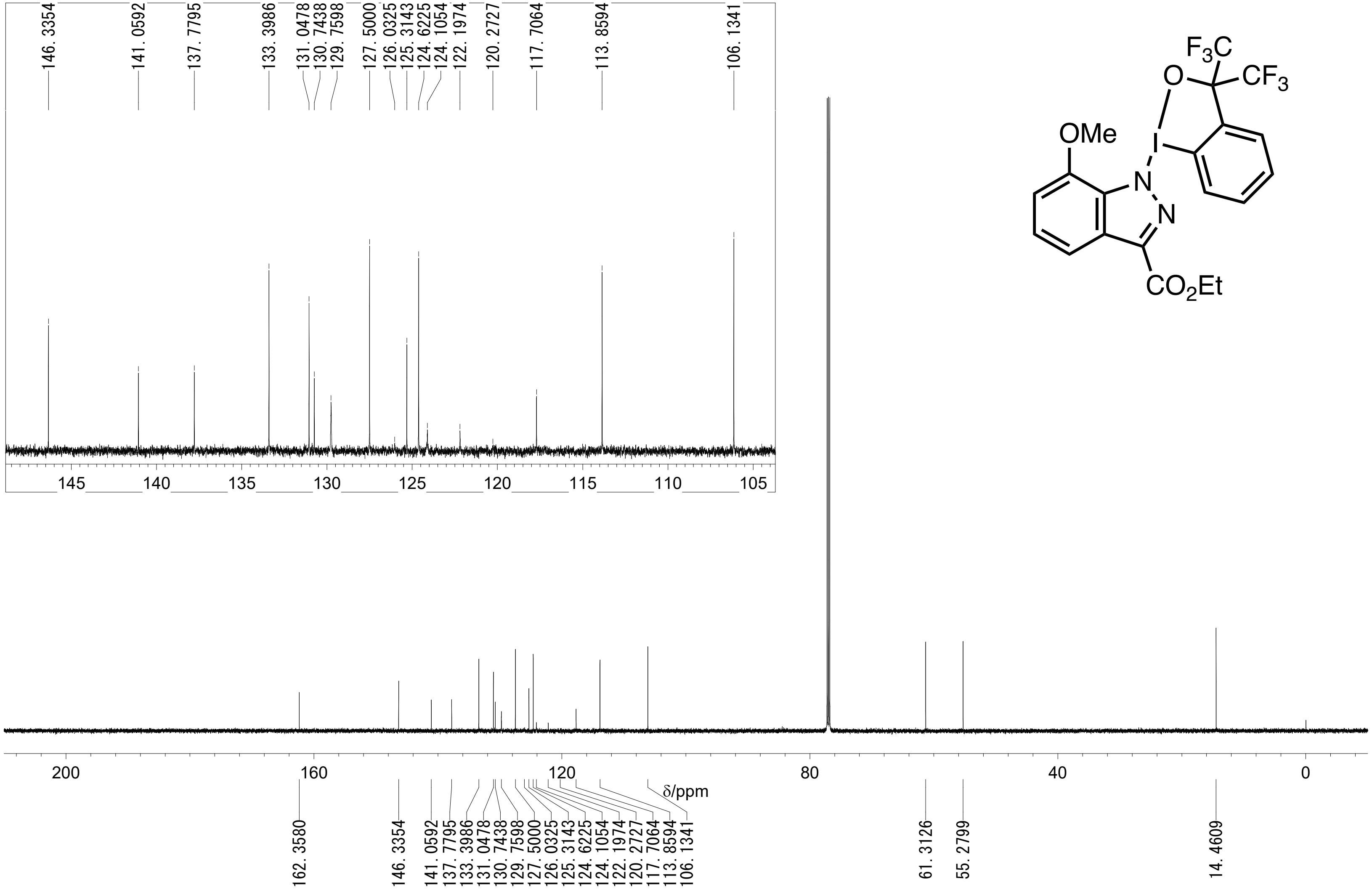

^{1}H NMR spectrum of **3ab** (400 MHz, $CDCl_3$)

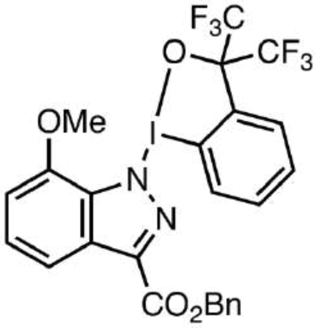

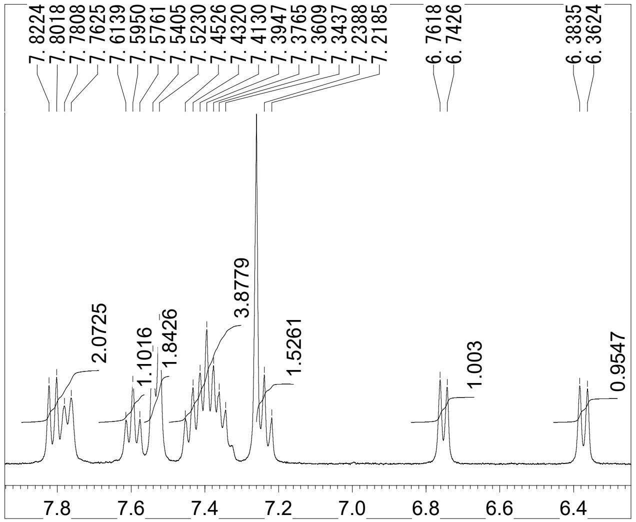

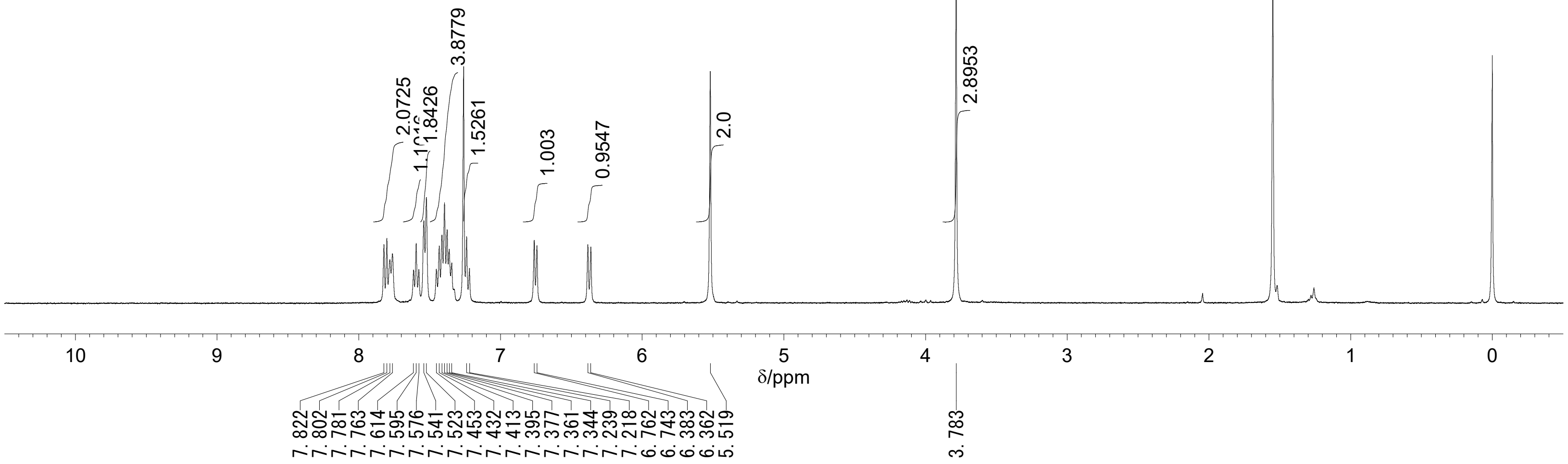

$^{13}C\{^1H\}$ NMR spectrum of **3ab** (150 MHz, $CDCl_3$)

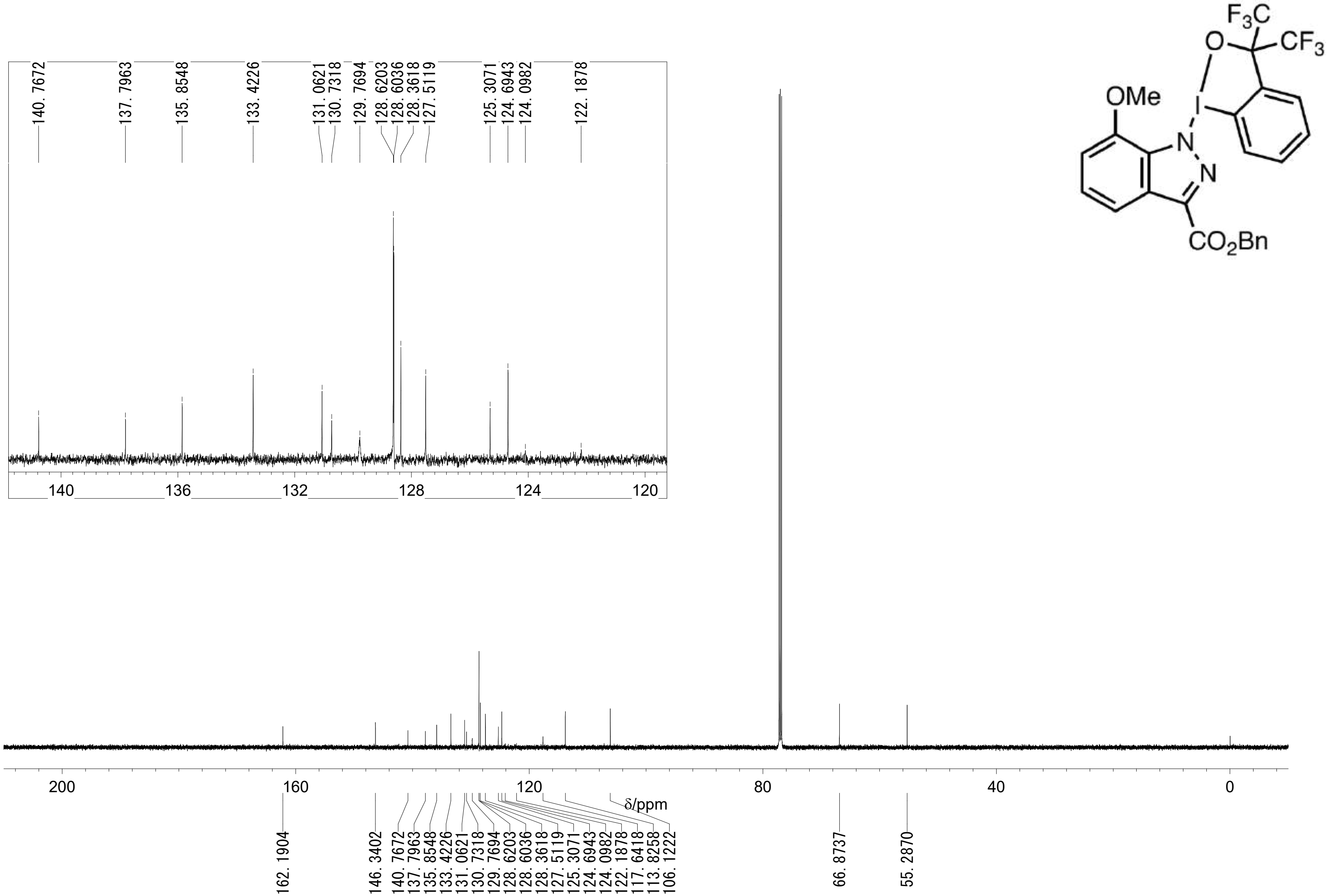

^{1}H NMR spectrum of **3ac** (400 MHz, $CDCl_3$)

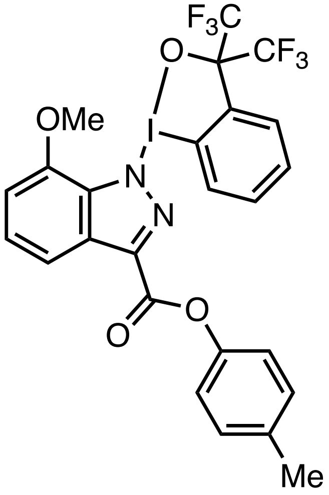

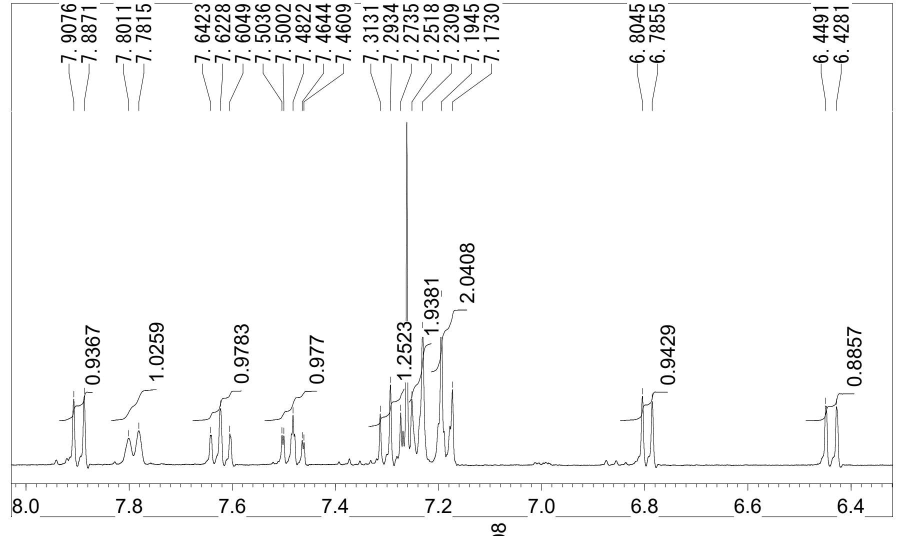

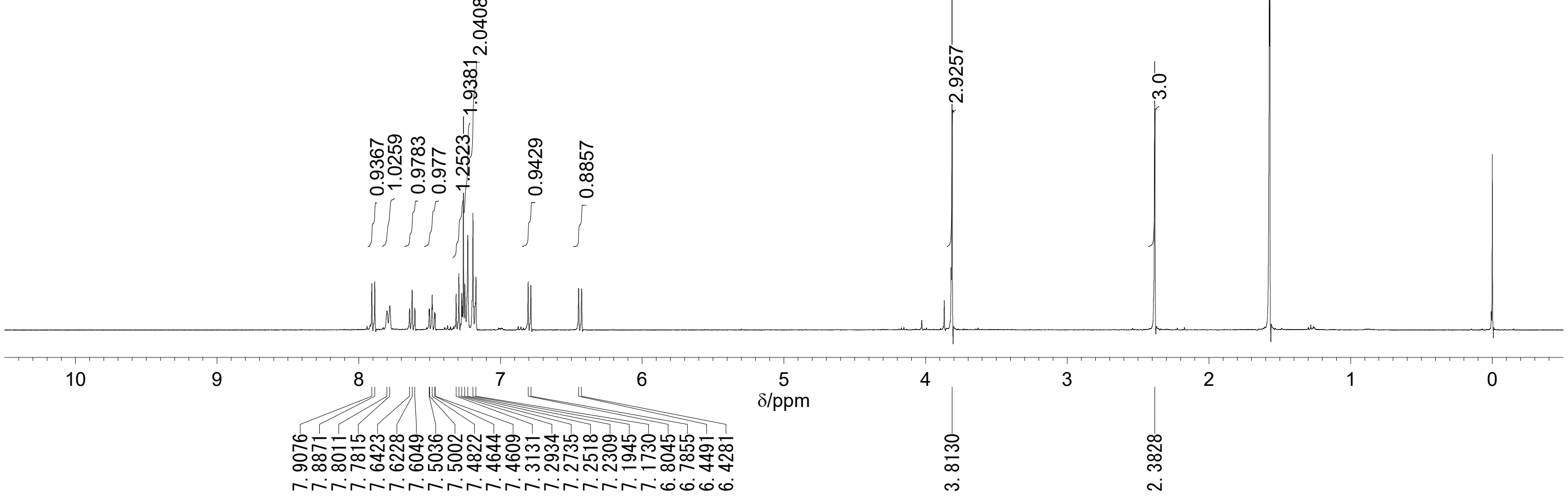

$^{13}C\{^1H\}$ NMR spectrum of **3ac** (150 MHz, $CDCl_3$)

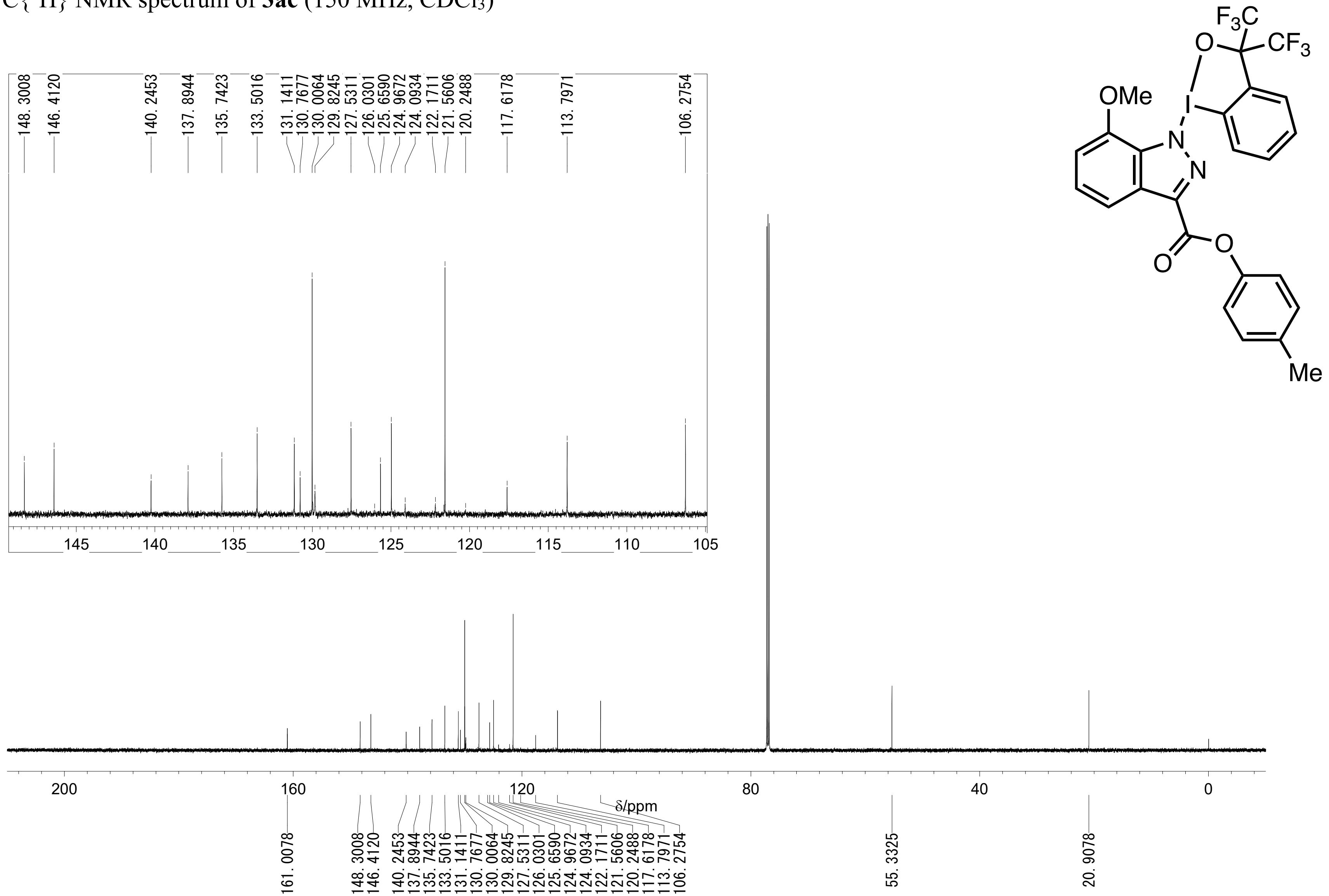

^{1}H NMR spectrum of **3ad** (400 MHz, $CDCl_3$)

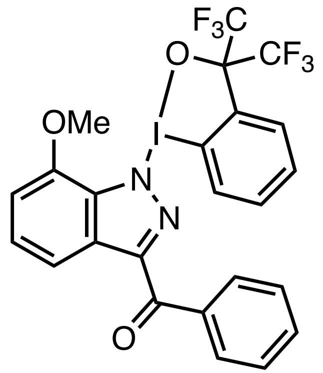

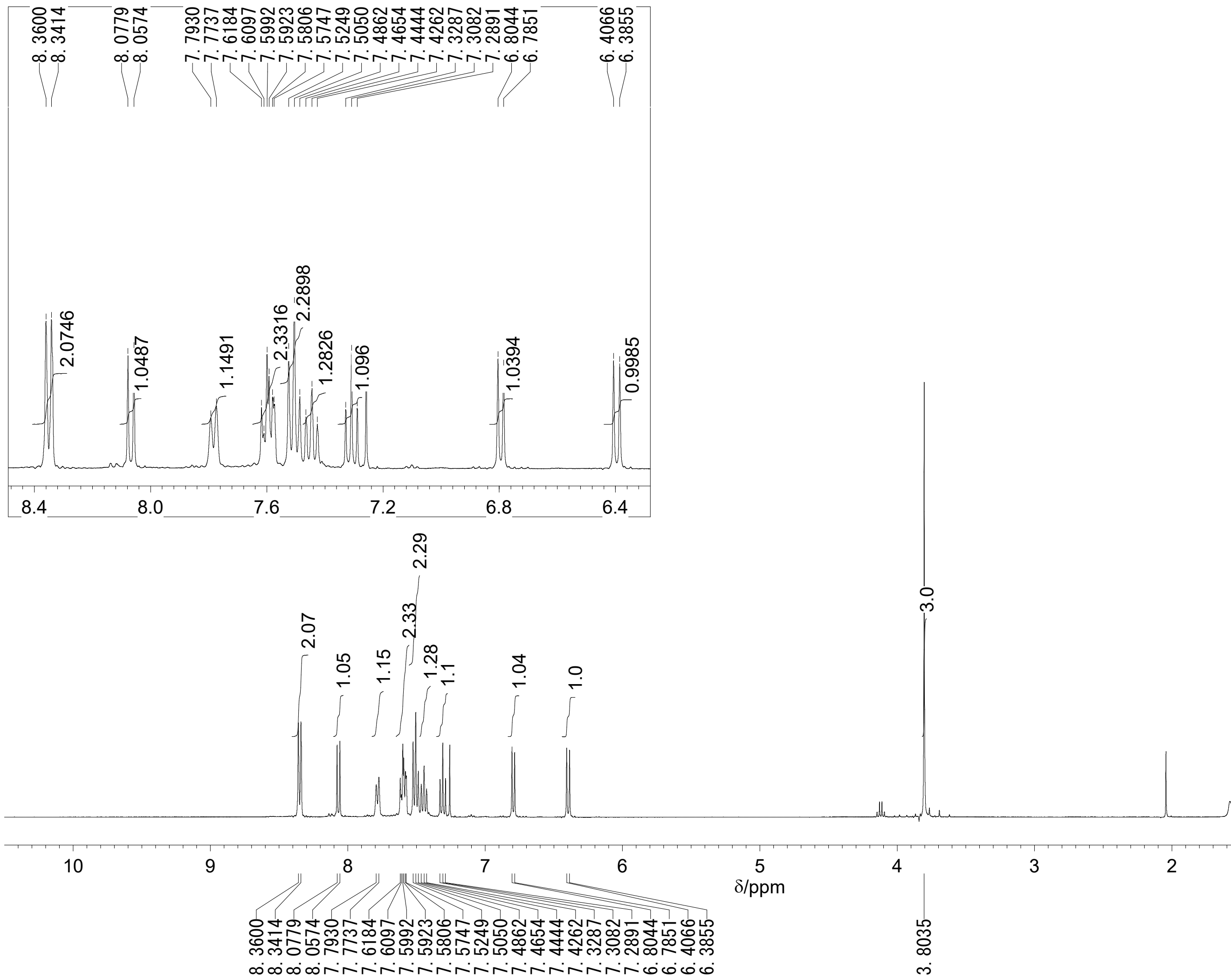

$^{13}C\{^1H\}$ NMR spectrum of **3ad** (150 MHz, $CDCl_3$)

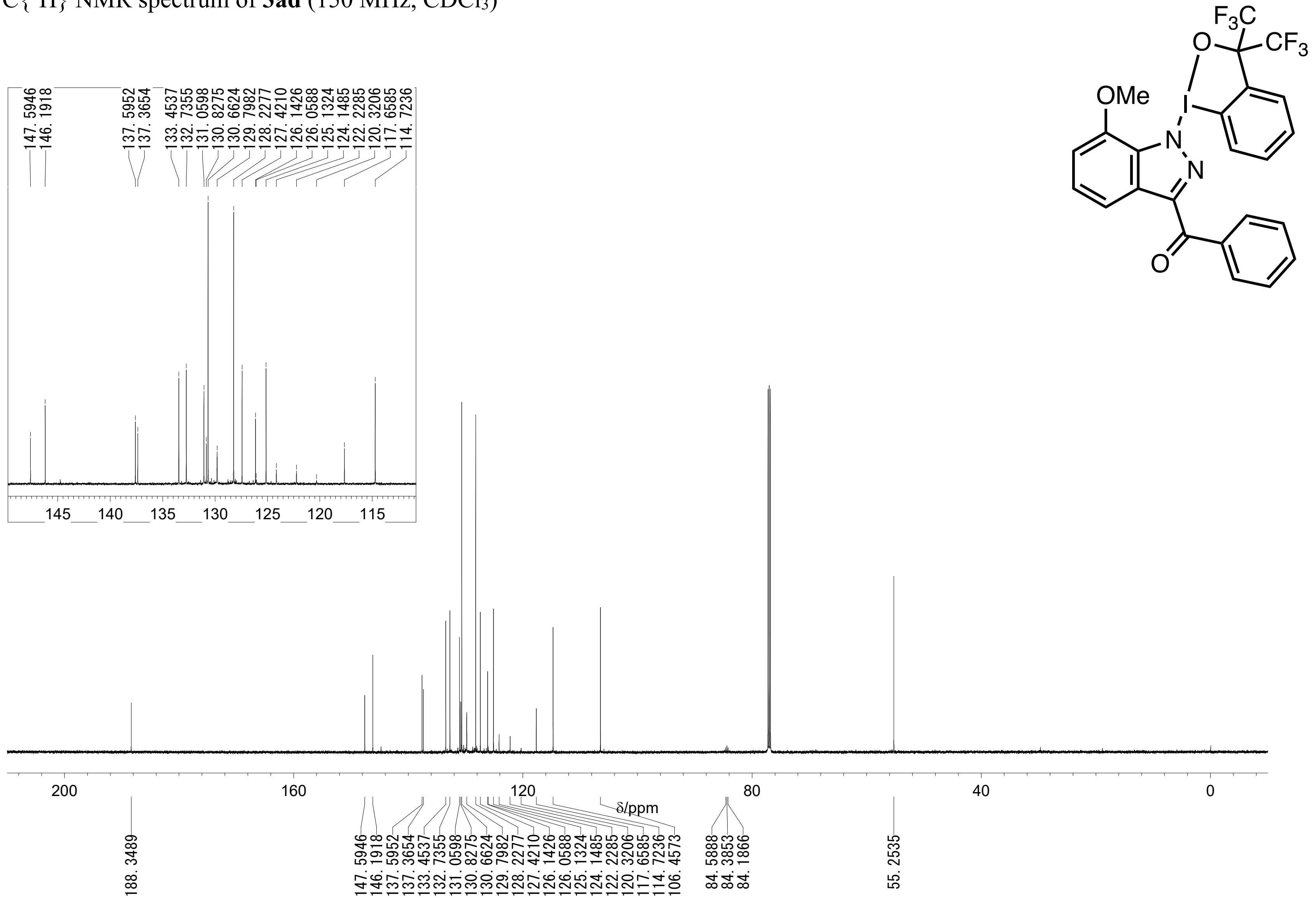

^{1}H NMR spectrum of **3ae** (400 MHz, $CDCl_3$)

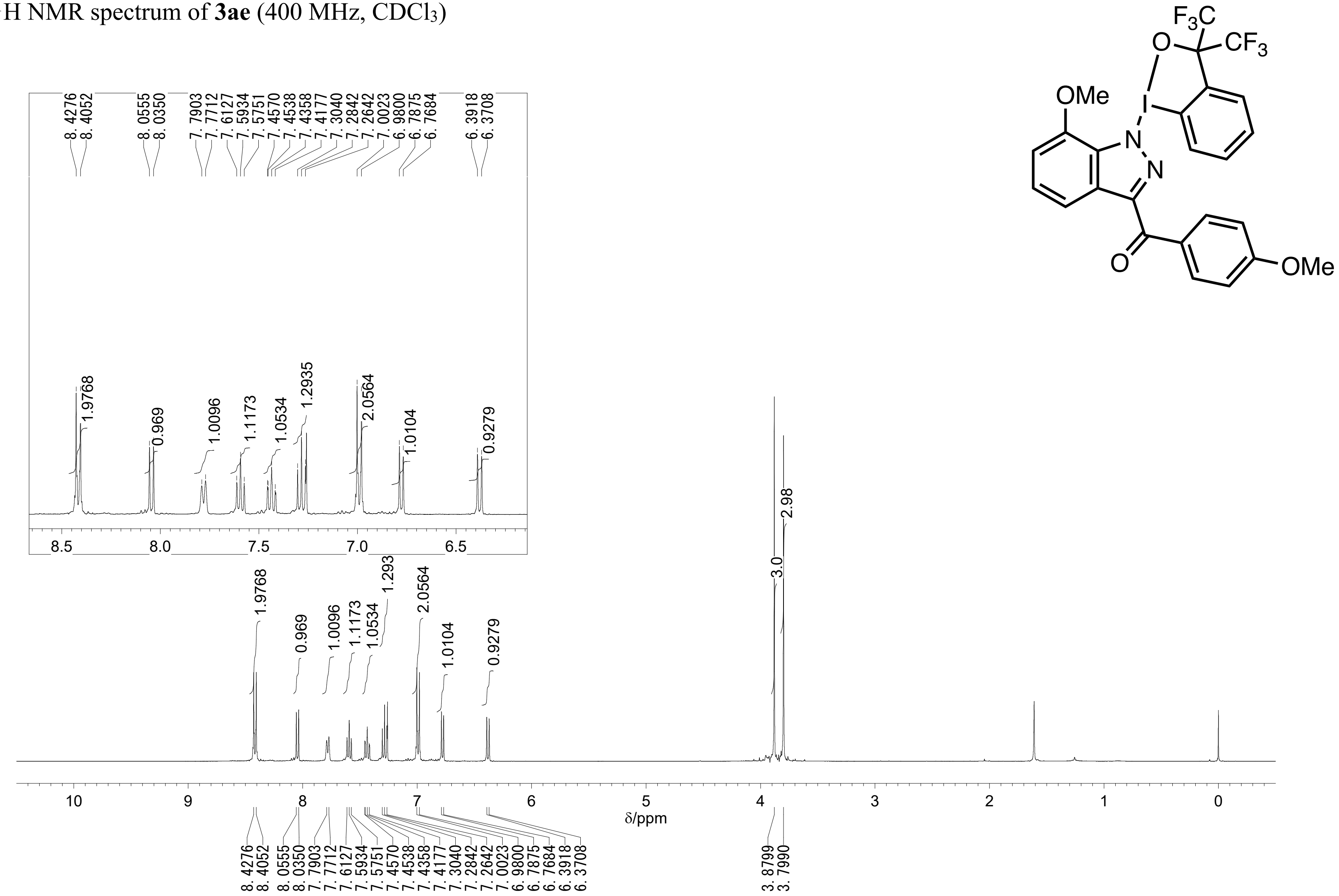

$^{13}C\{^{1}H\}$ NMR spectrum of **3ae** (150 MHz, $CDCl_3$)

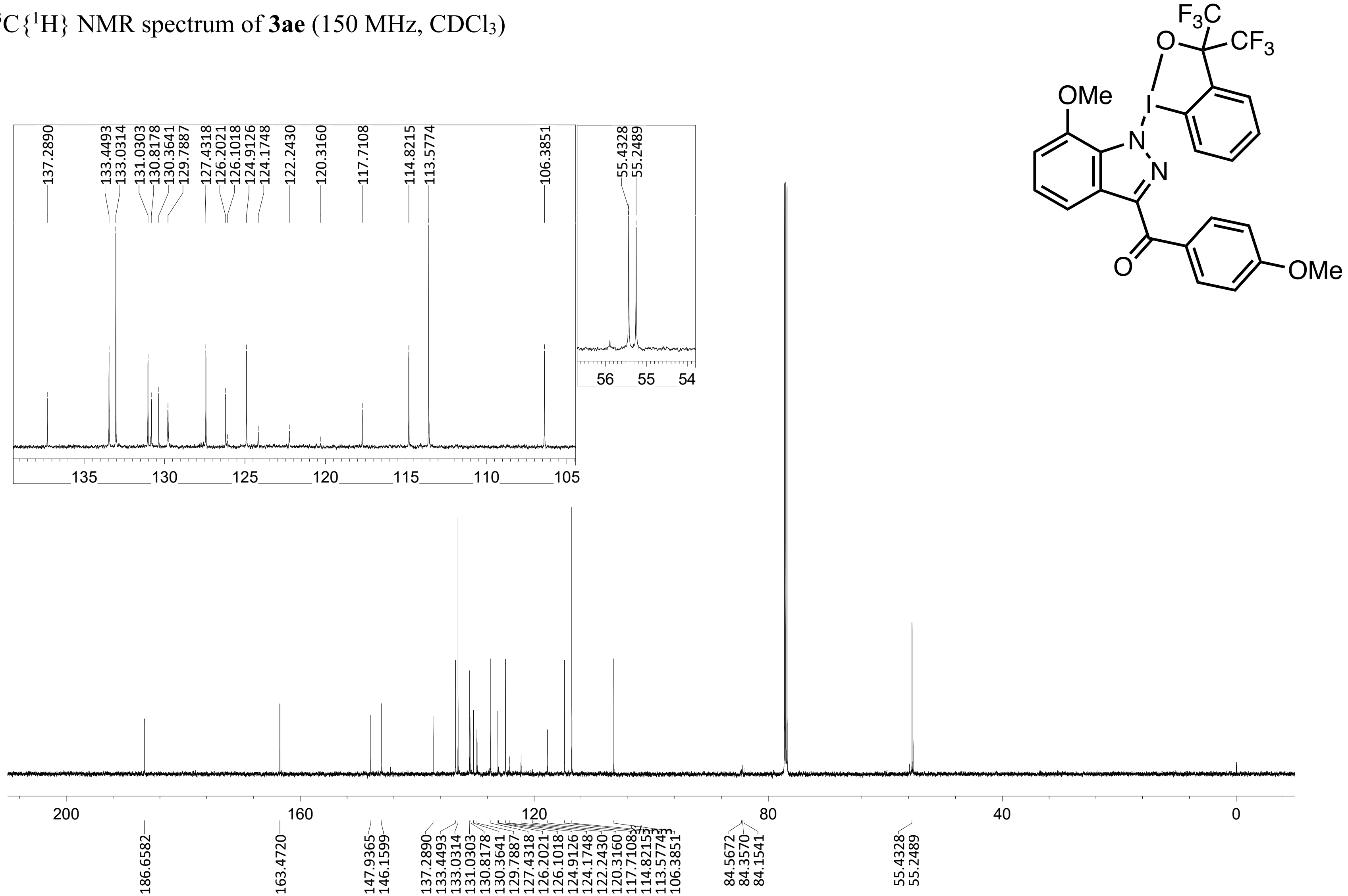

^{1}H NMR spectrum of **3af** (400 MHz, $CDCl_3$)

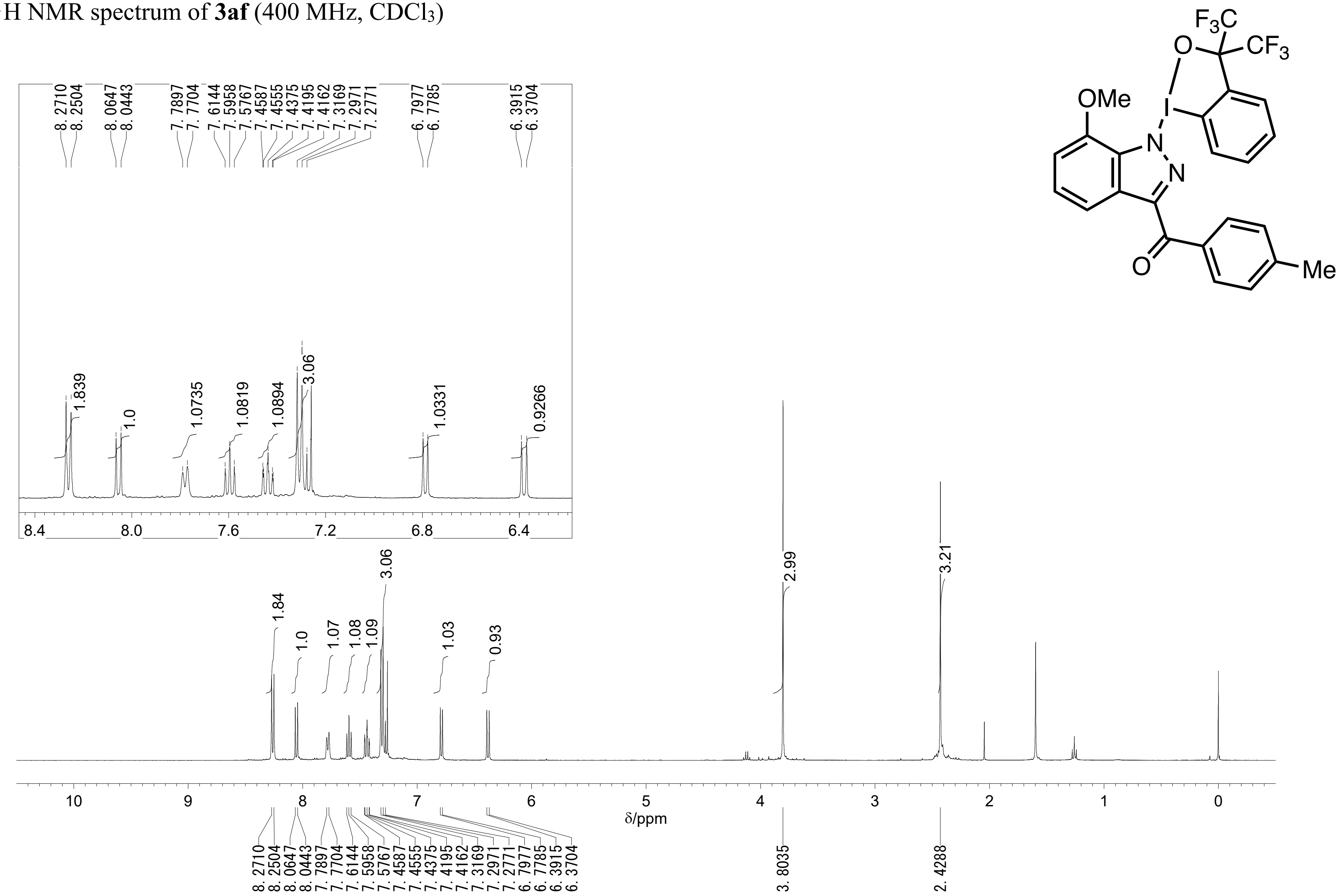

$^{13}C\{^1H\}$ NMR spectrum of **3af** (150 MHz, $CDCl_3$)

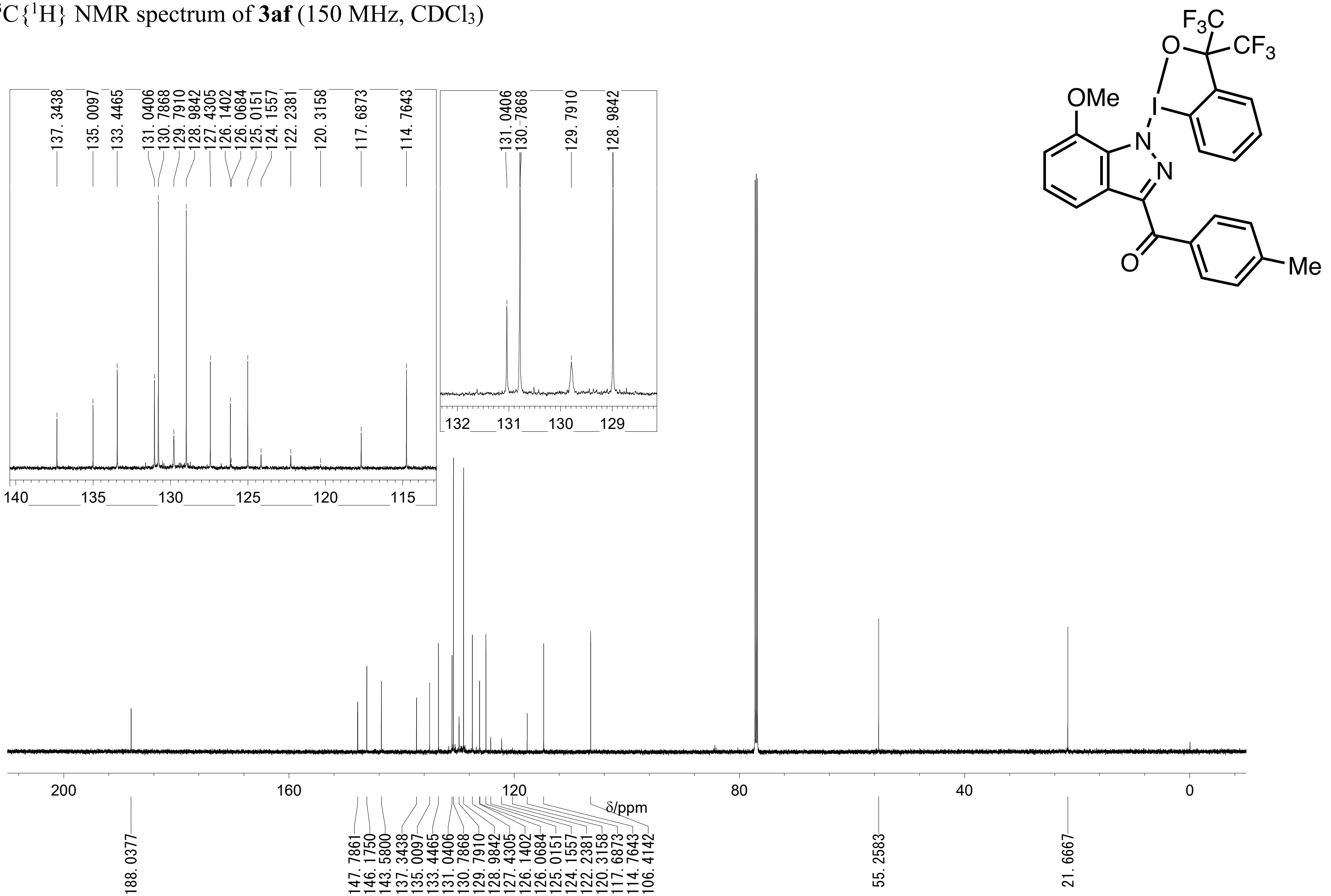

^{1}H NMR spectrum of **3ag** (400 MHz, $CDCl_3$)

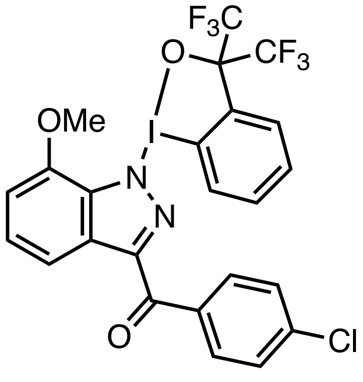

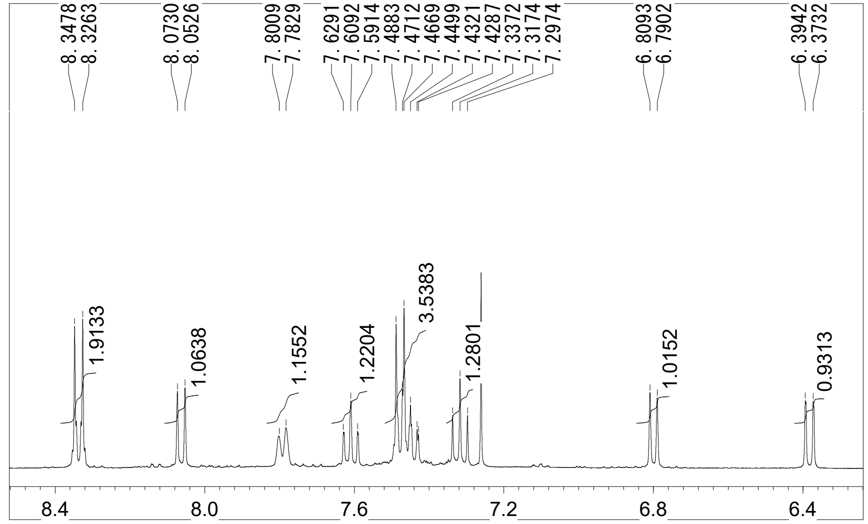

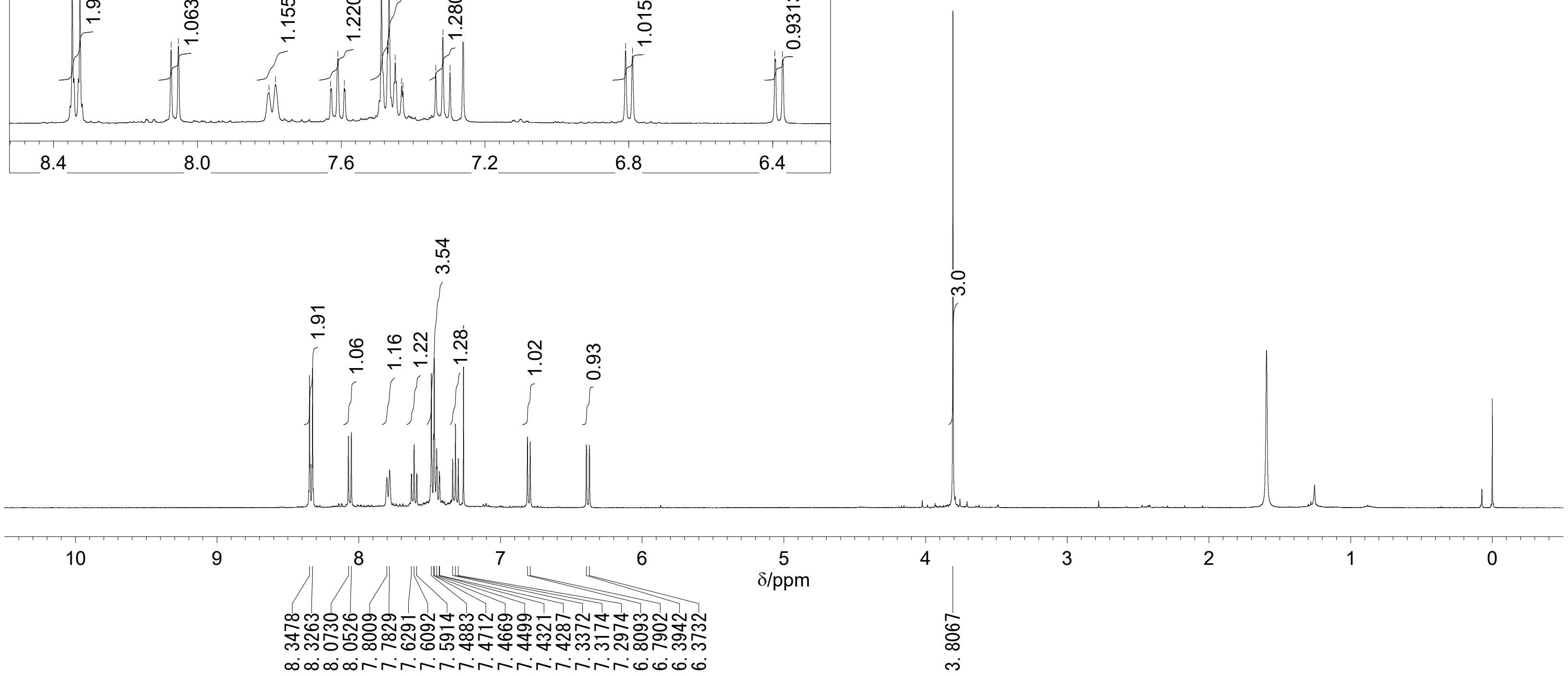

$^{13}C\{^{1}H\}$ NMR spectrum of **3ag** (150 MHz, $CDCl_3$)

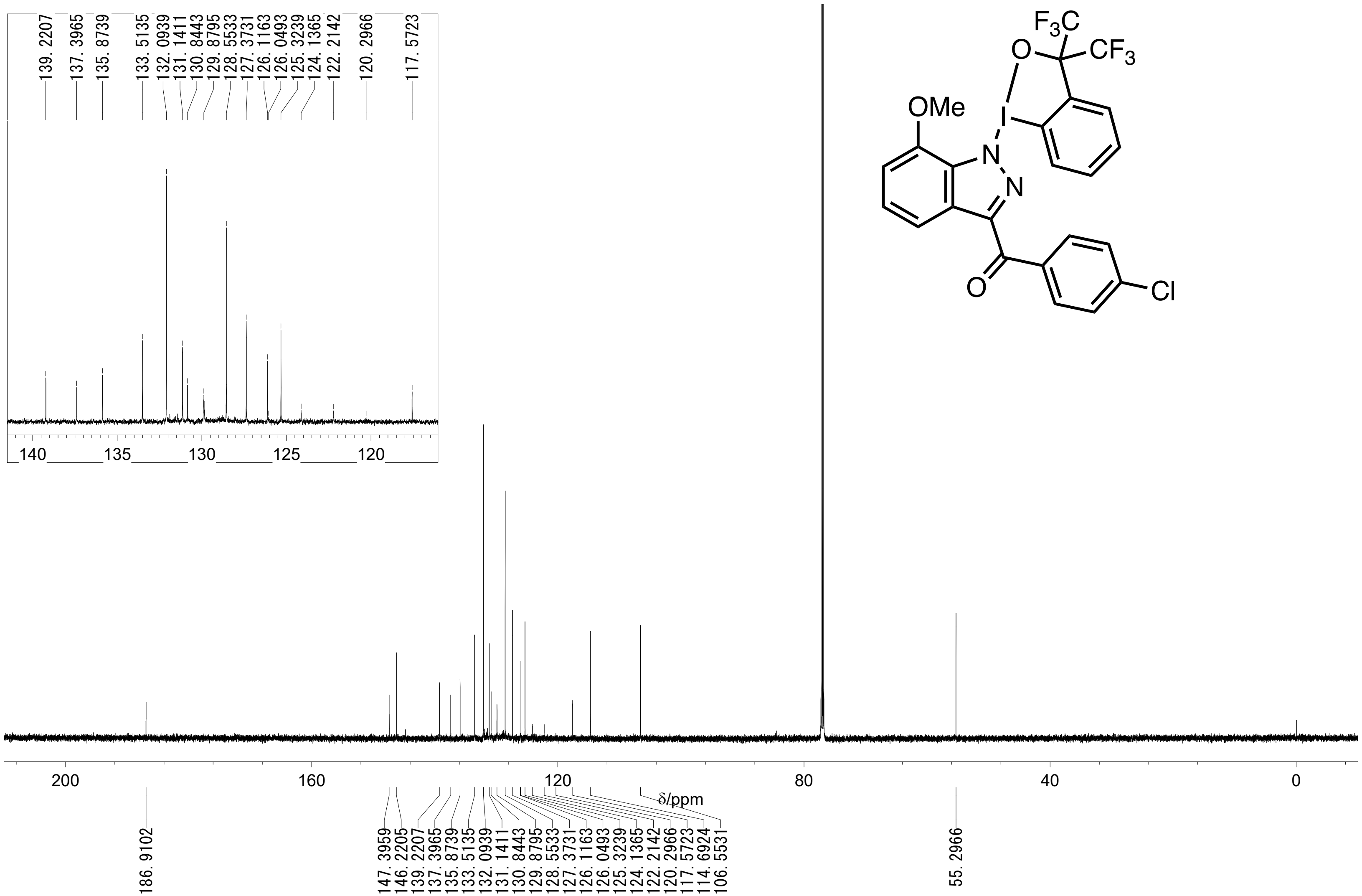

^{1}H NMR spectrum of **3ah** (400 MHz, $CDCl_3$)

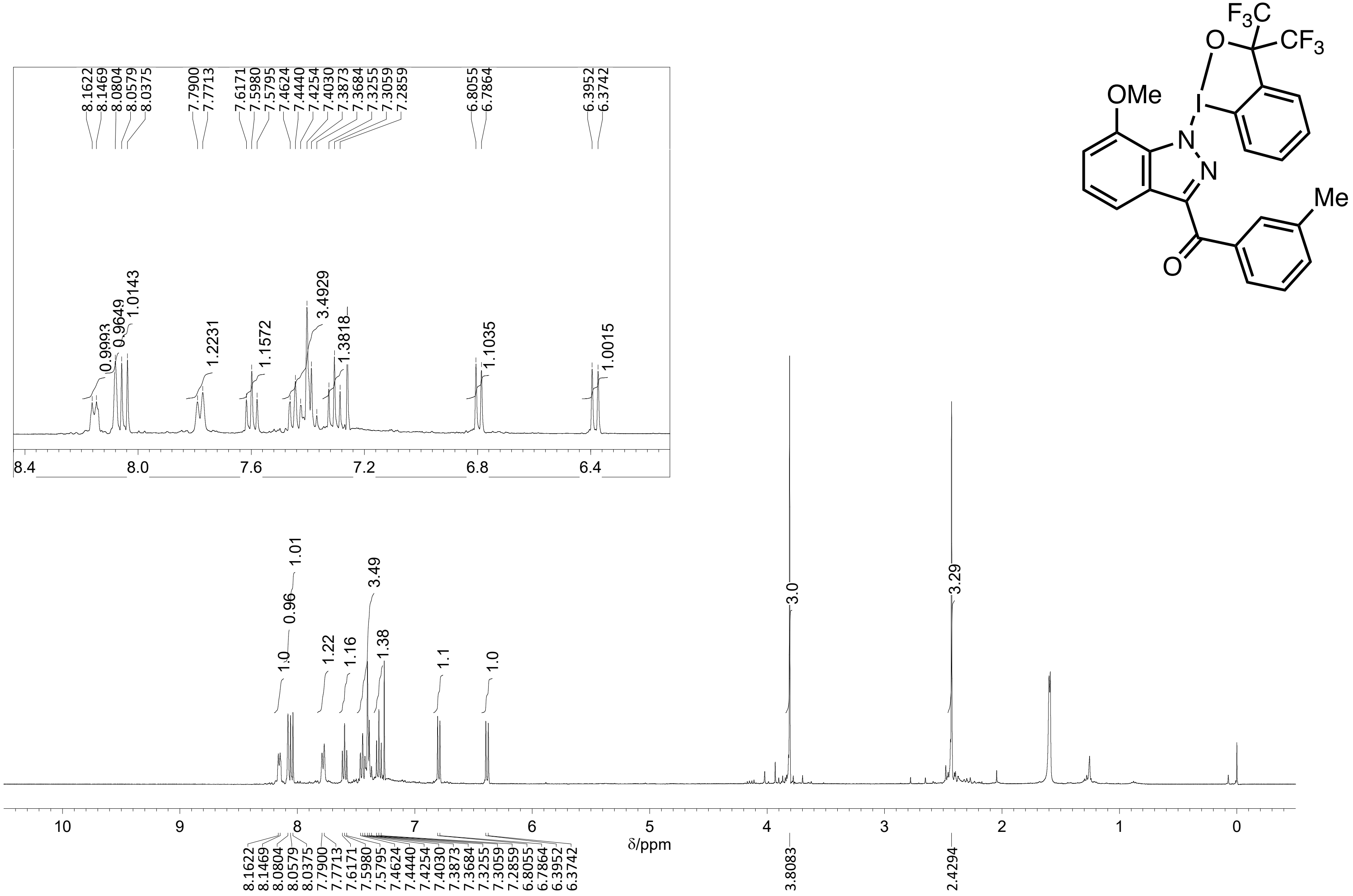

$^{13}C\{^{1}H\}$ NMR spectrum of **3ah** (150 MHz, $CDCl_3$)

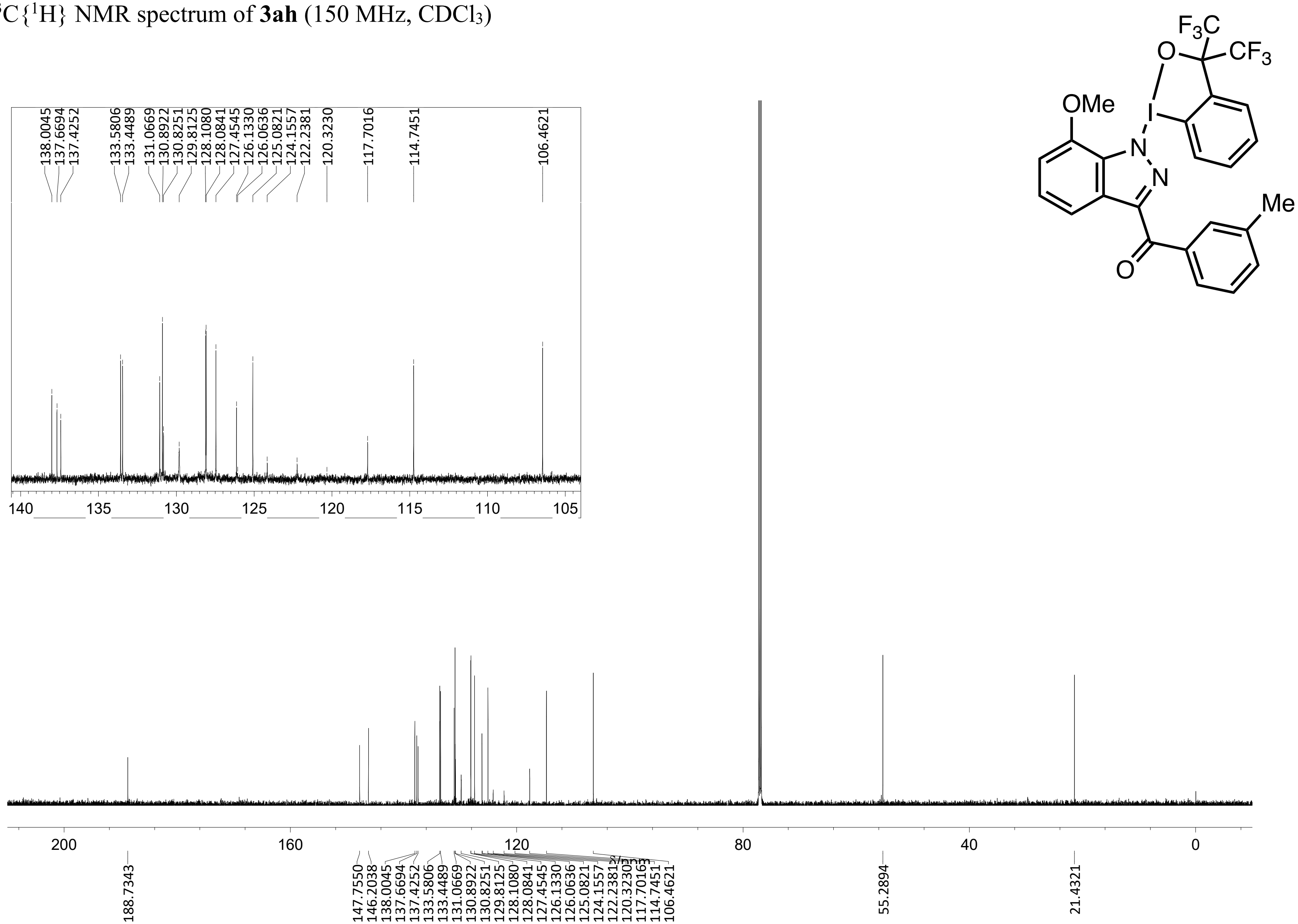

^{1}H NMR spectrum of **3aj** (600 MHz, $CDCl_3$)

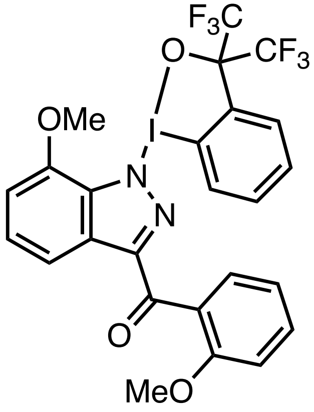

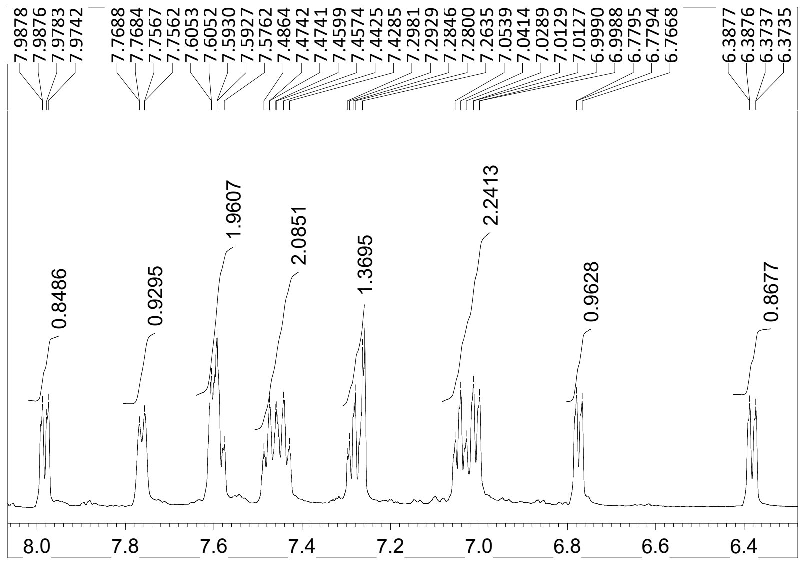

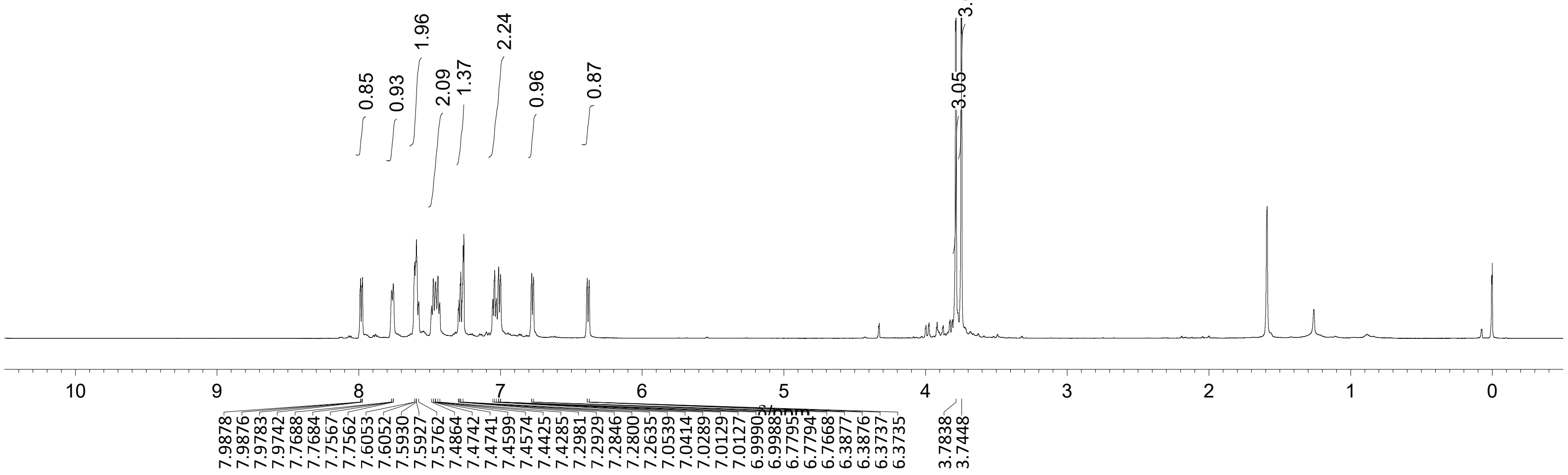

^{13}C{^{1}H} NMR spectrum of **3aj** (150 MHz, $CDCl_3$)

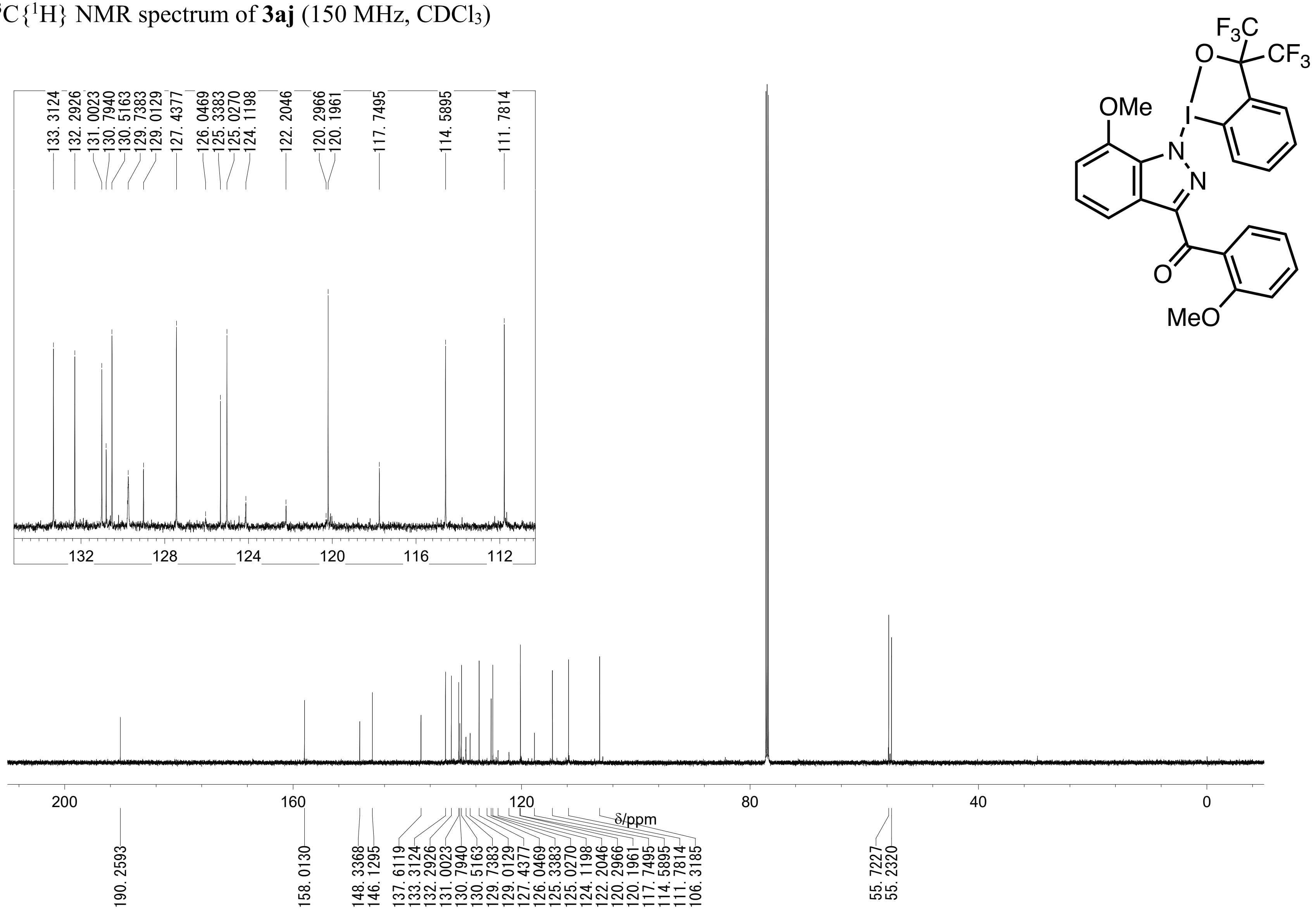

^{1}H NMR spectrum of **3ak** (400 MHz, $CDCl_3$)

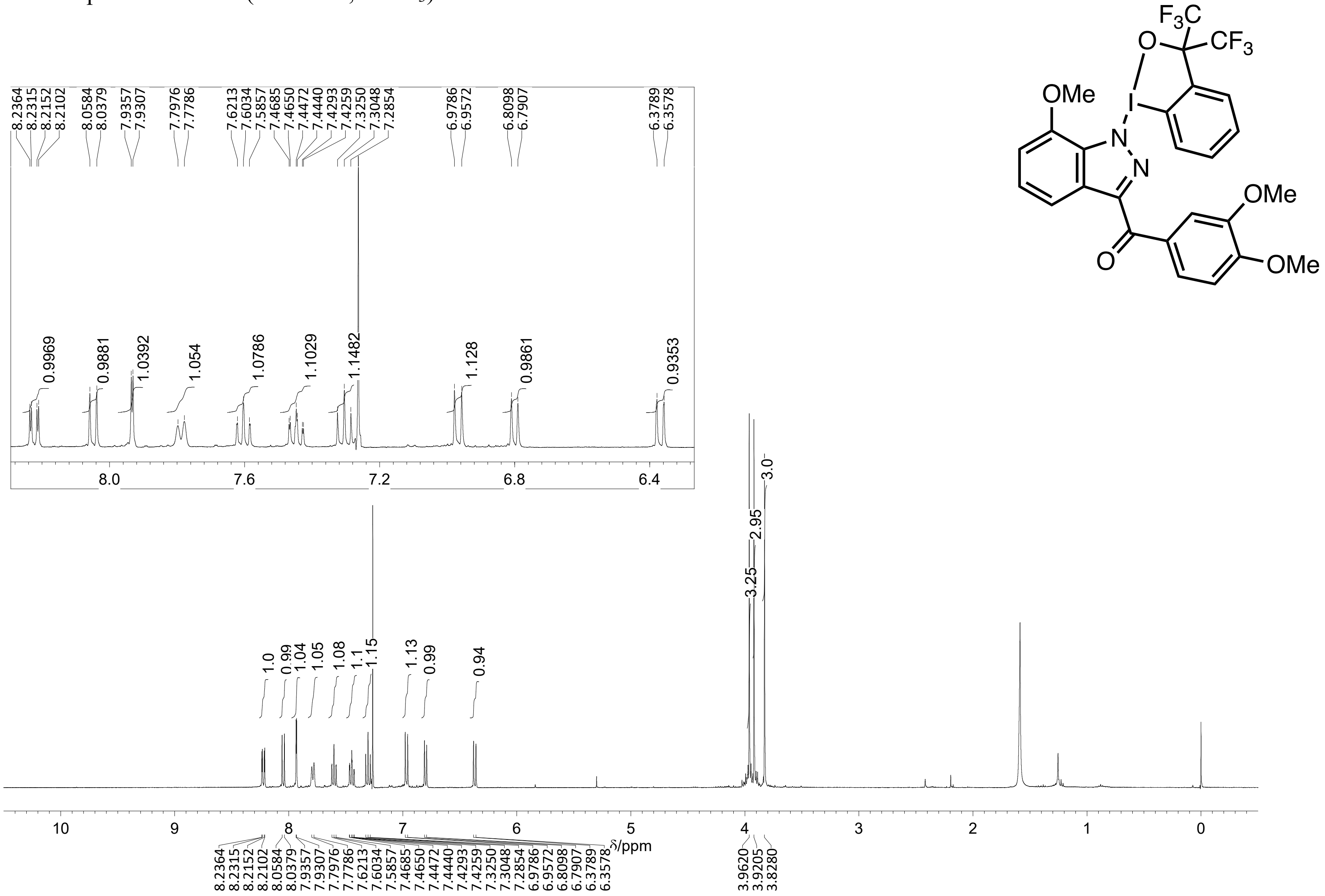

$^{13}C\{^1H\}$ NMR spectrum of **3ak** (150 MHz, $CDCl_3$)

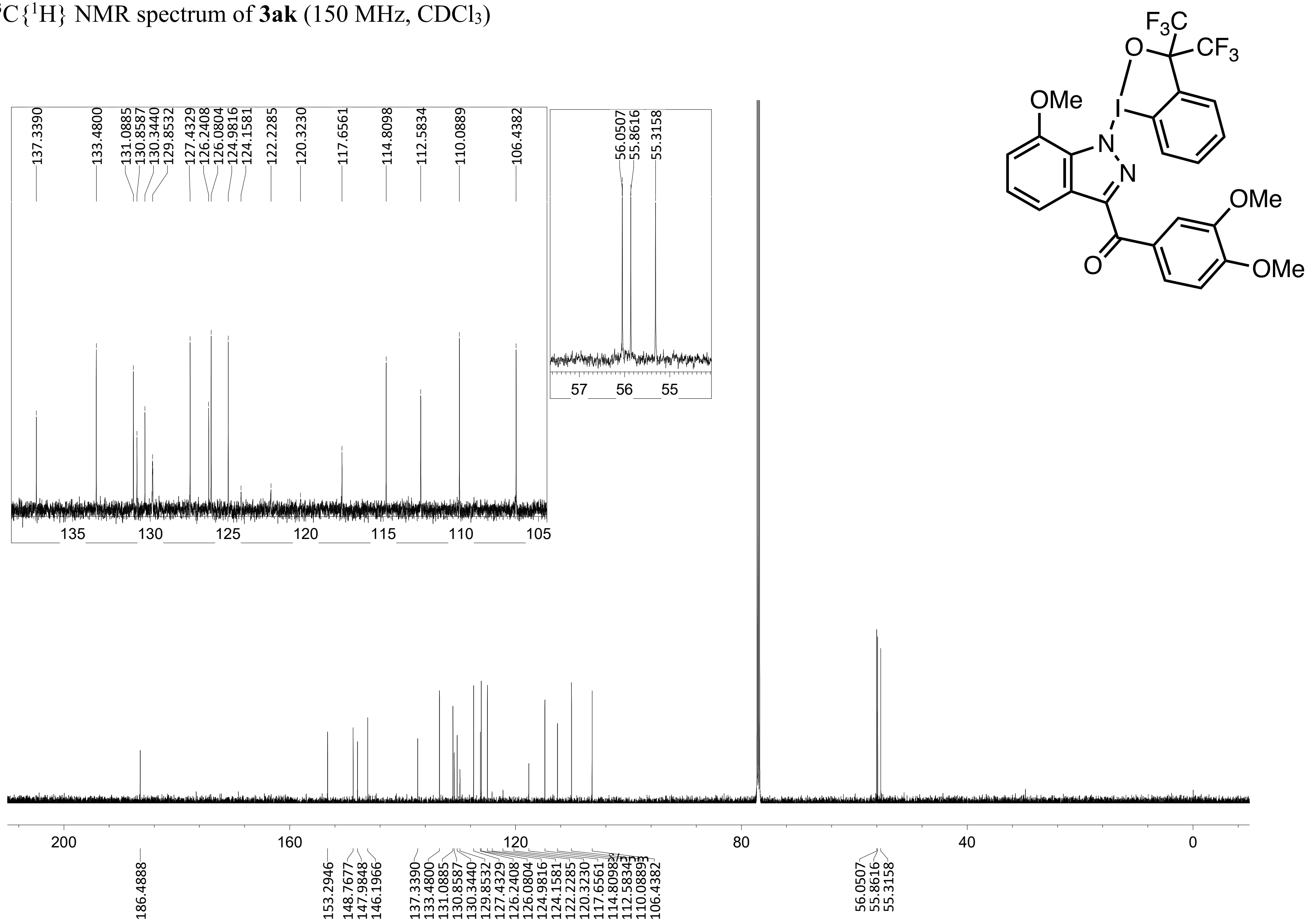

^{1}H NMR spectrum of **3ba** (400 MHz, $CDCl_3$)

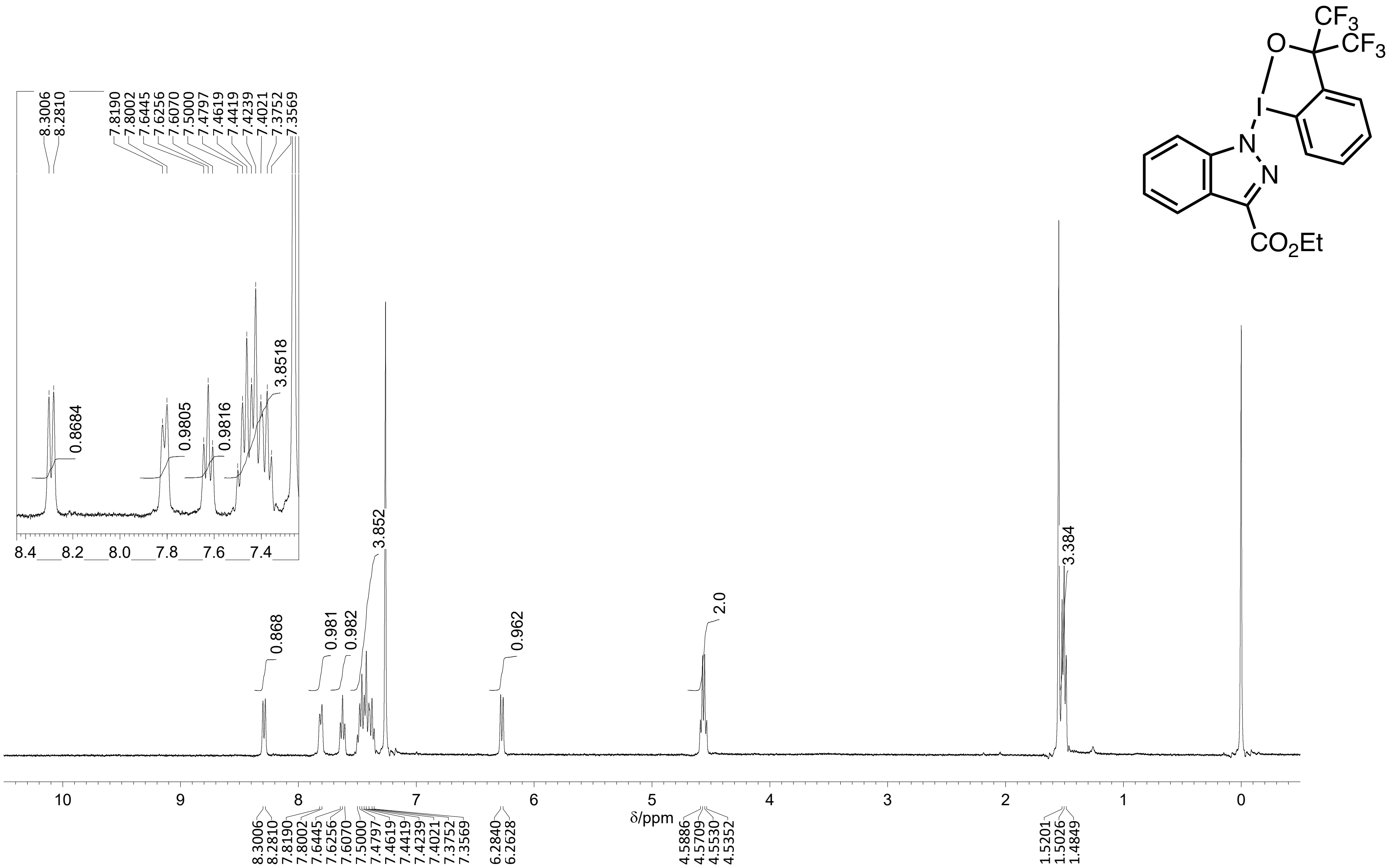

$^{13}C\{^1H\}$ NMR spectrum of **3ba** (150 MHz, $CDCl_3$)

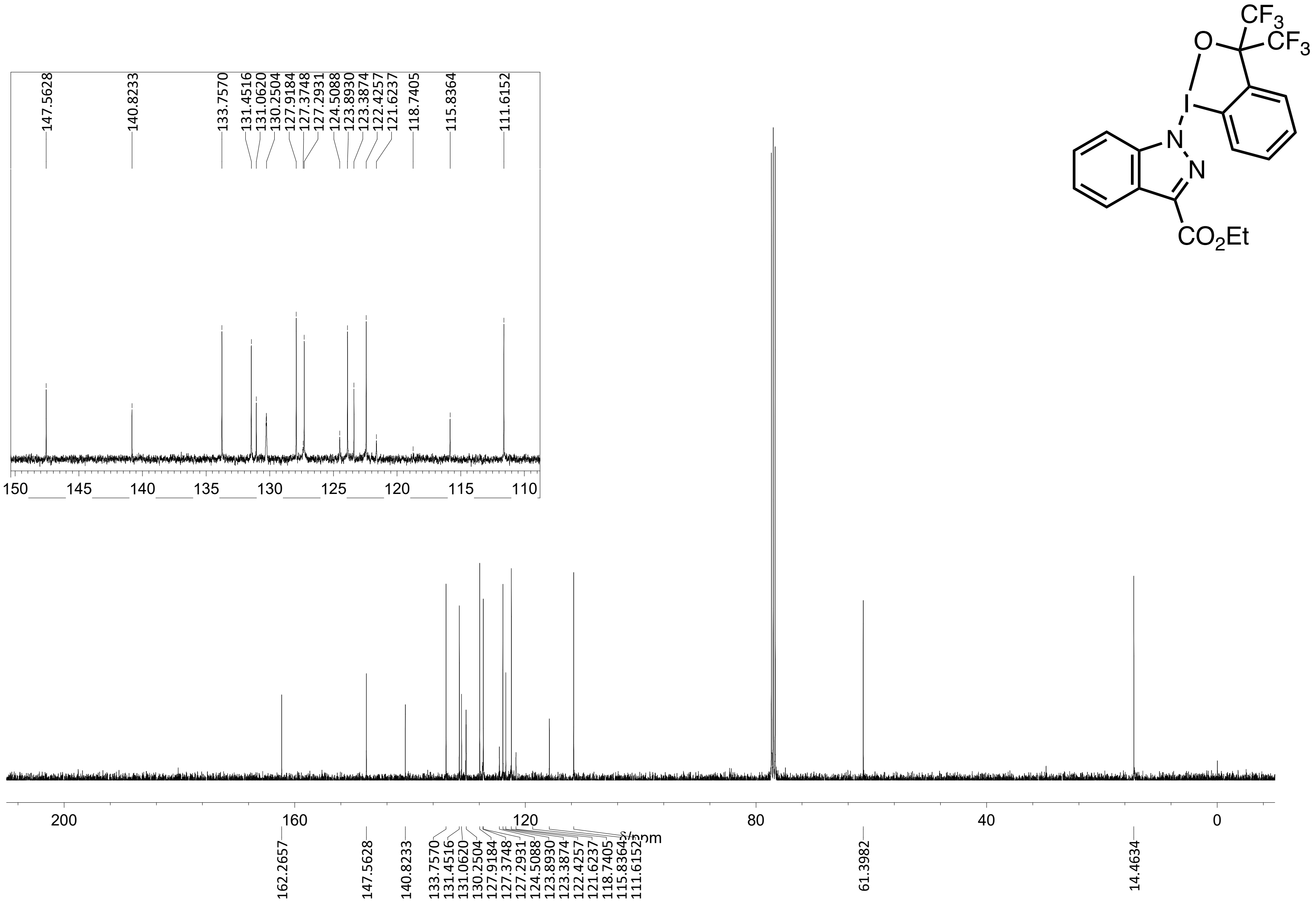

^{1}H NMR spectrum of **3ca** (400 MHz, $CDCl_3$)

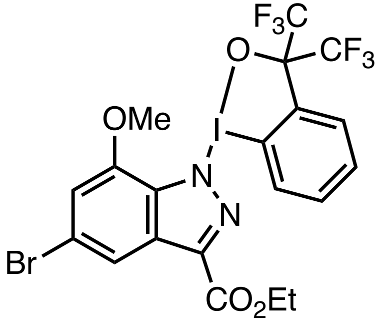

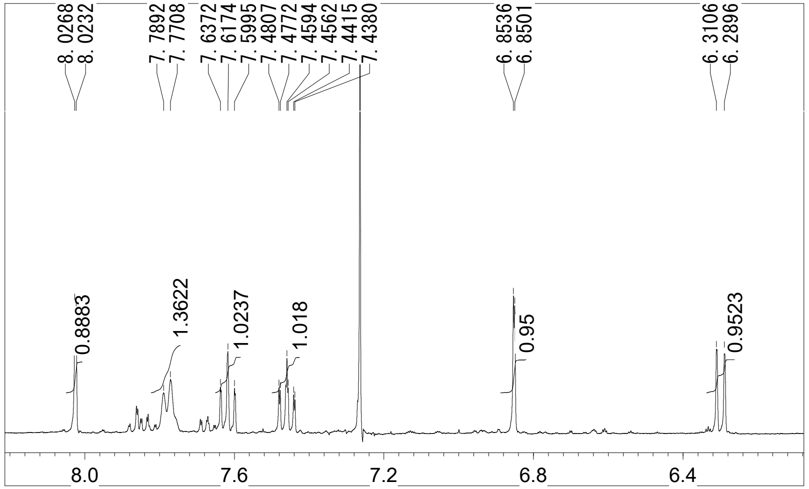

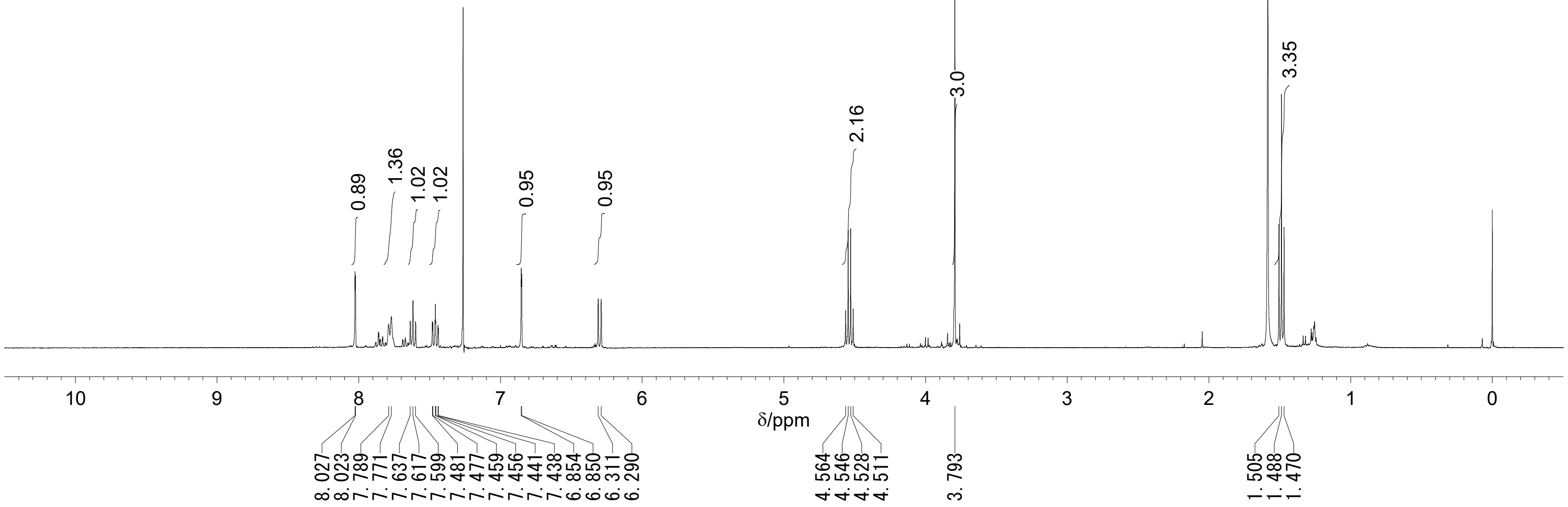

$^{13}C\{^{1}H\}$ NMR spectrum of **3ca** (150 MHz, $CDCl_3$)

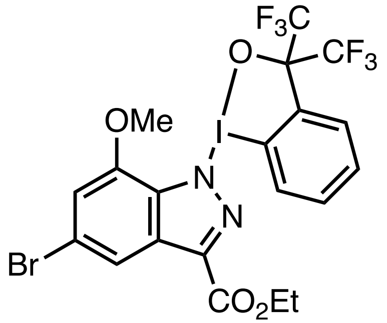

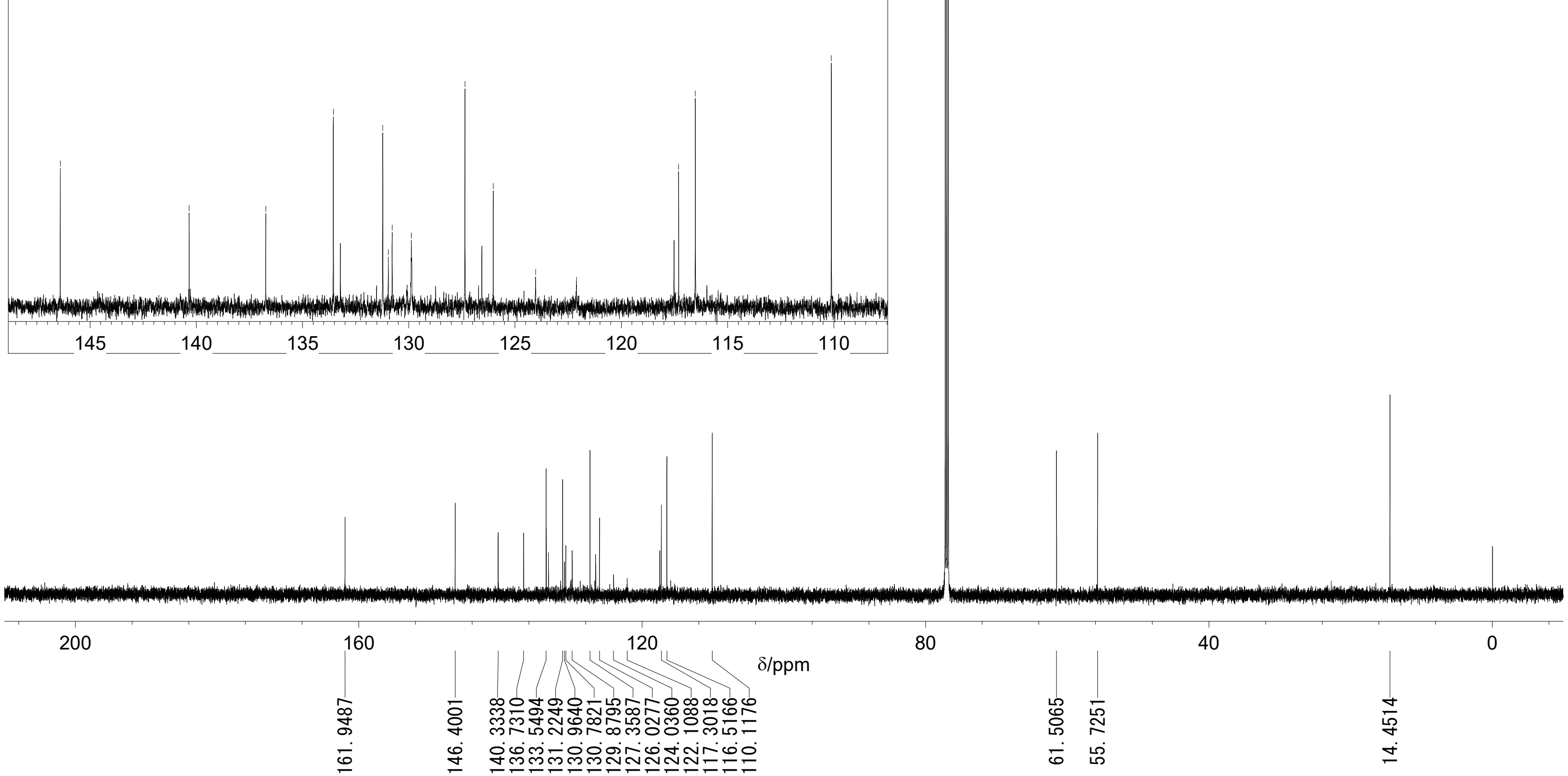

^{1}H NMR spectrum of **3da** (600 MHz, $CDCl_3$)

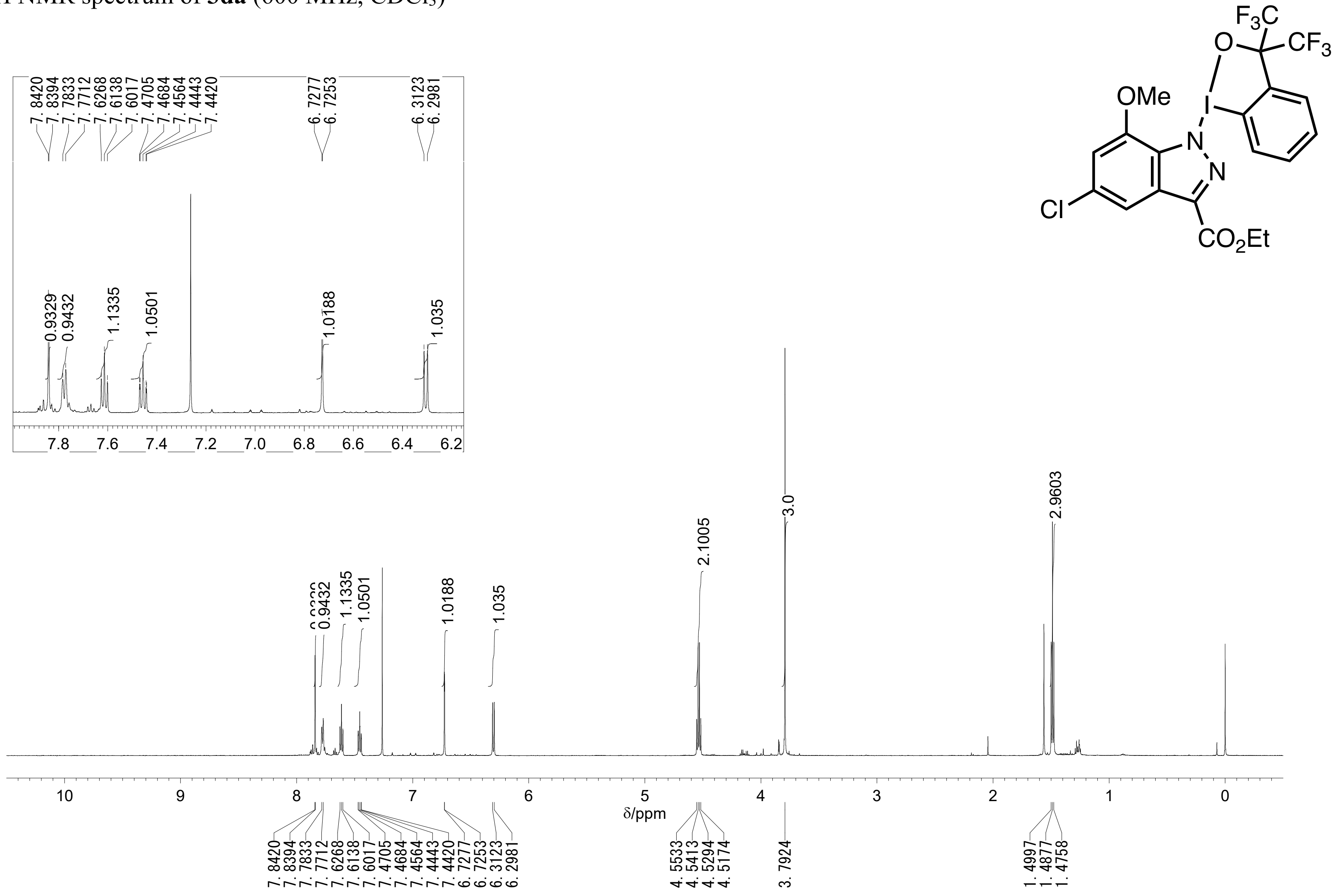

$^{13}C\{^1H\}$ NMR spectrum of **3da** (150 MHz, $CDCl_3$)

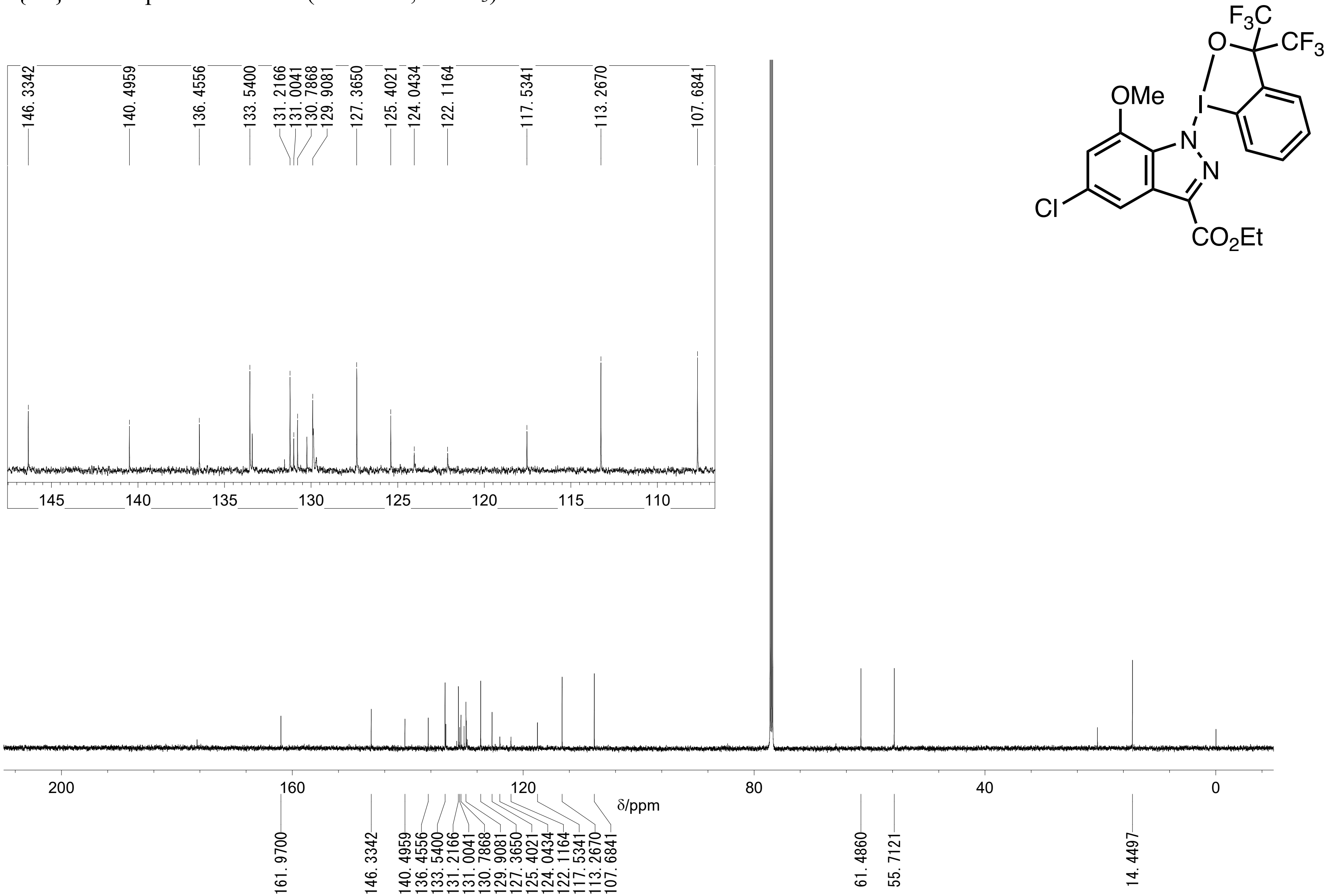

^{1}H NMR spectrum of **3ea** (400 MHz, $CDCl_3$)

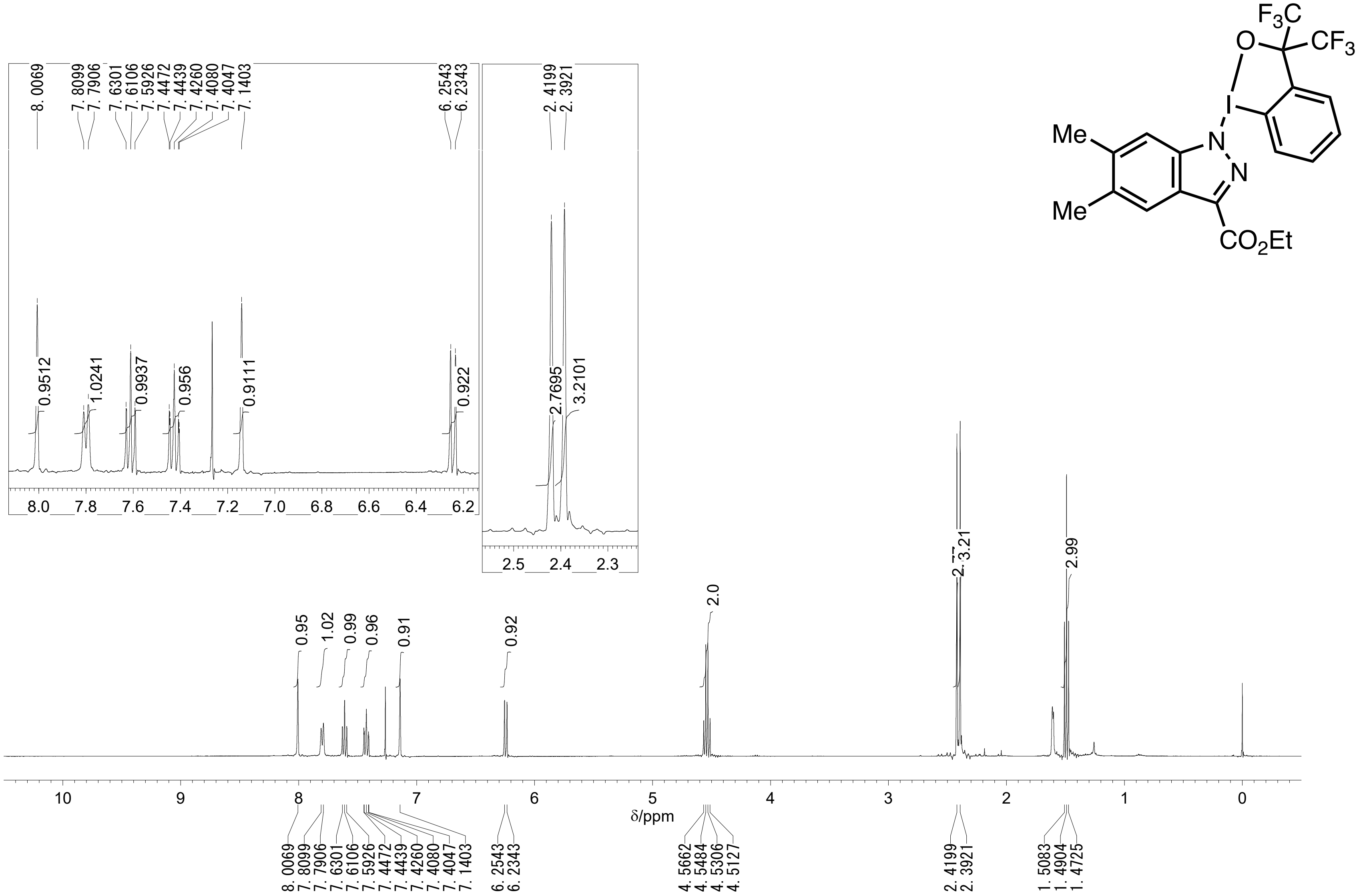

$^{13}C\{^1H\}$ NMR spectrum of **3ea** (150 MHz, $CDCl_3$)

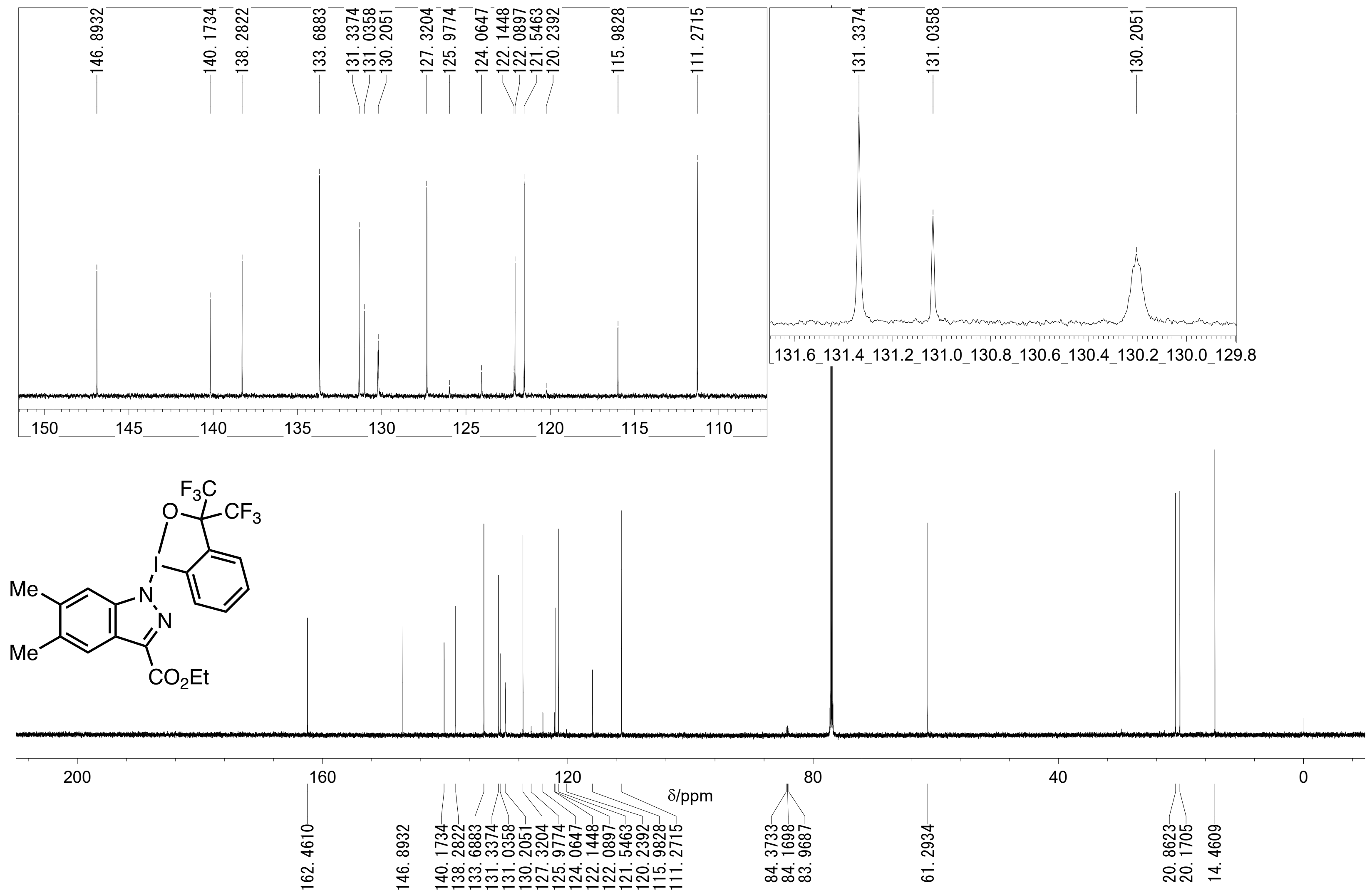

^{1}H NMR spectrum of **3fa** (400 MHz, $CDCl_3$)

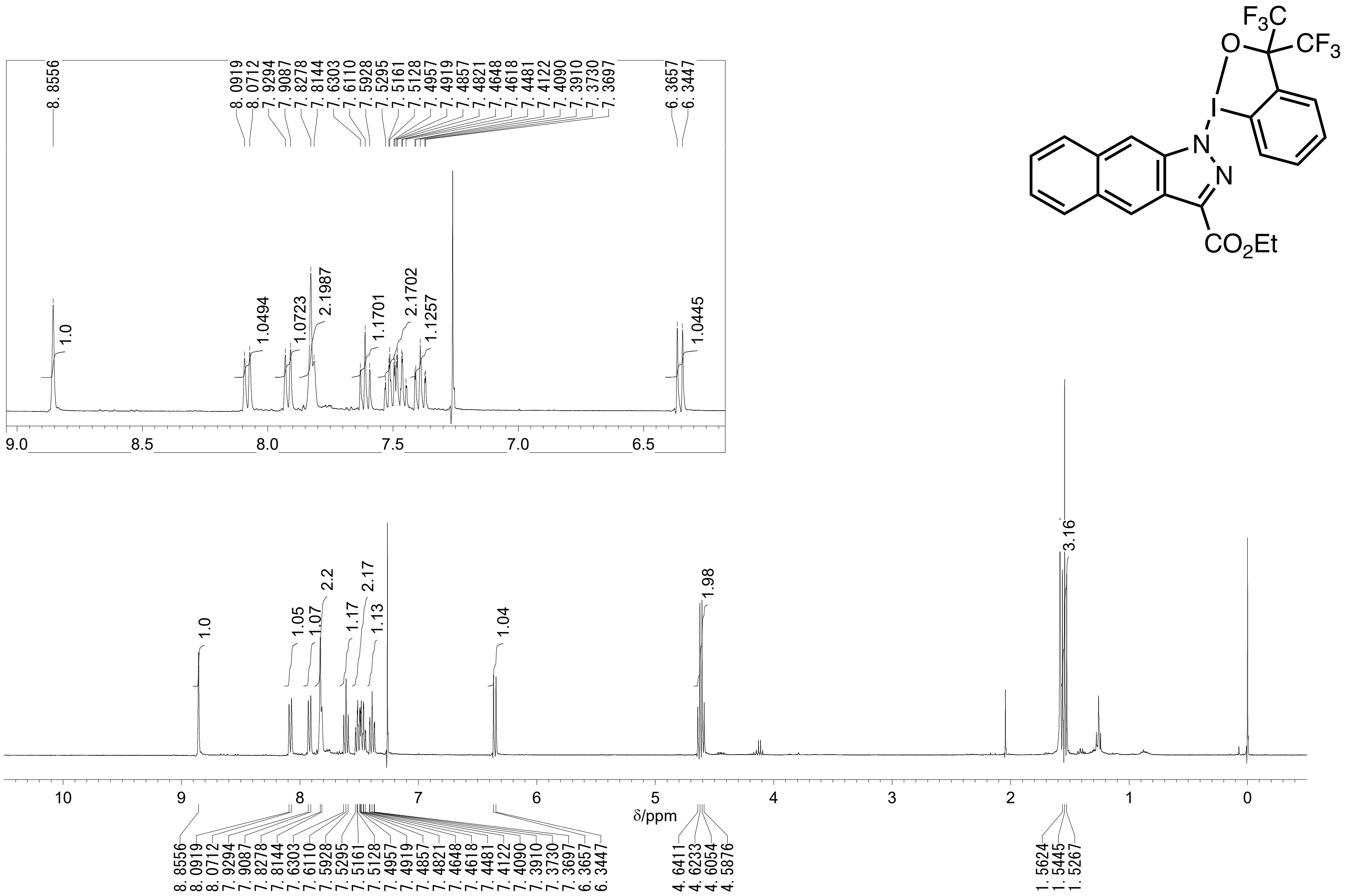

$^{13}C\{^{1}H\}$ NMR spectrum of **3fa** (150 MHz, $CDCl_3$)

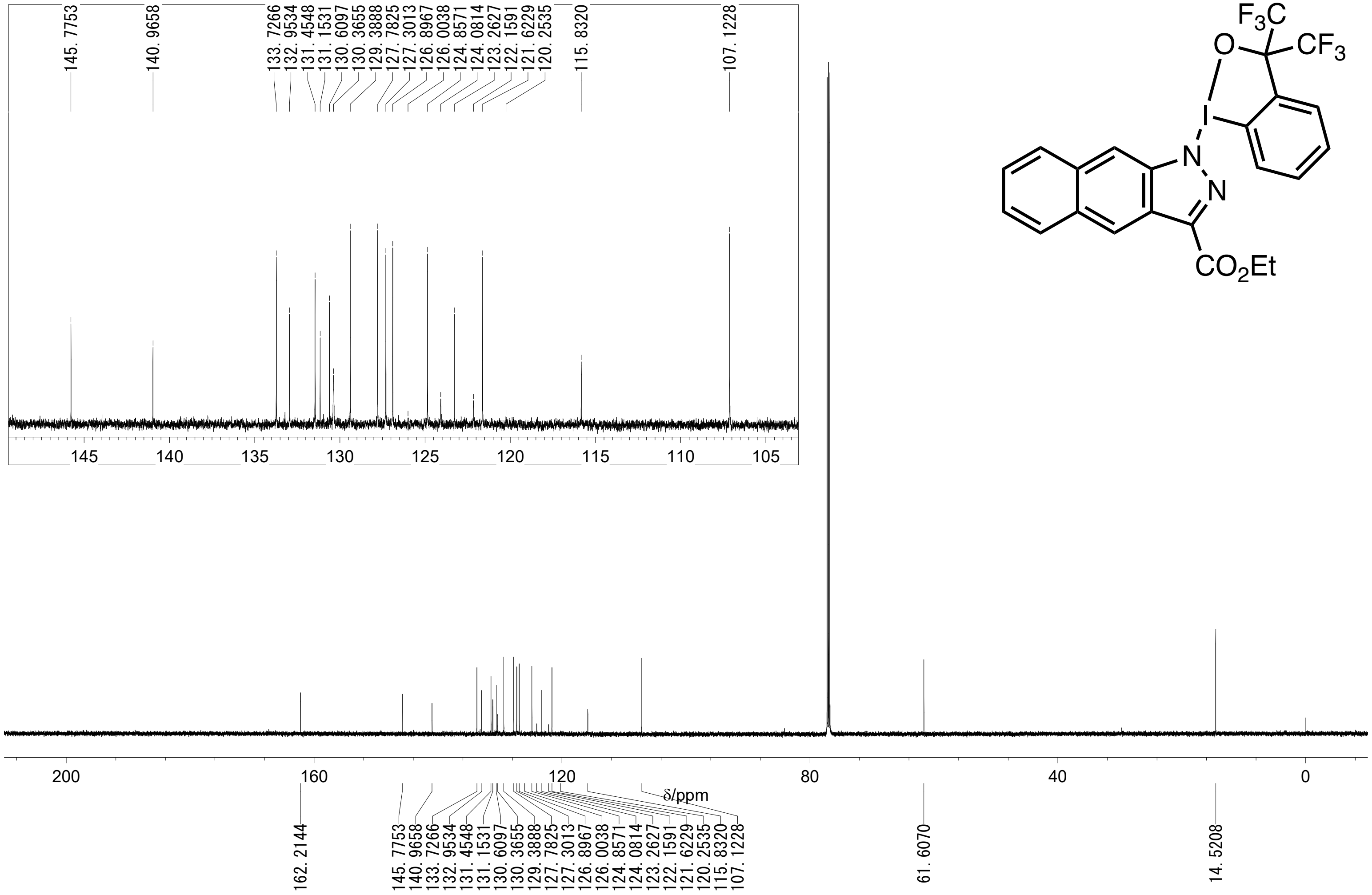

^{1}H NMR spectrum of **3ga** (400 MHz, $CDCl_3$)

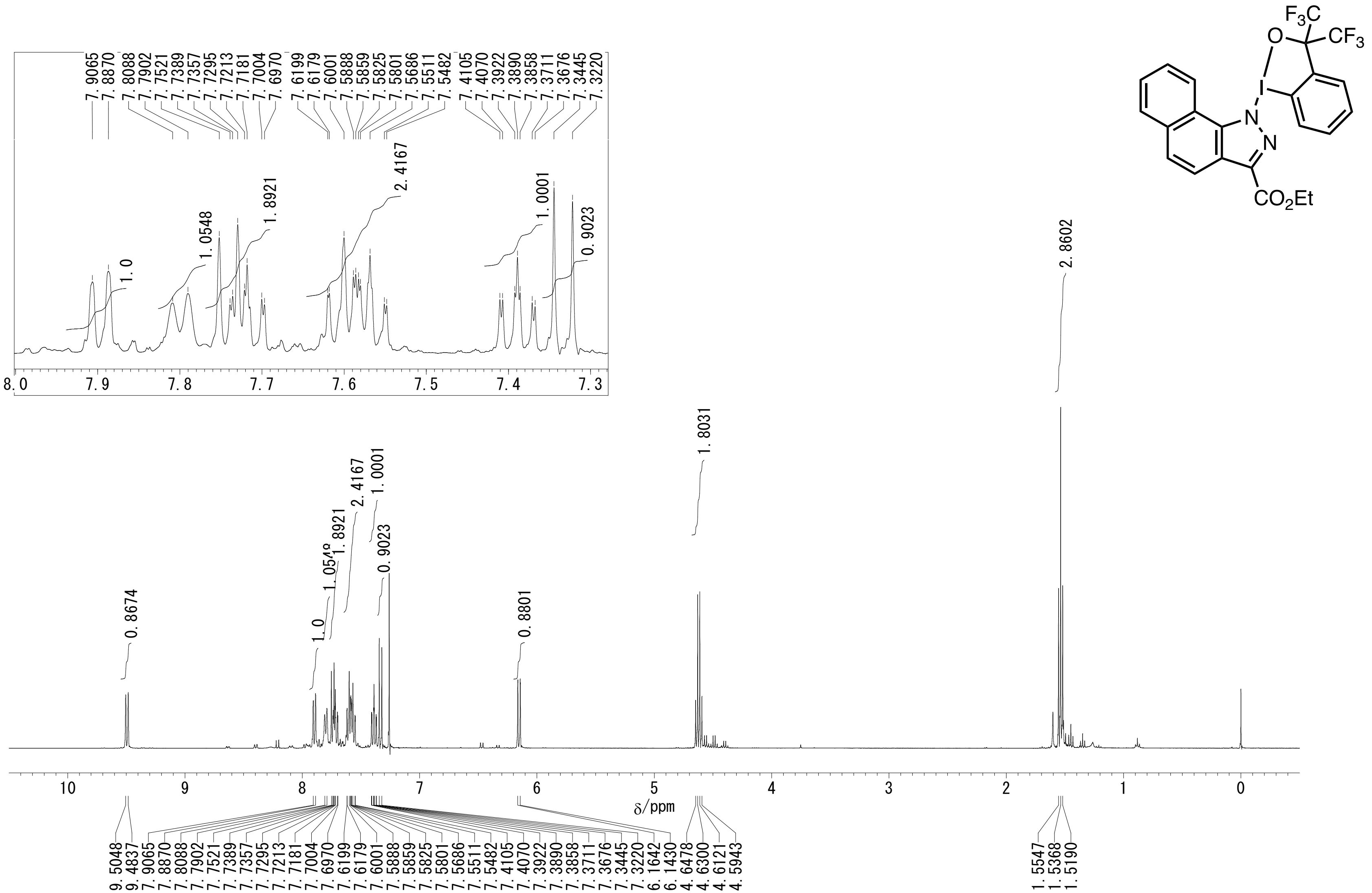

$^{13}C\{^{1}H\}$ NMR spectrum of **3ga** (150 MHz, $CDCl_3$)

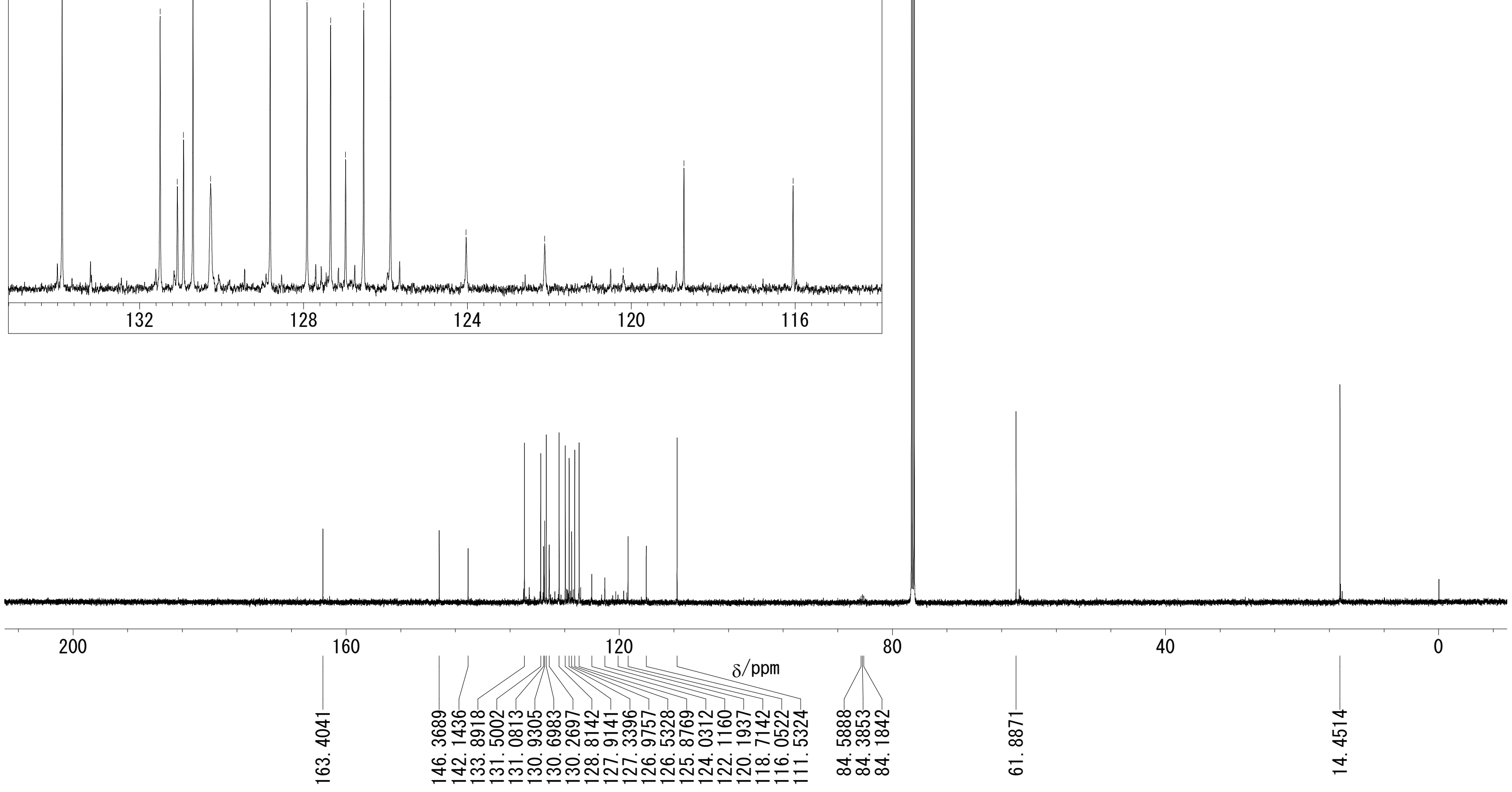

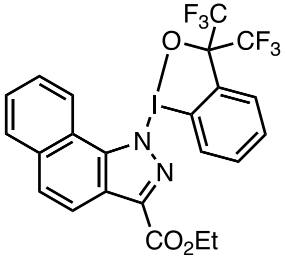

^{1}H NMR spectrum of **3ha** and **3ha’** (400 MHz, $CDCl_3$)

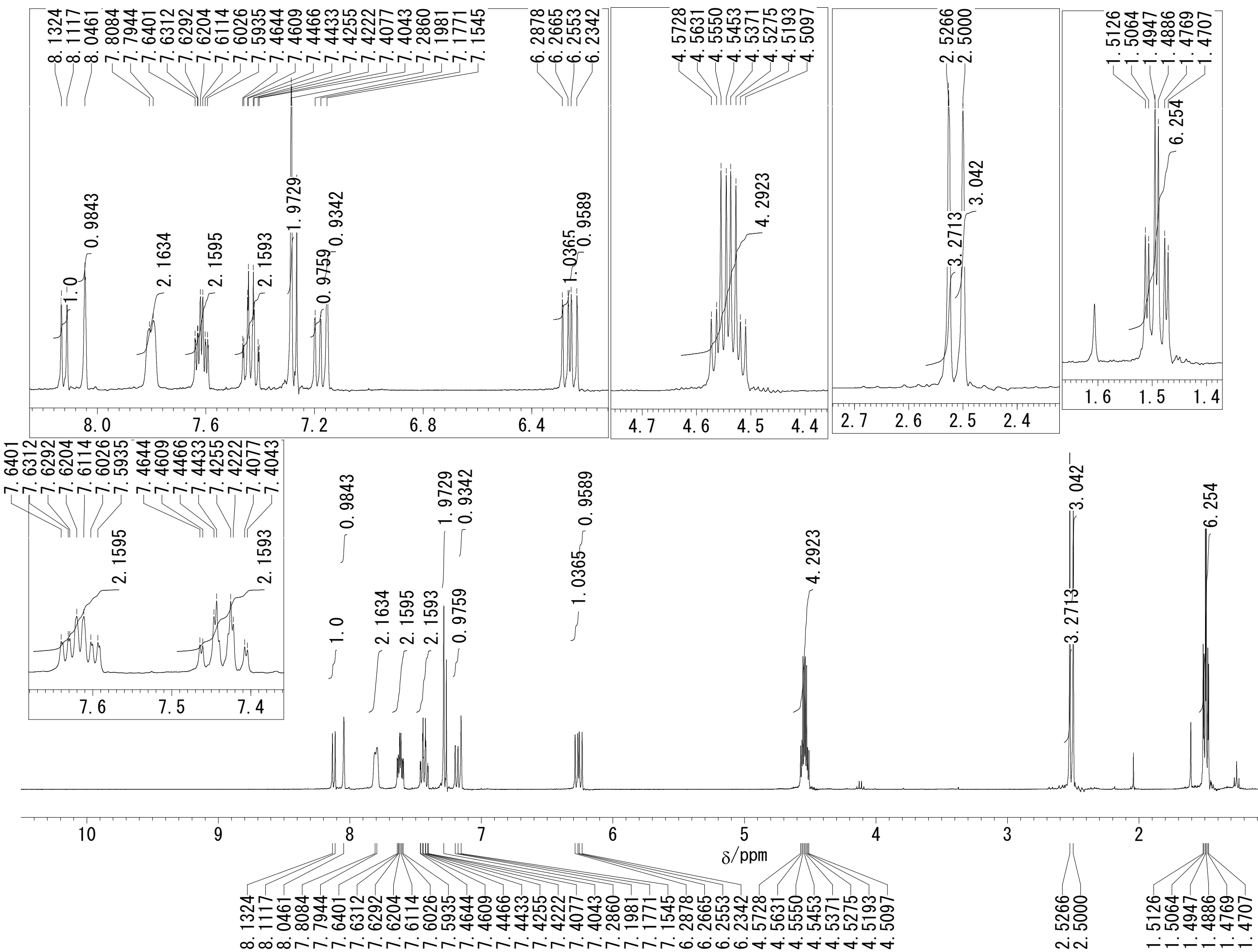

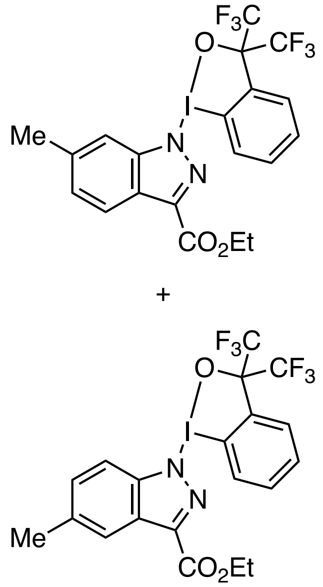

$^{13}C\{^1H\}$ NMR spectrum of **3ha** and **3ha'** (150 MHz, $CDCl_3$)

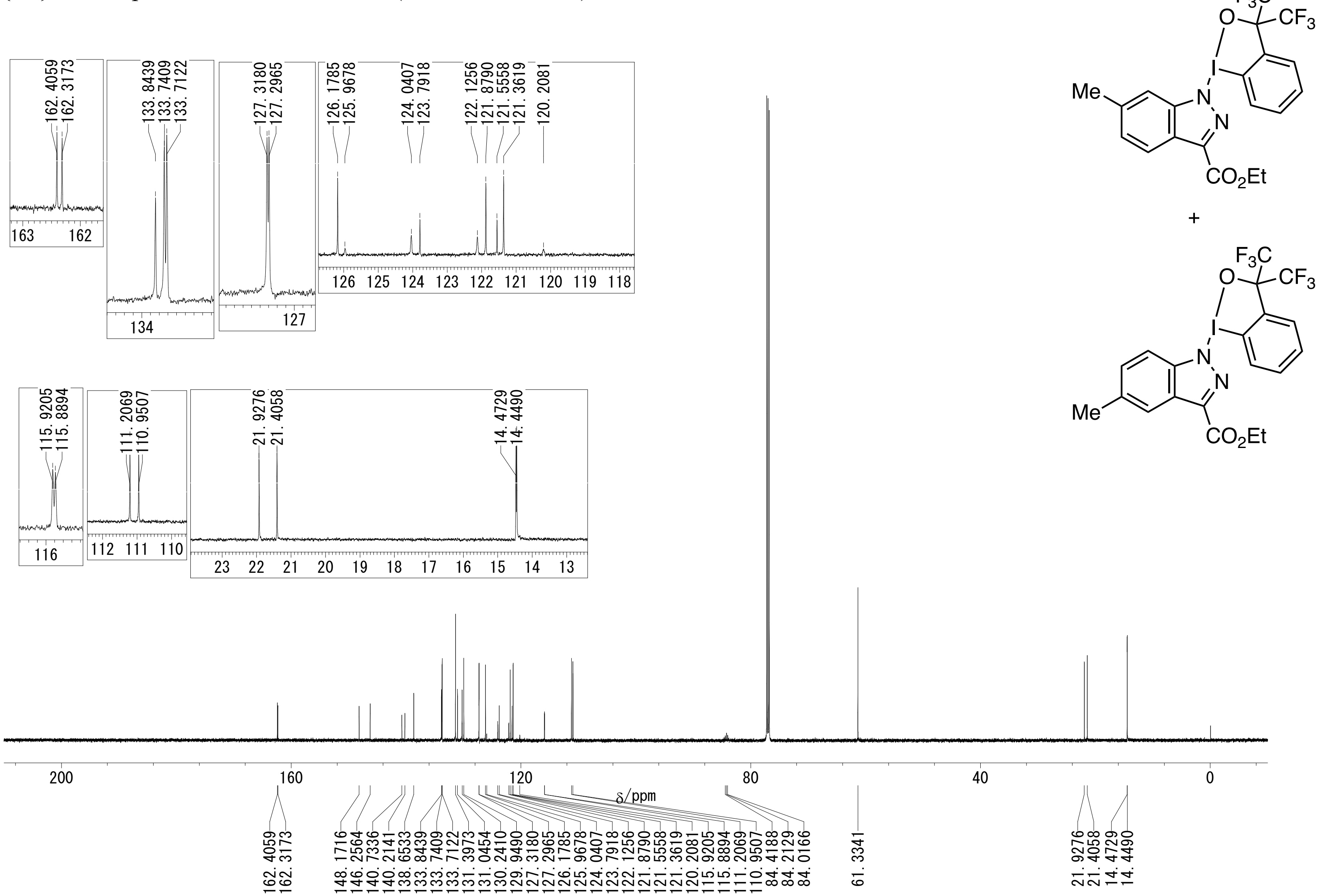

^{1}H NMR spectrum of **3ja** (400 MHz, $CDCl_3$)

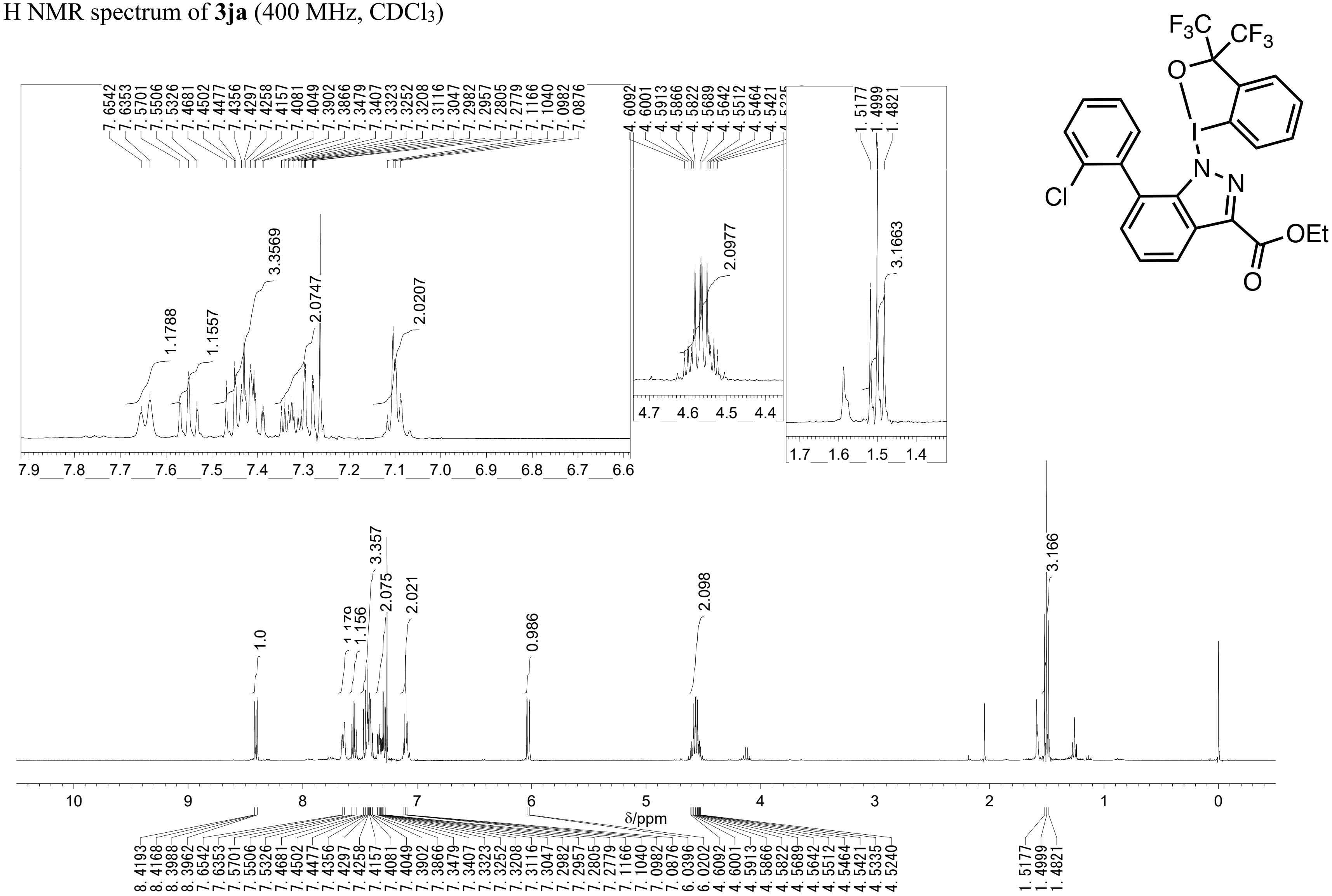

$^{13}C\{^{1}H\}$ NMR spectrum of **3ja** (150 MHz, $CDCl_3$)

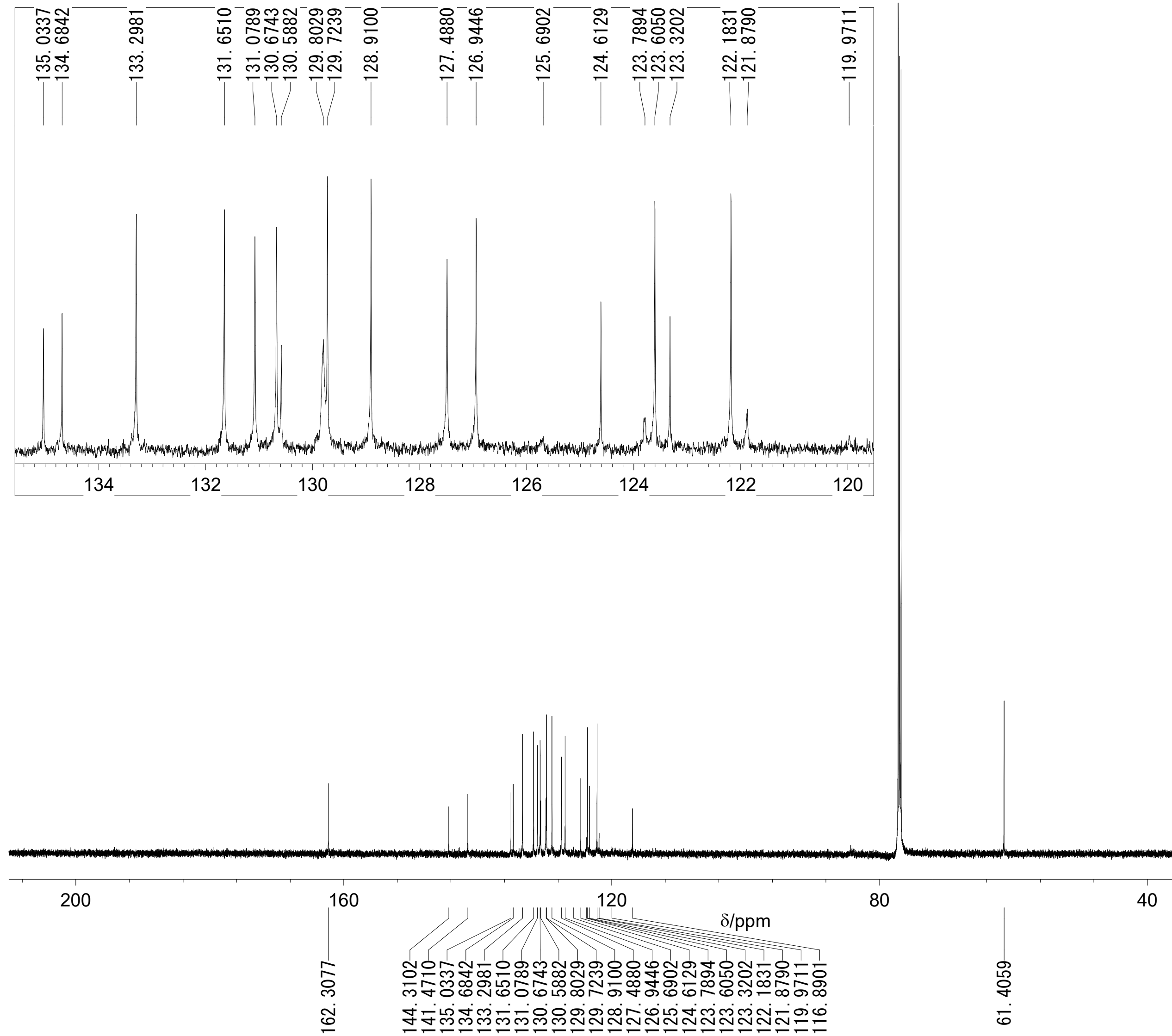

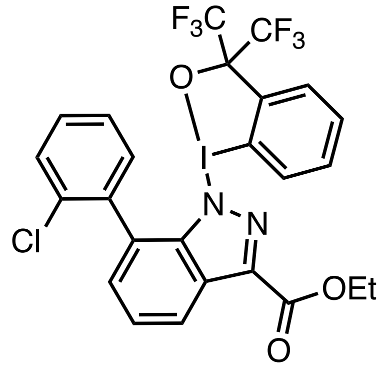

HMQC (left) and HMBC (right) spectra of **3ja**

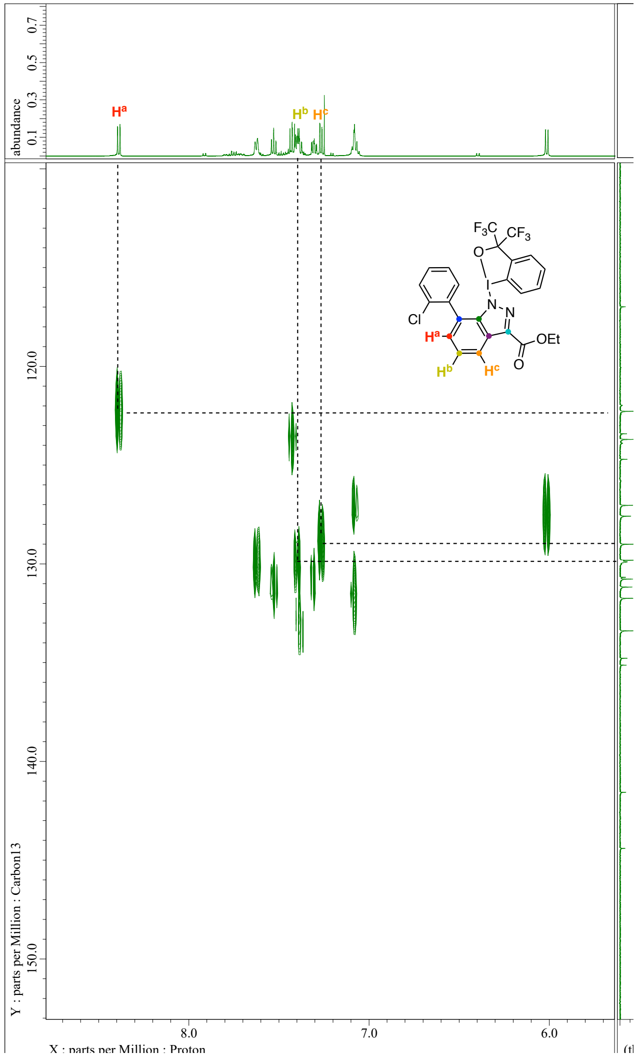

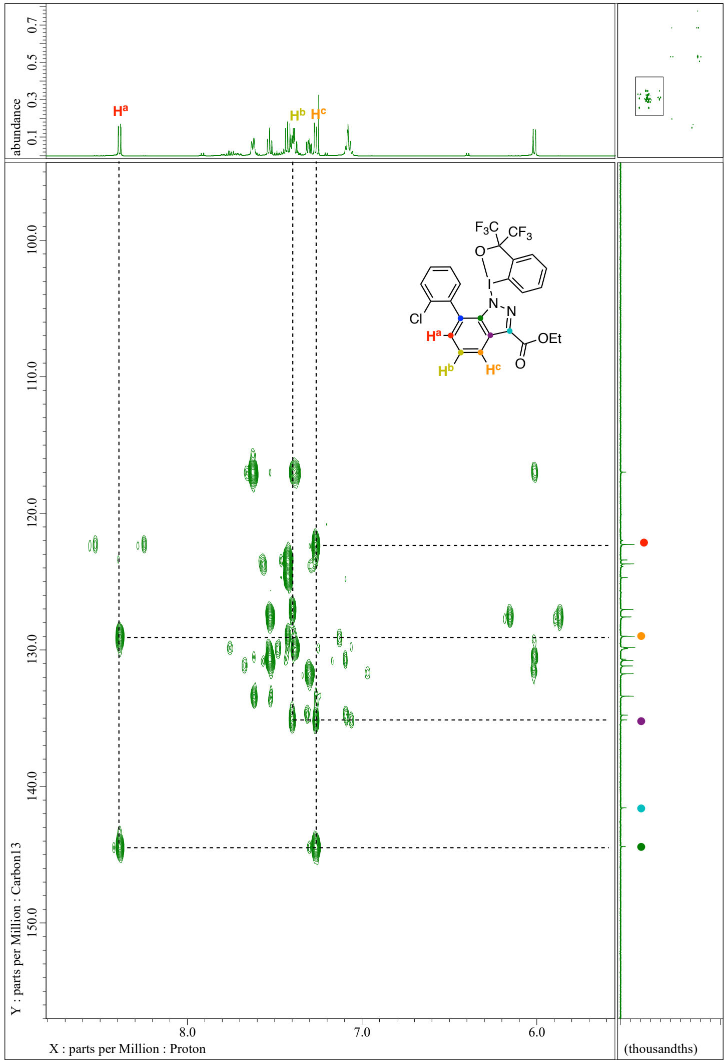

^{1}H NMR spectrum of **3ka** (600 MHz, $CDCl_3$)

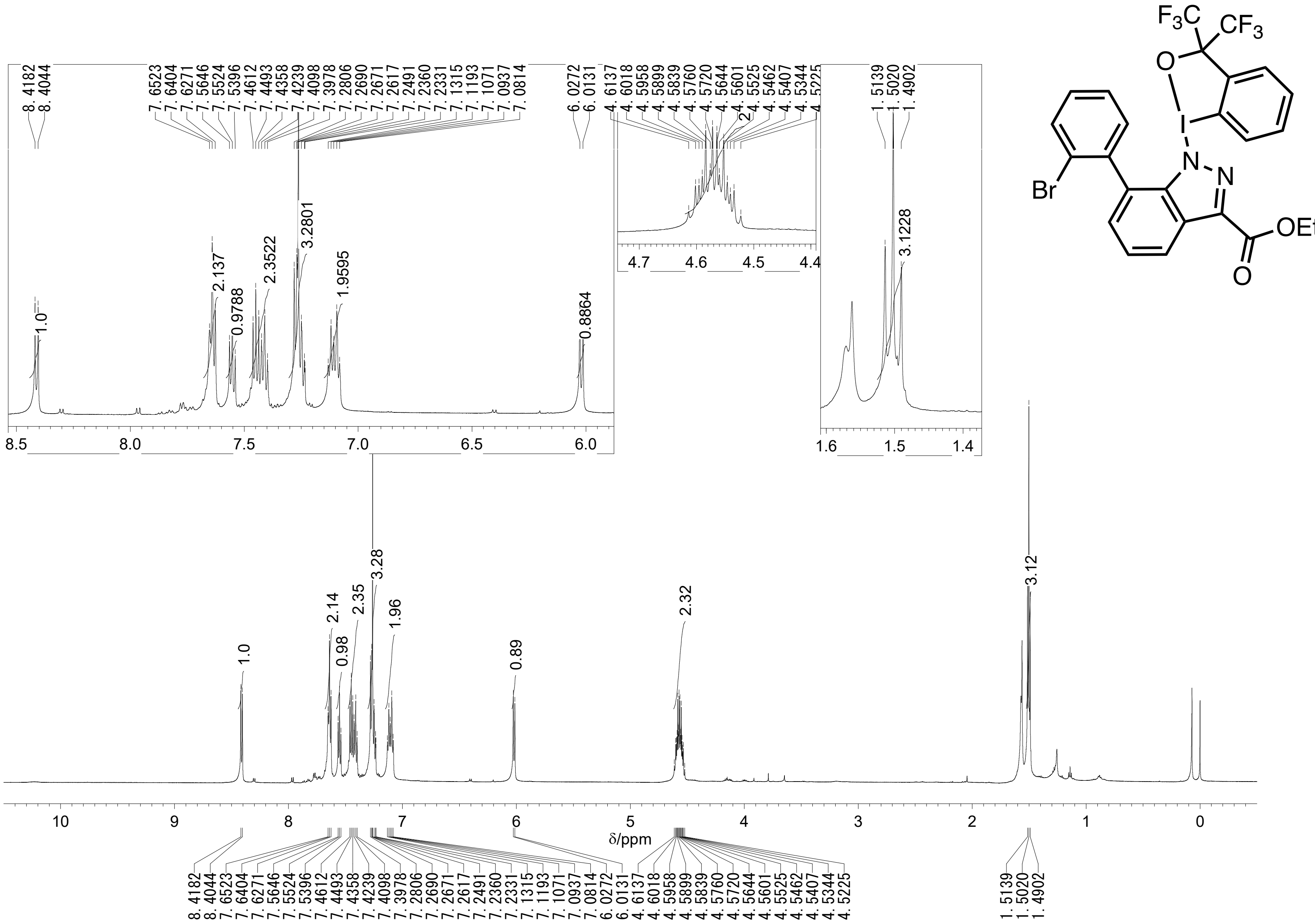

$^{13}C\{^1H\}$ NMR spectrum of **3ka** (150 MHz, $CDCl_3$)

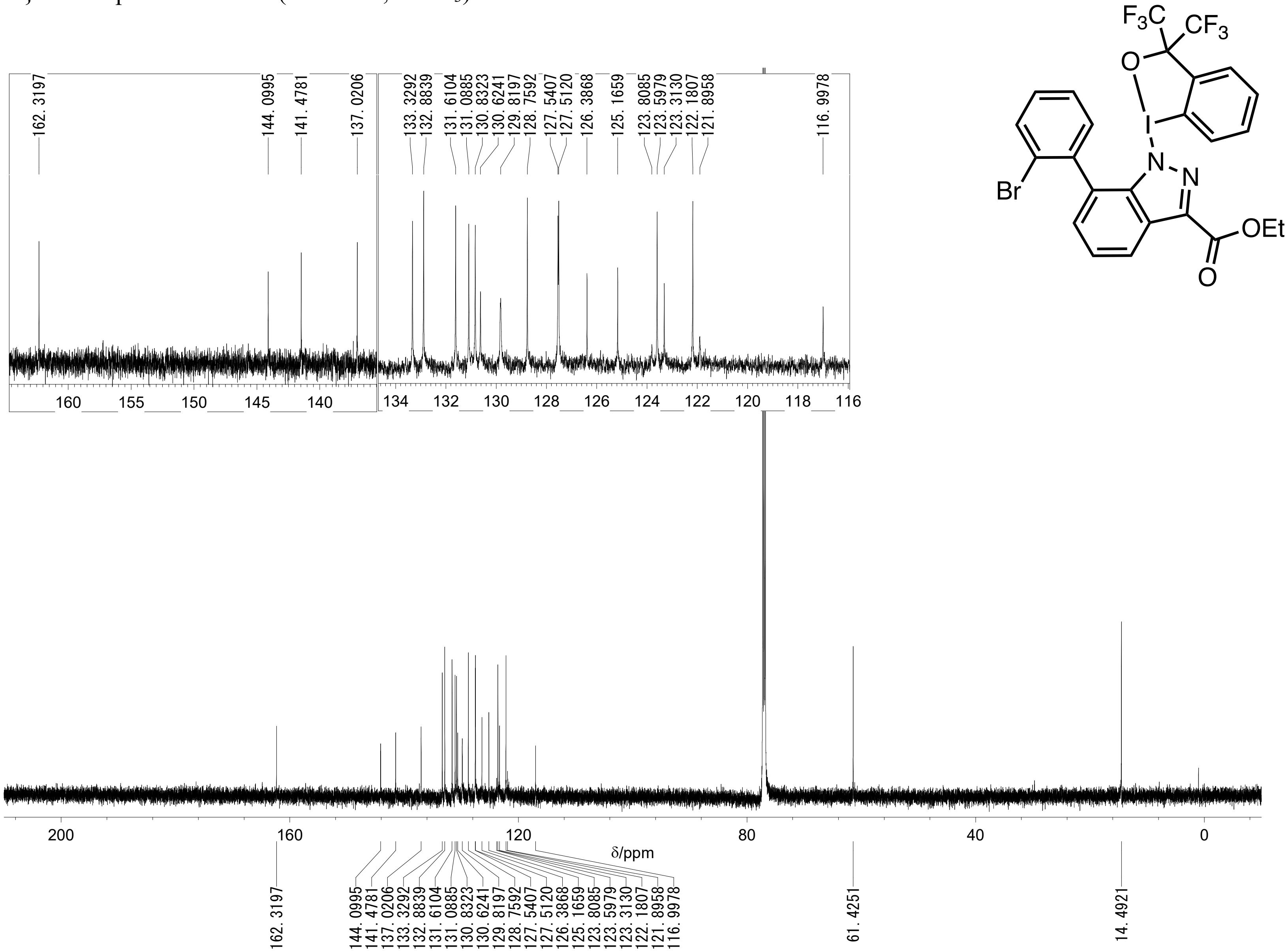

^{1}H NMR spectrum of **3jd** (400 MHz, $CDCl_3$)

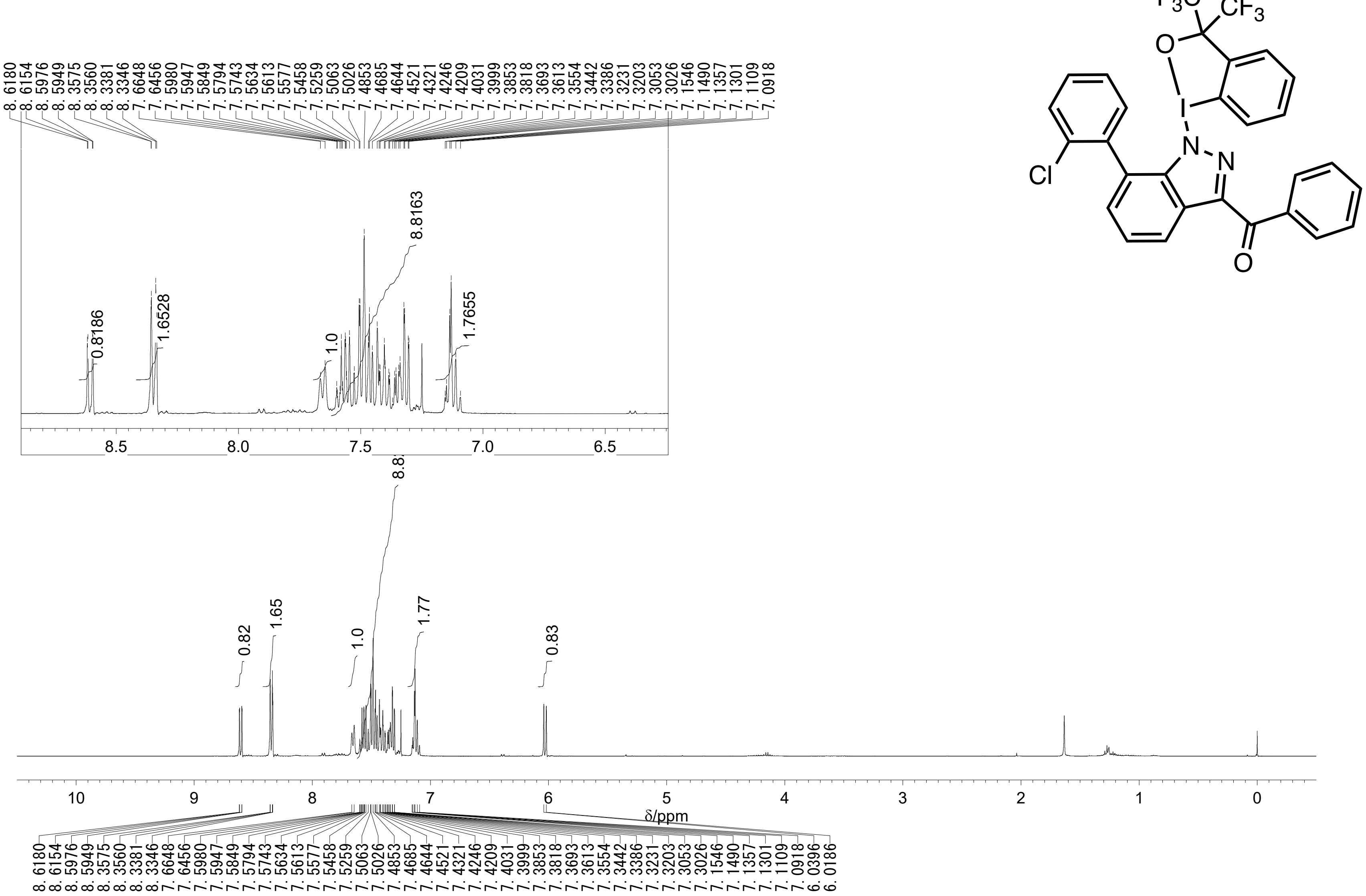

$^{13}C\{^{1}H\}$ NMR spectrum of **3jd** (150 MHz, $CDCl_3$)

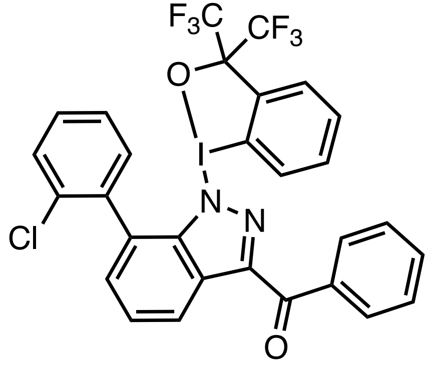

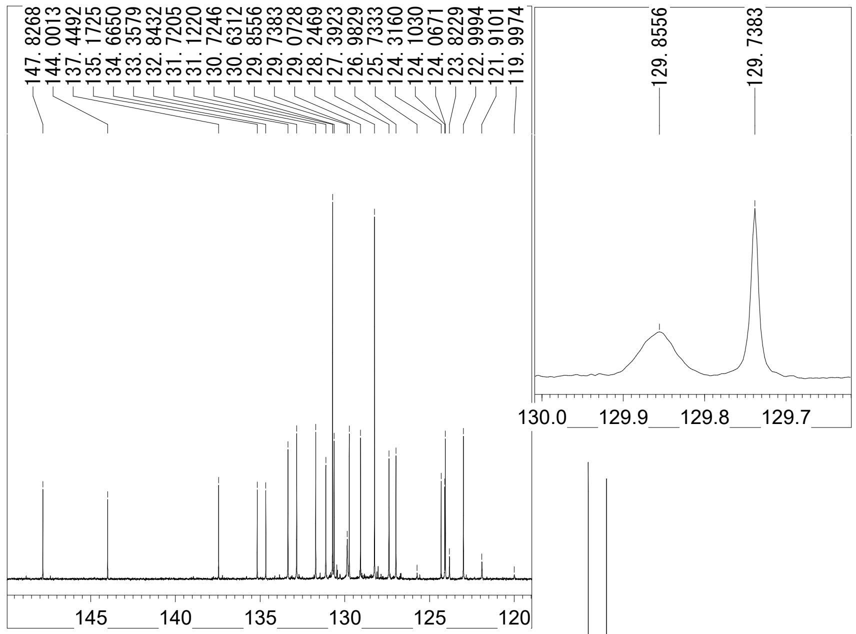

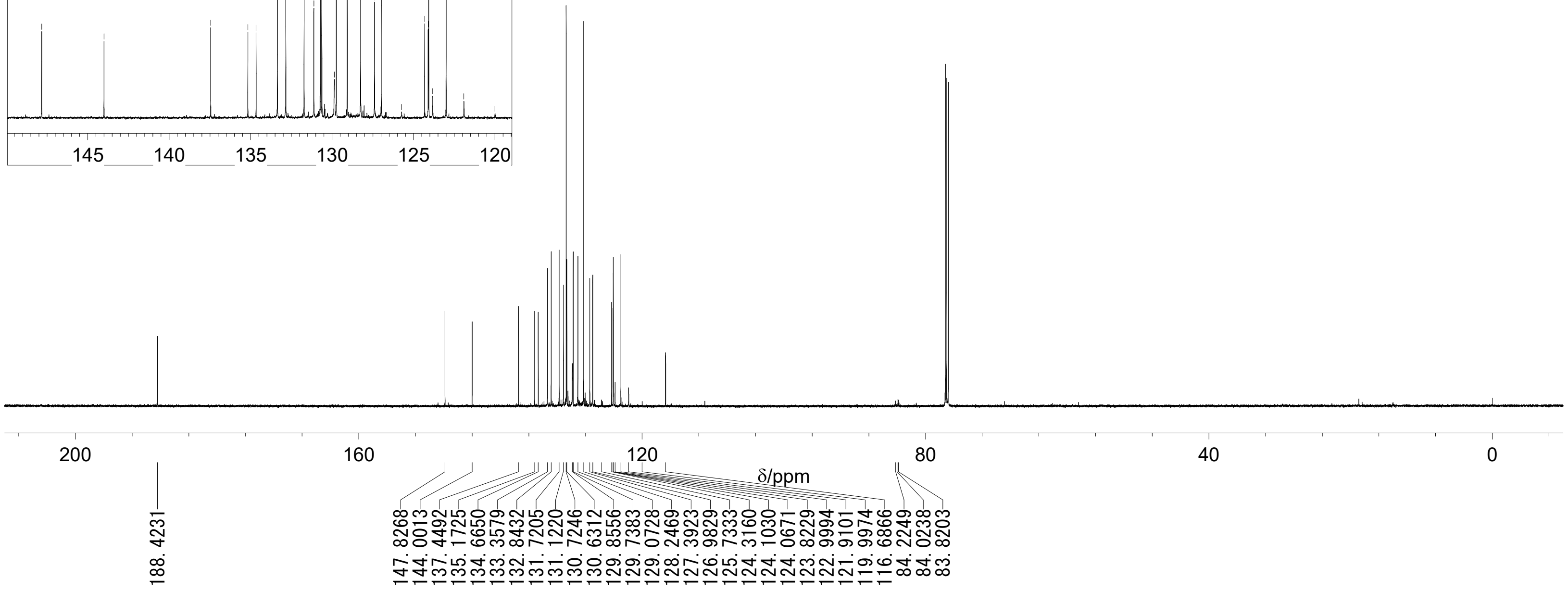

^{1}H NMR spectrum of **3kd** (400 MHz, $CDCl_3$)

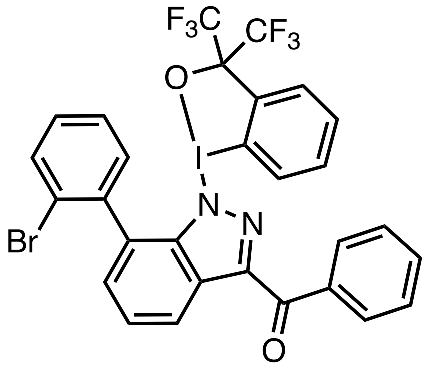

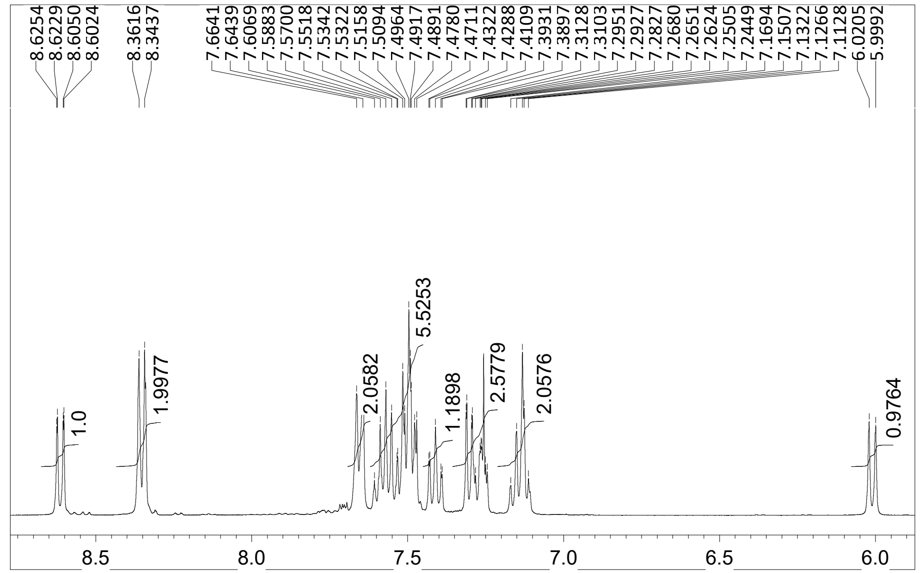

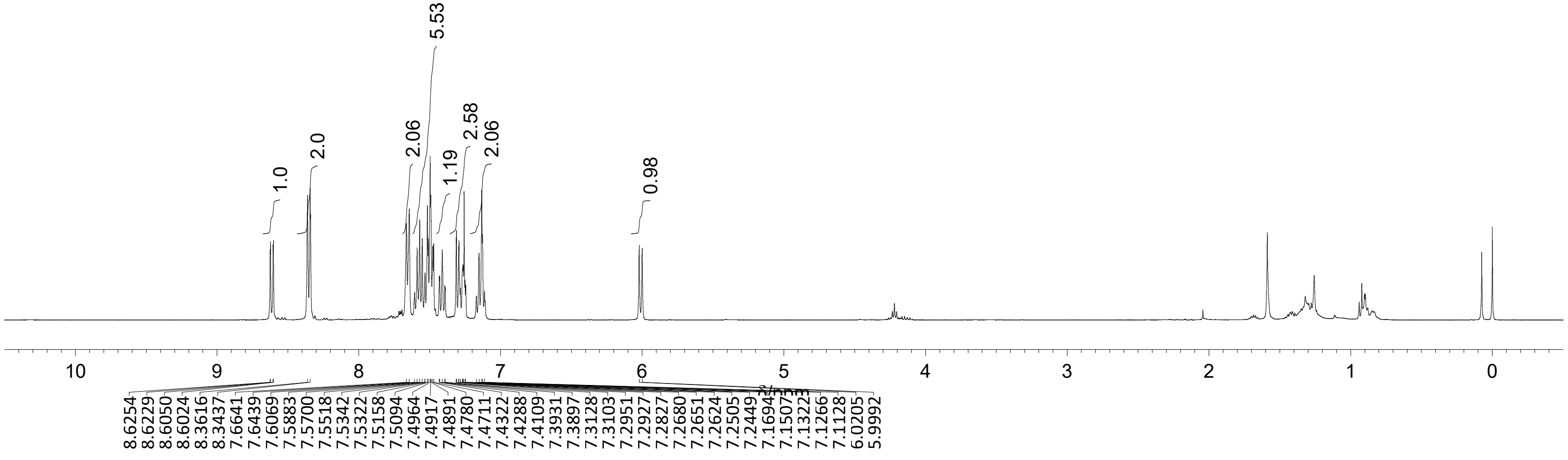

$^{13}C\{^1H\}$ NMR spectrum of **3kd** (150 MHz, $CDCl_3$)

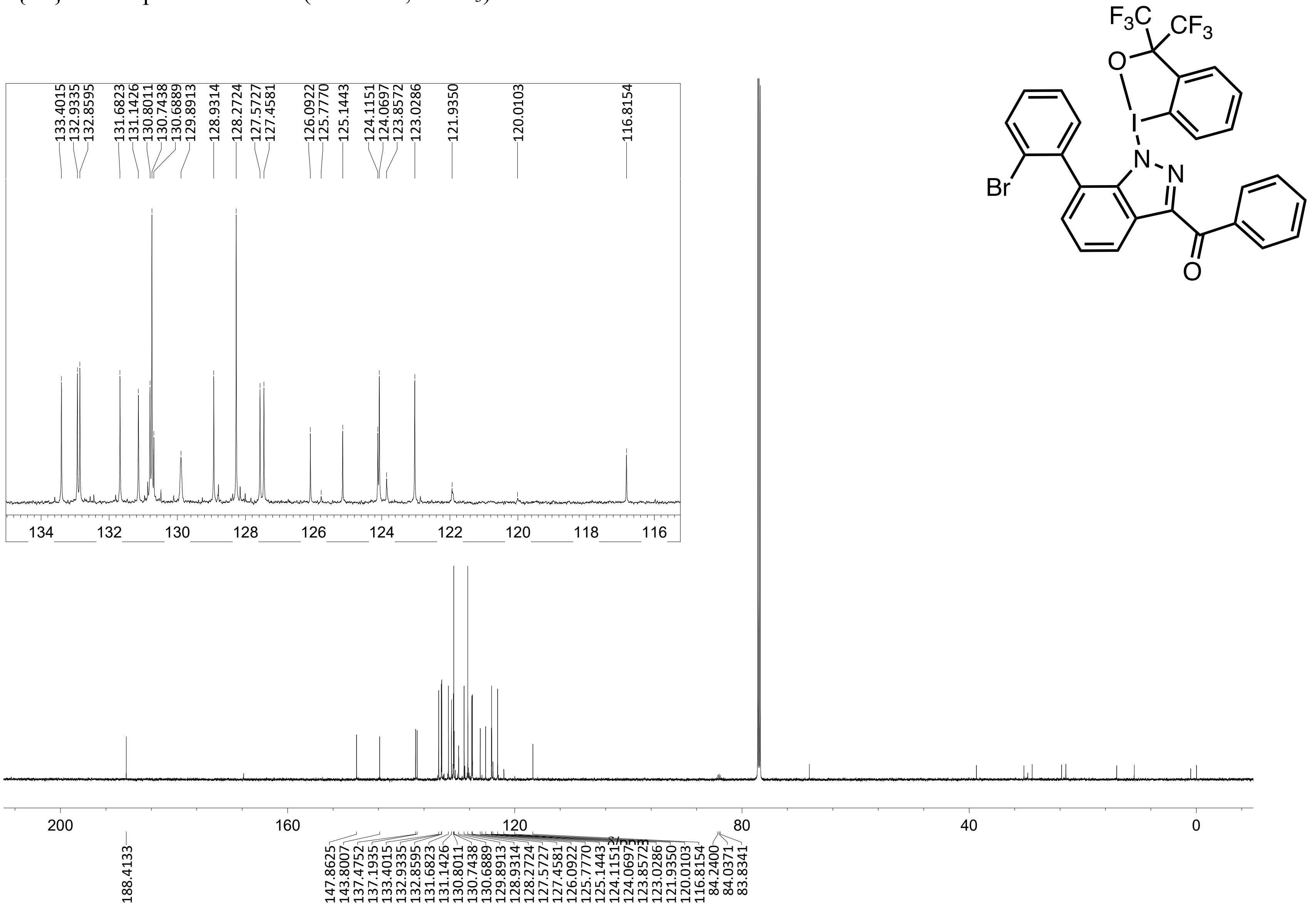

^{1}H NMR spectrum of **3ld** (400 MHz, $CDCl_3$)

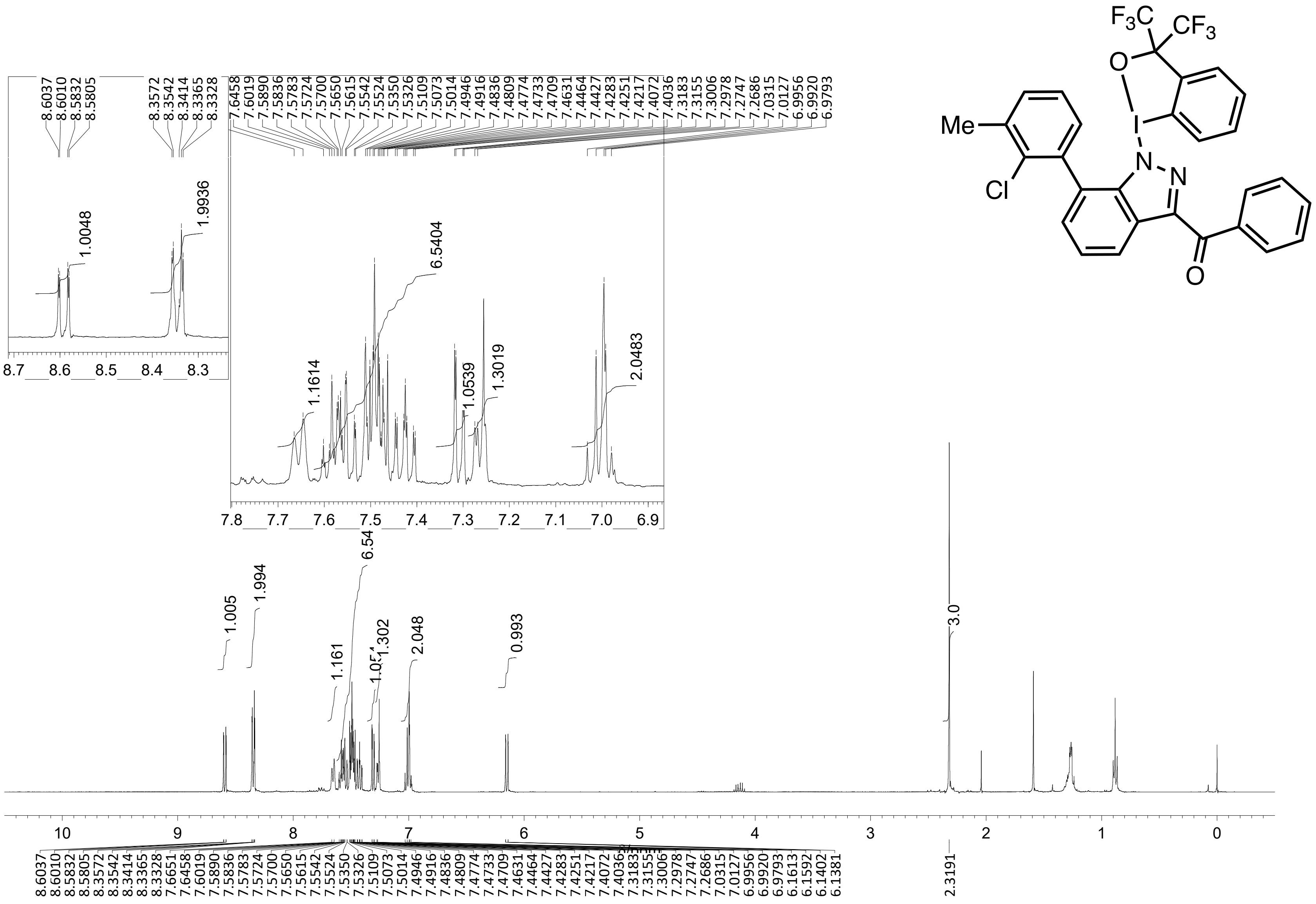

$^{13}C\{^{1}H\}$ NMR spectrum of **3ld** (150 MHz, $CDCl_3$)

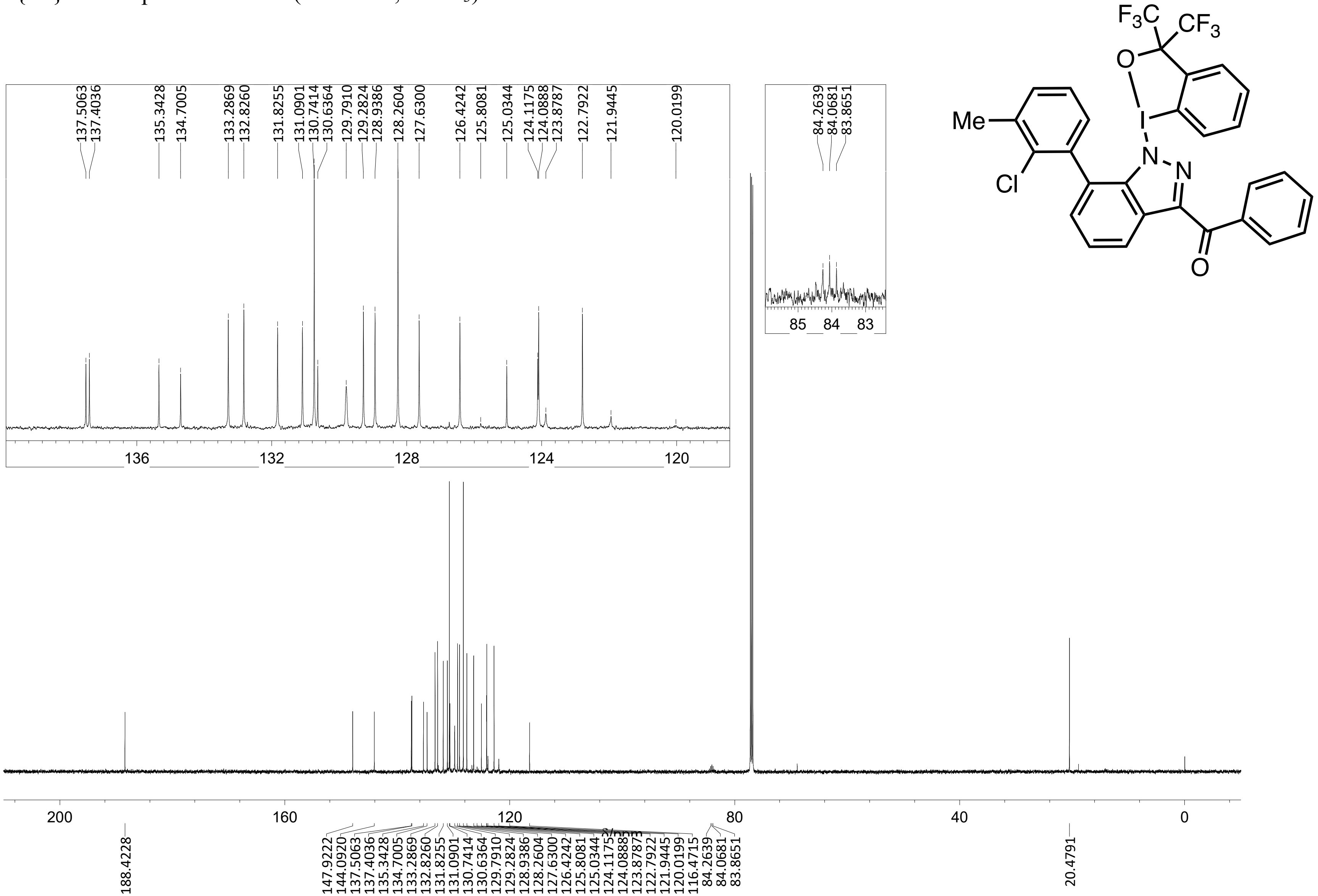

^{1}H NMR spectrum of **3md** (400 MHz, $CDCl_3$)

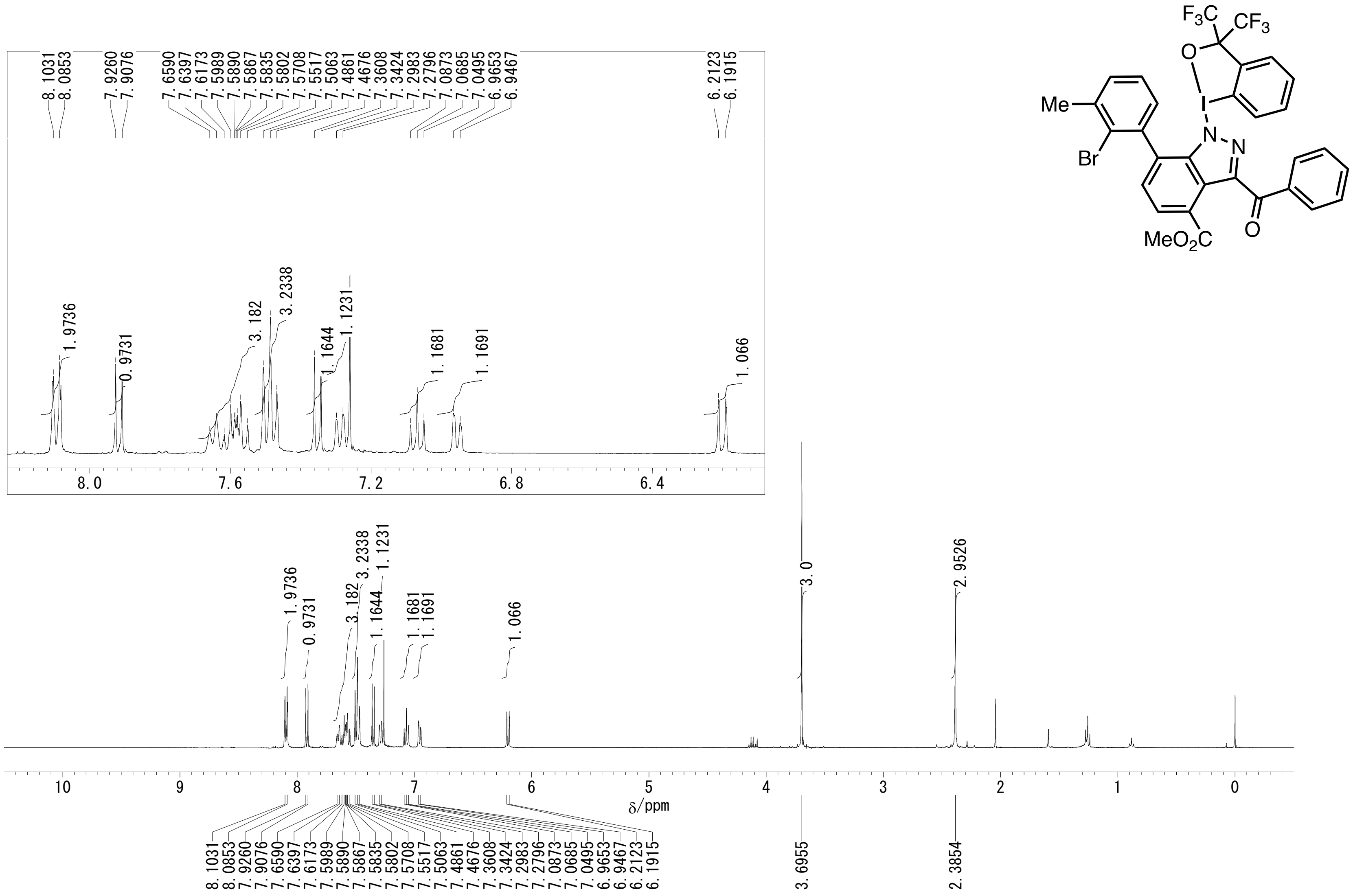

$^{13}C\{^{1}H\}$ NMR spectrum of **3md** (150 MHz, $CDCl_3$)

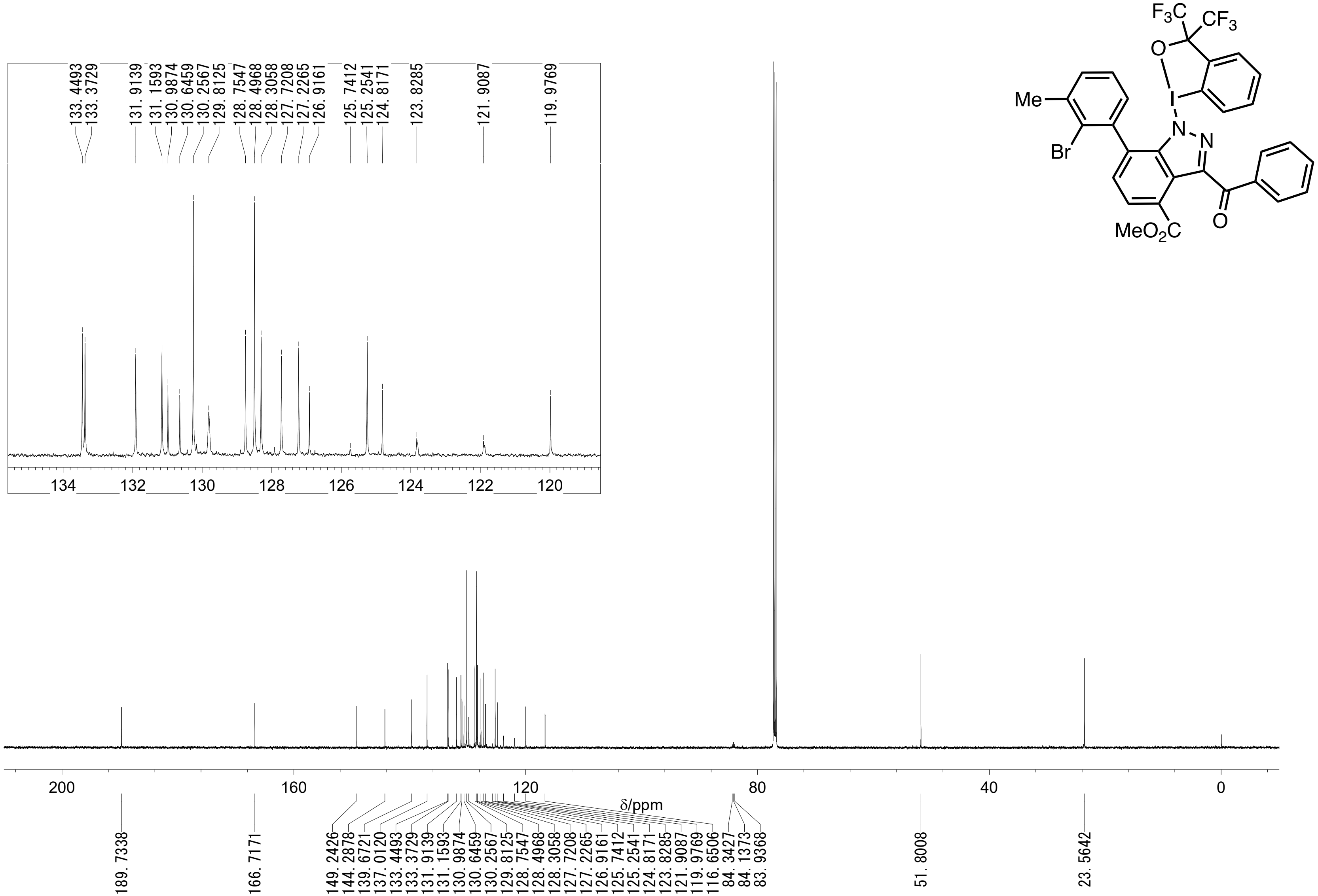

^{1}H NMR spectrum of **3nd** and **3nd’** (400 MHz, $CDCl_3$)

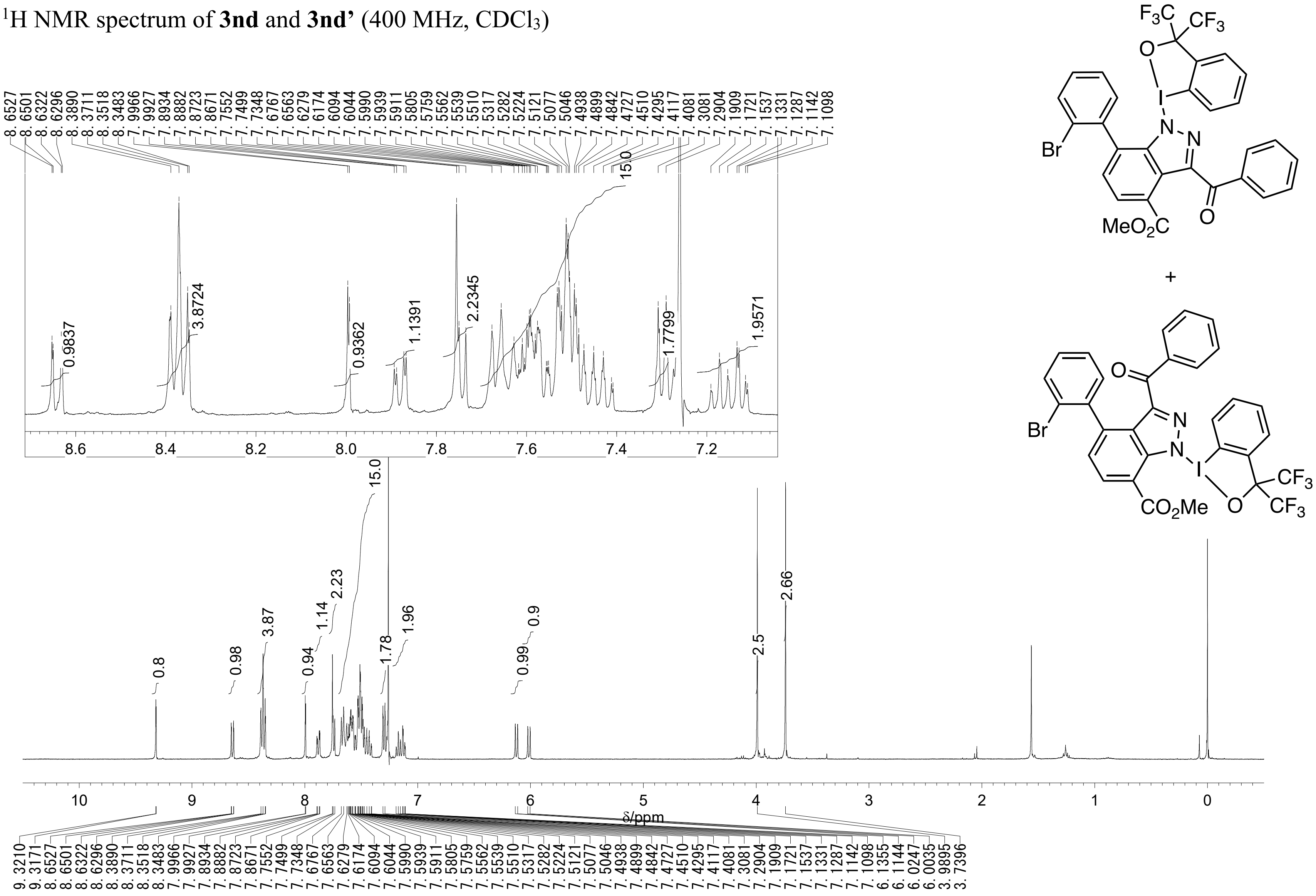

$^{13}C\{^1H\}$ NMR spectrum of **3nd** and **3nd'** (150 MHz, $CDCl_3$)

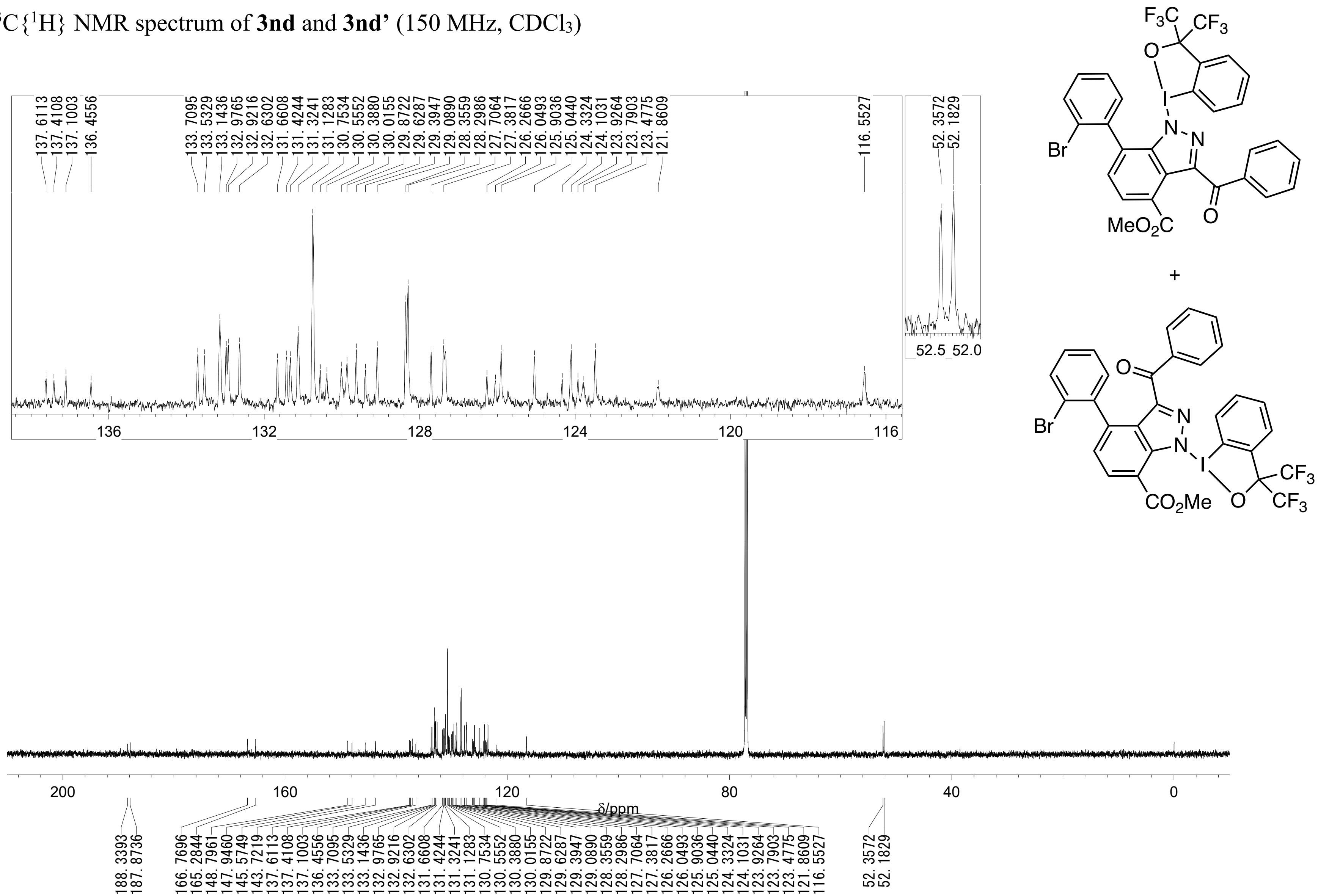

^{1}H NMR spectrum of **3jf** (400 MHz, $CDCl_3$)

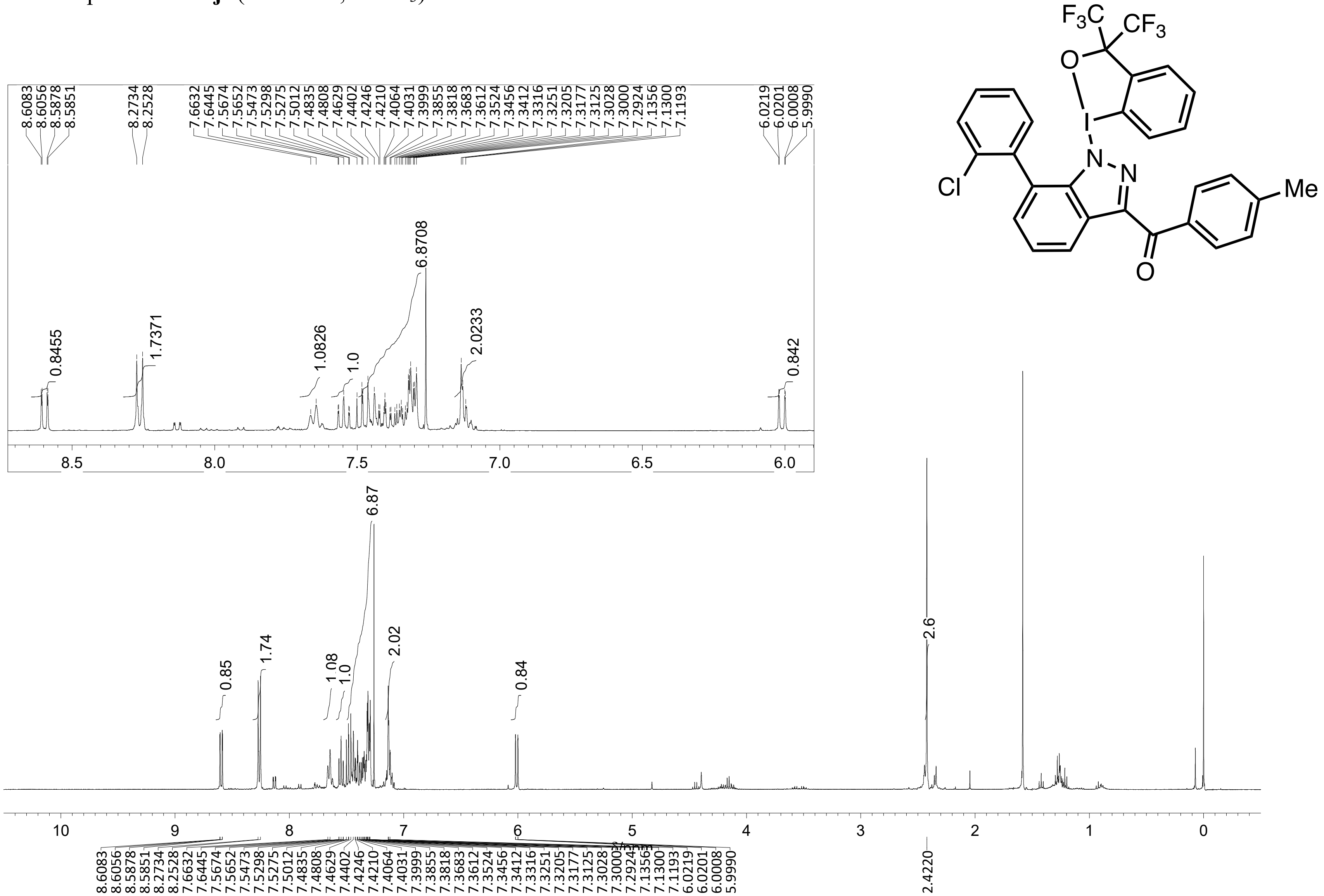

$^{13}C\{^1H\}$ NMR spectrum of **3jf** (150 MHz, $CDCl_3$)

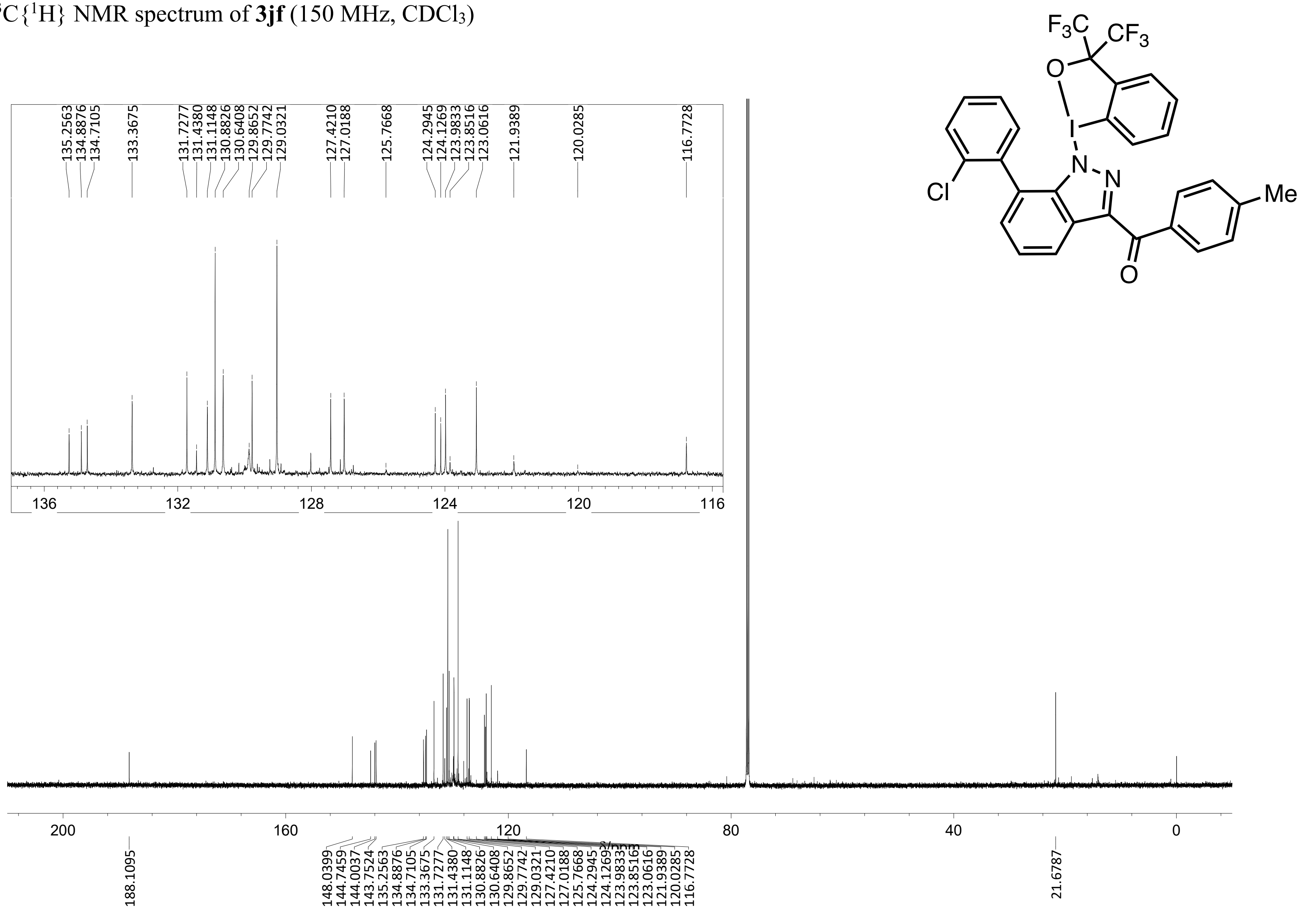

^{1}H NMR spectrum of **3jg** (400 MHz, $CDCl_3$)

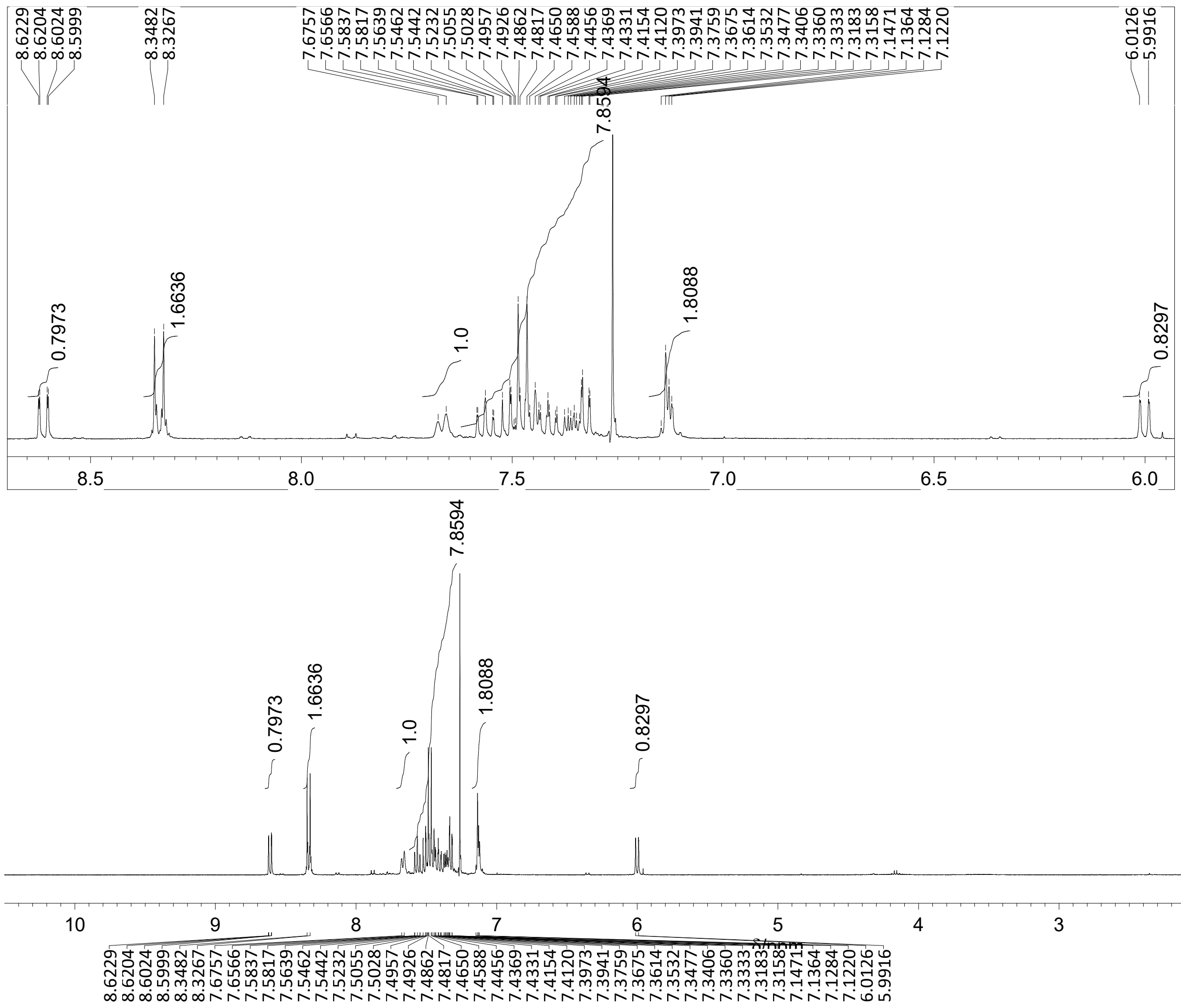

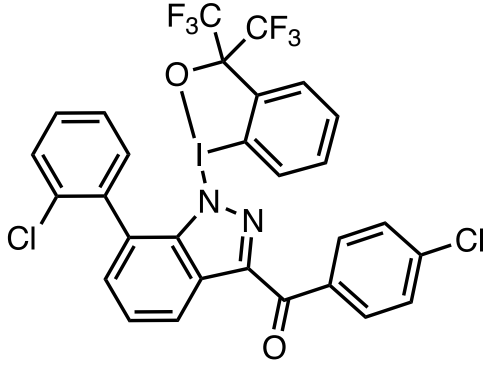

$^{13}C\{^1H\}$ NMR spectrum of **3jg** (150 MHz, $CDCl_3$)

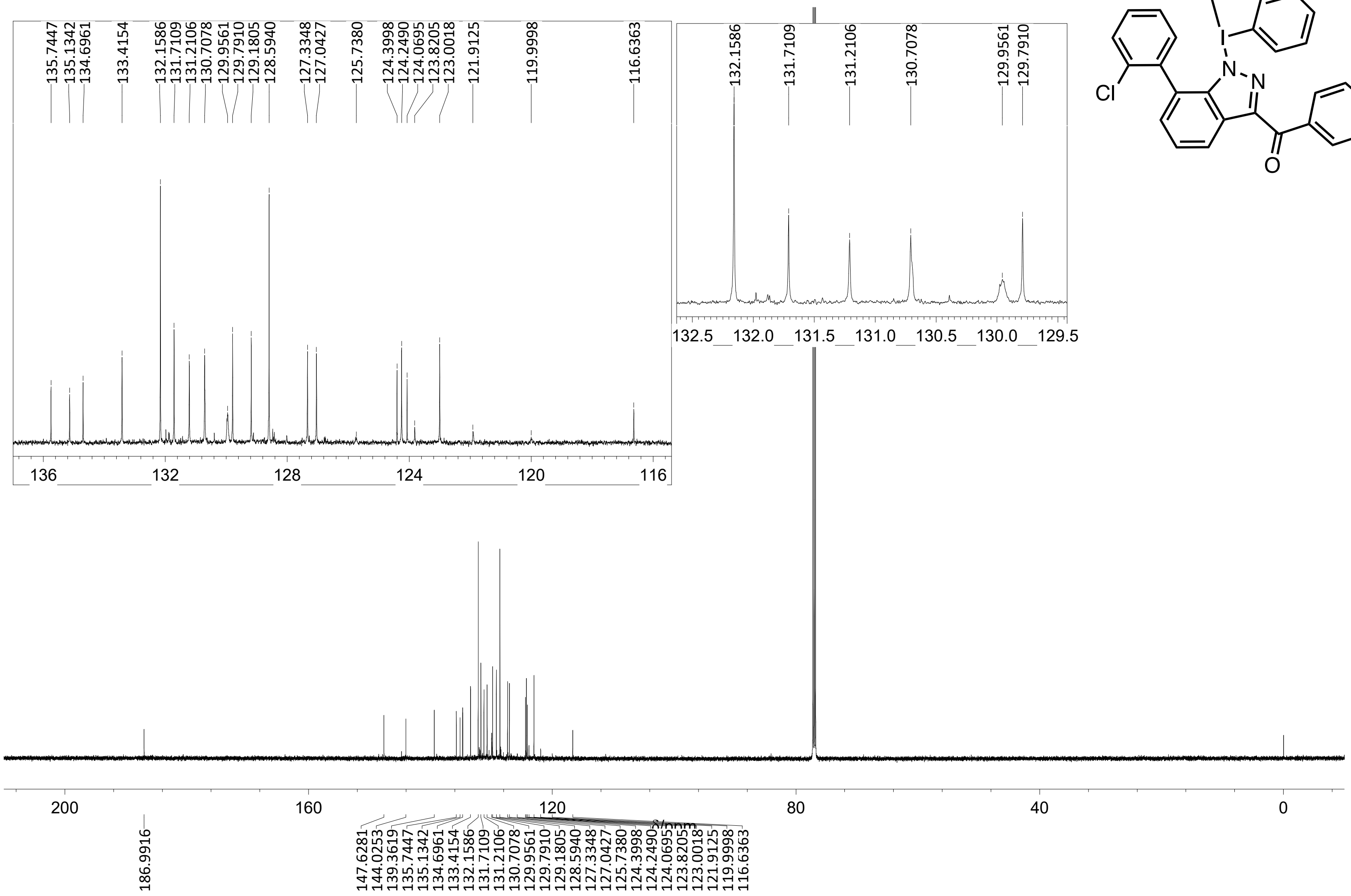

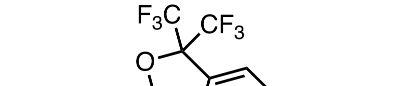

^{1}H NMR spectrum of **3ji** (400 MHz, $CDCl_3$)

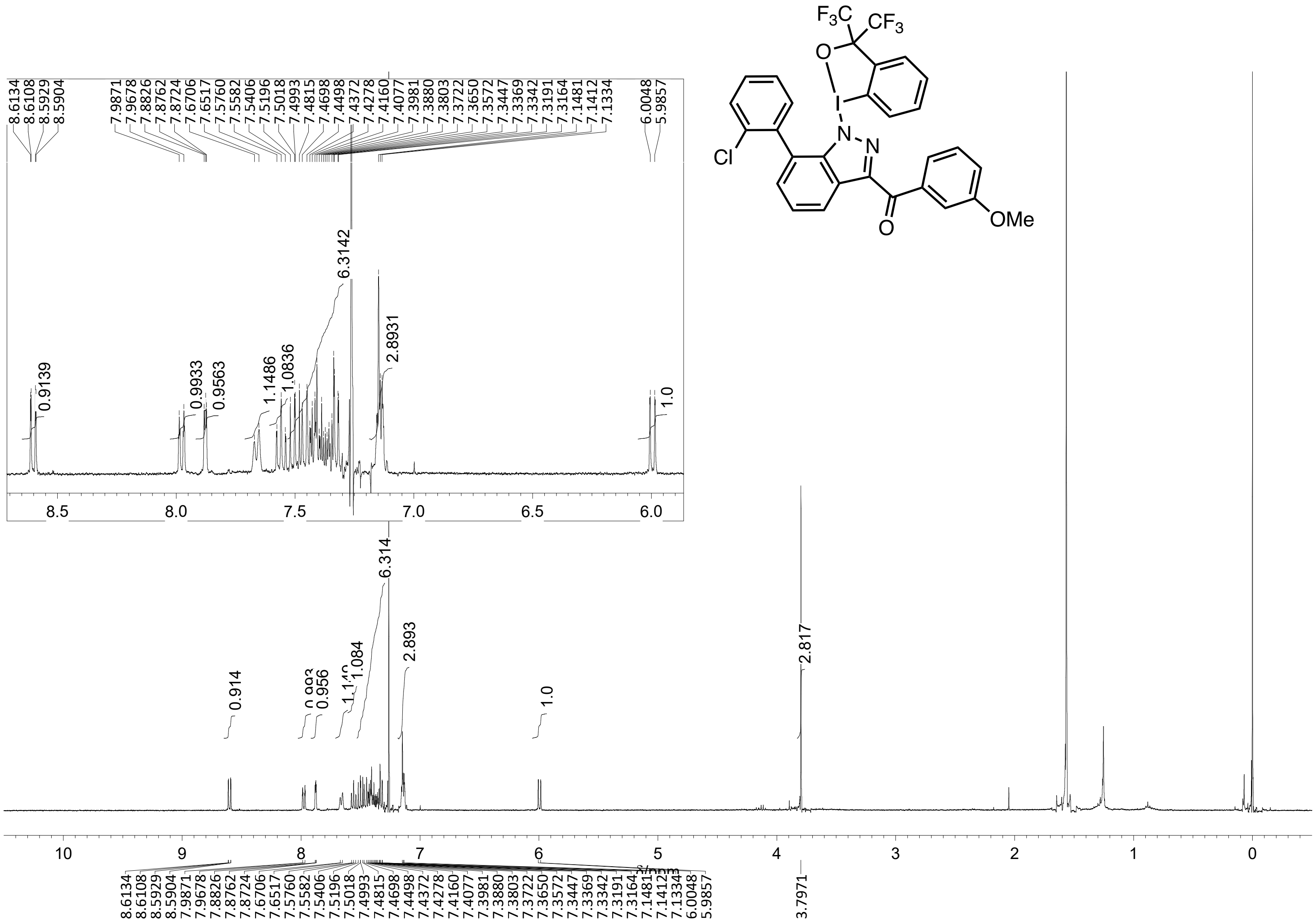

$^{13}C\{^{1}H\}$ NMR spectrum of **3ji** (150 MHz, $CDCl_3$)

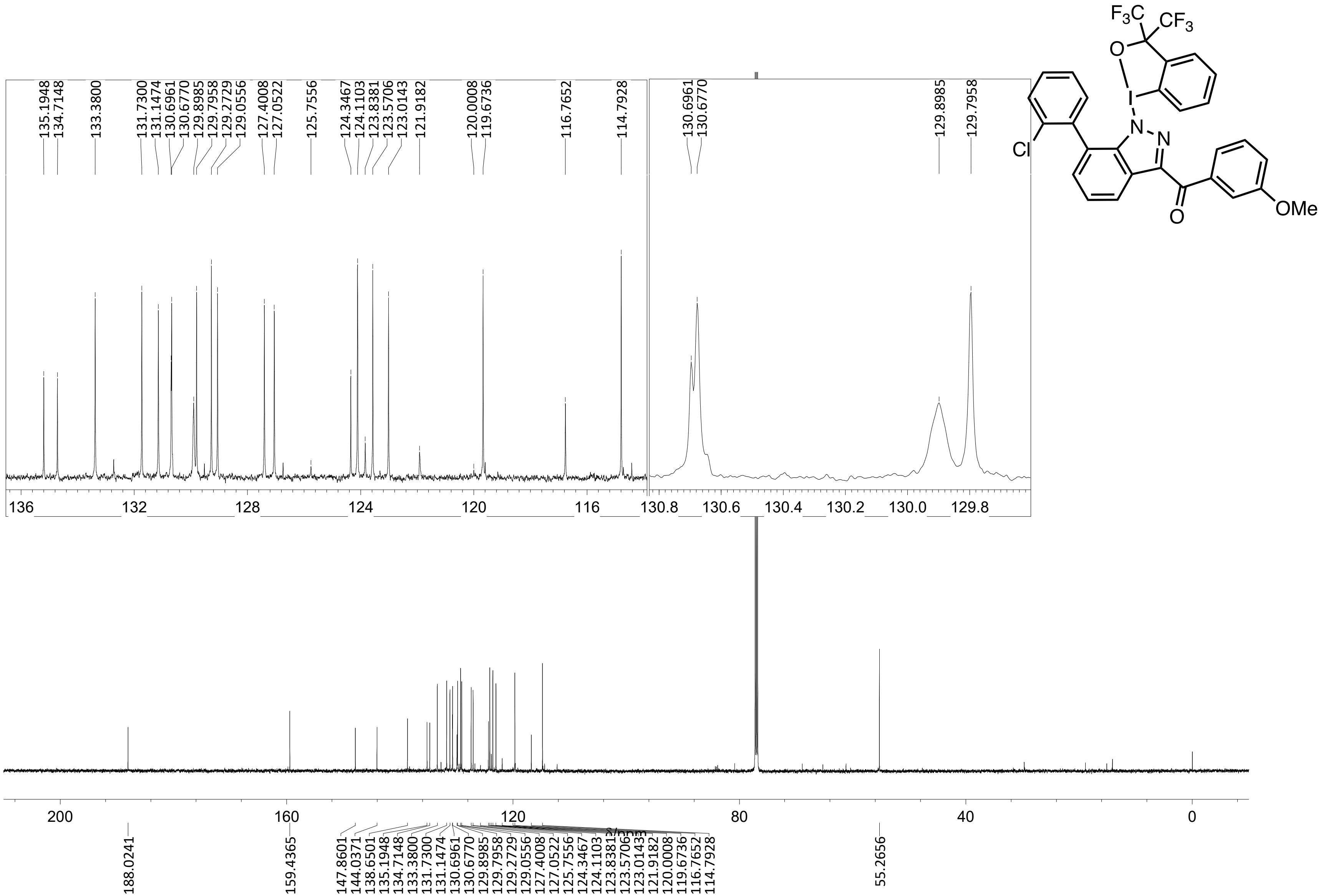

^{1}H NMR spectrum of **3jj** (400 MHz, $CDCl_3$)

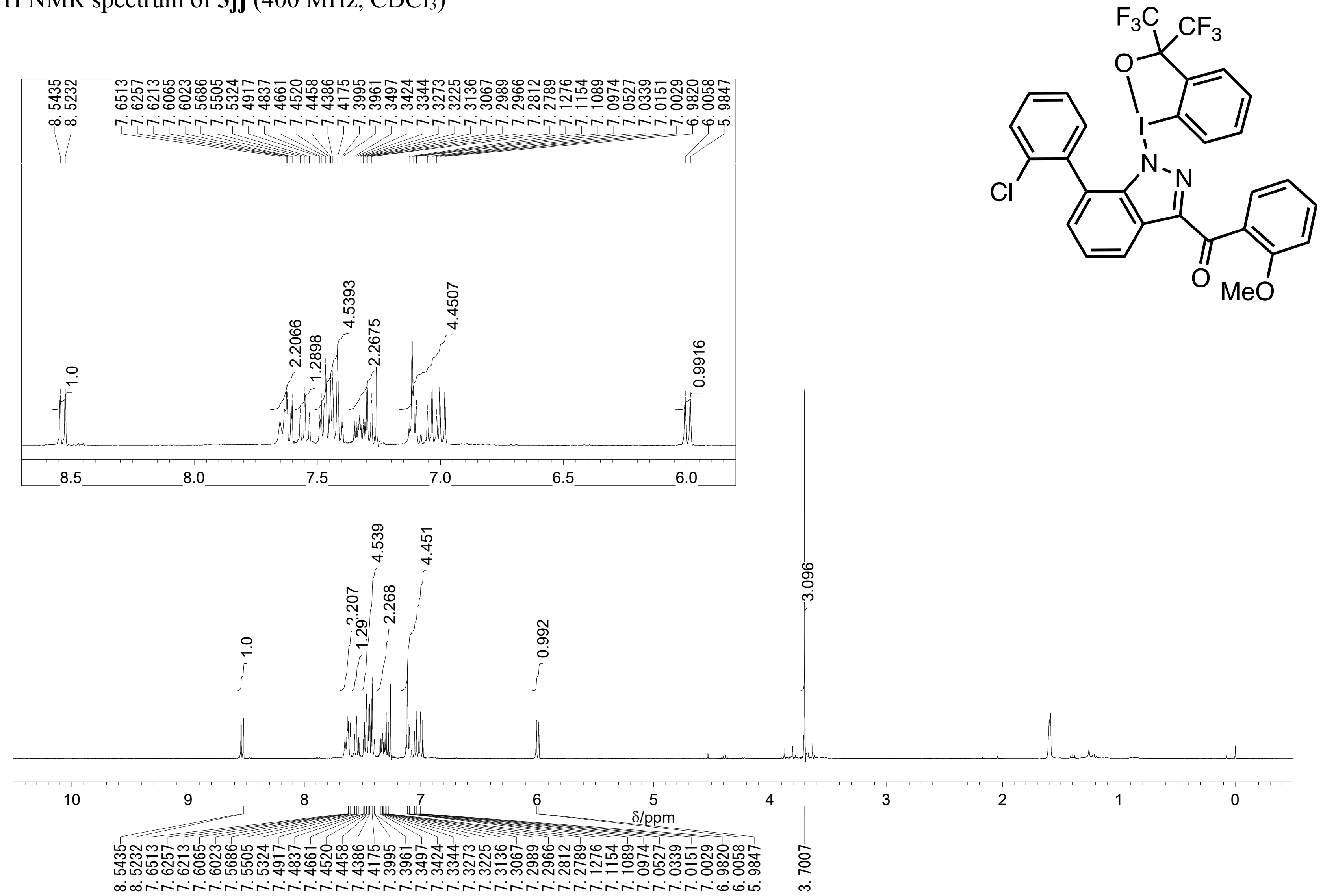

$^{13}C\{^{1}H\}$ NMR spectrum of **3jj** (150 MHz, $CDCl_3$)

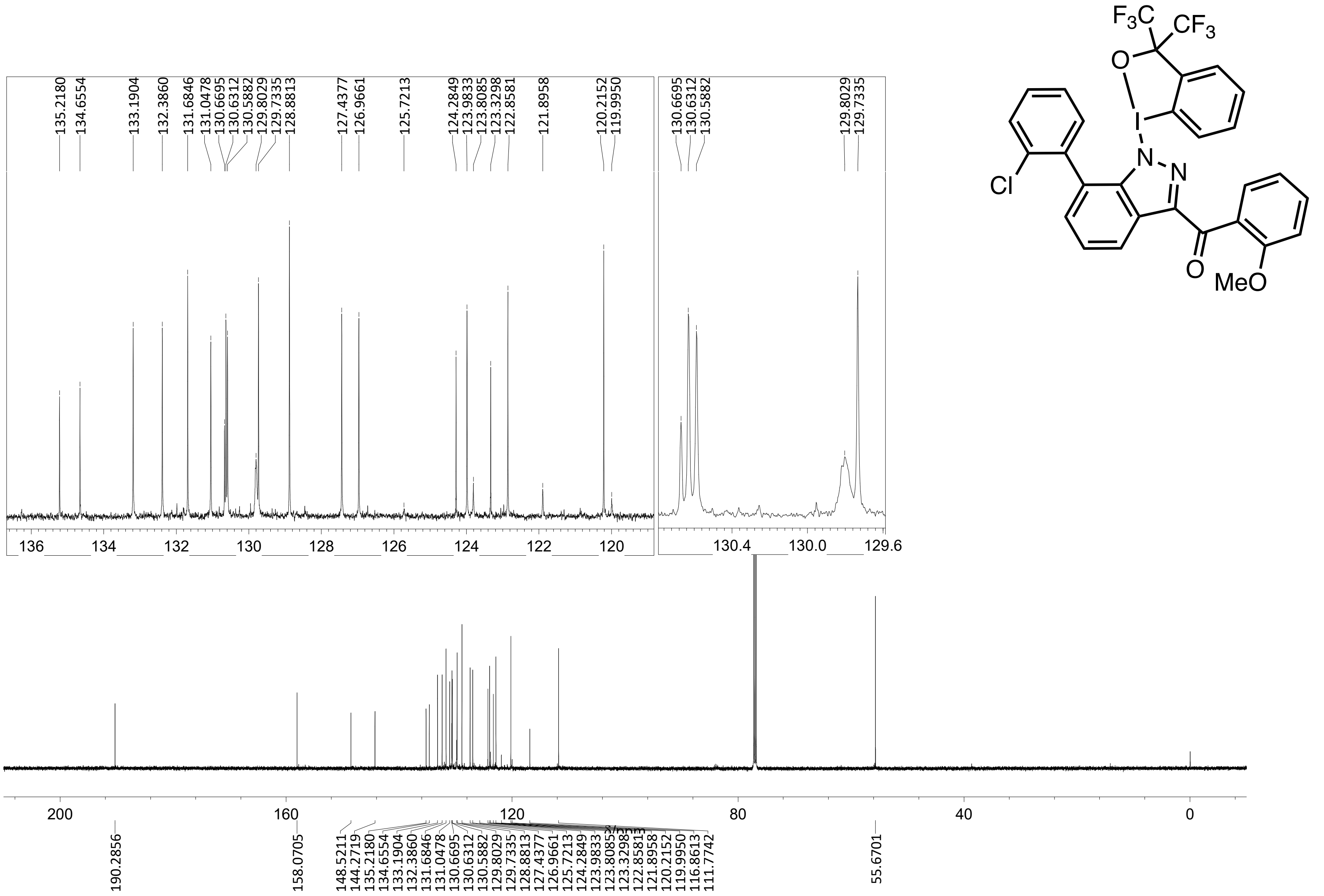

^{1}H NMR spectrum of **3jk** (400 MHz, $CDCl_3$)

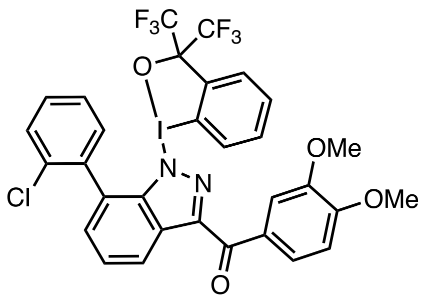

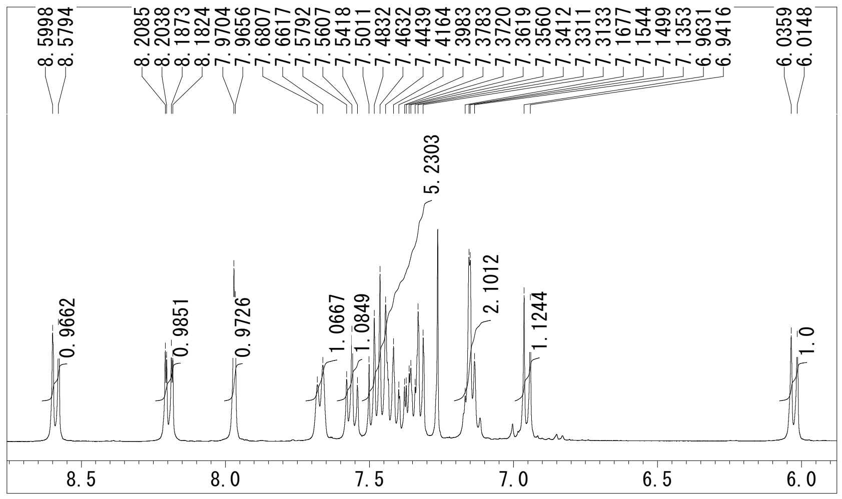

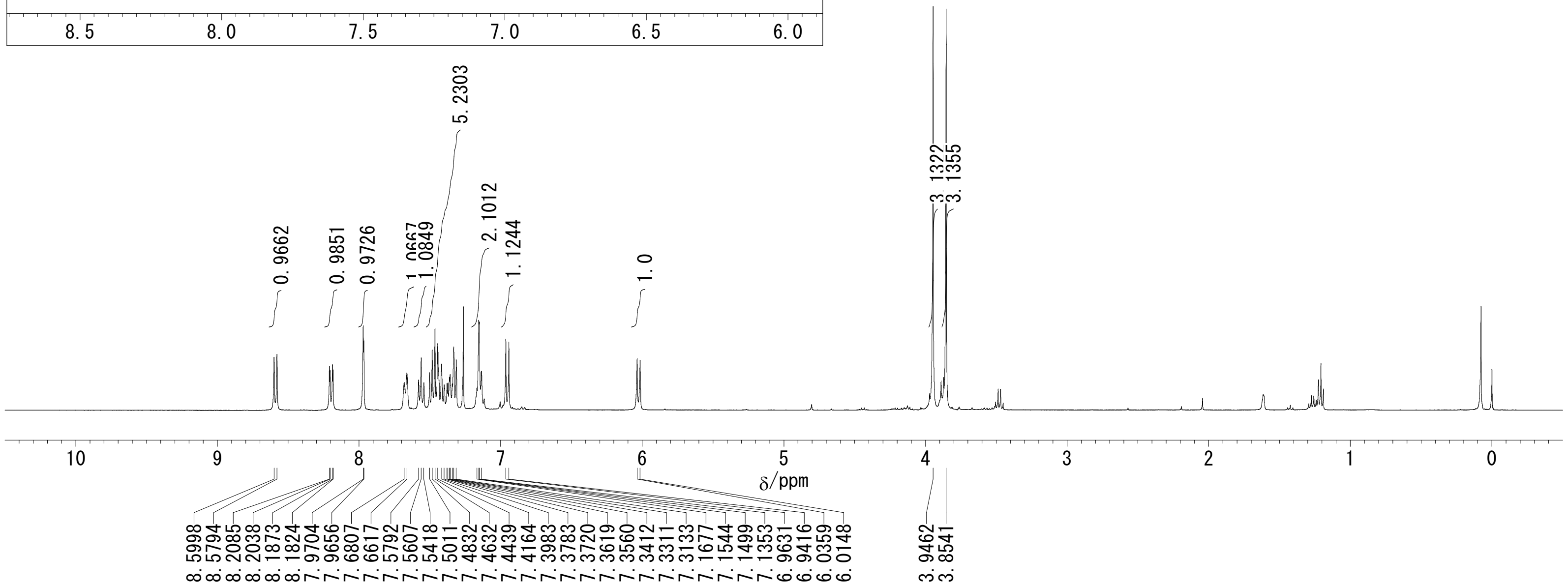

$^{13}C\{^{1}H\}$ NMR spectrum of **3jk** (150 MHz, $CDCl_3$)

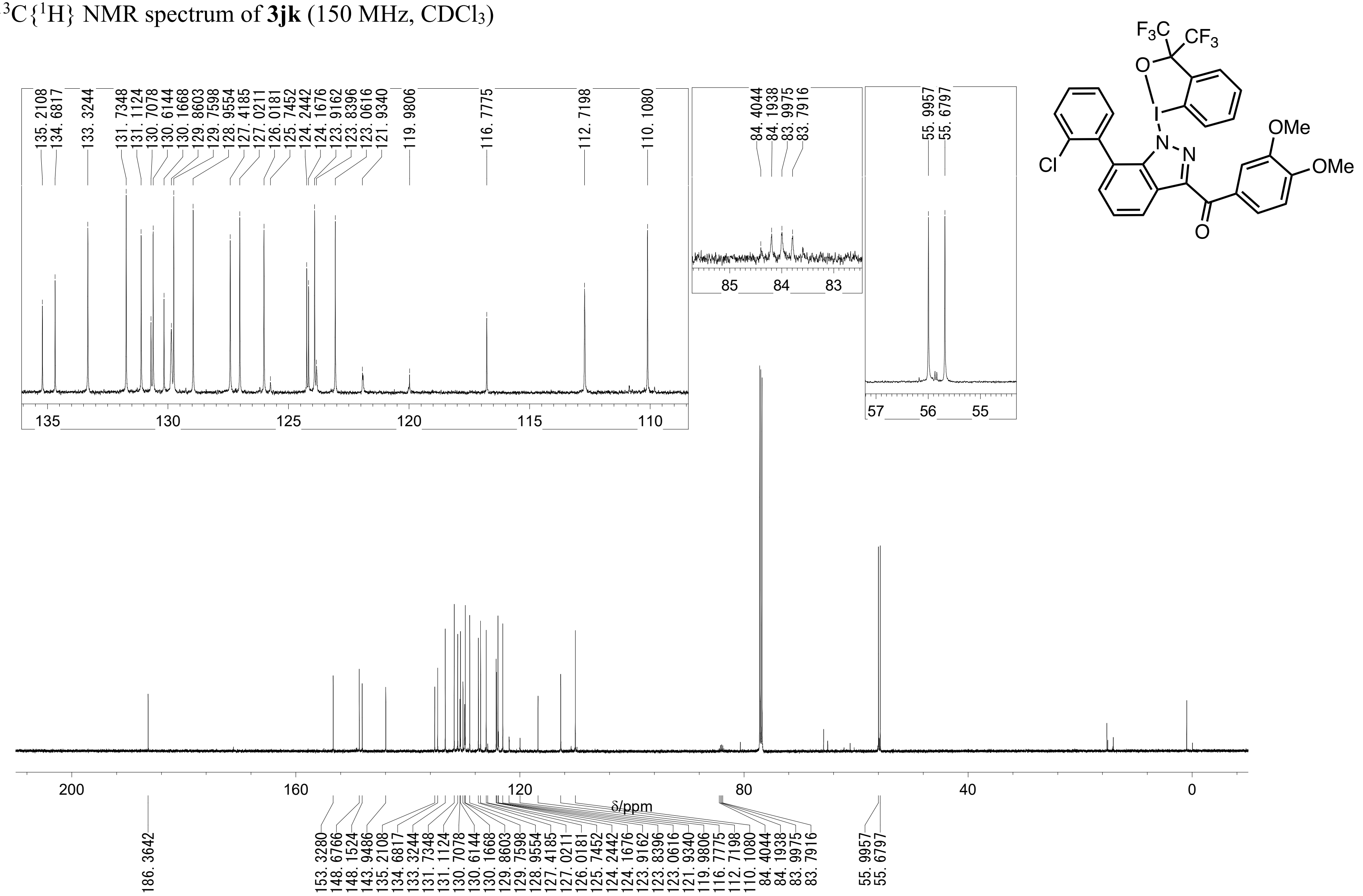

^{1}H NMR spectrum of **3jl** (400 MHz, $CDCl_3$)

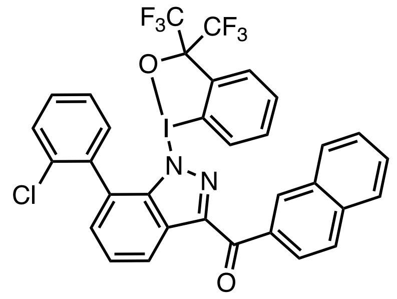

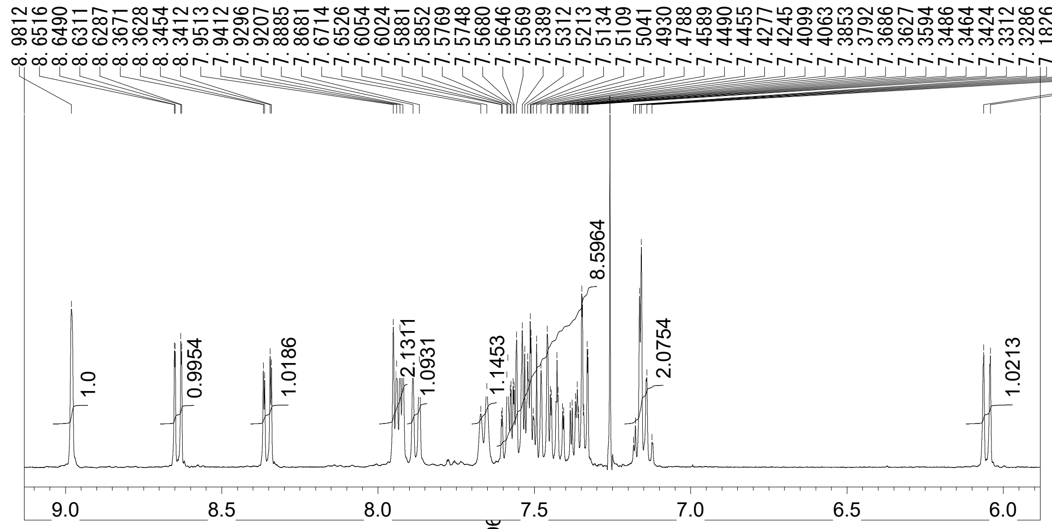

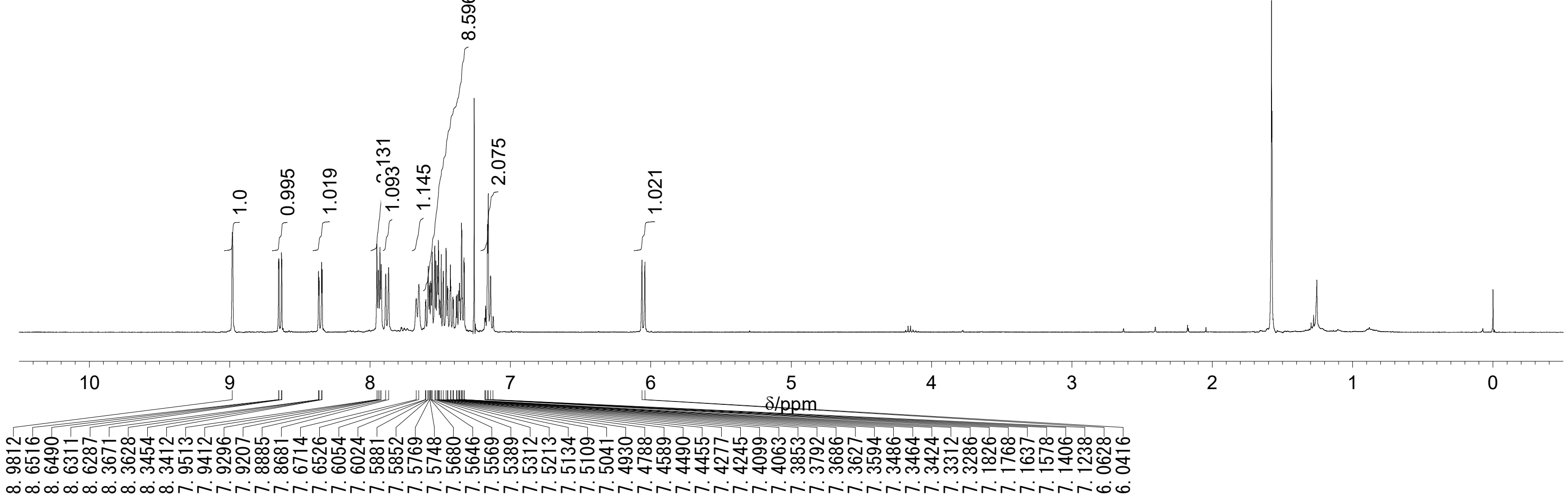

$^{13}C\{^{1}H\}$ NMR spectrum of **3jl** (150 MHz, $CDCl_3$)

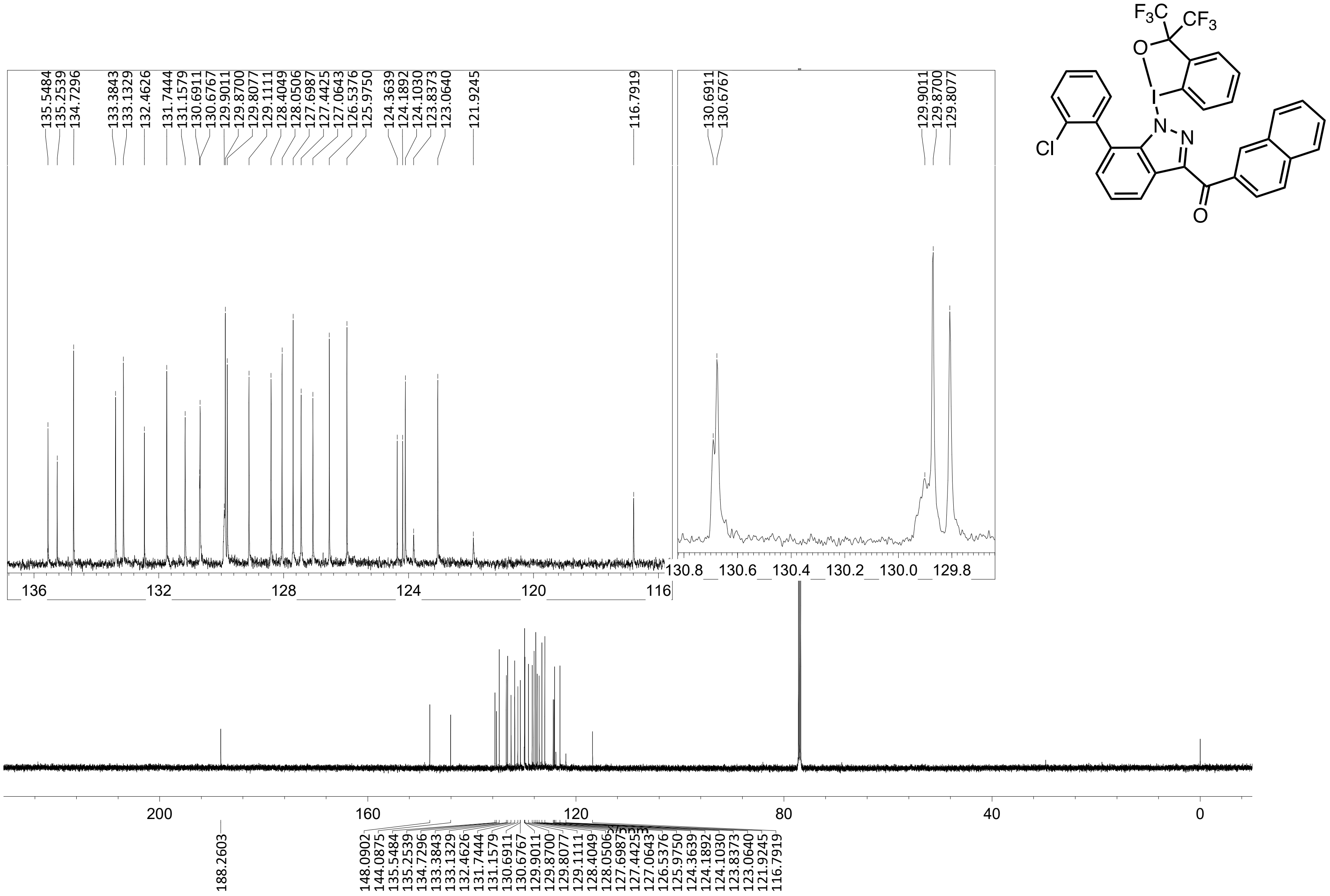

^{1}H NMR spectrum of **3jm** (400 MHz, $CDCl_3$)

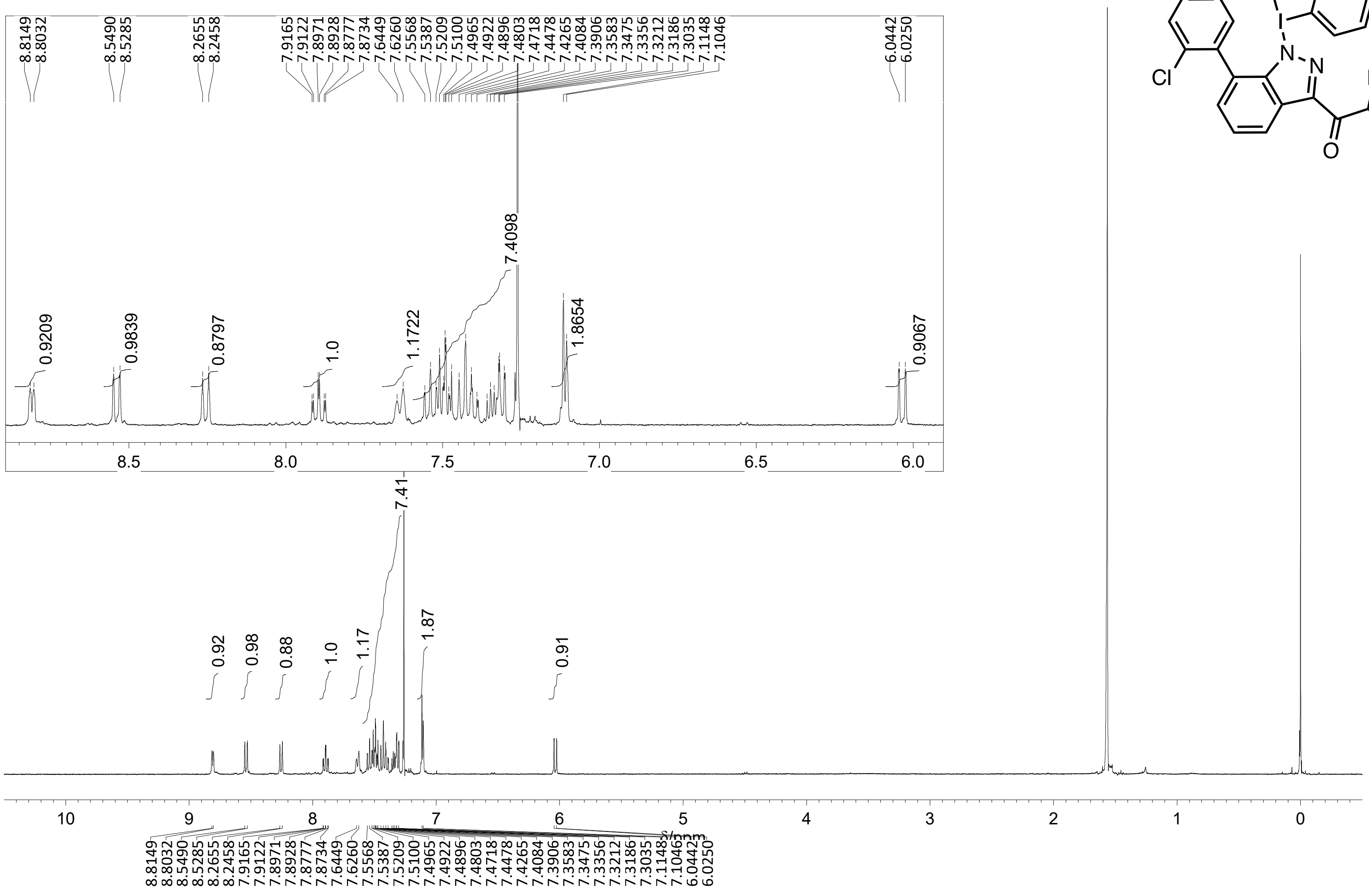

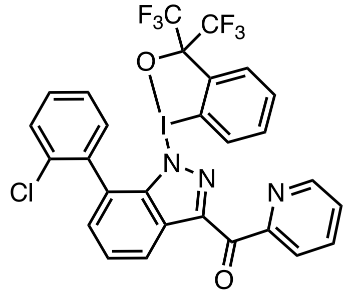

$^{13}C\{^{1}H\}$ NMR spectrum of **3jm** (150 MHz, $CDCl_3$)

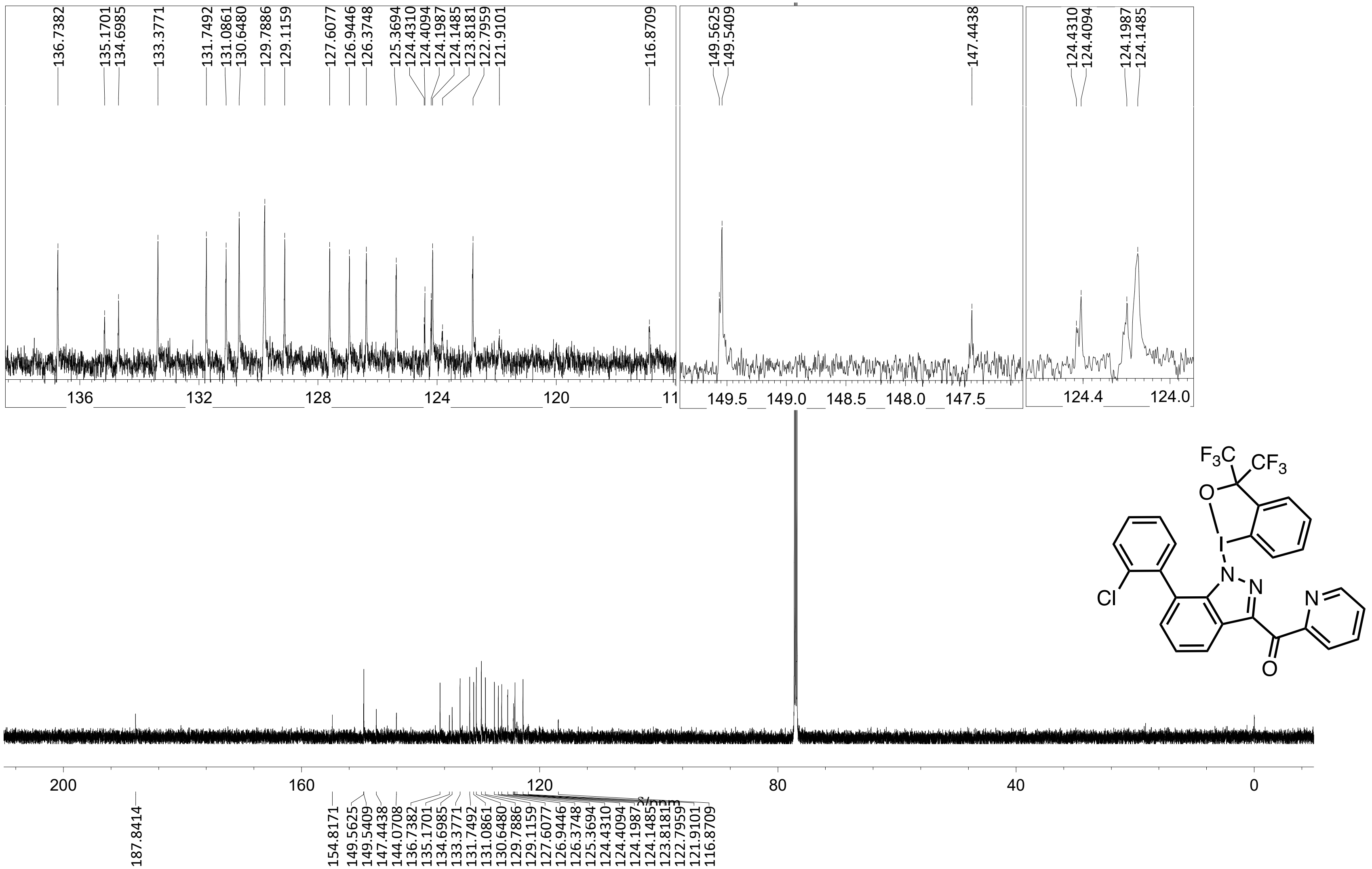

^{1}H NMR spectrum of **3jn** (400 MHz, $CDCl_3$)

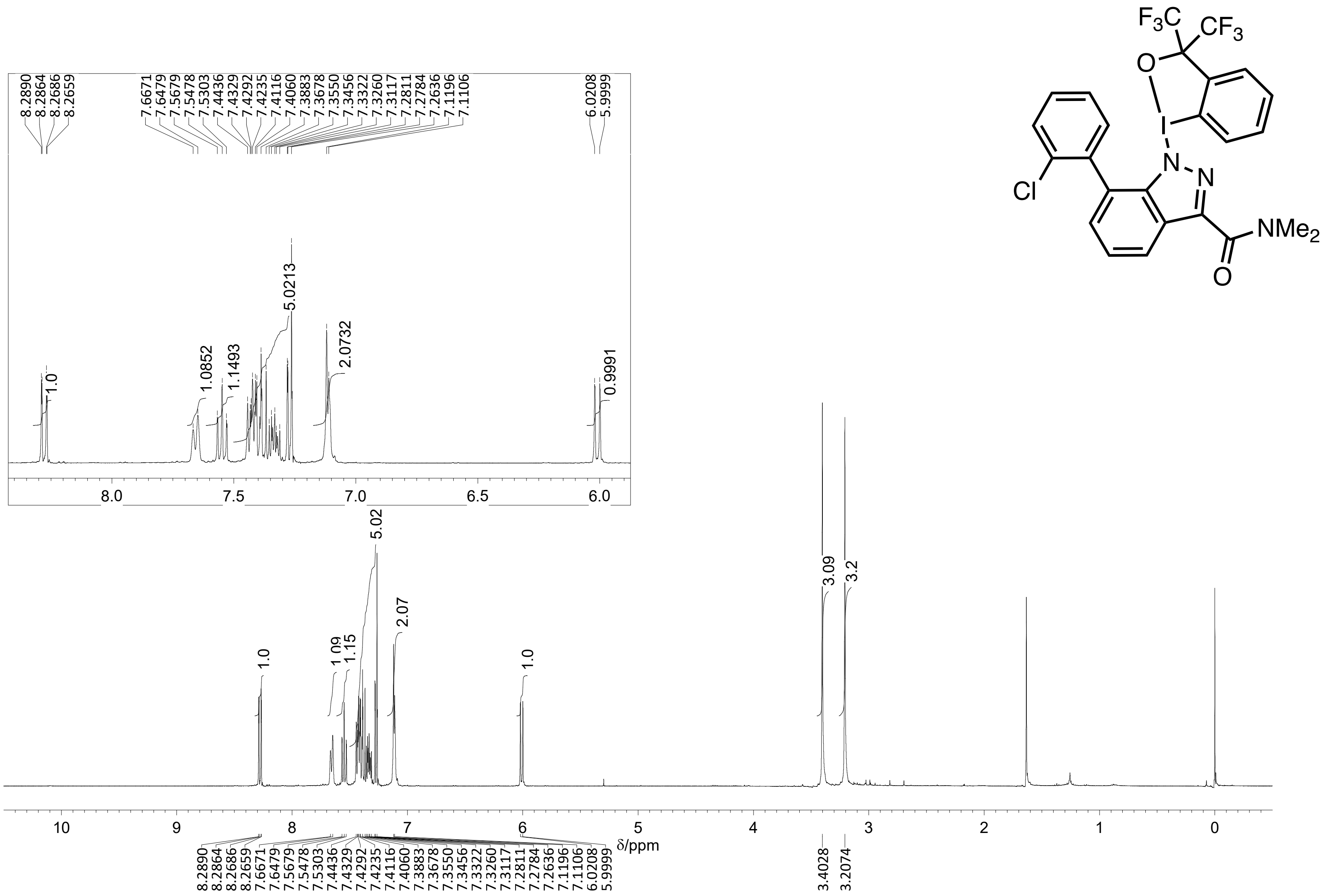

$^{13}C\{^{1}H\}$ NMR spectrum of **3jn** (150 MHz, $CDCl_3$)

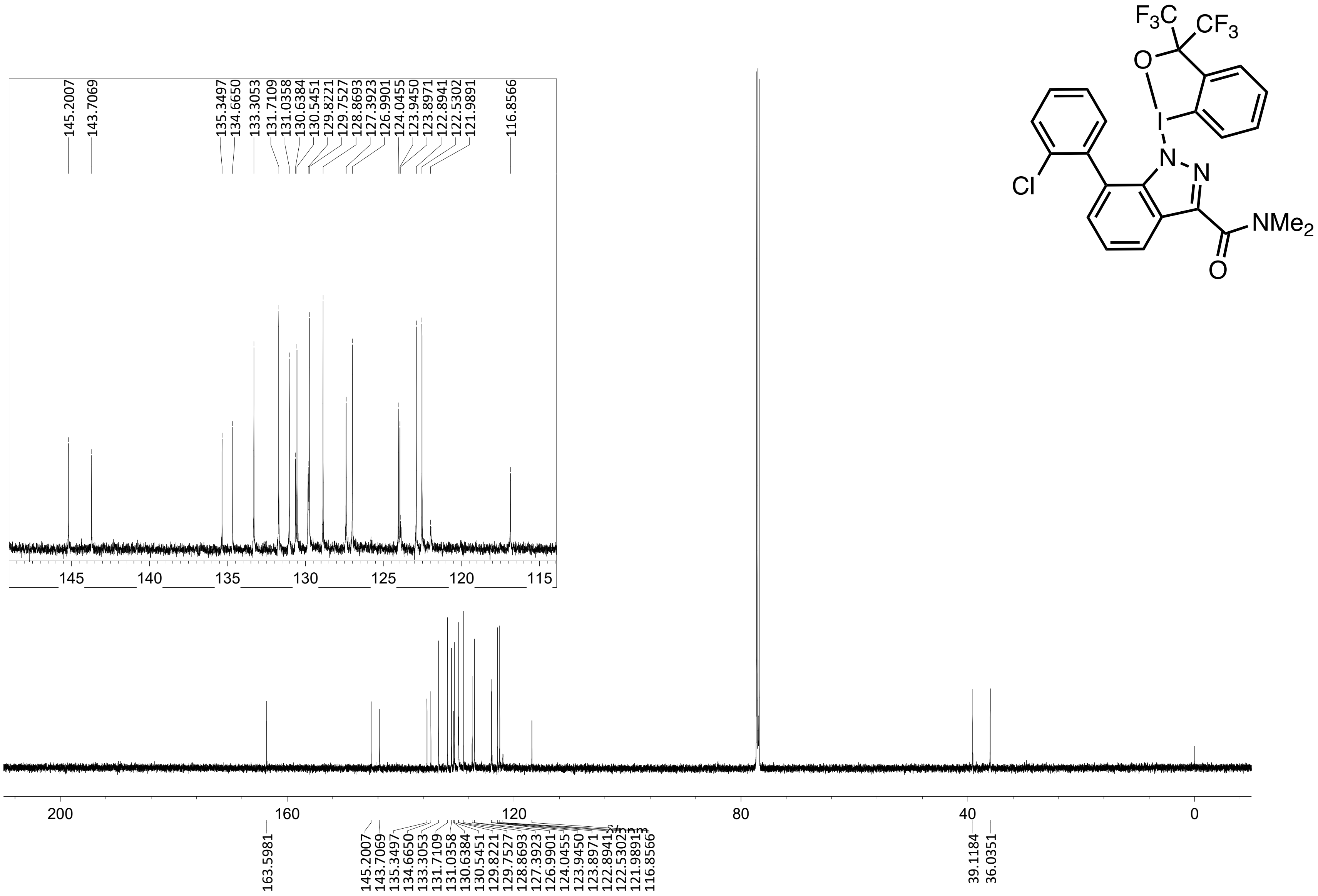

^{1}H NMR spectrum of **3jo** (400 MHz, $CDCl_3$)

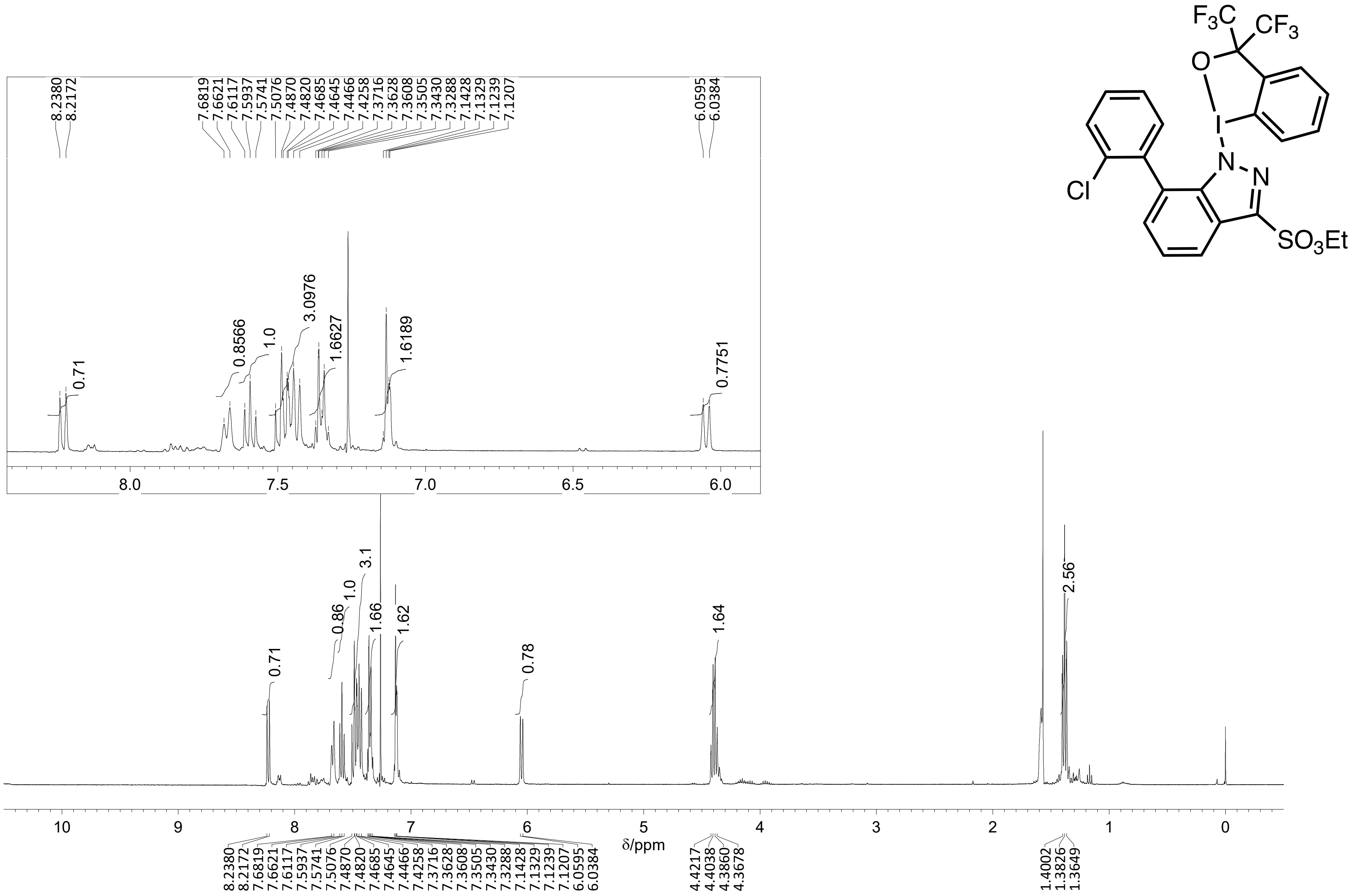

$^{13}C\{^{1}H\}$ NMR spectrum of **3jo** (150 MHz, $CDCl_3$)

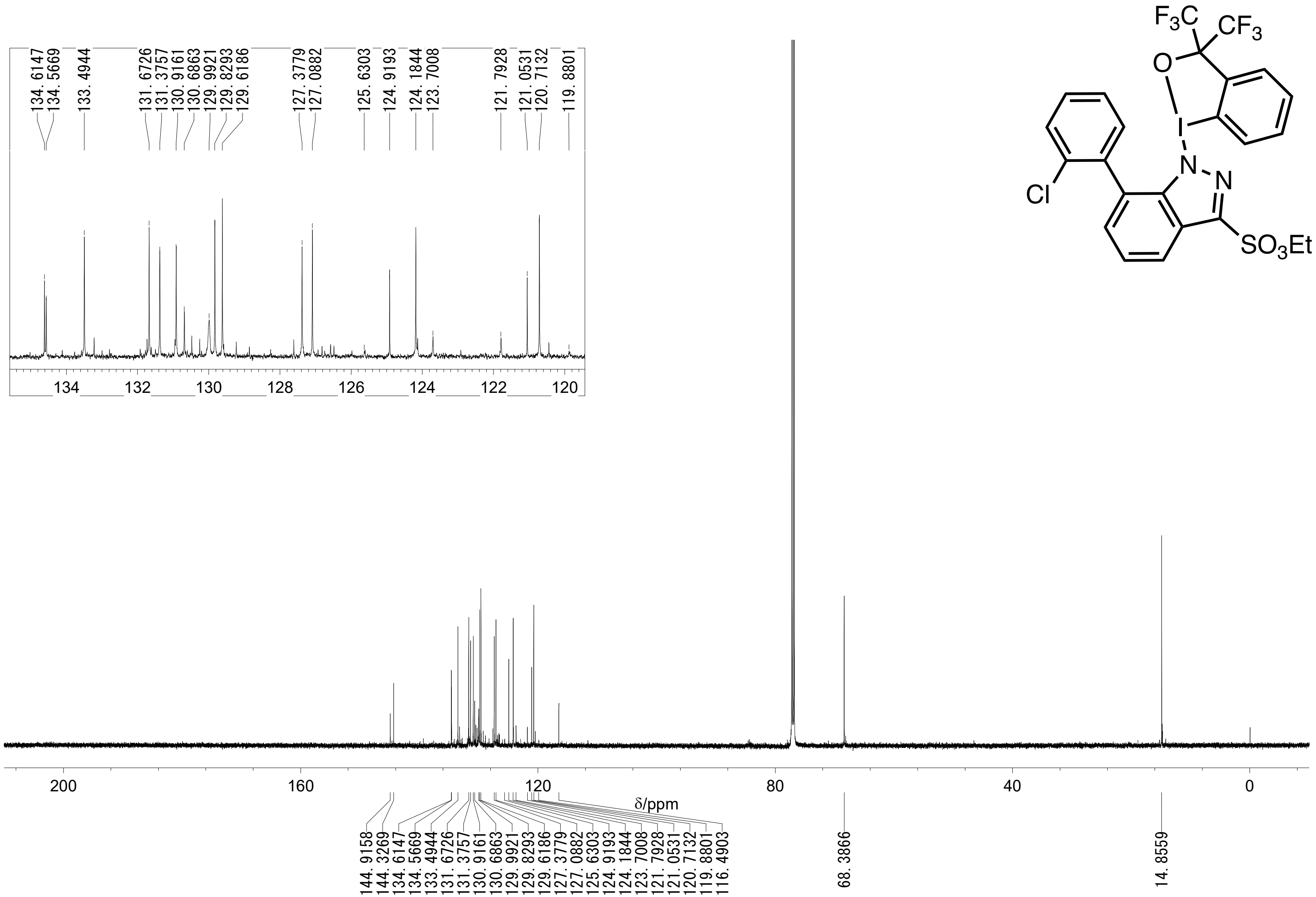

^{1}H NMR spectrum of **3jp** (400 MHz, $CDCl_3$)

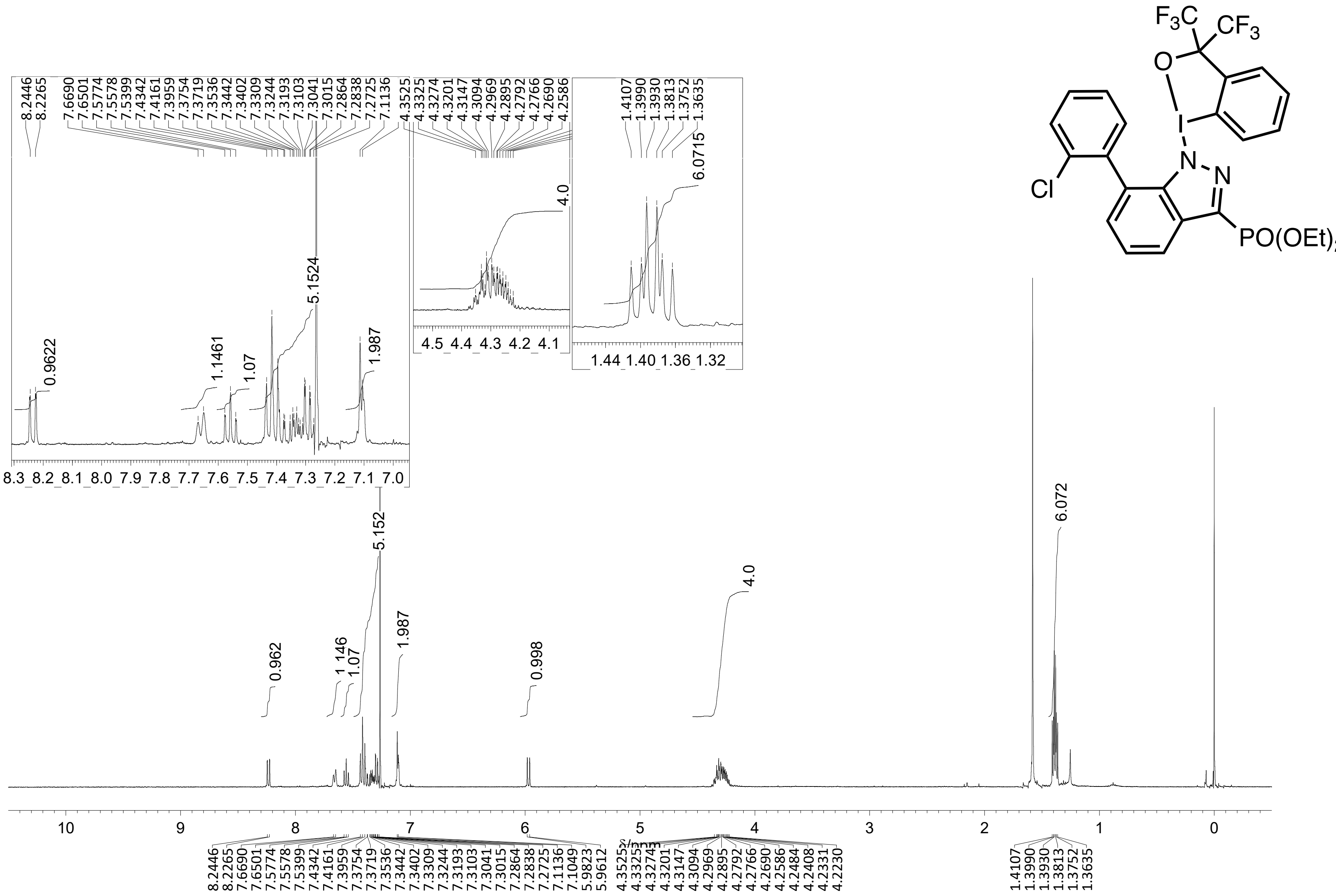

$^{13}C\{^{1}H\}$ NMR spectrum of **3jp** (150 MHz, $CDCl_3$)

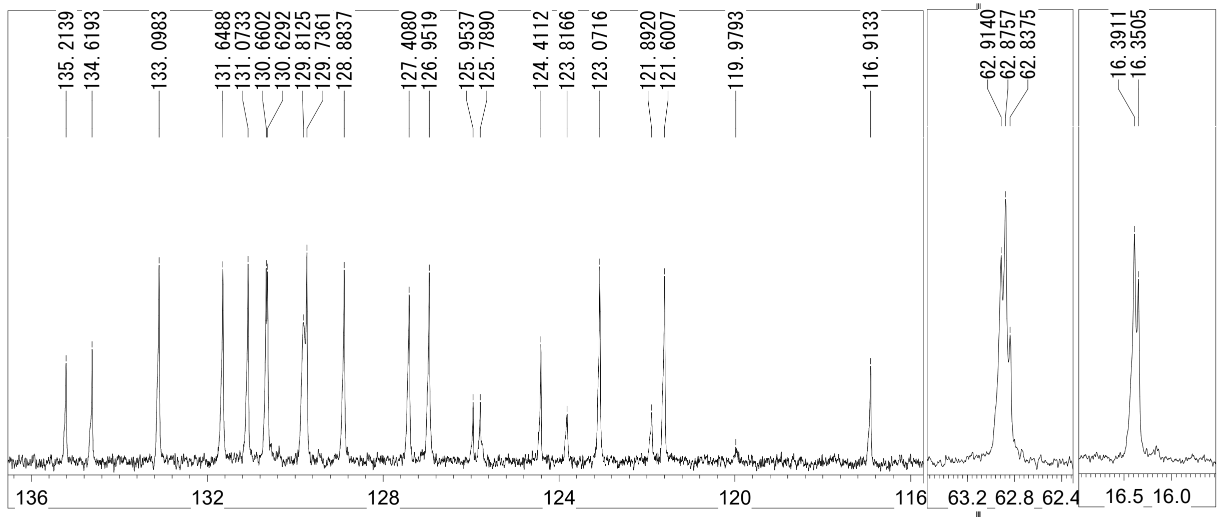

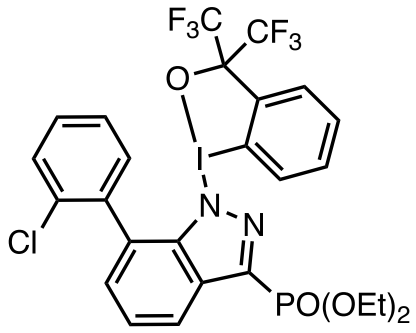

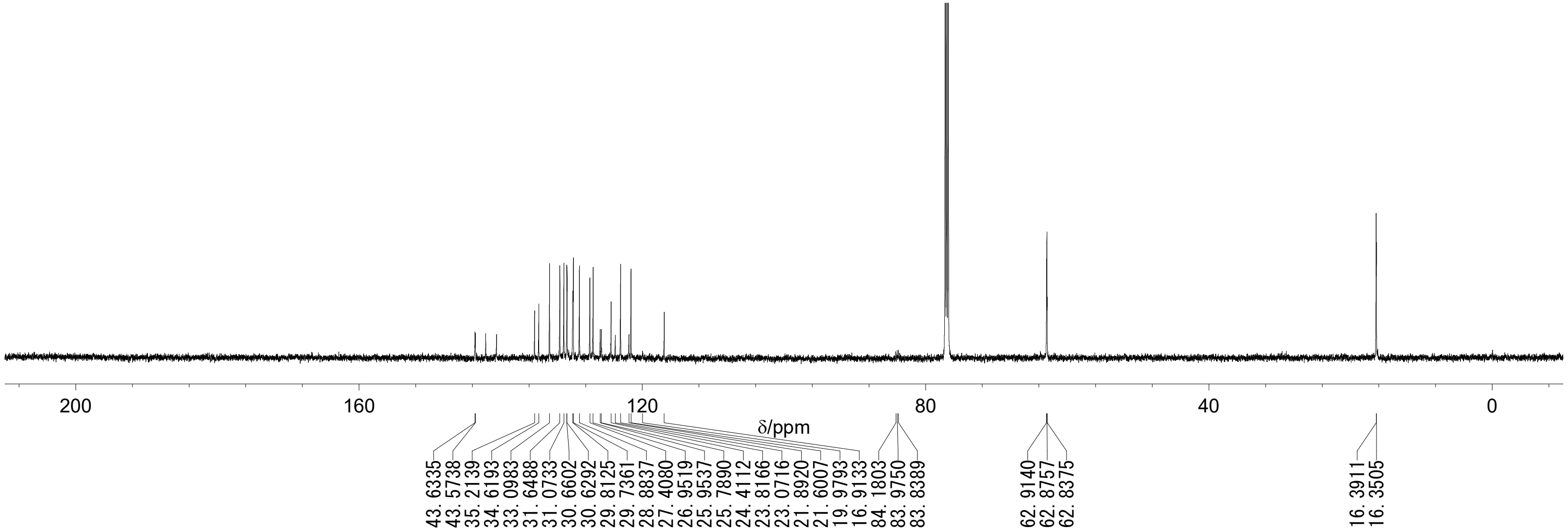

^{1}H NMR spectrum of **4a** (400 MHz, $CDCl_3$)

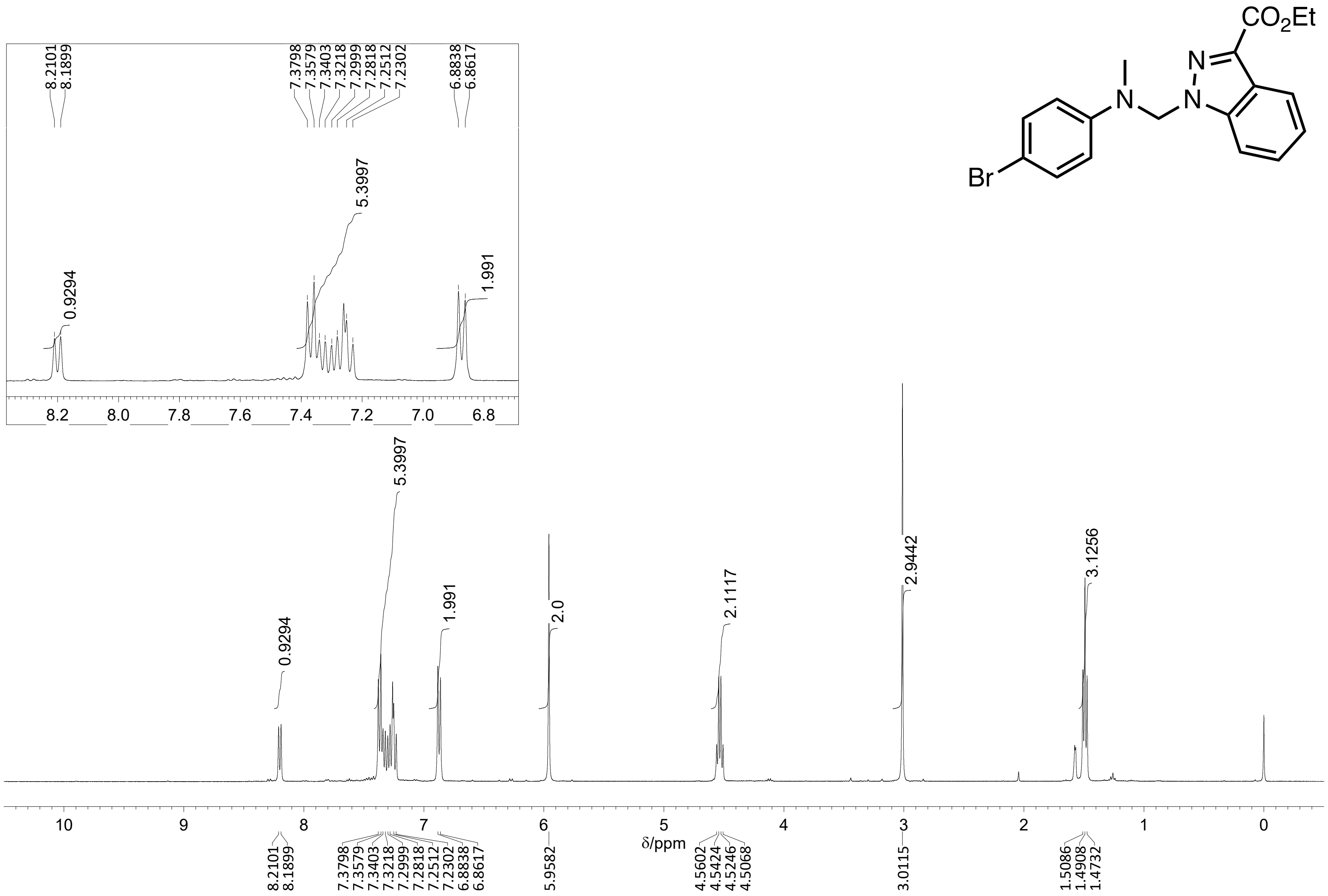

$^{13}C\{^1H\}$ NMR spectrum of **4a** (150 MHz, $CDCl_3$)

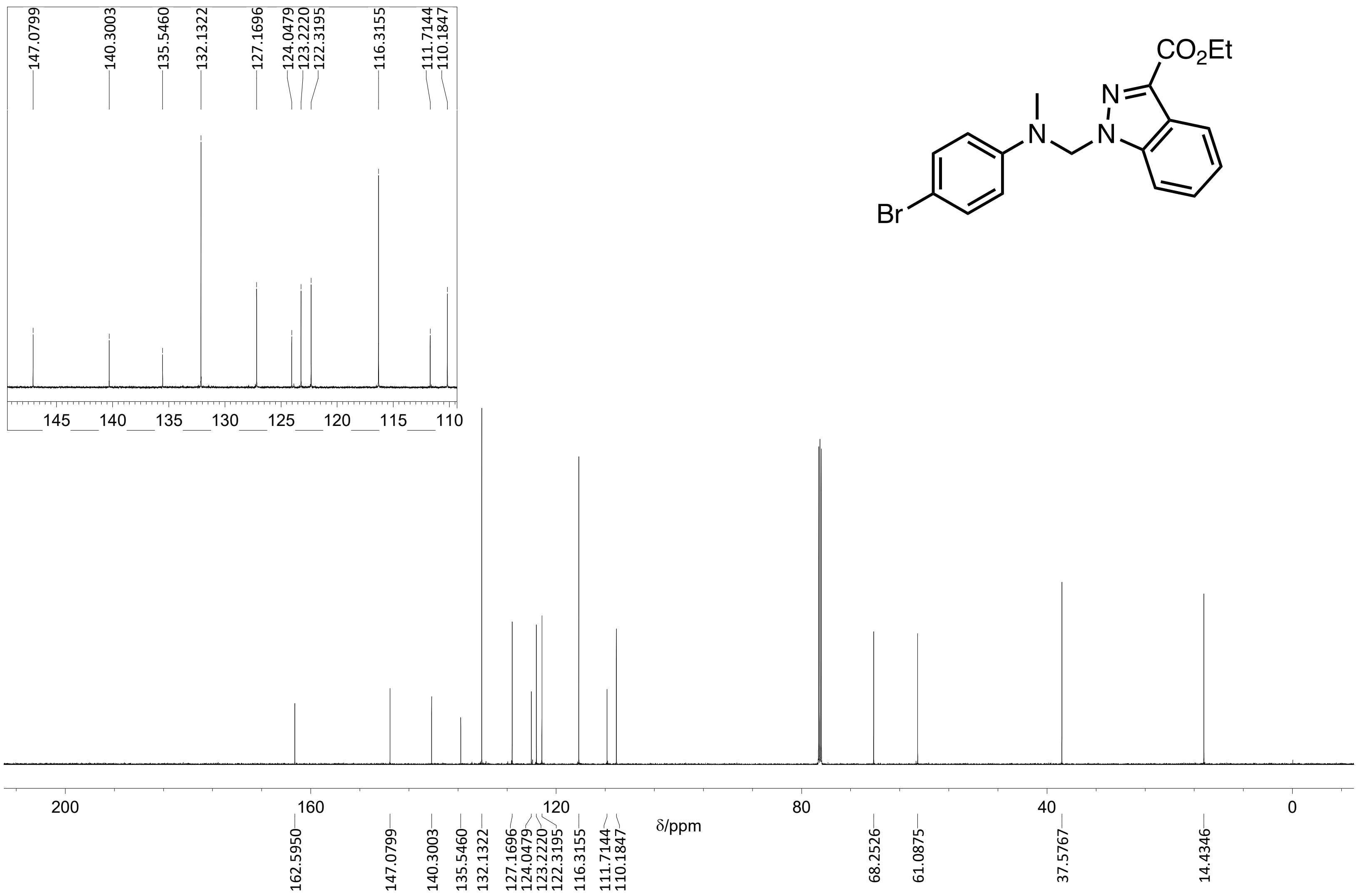

^{1}H NMR spectrum of **4b** (400 MHz, $CDCl_3$)

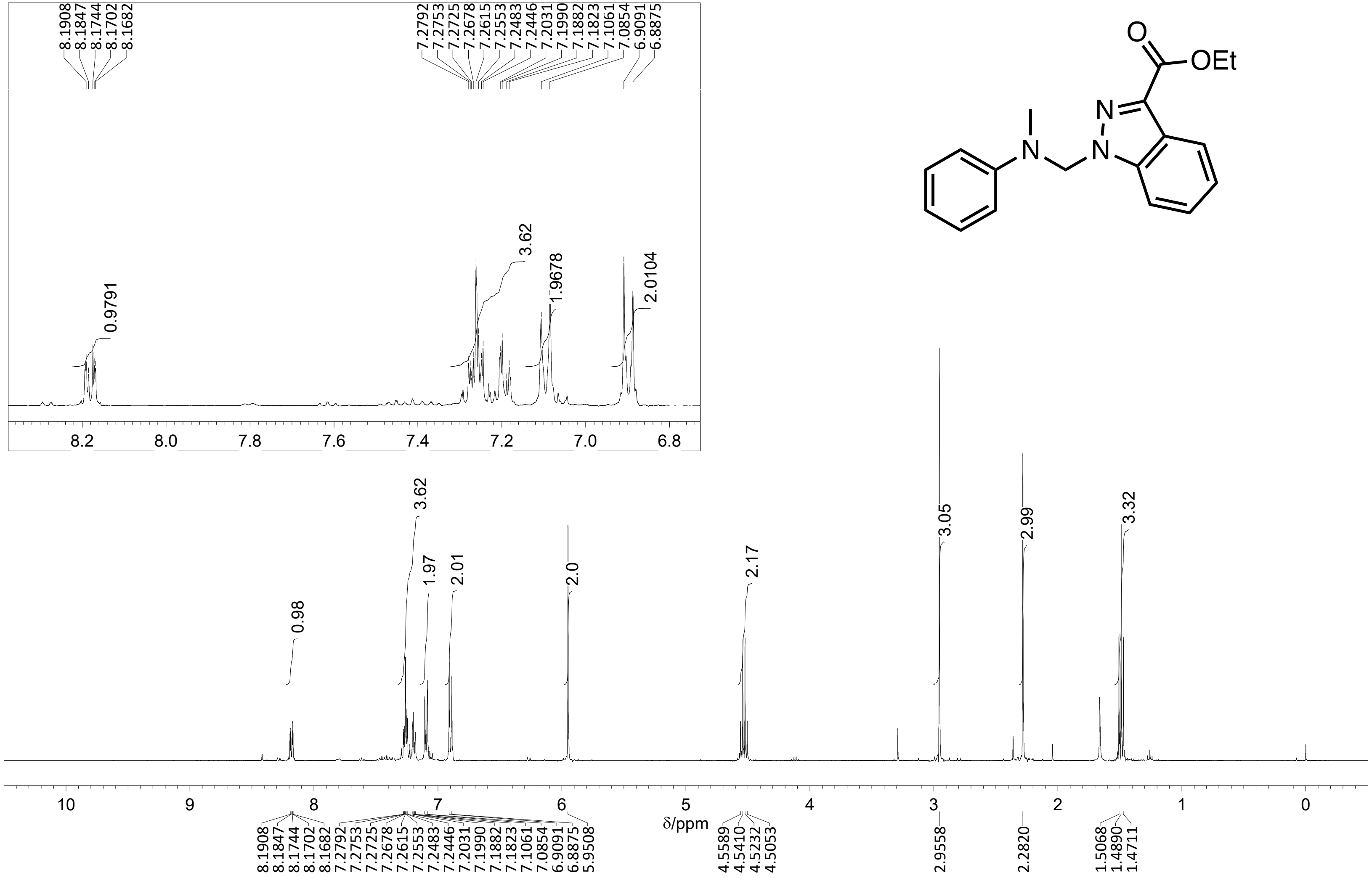

$^{13}C\{^{1}H\}$ NMR spectrum of **4b** (150 MHz, $CDCl_3$)

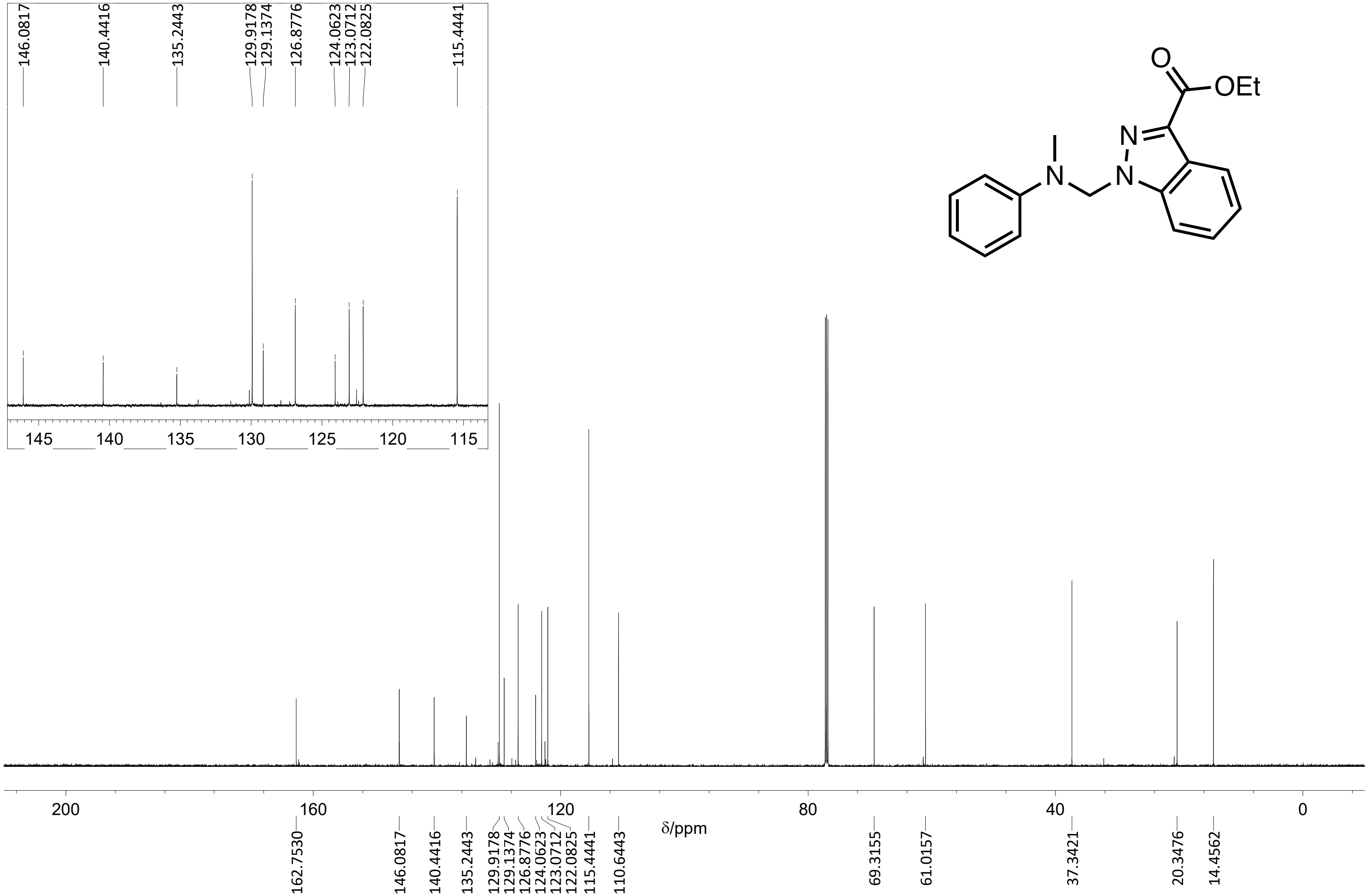

^{1}H NMR spectrum of **4c** (400 MHz, $CDCl_3$)

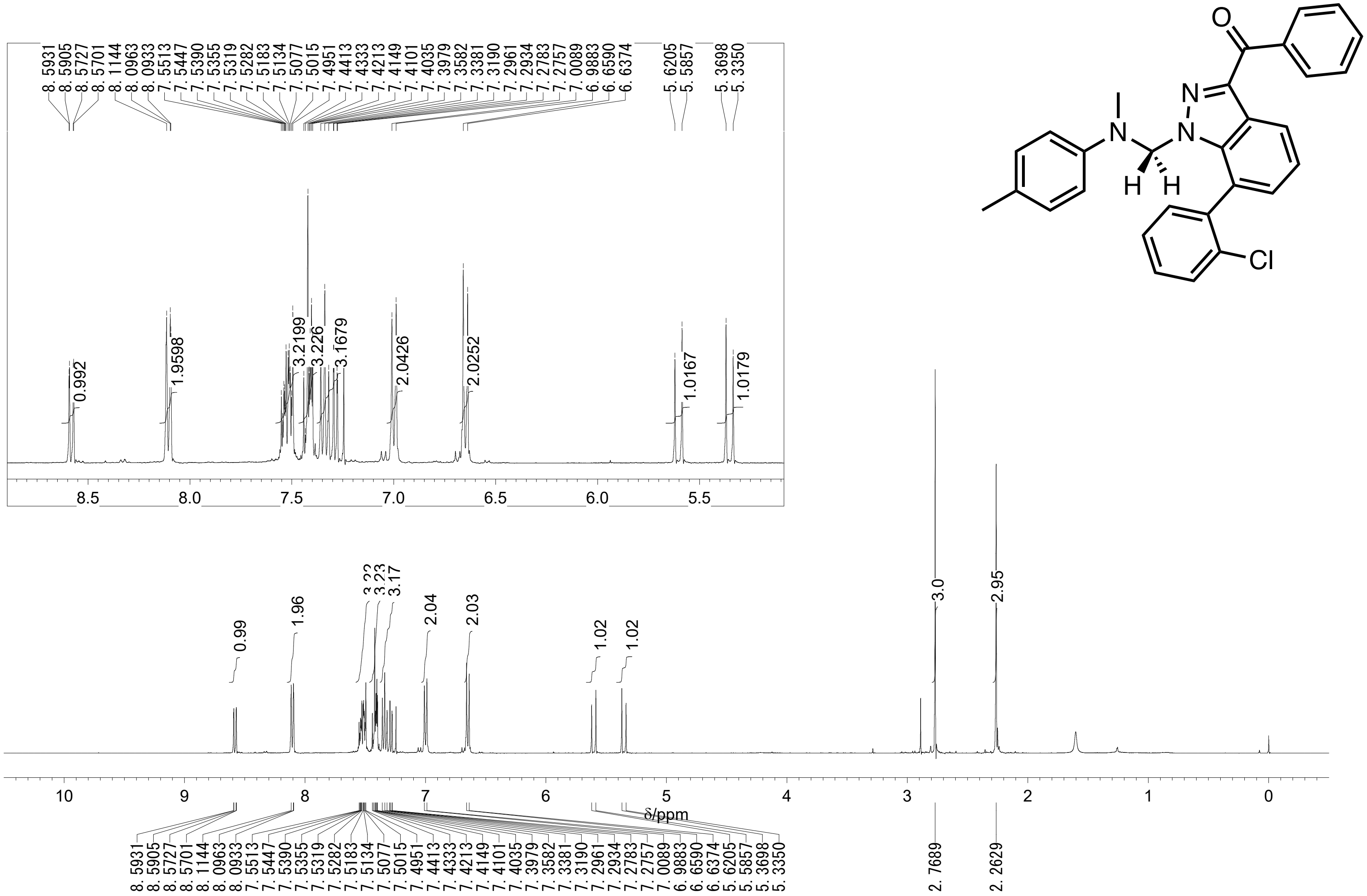

$^{13}C\{^{1}H\}$ NMR spectrum of **4c** (150 MHz, $CDCl_3$)

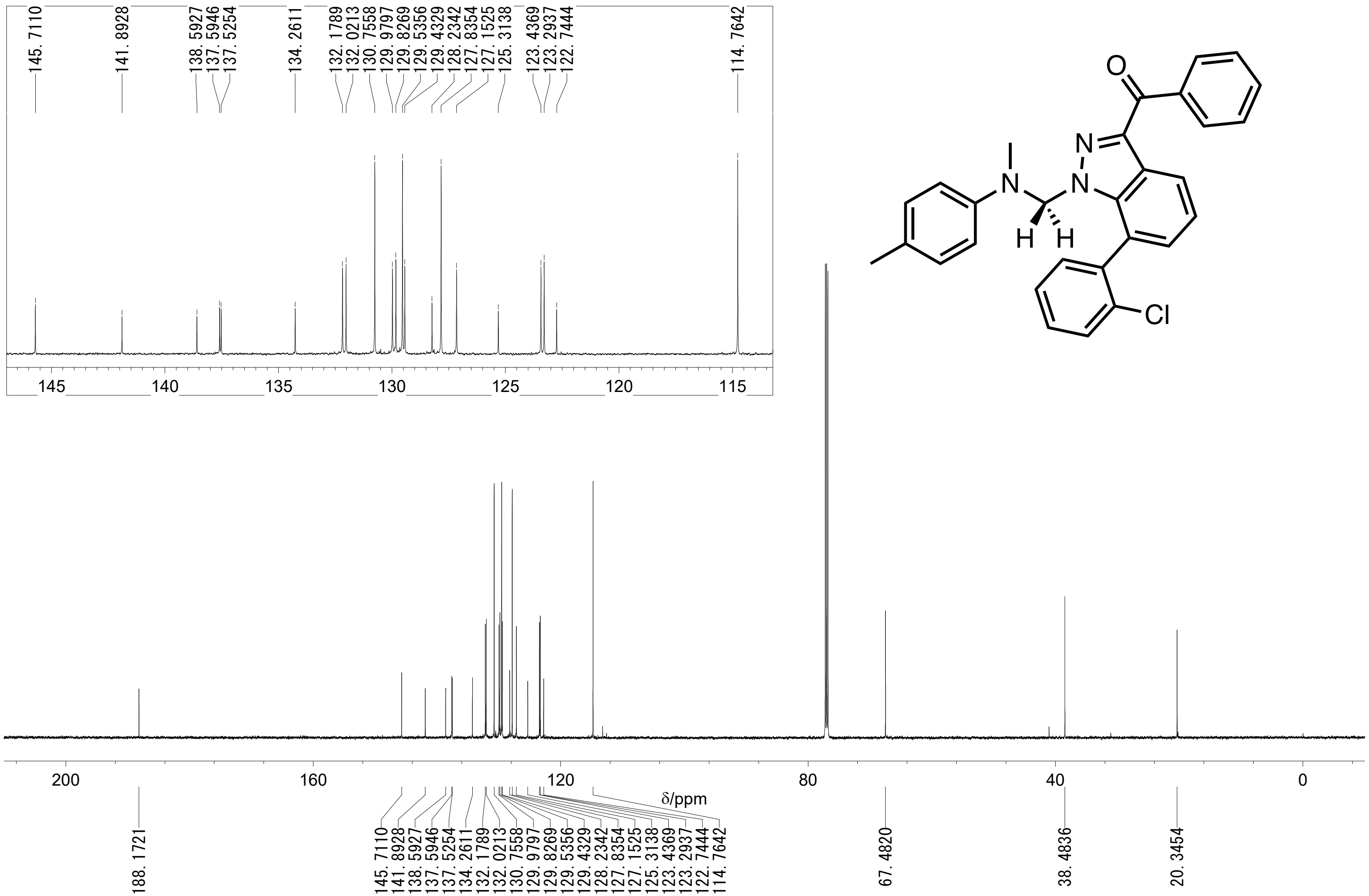

^{1}H NMR spectrum of **4d** (400 MHz, $CDCl_3$)

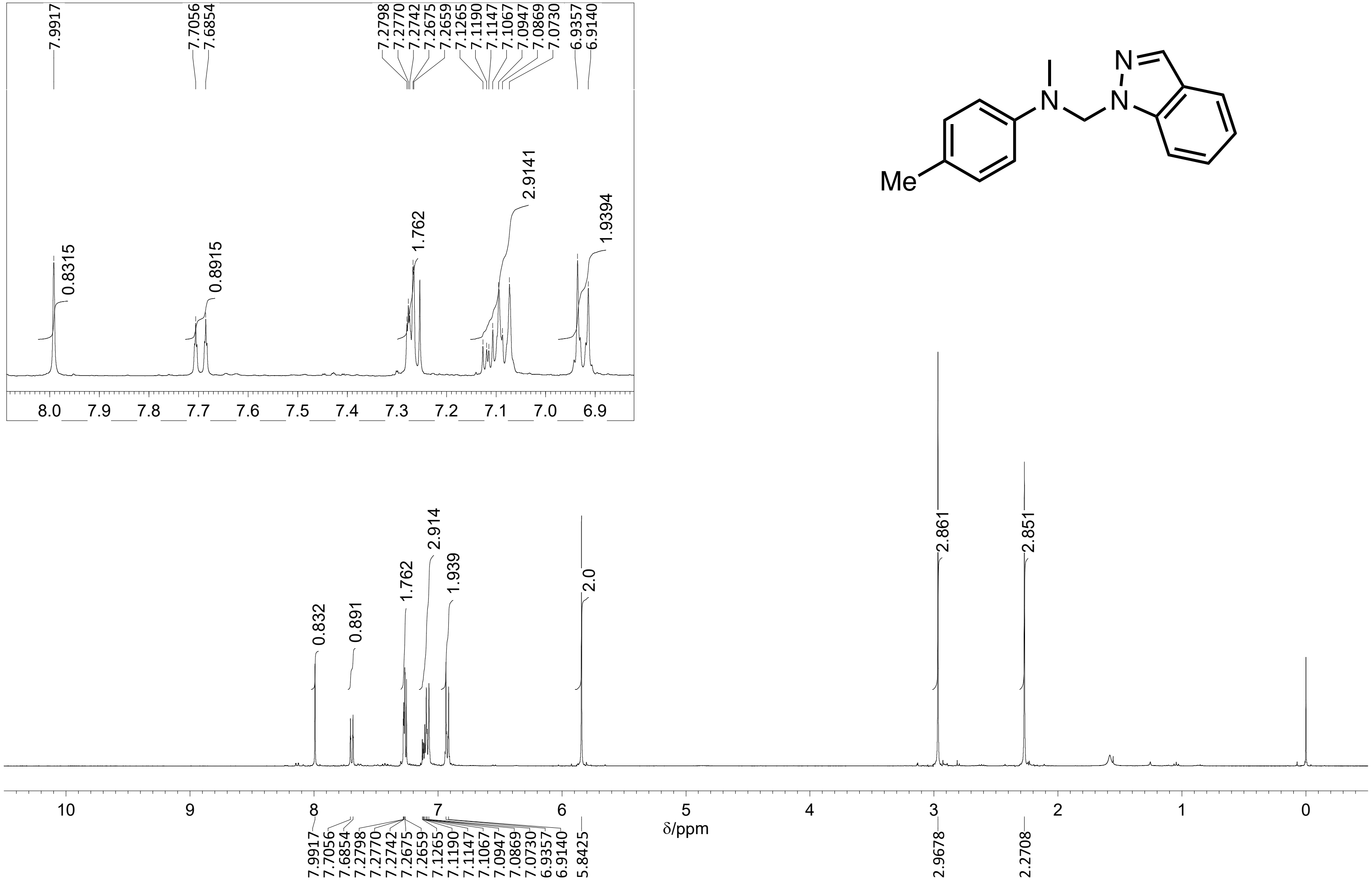

$^{13}C\{^{1}H\}$ NMR spectrum of **4d** (150 MHz, $CDCl_3$)

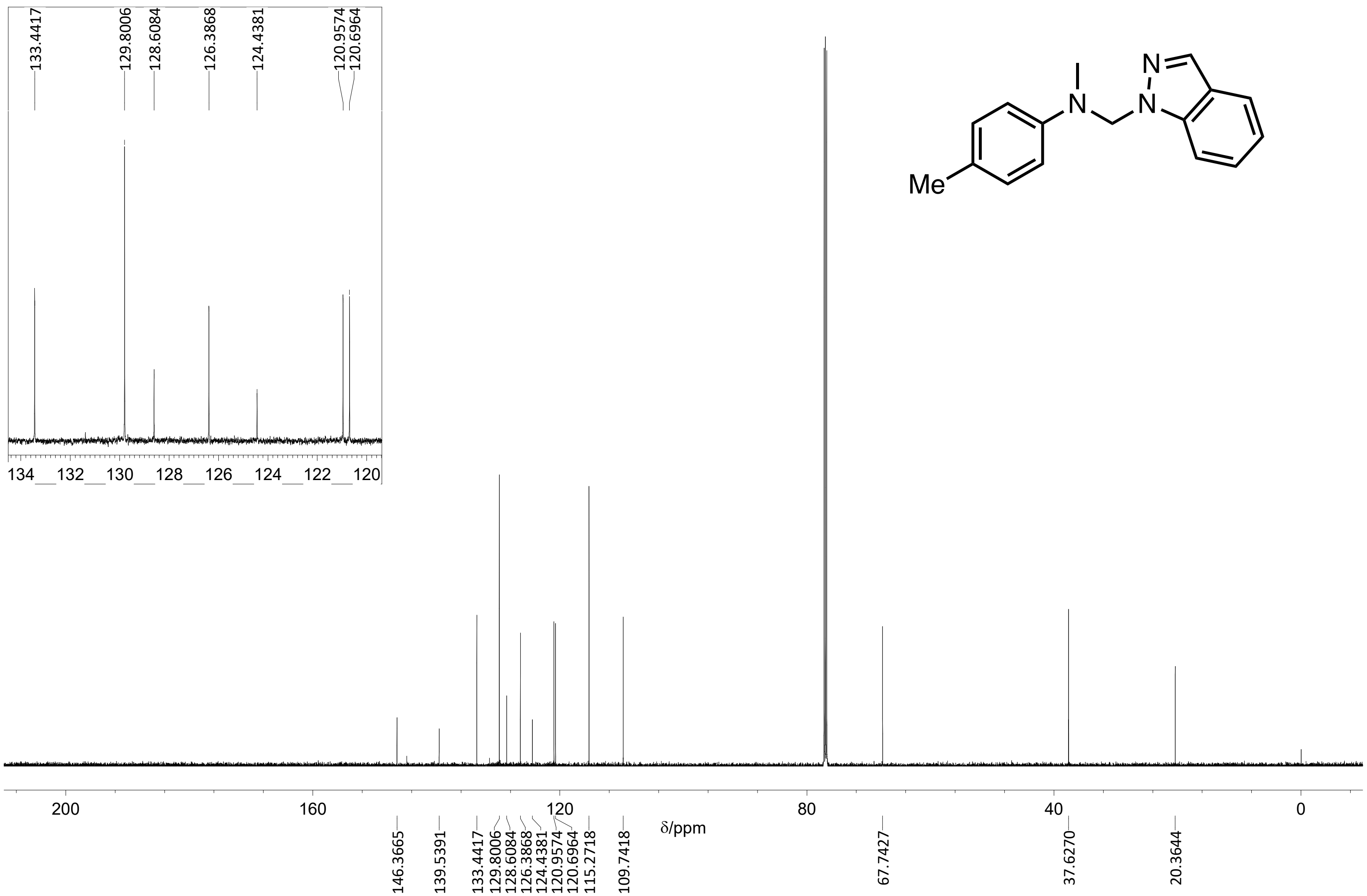

^{1}H NMR spectrum of **3x** (400 MHz, $CDCl_3$)

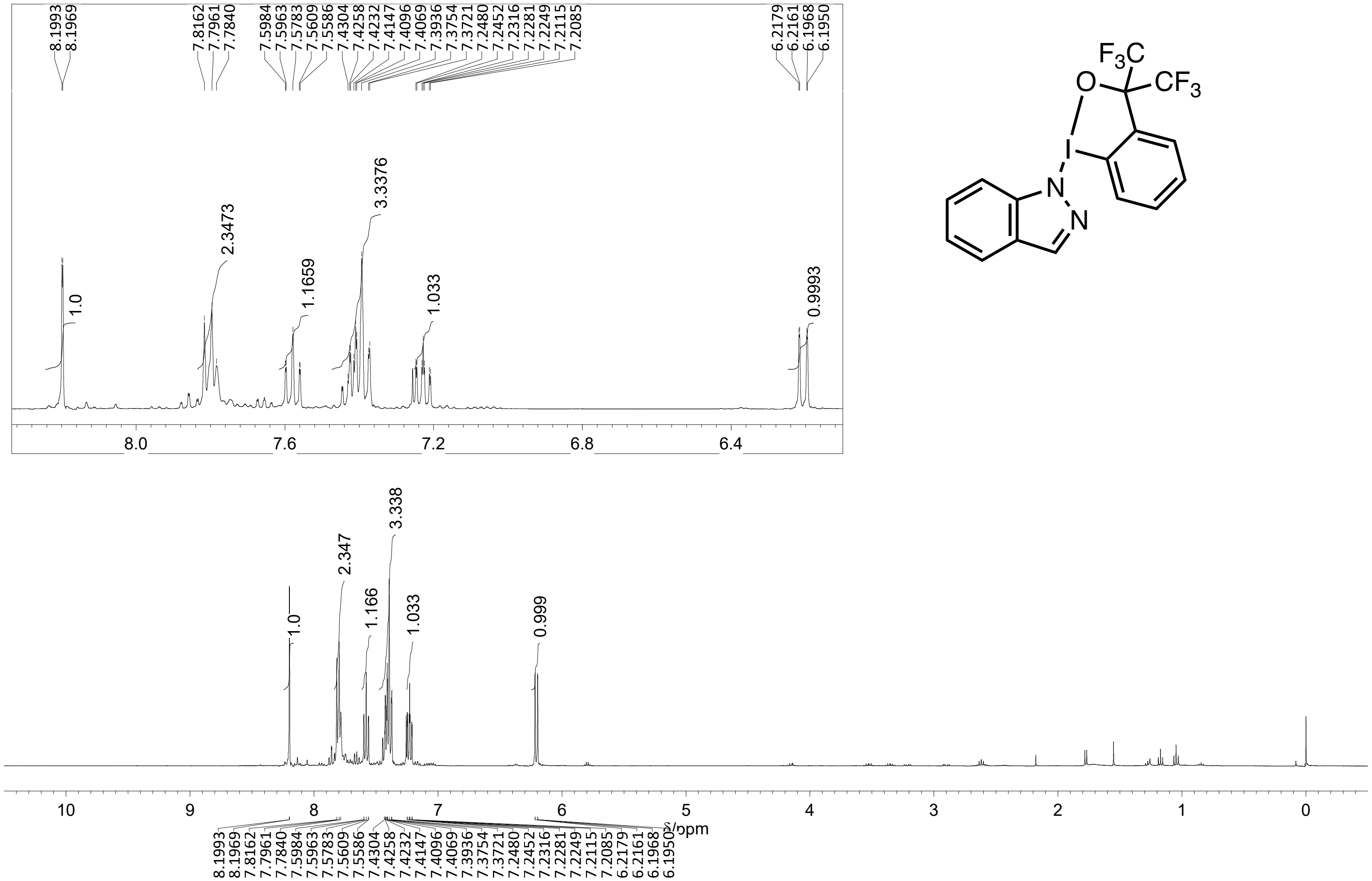

$^{13}C\{^1H\}$ NMR spectrum of **3x** (150 MHz, $CDCl_3$)

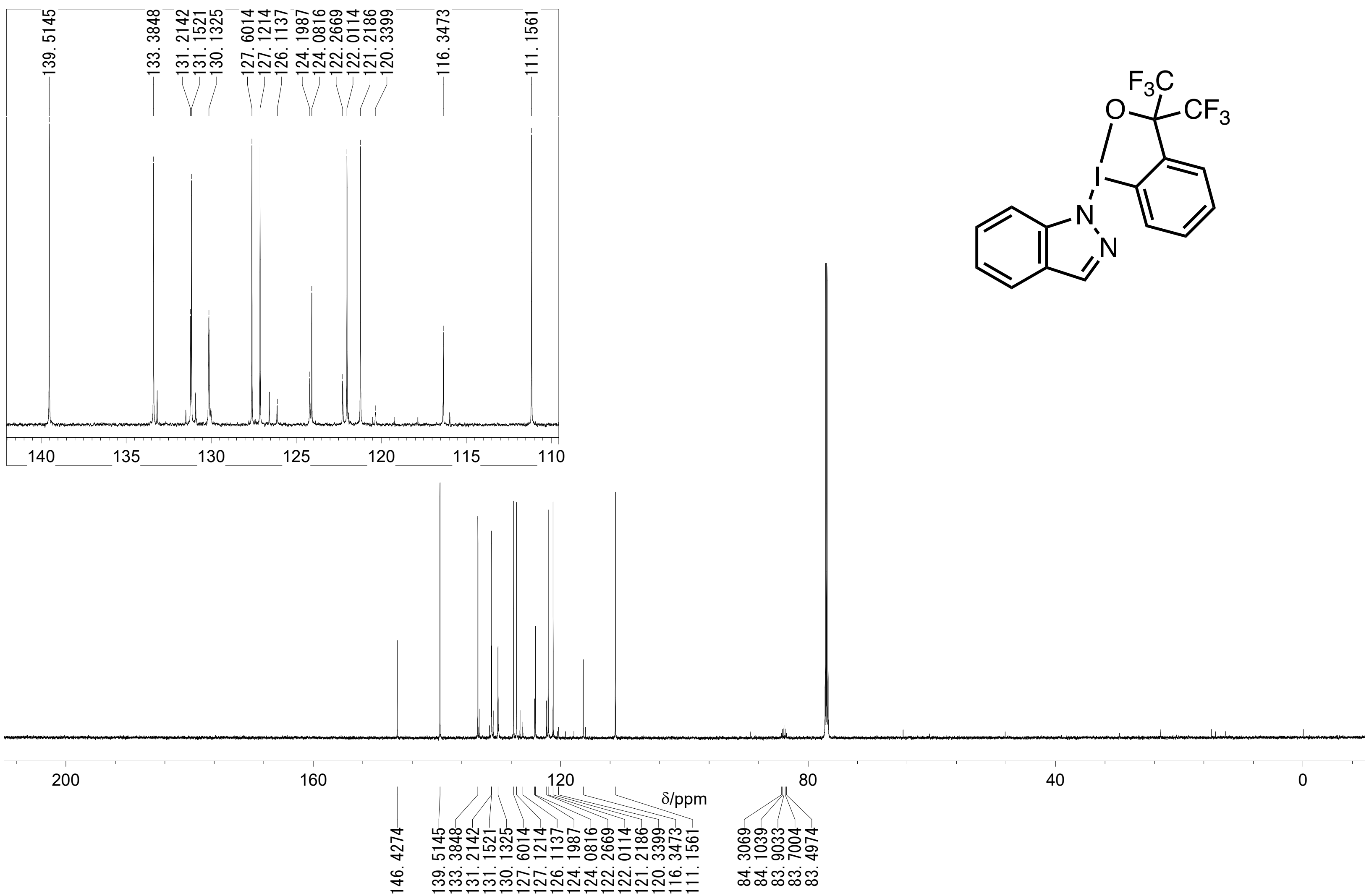

Supplement: SC-016-D5SC00266D-s001 [file SC-016-D5SC00266D-s001.pdf]
